# Risk of Rhabdomyolysis Associated with Dexmedetomidine Use over the Past 10 Years: Insights from the EudraVigilance Database

**DOI:** 10.3390/jpm14090961

**Published:** 2024-09-10

**Authors:** Nunzia Balzano, Annamaria Mascolo, Raffaella Di Napoli, Federica Colapietra, Marina Di Domenico, Annalisa Capuano, Francesca Gargano

**Affiliations:** 1 Department of Experimental Medicine, University of Campania “L. Vanvitelli”, 80138 Naples, Italy; nunzia.balzano@unicampania.it (N.B.); raffaella.dinapoli@unicampania.it (R.D.N.); annalisa.capuano@unicampania.it (A.C.); 2 Campania Regional Centre for Pharmacovigilance and Pharmacoepidemiology, 80138 Naples, Italy; 3 Department of Life Science, Health, and Health Professions, Link Campus University, 00165 Rome, Italy; 4 Department of Precision Medicine, University of Campania “L. Vanvitelli”, 80138 Naples, Italy; federica.colapietra@unicampania.it (F.C.); marina.didomenico@unicampania.it (M.D.D.); 5 Unit of Anesthesia and Intensive Care, Fondazione Policlinico Universitario Campus Bio-Medico, 00128 Rome, Italy; f.gargano@policlinicocampus.it

**Keywords:** dexmedetomidine, rhabdomyolysis, safety

## Abstract

Dexmedetomidine, a selective α2-adrenergic agonist, is favoured in intensive care for its minimal respiratory depression. This study evaluated the reporting frequency of rhabdomyolysis with dexmedetomidine compared to midazolam and propofol using the European pharmacovigilance database Eudravigilance. We conducted an observational, retrospective analysis of Individual Case Safety Reports (ICSRs) from 1 January 2013, to 31 December 2023. Primary and secondary outcomes included the reporting frequencies of rhabdomyolysis and its indicative signs and symptoms, respectively. We retrieved 19,268 ICSRs, of which 364 reported rhabdomyolysis associated with dexmedetomidine (3.8%), midazolam (10.2%), propofol (76.9%), or combinations thereof (9.1%). Dexmedetomidine showed a significantly lower reporting frequency of rhabdomyolysis compared to propofol (ROR, 0.32; 95% CI, 0.19–0.55) but no significant difference compared to midazolam. Subgroup analyses revealed higher frequencies in males, especially with propofol. Despite limitations such as underreporting, our findings suggest dexmedetomidine poses a lower rhabdomyolysis risk than propofol, supporting its safe use for sedation in high-risk patients. It is important to note that due to the retrospective design of this study our findings are indicative of correlations rather than causation. Continuous monitoring and further studies are recommended to validate these results.

## 1. Introduction

Dexmedetomidine, a potent and highly selective α2-adrenergic agonist, has emerged as a pivotal agent in modern anaesthesia. It can be considered an alternative or adjunct to midazolam or propofol in several critical care practices [1,2,3]. Midazolam, a benzodiazepine, is widely used for sedation due to its anxiolytic, amnesic, and hypnotic properties [4]. Propofol, a sedative–hypnotic, is favoured during general anesthesia practice for its rapid onset and short duration of action, enabling faster recovery compared to benzodiazepines [5]. However, both midazolam and propofol are associated with notable adverse effects. Midazolam can cause significant respiratory depression and is linked to the risk of delirium, particularly with prolonged use [4,6]. Propofol is also known for causing dose-dependent hypotension and respiratory depression, and long-term use can lead to propofol infusion syndrome, a rare but serious condition that includes metabolic acidosis, cardiac failure, and rhabdomyolysis [5]. The choice of sedative should be tailored based on the specific needs of each patient and clinical situation. Dexmedetomidine is particularly employed for sedation in intensive care units and procedural environments. With its pharmacological profile, dexmedetomidine offers sedation with minimal respiratory depression, especially when compared to propofol and midazolam [7]. It is indeed preferred when minimal respiratory depression is desired, such as in patients at risk of airway compromise or in those requiring light sedation with the ability to interact with caregivers during procedures [8,9]. Additionally, dexmedetomidine may offer advantages in mechanically ventilated adults and reduce delirium compared to other sedatives, especially benzodiazepines and propofol [10]. Administered intravenously, its mechanism of action involves binding to α2-adrenergic receptors in the brainstem, particularly in the locus coeruleus. This binding modulates noradrenergic pathways, leading to sedative, anxiolytic, and analgesic effects [9,10]. While dexmedetomidine is associated with some adverse effects such as bradycardia and hypotension due to its sympatholytic action [9,11,12], there is no evidence linking it directly to rhabdomyolysis, a condition characterized by the breakdown of skeletal muscle tissue and the release of muscle cell contents into the bloodstream [13]. However, in critically ill patients, prolonged immobility, the use of sedatives, and other factors that impair muscle function and increase the susceptibility to muscle damage can elevate the risk of developing rhabdomyolysis [14]. In this context, it is worth noting that the risk management plan for dexmedetomidine included a safety signal for rhabdomyolysis, identified through a cumulative increase in post-marketing reports [15]. However, clinical trial data showed a very low incidence of rhabdomyolysis with dexmedetomidine, comparable to that of comparators and placebo, with severe cases being rare and manageable [15]. Based on the current data, there is insufficient evidence to establish a causal link between dexmedetomidine and rhabdomyolysis. To improve the knowledge on the safety profile of dexmedetomidine and to have an updated overview of post-marketing safety reports, we decided to conduct a pharmacovigilance study focusing on the reporting frequency of rhabdomyolysis with dexmedetomidine compared to midazolam or propofol using data collected in the European spontaneous reporting system. Midazolam and propofol were chosen as comparators because they are therapeutic alternatives to dexmedetomidine. It is important to note that only correlations and not causation can be inferred from the results due to the retrospective nature of our analysis.

## 2. Materials and Methods

### 2.1. Study Design

Our study is an observational, retrospective, pharmacovigilance study, evaluating the reporting frequency of dexmedetomidine-related rhabdomyolysis compared to midazolam and propofol by analysing the data collected in the European spontaneous reporting system database.

### 2.2. Data Source

The European pharmacovigilance database Eudravigilance (EV) was used for this study. EV is managed by the European Medicine Agency (EMA) and is publicly available online on the EMA website (www.adrreports.eu). All Individual Case Safety Reports (ICSRs) of suspected adverse drug reactions (ADRs) and adverse events following immunization (AEFI), related to authorized drugs or vaccines and sent by healthcare professionals (HCP) and patients/citizens to a European National Competent Authority, are collected in this database. The analysis of EV allows us to uncover safety information from real-world contexts and to highlight new insights on drug safety that may not emerge during pre-marketing clinical trials.

### 2.3. Study Outcomes

The primary outcome was the reporting frequency of rhabdomyolysis. The secondary outcome was the reporting frequency of signs and symptoms of rhabdomyolysis.

### 2.4. ICSRs Selection

We retrieved all ICSRs reporting dexmedetomidine, midazolam, or propofol as suspect drugs over the past 10 years (from 1 January 2013 to 31 December 2023) using the line-listing function of the EV website. In our dataset, we identified potential duplicates and removed them accordingly. The Medical Dictionary for Regulatory Activities (MedDRA) was used to select adverse events. MedDRA is a standardized medical terminology globally used for adverse event classification and organized into five hierarchical levels, listed below from the most detailed to the most generic: lowest level terms (LLT), preferred term (PT), high-level terms (HLT), high-level group terms (HLGT), and system organ class (SOC). For the descriptive and primary analysis, we conducted a preliminary selection of ICSRs by searching for the PT “Rhabdomyolysis” among reported adverse events. Additionally, for the secondary analysis, we considered all ICSRs reporting PTs indicative of rhabdomyolysis based on signs and symptoms consistent with its pathogenesis [13,16,17,18]. The list of PTs considered as indicative of rhabdomyolysis is shown in Supplementary Table S1.

### 2.5. Descriptive Analysis

We classified ICSRs reporting “Rhabdomyolysis” as an adverse event for the suspected drug: dexmedetomidine, midazolam, propofol, or a combination of these sedatives. We analysed data in terms of sex and age group, reporter type (HCP or Non-HCP), country for regulatory purposes (European Economic Area or Non-European Economic Area), adverse events (type, seriousness, and outcome) and treatments (other suspected drugs and concomitant drugs). All ADRs were categorized according to the MedDRA SOC. The seriousness of ADRs was assessed in accordance with the International Council on Harmonization E2D guidelines. Specifically, an ADR was classified as “serious” if it resulted in death, was life threatening, required or prolonged hospitalization, caused persistent or significant disability/incapacity, resulted in a congenital anomaly/congenital disability, or led to other clinically important conditions. When additional criteria were provided for each ADR, we selected the most serious criterion for classification. The outcome of adverse events was considered favourable if it resulted in “recovered/resolved” or “recovering/resolving”. On the contrary, the outcome was classified as unfavourable if it resulted in “recovered/resolved with sequelae”, “not recovered/not resolved”, or “fatal”. All qualitative variables were expressed as numbers and percentages.

### 2.6. Disproportionality Analysis

For the primary disproportionality analysis, the Reporting Odds Ratio (ROR) with its 95% confidence interval (95% CI) were computed to assess the reporting frequency of rhabdomyolysis with dexmedetomidine compared to midazolam, propofol, or combinations of sedatives (dexmedetomidine/midazolam, dexmedetomidine/propofol, midazolam/propofol, and dexmedetomidine/midazolam/propofol). The ROR was calculated as (a/c)/(b/d): “a” is the number of events reported with the drug of interest, “c” is the number of events reported with a comparator, “b” is the number of other events reported with the drug of interest, and “d” is the number of other events reported with a comparator. A secondary disproportionality analysis was computed considering all PTs indicative of rhabdomyolysis (Supplementary Table S1) for the same aforementioned comparisons. Moreover, sub-analyses for primary and secondary outcomes were performed for the following comparisons: midazolam compared to propofol; dexmedetomidine/midazolam compared to a combination of drugs (dexmedetomidine/propofol, midazolam/propofol, and dexmedetomidine/midazolam/propofol); dexmedetomidine/propofol compared to a combination of drugs (midazolam/propofol and dexmedetomidine/midazolam/propofol); and midazolam/propofol compared to dexmedetomidine/midazolam/propofol.

Subgroup analyses were performed to evaluate the reporting frequency of rhabdomyolysis or events indicative of rhabdomyolysis between gender (male vs. female).

At least 3 events must be reported for each drug to perform disproportionality analyses and a *p*-value ≤ 0.05 was applied for statistical significance. Data management and analyses were performed using Excel 365 (Microsoft Office) and R (version 4.2.2, R Development Core Team).

## 3. Results

### 3.1. Descriptive Results

During our study period, we retrieved a total of 19,268 ICSRs from the EV database. Among these, 364 ICSRs reported rhabdomyolysis as an adverse event and dexmedetomidine (N = 14; 3.8%), midazolam (N = 37; 10.2%), propofol (N = 280; 76.9%), or a combination of these sedatives (N = 33; 9.1%) as suspected drugs. In particular, the most reported combination was midazolam/propofol (N = 21; 5.8%). The main demographic and clinical characteristics of ICSRs for all drugs are described in Table 1.

Overall, the majority of the ICSRs referred to adult (N = 247; 67.9%) and male (N = 245; 67.3%) patients. HCPs were the main source of reporting (N = 355; 97.5%). In terms of the primary source country for regulatory purposes, the Non-European Economic Area was the most representative (N = 251; 69.0%).

More than half of cases (N = 226; 62.1%) did not exclusively report dexmedetomidine, midazolam, or propofol as the suspected drug. Moreover, 54.4% of ICSRs (N = 198) presented at least one concomitant medication. Generally, anaesthetics (N = 158; 13.1%) were the most frequently reported concomitant agents, followed by analgesics (N = 84; 7.0%) and psycholeptics (N = 82; 6.8%). The distribution of concomitant drugs is presented in Supplementary Figure S1.

We analysed a total of 1810 ADRs (Supplementary Table S2), of which 20.1% were rhabdomyolysis (N = 364). The 99.2% of rhabdomyolysis events (N = 361) were classified as serious. “Caused/prolonged hospitalisation” for propofol (N = 82; 29.3%), “life threatening” for dexmedetomidine (N = 8; 57.1%), “results in death” for midazolam (N = 12; 32.4%), and “other medically important condition” for combination of sedatives (N = 12; 36.4%) were the most frequently reported seriousness criteria.

Moreover, more than half of rhabdomyolysis events (N = 192; 52.7%) had a favourable outcome, resulting in a complete resolution (N = 117; 32.1%) or resolving (N = 75; 20.6) at the reporting time. Specifics for seriousness and outcome criteria of rhabdomyolysis are presented in Table 2.

The remaining 79.9% of ADRs (N = 1446) were categorized by MedDRA SOCs. In particular, “General disorders and administration site conditions” was the most representative SOC for dexmedetomidine (N = 10; 19.2%) and combination of sedatives (N = 21; 18.3%). On the contrary, the most representative SOC was “Metabolism and nutrition disorders” (N = 231; 22.1%) for propofol and “Nervous system disorders” (N = 38; 16.3%) for midazolam (Table 3).

### 3.2. RORs of Rhabdomyolysis

**Figure 1:**
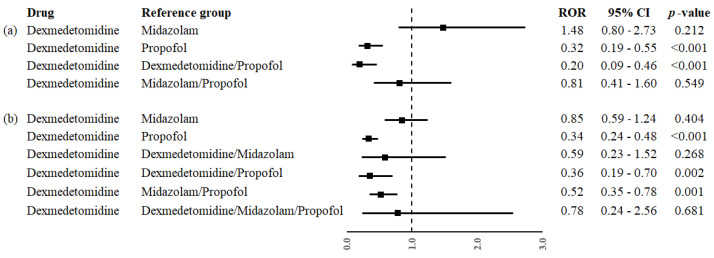
Reporting odds ratio (ROR) of (**a**) rhabdomyolysis and (**b**) all PTs indicative of rhabdomyolysis with dexmedetomidine compared to midazolam, propofol, or sedatives’ combinations. CI, confidence interval.

Dexmedetomidine was associated with a significantly lower reporting frequency of rhabdomyolysis when compared to propofol (ROR, 0.32; 95% CI, 0.19–0.55). A significantly lower reporting frequency of rhabdomyolysis was also observed when dexmedetomidine was compared to the combination of dexmedetomidine/propofol (ROR, 0.20; 95% CI, 0.09–0.46). Conversely, no difference was observed for dexmedetomidine compared to midazolam (ROR, 1.48; 95% CI, 0.80–2.73) and midazolam/propofol (ROR, 0.81; 95% CI, 0.41–1.60). All RORs of primary analysis are reported in Figure 1a.

In the primary sub-analysis, midazolam was associated with a significantly lower reporting frequency of rhabdomyolysis compared to propofol (ROR, 0.22; 95% CI, 0.15–0.31). Moreover, the combination of dexmedetomidine/propofol was associated with a significantly higher reporting frequency of rhabdomyolysis compared to the combination of midazolam/propofol (ROR, 4.10; 95% CI, 1.87–9.00). All RORs of primary sub-analysis are reported in Supplementary Figure S2a.

### 3.3. RORs of All PTs Indicative of Rhabdomyolysis

Dexmedetomidine exhibited a significantly lower reporting frequency of signs and symptoms of rhabdomyolysis compared to propofol (ROR, 0.34; 95% CI, 0.24–0.48). A significantly lower reporting frequency of signs and symptoms of rhabdomyolysis was also observed when dexmedetomidine was compared to the combination of dexmedetomidine/propofol (ROR, 0.36; 95% CI, 0.19–0.70) and midazolam/propofol (ROR, 0.52; 95% CI, 0.35–0.78). No difference was instead observed for all other comparisons. All RORs for secondary analysis are reported in Figure 1b.

In the secondary sub-analysis, midazolam was associated with a significantly lower reporting frequency of signs and symptoms of rhabdomyolysis compared to propofol (ROR, 0.40; 95% CI, 0.33–0.47). No statistically significant difference was observed for all other comparisons. All RORs for secondary sub-analysis are reported in Supplementary Figure S2b.

### 3.4. RORs of Rhabdomyolysis for Gender

A significantly higher reporting frequency of rhabdomyolysis was found in males than females who were administered midazolam (ROR, 2.28; 95% CI, 1.14–4.55) or propofol (ROR, 3.02; 95% CI, 2.31–3.94, Supplementary Figure S3a). However, this significantly higher frequency in males was confirmed only for propofol (ROR, 1.91; 95% CI, 1.62–2.26, Supplementary Figure S3b) when the analysis included all PTs indicative of rhabdomyolysis. For dexmedetomidine, no statistically significant estimate was found in both analyses (Supplementary Figure S3a,b).

## 4. Discussion

Our study aimed to evaluate the reporting frequency of rhabdomyolysis with dexmedetomidine using data from the large EV database. The risk of rhabdomyolysis poses a significant concern for critically ill patients receiving sedation in the intensive care unit, particularly if they are on multiple medications or have underlying conditions predisposing them to muscle breakdown [19]. Rhabdomyolysis is a serious medical condition characterized by the breakdown of muscle tissue, leading to the release of myoglobin, sarcoplasmic proteins (creatine kinase, lactate dehydrogenase, aldolase, alanine, and aspartate aminotransferase), electrolytes, and other muscle cell components into the bloodstream. Symptoms can range from mild muscle discomfort to life-threatening complications, including muscle weakness, muscle pain, localized swelling and tenderness in affected muscle groups, fatigue, malaise, dark red or brown-coloured urine due to myoglobinuria, and decreased urine output [13,16,17,18].

In our primary and secondary disproportionality analyses, we observed a lower reporting frequency of rhabdomyolysis with dexmedetomidine compared to propofol. Propofol-induced rhabdomyolysis is well documented in the literature, especially in patients undergoing prolonged infusions or receiving high doses of the drug [5]. Zhou Z. et al. described a case of fatal myolysis affecting both skeletal and cardiac muscles following anesthesia induction with propofol in a 54-year-old woman who underwent a hysterectomy. The evidence of rhabdomyolysis was confirmed by high levels of blood myoglobin, creatinine kinase, and aspartate aminotransferase [20]. Rhabdomyolysis may also occur after propofol administration as a consequence of propofol infusion syndrome. This is a rare multifactorial condition characterized by metabolic acidosis, hyperkalemia, lipidemia, cardiac failure, and rhabdomyolysis [21]. Propofol infusion syndrome is more commonly reported in children than in adults. This is often attributed to the fact that children may require relatively higher doses of propofol for sedation or anesthesia compared to adults [21]. The risk of rhabdomyolysis with propofol could be attributed to its potential metabolic effects, which may pose a higher risk of muscle injury and rhabdomyolysis compared to dexmedetomidine. Several mechanisms have been proposed to explain propofol-induced rhabdomyolysis. Propofol can cause direct damage to muscle cells as the lipid emulsion used to deliver propofol can accumulate in muscle tissues, breaking cellular integrity and function. It can disrupt mitochondrial function, leading to reduced ATP production and increased reactive oxygen species. Additionally, propofol can interfere with calcium regulation, causing prolonged muscle contraction, and impair energy metabolism, making muscle cells more vulnerable to damage [22,23,24].

Conversely, there is no evidence in the literature suggesting a risk of rhabdomyolysis associated with dexmedetomidine over the years. Dexmedetomidine is a selective α2-adrenergic agonist. Its primary mechanism of action involves binding to α2-receptors in the central nervous system, leading to decreased sympathetic outflow [25]. Peripheral α2-adrenoreceptors are both prejunctional and postjunctional receptors. While prejunctional α2-autoreceptors results in a sympatholytic action, postjunctional α2-adrenoreceptors mediate smooth muscle contraction, platelet aggregation, and inhibition of insulin secretion [26]. In the skeletal muscle, α2-adrenoreceptors are present on both small and large vessels, and together with α1 receptors participate to the adrenergic regulation of large arterioles and venules [27,28]. Moreover, they seem to exert a predominant effect on precapillary arterioles [27]. Based on these mechanisms, the effects of dexmedetomidine on peripheral vasculature are biphasic and dose-dependent: at a low dose and slow infusion rate, it causes vasodilation due to the reduction in sympathetic nervous activity (sympatholysis), while at a high dose and fast infusion rate, it leads to vasoconstriction through direct action on vascular smooth muscles [9,29]. In fact, dexmedetomidine can initially induce a transient increase in blood pressure and a reflex decrease in heart rate, followed later by a hypotensive effect related to its sympatholytic action [9]. However, the role of these vascular pharmacodynamic effects in skeletal muscle integrity is unknown. Moreover, α2-adrenoceptors are on the prejunctional side of the postganglionic cholinergic nerve–smooth muscle junction, where they reduce the release of acetylcholine. In this regard, dexmedetomidine was demonstrated to reduce cholinergic electrical field stimulation-induced contractions and acetylcholine levels [30]. Another potential protective effect of dexmedetomidine is related to its anti-inflammatory properties [9], which can mitigate muscle inflammation and damage, protect muscle tissue, and prevent complications associated with muscle breakdown. Dexmedetomidine can indeed reduce serum levels of several inflammation factors, including interleukin-6, tumour necrosis factor-α, cortisol, S100 calcium-binding protein-β, and superoxide dismutase [31].

Finally, our results did not show a statistically significant difference in the reporting frequency of rhabdomyolysis when dexmedetomidine was compared to midazolam. Moreover, midazolam was found to have a significantly lower reporting frequency of rhabdomyolysis compared to propofol. In the literature, the evidence on midazolam-induced rhabdomyolysis is meagre. A case report described potential risks when midazolam is associated with atorvastatin in a patient with chronic liver disease who underwent gastric endoscopy [32], thus suggesting muscle damage only in presence of other contributing factors. The lower risk with midazolam may also be linked to the different formulation of this drug. Unlike propofol, midazolam is water-soluble and does not require a lipid vehicle for administration, thus not having direct toxic effects on muscle tissues [4].

Despite any causal conclusion, the association of rhabdomyolysis needs to be considered also in the context of concomitant medications. We found that more than half of cases reported other concomitant drugs, highlighting the importance of considering drug interactions in the risk evaluation. The concomitant use of anaesthetics, analgesics, and psycholeptics can indeed contribute to an overall higher risk of adverse events, including rhabdomyolysis [33]. This underscores the need for the careful monitoring of patients receiving multiple sedatives and for considering potential drug interactions in the treatment plan.

In our dataset, rhabdomyolysis cases are more frequently reported in adult males. While the literature has not consistently shown a significant gender discrepancy in the overall incidence, there is evidence indicating that adult men tend to have a higher incidence of exertional rhabdomyolysis compared to women. This difference is often attributed to several factors, including higher muscle mass and differences in hormonal profiles between males and females [34,35,36].

Rhabdomyolysis is included in the Important Medical Event List (IME List) of the EMA. This list comprises medical events classified as serious, providing a standardized reference for the classification and reporting of adverse events based on their clinical significance [37]. However, we found that 0.8% of rhabdomyolysis cases were reported as not serious in our dataset. This discrepancy indicates an error in the seriousness classification of these cases, suggesting a limitation in the pharmacovigilance database.

More than half of the rhabdomyolysis events had a favourable outcome. These findings indicate that, in spite of the significant risk associated with rhabdomyolysis and the use of sedatives in intensive care, timely and appropriate management can lead to positive outcomes in the majority of cases [16]. This emphasizes the importance of early recognition and treatment of rhabdomyolysis to improve patient outcomes.

### Strength and Limitations

Our pharmacovigilance study plays a crucial role in improving the knowledge of the safety profile of sedatives commonly used in daily clinical practice. Pharmacovigilance databases like EV are useful to identify a possible association between a medicine and an adverse event by collecting real-world data from different patient populations. Indeed, EV is a large database collecting post-marketing safety reports from all European Countries. It supports regulatory agencies for the identification of new safety signals for medicines authorized in Europe, including rare adverse events, not identifiable during the pre-marketing phase. However, as with any pharmacovigilance study, our research has limitations. Underreporting and reporting bias may lead to incomplete data. Additionally, variability in data quality and the difficulty of controlling confounding factors can influence our findings. Another significant limitation of this study is its retrospective design, which inherently limits the ability to establish causative relationships between dexmedetomidine use and rhabdomyolysis. Unlike prospective studies or randomized controlled trials, which can be designed to minimize bias and better control for confounding factors, our study relies on spontaneous reports. This retrospective approach means that while we can identify correlations or associations between dexmedetomidine and rhabdomyolysis, we cannot definitively establish causation. Despite these challenges, pharmacovigilance remains essential for continuous post-market surveillance. Ongoing efforts are needed to enhance the spontaneous reporting system, improve data quality, and refine analytical methods to bolster their effectiveness.

## 5. Conclusions

In conclusion, our results showed significant differences in the reporting frequency of rhabdomyolysis among dexmedetomidine, midazolam, and propofol, with dexmedetomidine being associated with a lower reporting frequency compared to propofol. This supports the use of dexmedetomidine as a safe sedative option in terms of rhabdomyolysis risk, especially in patients requiring prolonged sedation or those with risk factors for rhabdomyolysis. However, as with any drug, particularly those affecting cardiovascular and neurological functions, it remains crucial to continuously monitor patients for adverse events and adjust therapy based on individual patient needs. Moreover, considering our study limitations, including its retrospective design, caution should be exercised when interpreting these results. Further studies, employing more rigorous study designs, are necessary to confirm these findings and provide a more comprehensive understanding of the safety profile of dexmedetomidine.

## Figures and Tables

**Table 1:** Characteristics of Individual Case Safety Reports (ICSRs) reporting rhabdomyolysis with dexmedetomidine, midazolam, propofol, or combinations of these sedatives recognized in the EudraVigilance spontaneous reporting system from 1 January 2013 to 31 December 2023.

	DEX (N = 14)	MID (N = 37)	PRO (N = 280)	DEX/MID (N = 2)	DEX/PRO (N = 9)	MID/PRO (N = 21)	DEX/MID/PRO (N = 1)	Overall (N = 364)
Age								
0–1 Month	-	-	1 (0.4)	-	-	-	-	1 (0.3)
2 Months–2 Years	1 (7.1)	2 (5.4)	3 (1.1)	1 (50.0)	-	-	-	7 (1.9)
3–11 Years	-	-	20 (7.1)	-	-	1 (4.8)	-	21 (5.8)
12–17 Years	1 (7.1)	5 (13.5)	15 (5.4)	-	-	1 (4.8)	-	22 (6.0)
18–64 Years	9 (64.3)	21 (56.8)	196 (70.0)	1 (50.0)	7 (77.8)	13 (61.9)	-	247 (67.9)
65–85 Years	2 (14.3)	9 (24.3)	36 (12.9)	-	-	5 (23.8)	-	52 (14.3)
More than 85 Years	-	-	1 (0.4)	-	-	-	-	1 (0.3)
Not Specified	1 (7.1)	-	8 (2.9)	-	2 (22.2)	1 (4.8)	1 (100)	13 (3.6)
Sex								
Female	4 (28.6)	12 (32.4)	77 (27.5)	1 (50.0)	3 (33.3)	5 (23.8)	-	102 (28.0)
Male	9 (64.3)	24 (64.9)	191 (68.2)	1 (50.0)	4 (44.4)	15 (71.4)	1 (100)	245 (67.3)
Not Specified	1 (7.1)	1 (2.7)	12 (4.3)	-	2 (22.2)	1 (4.8)	-	17 (4.7)
Source								
Healthcare Professional	14 (100)	31 (83.8)	278 (99.3)	2 (100)	9 (100)	20 (95.2)	1 (100)	355 (97.5)
Non-Healthcare Professional	-	6 (16.2)	1 (0.4)	-	-	-	-	7 (1.9)
Not Specified	-	-	1 (0.4)	-	-	1 (4.8)	-	2 (0.5)
Country								
European Economic Area	2 (14.3)	15 (40.5)	90 (32.1)	-	1 (11.1)	5 (23.8)	-	113 (31.0)
Non-European Economic Area	12 (85.7)	22 (59.5)	190 (67.9)	2 (100)	8 (88.9)	16 (76.2)	1 (100)	251 (69.0)
Suspects								
1	7 (50.0)	3 (8.1)	128 (45.7)	-	-	-	-	138 (37.9)
2	2 (14.3)	6 (16.2)	50 (17.9)	-	6 (66.7)	1 (4.8)	-	65 (17.9)
3	-	6 (16.2)	35 (12.5)	-	2 (22.2)	2 (9.5)	-	45 (12.4)
4	2 (14.3)	3 (8.1)	30 (10.7)	-	1 (11.1)	1 (4.8)	-	37 (10.2)
≥5	3 (21.4)	19 (51.4)	37 (13.2)	2 (100)	-	17 (81.0)	1 (100)	79 (21.7)
Concomitants								
1	-	-	-	-	-	1 (4.8)	21 (7.5)	22 (6.0)
2	3 (21.4)	-	-	-	2 (5.4)	1 (4.8)	25 (8.9)	31 (8.5)
3	-	1 (50.0)	-	1 (11.1)	1 (2.7)	3 (14.3)	19 (6.8)	25 (6.9)
4	1 (7.1)	-	-	-	2 (5.4)	-	19 (6.8)	22 (6.0)
≥5	2 (14.3)	1 (50.0)	1 (100)	3 (33.3)	13 (35.1)	5 (23.8)	73 (26.1)	98 (26.9)
Not reported	8 (57.1)	-	-	5 (55.6)	19 (51.4)	11 (52.4)	123 (43.9)	166 (45.6)

DEX: dexmedetomidine; MID: midazolam; PRO: propofol; DEX/MID: combinations of dexmedetomidine and midazolam; DEX/PRO: combinations of dexmedetomidine and propofol; MID/PRO: combinations of midazolam and propofol; DEX/MID/PRO: combinations of dexmedetomidine, midazolam, and propofol.

**Table 2:** Seriousness and outcome criteria of rhabdomyolysis for dexmedetomidine, midazolam, propofol, or combinations of these drugs recognized in the EudraVigilance spontaneous reporting system from 1 January 2013 to 31 December 2023.

	DEX (N = 14)	MID (N = 37)	PRO (N = 280)	DEX/MID (N = 2)	DEX/PRO (N = 9)	MID/PRO (N = 21)	DEX/MID/PRO (N = 1)	Overall (N = 364)
Seriousness								
Not serious	-	1 (2.7)	1 (0.4)	-	-	1 (4.8)	-	3 (0.8)
Caused/Prolonged Hospitalization	1 (7.1)	10 (27.0)	82 (29.3)	2 (100)	-	5 (23.8)	-	100 (27.5)
Disabling	-	-	6 (2.1)	-	-	-	-	6 (1.6)
Life Threatening	8 (57.1)	5 (13.5)	68 (24.3)	-	-	6 (28.6)	1 (100)	88 (24.2)
Results in Death	2 (14.3)	12 (32.4)	54 (19.3)	-	3 (33.3)	3 (14.3)	-	74 (20.3)
Other Medically Important Condition	3 (21.4)	9 (24.3)	69 (24.6)	-	6 (66.7)	6 (28.6)	-	93 (25.5)
Outcome								
Recovered/Resolved	5 (35.7)	11 (29.7)	94 (33.6)	-	-	7 (33.3)	-	117 (32.1)
Recovering/Resolving	4 (28.6)	5 (13.5)	63 (22.5)	-	-	3 (14.3)	-	75 (20.6)
Recovered/Resolved With Sequelae	-	1 (2.7)	3 (1.1)	-	-	-	1 (100)	5 (1.4)
Not Recovered/Not Resolved	-	1 (2.7)	19 (6.8)	1 (50.0)	-	-	-	21 (5.8)
Fatal	2 (14.3)	12 (32.4)	54 (19.3)	-	3 (33.3)	3 (14.3)	-	74 (20.3)
Unknown	3 (21.4)	7 (18.9)	47 (16.8)	1 (50.0)	6 (66.7)	8 (38.1)	-	72 (19.8)

DEX: dexmedetomidine; MID: midazolam; PRO: propofol; DEX/MID: combinations of dexmedetomidine and midazolam; DEX/PRO: combinations of dexmedetomidine and propofol; MID/PRO: combinations of midazolam and propofol; DEX/MID/PRO: combinations of dexmedetomidine, midazolam, and propofol.

**Table 3:** Distribution of other adverse events reported in Individual Case Safety Reports (ICSRs) with rhabdomyolysis and dexmedetomidine, midazolam, propofol, or combinations of these drugs retrieved from the EudraVigilance spontaneous reporting system from 1 January 2013 to 31 December 2023, categorized by MedDRA system organ class (SOC).

	DEX (N = 52)	MID (N = 233)	PRO (N = 1046)	DEX/MID (N = 18)	DEX/PRO (N = 9)	MID/PRO (N = 86)	DEX/MID/PRO (N = 2)	Overall (N = 1446)
SOC								
Blood and lymphatic system disorders	2 (3.8)	9 (3.9)	22 (2.1)	-	-	2 (2.3)	-	35 (2.4)
Cardiac disorders	1 (1.9)	10 (4.3)	97 (9.3)	6 (33.3)	1 (11.1)	6 (7.0)	-	121 (8.4)
Congenital, familial, and genetic disorders	-	-	1 (0.1)	-	-	-	-	1 (0.1)
Ear and labyrinth disorders	-	-	1 (0.1)	-	-	-	-	1 (0.1)
Endocrine disorders	-	1 (0.4)	2 (0.2)	-	-	-	-	3 (0.2)
Eye disorders	-	6 (2.6)	1 (0.1)	-	-	-	-	7 (0.5)
Gastrointestinal disorders	2 (3.8)	5 (2.1)	22 (2.1)	-	-	-	-	29 (2.0)
General disorders and administration site conditions	10 (19.2)	31 (13.3)	109 (10.4)	1 (5.6)	5 (55.6)	14 (16.3)	1 (50.0)	171 (11.8)
Hepatobiliary disorders	-	4 (1.7)	38 (3.6)	-	-	2 (2.3)	-	44 (3.0)
Infections and infestations	1 (1.9)	8 (3.4)	13 (1.2)	-	-	2 (2.3)	-	24 (1.7)
Injury, poisoning, and procedural complications	9 (17.3)	17 (7.3)	42 (4.0)	1 (5.6)	-	3 (3.5)	-	72 (5.0)
Investigations	8 (15.4)	30 (12.9)	121 (11.6)	2 (11.1)	1 (11.1)	17 (19.8)	-	179 (12.4)
Metabolism and nutrition disorders	1 (1.9)	11 (4.7)	231 (22.1)	1 (5.6)	-	8 (9.3)	-	252 (17.4)
Musculoskeletal and connective tissue disorders	-	5 (2.1)	57 (5.4)	-	-	6 (7.0)	-	68 (4.7)
Neoplasms, benign, malignant, and unspecified (incl cysts and polyps)	-	-	-	-	-	1 (1.2)	-	1 (0.1)
Nervous system disorders	7 (13.5)	38 (16.3)	50 (4.8)	2 (11.1)	1 (11.1)	5 (5.8)	-	103 (7.1)
Psychiatric disorders	2 (3.8)	23 (9.9)	14 (1.3)	-	-	-	-	39 (2.7)
Renal and urinary disorders	8 (15.4)	12 (5.2)	125 (12.0)	1 (5.6)	-	10 (11.6)	1 (50.0)	157 (10.9)
Reproductive system and breast disorders	-	-	1 (0.1)	-	-	-	-	1 (0.1)
Respiratory, thoracic, and mediastinal disorders	1 (1.9)	10 (4.3)	30 (2.9)	3 (16.7)	-	5 (5.8)	-	49 (3.4)
Skin and subcutaneous tissue disorders	-	7 (3.0)	7 (0.7)	-	-	3 (3.5)	-	17 (1.2)
Social circumstances	-	1 (0.4)	2 (0.2)	-	-	-	-	3 (0.2)
Surgical and medical procedures	-	-	9 (0.9)	-	-	-	-	9 (0.6)
Vascular disorders	-	5 (2.1)	51 (4.9)	1 (5.6)	1 (11.1)	2 (2.3)	-	60 (4.1)

DEX: dexmedetomidine; MID: midazolam; PRO: propofol; DEX/MID: combinations of dexmedetomidine and midazolam; DEX/PRO: combinations of dexmedetomidine and propofol; MID/PRO: combinations of midazolam and propofol; DEX/MID/PRO: combinations of dexmedetomidine, midazolam, and propofol.

## Data Availability

EV data are publicly available at https://www.adrreports.eu/ (accessed on 27 March 2024).

## Supplementary Materials

The following supporting information can be downloaded at: https://www.mdpi.com/article/10.3390/jpm14090961/s1, Supplementary Table S1. List of preferred terms (PTs) reported in Individual Case Safety Reports (ICSRs) with dexmedetomidine, midazolam, propofol or combinations of these sedatives as suspect drug retrieved from the EudraVigilance spontaneous reporting system from 1 January 2013 to 31 December 2023 that were considered as indicative of rhabdomyolysis and were used for secondary analysis. Supplementary Figure S1. Distribution of concomitant drugs classified by the second level of the Anatomical Therapeutic Chemical (ATC) classification system reported in the Individual Case Safety Reports (ICSRs) related rhabdomyolysis and dexmedetomidine, midazolam, propofol or combinations of these drugs retrieved from the EudraVigilance spontaneous reporting system from 1 January 2013 to 31 December 2023. Supplementary Table S2. List of all adverse events reported in Individual Case Safety Reports (ICSRs) with rhabdomyolysis as adverse event and dexmedetomidine, midazolam, propofol or combinations of these sedatives as suspect drugs retrieved from the EudraVigilance spontaneous reporting system from 1 January 2013 to 31 December 2023. Supplementary Table S3. List of all preferred terms (PTs) reported in Individual Case Safety Reports (ICSRs) with dexmedetomidine, midazolam, propofol or combinations of these sedatives as suspect drug retrieved from the EudraVigilance spontaneous reporting system from 1st January 2013 to 31th December 2023. Supplementary Figure S2. Reporting odds ratio (ROR) of (a) rhabdomyolysis and (b) all PT indicative of rhabdomyolysis for the subgroup analysis. CI, confidence interval. Supplementary Figure S3. Reporting odds ratio (ROR) of (a) rhabdomyolysis and (b) all PT indicative of rhabdomyolysis between gender (male vs. female). CI, confidence interval.
