# Supplementary material for: Risk of Rhabdomyolysis Associated with Dexmedetomidine Use over the Past 10 Years: Insights from the EudraVigilance Database
Source: J Pers Med. 2024 Sep 10;14(9):961. doi: 10.3390/jpm14090961 (PMC11432986; doi:10.3390/jpm14090961)

Supplementary Table S1. List of preferred terms (PTs) reported in Individual Case Safety Reports (ICSRs) with dexmedetomidine, midazolam, propofol or combinations of these sedatives as suspect drug retrieved from the EudraVigilance spontaneous reporting system from 1st January 2013 to 31th December 2023 that were considered as indicative of rhabdomyolysis and were used for secondary analysis.

	**DEX** (N=34)	**MID** (N=155)	**PRO** (N=640)	**DEX/MID** (N=5)	**DEX/PRO** (N=12)	**MID/PRO** (N=79)	**DEX/MID/PRO** (N=3)	**Overall** (N=928)
**PT**								
Rhabdomyolysis	14 (41.2)	37 (23.9)	280 (43.8)	2 (40.0)	9 (75.0)	21 (26.6)	1 (33.3)	364 (39.3)
Blood creatine phosphokinase increased	9 (26.5)	20 (12.9)	96 (15.0)	-	2 (16.7)	13 (16.5)	-	140 (15.1)
Myalgia	-	18 (11.6)	62 (9.7)	-	-	2 (2.5)	-	82 (8.8)
Muscle spasms	2 (5.9)	14 (9.0)	39 (6.1)	1 (20.0)	-	8 (10.1)	-	64 (6.9)
Muscular weakness	1 (2.9)	17 (11.0)	40 (6.3)	2 (40.0)	-	2 (2.5)	-	62 (6.7)
Muscle twitching	-	13 (8.4)	32 (5.0)	-	-	6 (7.6)	-	51 (5.5)
Muscle rigidity	1 (2.9)	9 (5.8)	23 (3.6)	-	-	14 (17.7)	1 (33.3)	48 (5.2)
Blood creatinine increased	3 (8.8)	14 (9.0)	21 (3.3)	-	-	5 (6.3)	-	43 (4.6)
Myoglobin blood increased	2 (5.9)	-	12 (1.9)	-	1 (8.3)	1 (1.3)	-	16 (1.7)
Muscle tightness	-	5 (3.2)	5 (0.8)	-	-	-	1 (33.3)	11 (1.2)
Myoglobinaemia	-	1 (0.6)	5 (0.8)	-	-	-	-	6 (0.6)
Muscle contracture	-	-	4 (0.6)	-	-	2 (2.5)	-	6 (0.6)
Blood creatine increased	-	1 (0.6)	4 (0.6)	-	-	1 (1.3)	-	6 (0.6)
Myoglobinuria	-	1 (0.6)	5 (0.8)	-	-	-	-	6 (0.6)
Muscle disorder	1 (2.9)	1 (0.6)	1 (0.2)	-	-	1 (1.3)	-	4 (0.4)
Muscle necrosis	-	-	4 (0.6)	-	-	-	-	4 (0.4)
Myoglobin urine present	-	-	2 (0.3)	-	-	2 (2.5)	-	4 (0.4)
Muscle atrophy	-	2 (1.3)	1 (0.2)	-	-	-	-	3 (0.3)
Blood creatine phosphokinase abnormal	1 (2.9)	1 (0.6)	1 (0.2)	-	-	-	-	3 (0.3)
Musculoskeletal pain	-	-	1 (0.2)	-	-	1 (1.3)	-	2 (0.2)
Musculoskeletal discomfort	-	1 (0.6)	-	-	-	-	-	1 (0.1)
Muscle fatigue	-	-	1 (0.2)	-	-	-	-	1 (0.1)
Muscle swelling	-	-	1 (0.2)	-	-	-	-	1 (0.1)

DEX: dexmedetomidine; MID: midazolam; PRO: propofol; DEX/MID: combinations of dexmedetomidine and midazolam; DEX/PRO: combinations of dexmedetomidine and propofol; MID/PRO: combinations of midazolam and propofol; DEX/MID/PRO: combinations of dexmedetomidine, midazolam and propofol.

Supplementary Figure S1. Distribution of concomitant drugs classified by the second level of the Anatomical Therapeutic Chemical (ATC) classification system reported in the Individual Case Safety Reports (ICSRs) related rhabdomyolysis and dexmedetomidine, midazolam, propofol or combinations of these drugs retrieved from the EudraVigilance spontaneous reporting system from 1st January 2013 to 31th December 2023.

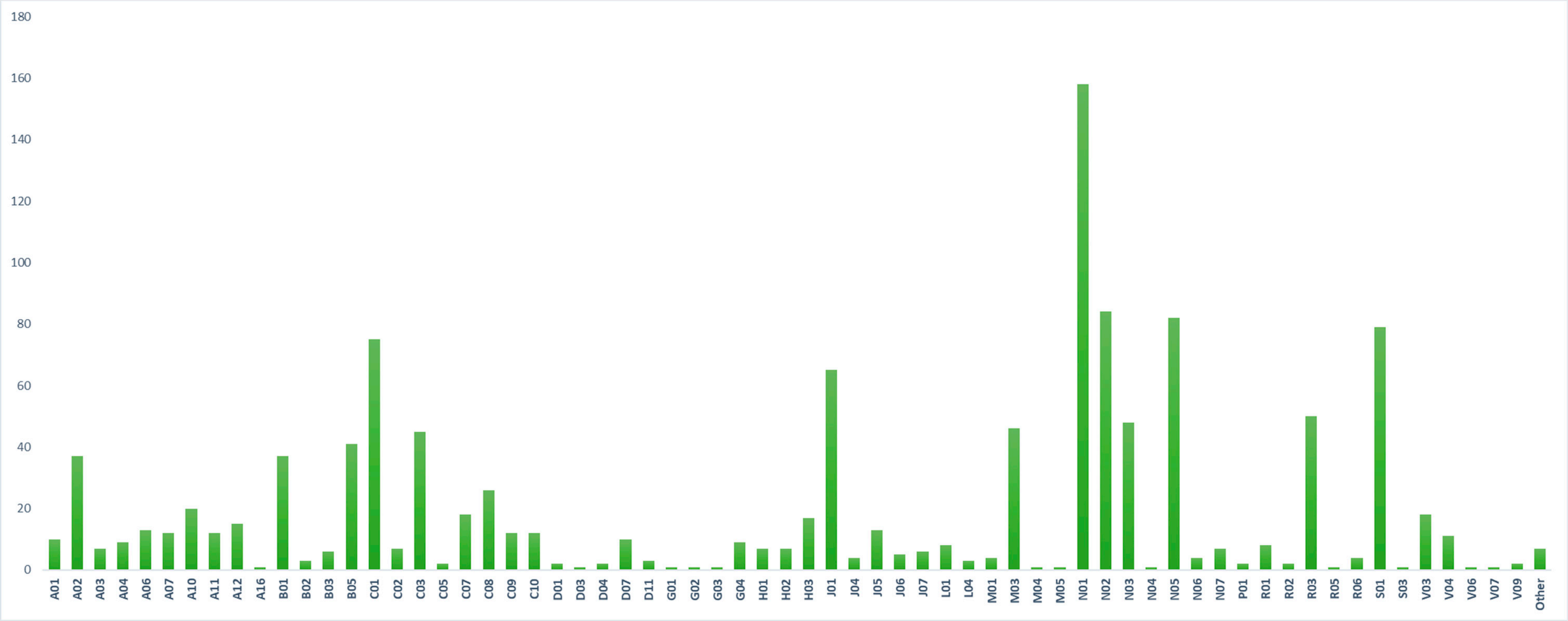

Legend: A01: stomatological preparations (N=10; 0.8%); A02: drugs for acid related disorders (N=37; 3.1%); A03: drugs for functional gastrointestinal disorders (N=7; 0.6%); A04: antiemetics and antinauseants (N=9; 0.7%); A06: drugs for constipation (N=13; 1.1%); A07: antidiarrheals (N=12; 1.0%); A10: drugs used in diabetes (N=20; 1.7%); A11: vitamins (N=12; 1.0%); A12: mineral supplements (N=15; 1.2%); A16: other alimentary tract and metabolism products (N=1; 0.1%); B01: antithrombotic agents (N=37; 3.1%); B02: antihemorrhagics (N=3; 0.2%); B03: antianemic preparations (N=6; 0.5%); B05: blood substitutes and perfusion solutions (N=41; 3.4%); C01: cardiac therapy (N=75; 6.2%); C02: antihypertensives (N=7; 0.6%); C03: diuretics (N=45; 3.7%); C05: vasoprotectives (N=2; 0.2%); C07: beta-blocking agents (N=18; 1.5%); C08: calcium channel blockers (N=26; 2.2%); C09:agents acting on the renin–angiotensin system (N=12; 1.0%); C10: lipid-modifying agents (N=12; 1.0%); D01: antifungals for dermatological use (N=2; 0.2%); D03: preparations for treatment of wounds and ulcers (N=1; 0.1%); D04: antipruritics, incl. antihistamines, anesthetics, etc. (N=2; 0.2%); D07: corticosteroids, dermatological preparations (N=10; 0.8%); D11: other dermatological preparations (N=3; 0.2%); G01: gynecological antiinfectives and antiseptics (N=1; 0.1%); G02: other gynecologicals (N=1; 0.1%); G03: sex hormones and modulators of the genital system (N=1; 0.1%); G04: urologicals (N=9; 0.7%); H01: pituitary and hypothalamic hormones and analogues (N=7; 0.6%); H02: corticosteroids for systemic use (N=7; 0.6%); H03: thyroid therapy (N=17; 1.4%); J01: antibacterials for systemic use (N=65; 5.4%); J04: antimycobacterials (N=4; 0.3%); J05: antivirals for systemic use (N=13; 1.1%); J06: immune sera and immunoglobulins (N=5; 0.4%); J07: vaccines (N=6; 0.5%); L01: antineoplastic agents (N=8; 0.7%);

L04: immunosuppressants (N=3; 0.2%); M01: antiinflammatory and antirheumatic products, non-steroids (N=4; 0.3%); M03: muscle relaxants (N=46;3.8%); M04: antigout preparations (N=1;0.1%); M05: drugs for treatment of bone diseases (N=1; 0.1%); N01: anesthetics (N=158; 13.1%); N02: analgesics (N=84; 7.0%); N03: antiepileptics (N=48; 4.0%); N04: anti-parkinson drugs (N=1; 0.1%); N05: psycholeptics (N=82; 6.8%); N06: psychoanaleptics (N=4; 0.3%); N07: other nervous system drugs (N=7; 0.6%); Other (N=7; 0.6%); P01: antiprotozoals (N=2; 0.2%); R01: nasal preparations (N=8; 0.7%); R02: throat preparations (N=2; 0.2%); R03: drugs for obstructive airway diseases (N=50; 4.1%); R05: cough and cold preparations (N=1; 0.1%); R06: drugs for obstructive airway diseases (N=4; 0.3%); S01: ophthalmologicals (N=79; 6.5%); S03: ophthalmological and otological preparations (N=1; 0.1%); V03: all other therapeutic products (N=18; 1.5%); V04: diagnostic agents (N=11; 0.9%); V06: general nutrients (1; 0.1%):V07: all other non-therapeutic products (N=1; 0.1%); V09: diagnostic radiopharmaceuticals (2; 0.2%).

Supplementary Table S2. List of all adverse events reported in Individual Case Safety Reports (ICSRs) with rhabdomyolysis as adverse event and dexmedetomidine, midazolam, propofol or combinations of these sedatives as suspect drugs retrieved from the EudraVigilance spontaneous reporting system from 1st January 2013 to 31th December 2023.

	DEX (N=66)	MID (N=270)	PRO (N=1326)	DEX/MID (N=20)	DEX/PRO (N=18)	MID/PRO (N=107)	DEX/MID/PRO (N=3)	Overall (N=1810)
**PT**								
Acute kidney injury	5 (7.6)	5 (1.9)	63 (4.8)	-	-	6 (5.6)	-	79 (4.4)
Blood chloride increased	1 (1.5)	-	-	-	-	-	-	1 (0.1)
Blood creatine phosphokinase increased	3 (4.5)	7 (2.6)	32 (2.4)	-	-	4 (3.7)	-	46 (2.5)
Blood creatinine increased	3 (4.5)	2 (0.7)	10 (0.8)	-	-	1 (0.9)	-	16 (0.9)
Cardiac failure acute	1 (1.5)	-	1 (0.1)	-	-	-	-	2 (0.1)
Catatonia	2 (3.0)	-	-	-	-	-	-	2 (0.1)
Constipation	2 (3.0)	-	-	-	-	-	-	2 (0.1)
Cranial nerve disorder	1 (1.5)	-	-	-	-	-	-	1 (0.1)
Drug ineffective	1 (1.5)	2 (0.7)	2 (0.2)	-	-	1 (0.9)	-	6 (0.3)
Drug ineffective for unapproved indication	1 (1.5)	1 (0.4)	-	-	-	-	-	2 (0.1)
General physical health deterioration	1 (1.5)	-	-	-	-	-	-	1 (0.1)
Hypernatraemia	1 (1.5)	1 (0.4)	-	-	-	-	-	2 (0.1)
Hyperpyrexia	2 (3.0)	-	-	-	-	-	-	2 (0.1)
Hyperthermia	3 (4.5)	4 (1.5)	7 (0.5)	-	1 (5.6)	-	-	15 (0.8)
Hyperthermia malignant	1 (1.5)	1 (0.4)	10 (0.8)	-	2 (11.1)	5 (4.7)	-	19 (1.0)
Intensive care unit acquired weakness	1 (1.5)	-	1 (0.1)	-	-	-	-	2 (0.1)
Intentional product use issue	2 (3.0)	-	1 (0.1)	-	-	-	-	3 (0.2)
Interstitial lung disease	1 (1.5)	-	-	-	-	-	-	1 (0.1)
Leukocytosis	2 (3.0)	3 (1.1)	1 (0.1)	-	-	-	-	6 (0.3)
Maternal exposure during pregnancy	2 (3.0)	-	-	-	-	-	-	2 (0.1)
Myoglobin blood increased	1 (1.5)	-	2 (0.2)	-	1 (5.6)	1 (0.9)	-	5 (0.3)
Neuroleptic malignant syndrome	2 (3.0)	2 (0.7)	7 (0.5)	1 (5.0)	1 (5.6)	1 (0.9)	-	14 (0.8)
Off label use	1 (1.5)	-	11 (0.8)	-	-	2 (1.9)	-	14 (0.8)

	DEX (N=66)	MID (N=270)	PRO (N=1326)	DEX/MID (N=20)	DEX/PRO (N=18)	MID/PRO (N=107)	DEX/MID/PRO (N=3)	Overall (N=1810)
Overdose	1 (1.5)	6 (2.2)	7 (0.5)	-	-	1 (0.9)	-	15 (0.8)
Product administered to patient of inappropriate age	1 (1.5)	-	1 (0.1)	1 (5.0)	-	-	-	3 (0.2)
Product use in unapproved indication	1 (1.5)	-	7 (0.5)	-	-	-	-	8 (0.4)
Product use issue	1 (1.5)	1 (0.4)	3 (0.2)	-	-	-	-	5 (0.3)
Pyrexia	1 (1.5)	8 (3.0)	23 (1.7)	-	1 (5.6)	3 (2.8)	1 (33.3)	37 (2.0)
Quadriparesis	1 (1.5)	-	-	-	-	-	-	1 (0.1)
Quadriplegia	1 (1.5)	-	-	-	-	-	-	1 (0.1)
Radiculopathy	1 (1.5)	-	-	-	-	-	-	1 (0.1)
Renal impairment	1 (1.5)	1 (0.4)	11 (0.8)	-	-	-	-	13 (0.7)
Rhabdomyolysis	14 (21.2)	37 (13.7)	280 (21.1)	2 (10.0)	9 (50.0)	21 (19.6)	1 (33.3)	364 (20.1)
Systemic candida	1 (1.5)	-	-	-	-	-	-	1 (0.1)
Urinary retention	2 (3.0)	-	-	-	-	-	-	2 (0.1)
Arrhythmia	-	1 (0.4)	15 (1.1)	1 (5.0)	-	1 (0.9)	-	18 (1.0)
Bradyarrhythmia	-	-	2 (0.2)	1 (5.0)	-	-	-	3 (0.2)
Chronic kidney disease	-	-	-	1 (5.0)	-	-	-	1 (0.1)
Conduction disorder	-	-	-	1 (5.0)	-	-	-	1 (0.1)
Drug interaction	-	8 (3.0)	4 (0.3)	1 (5.0)	-	1 (0.9)	-	14 (0.8)
Electrocardiogram QT prolonged	-	2 (0.7)	-	1 (5.0)	-	1 (0.9)	-	4 (0.2)
Electrocardiogram repolarisation abnormality	-	-	-	1 (5.0)	-	-	-	1 (0.1)
Hypocalcaemia	-	-	6 (0.5)	1 (5.0)	-	-	-	7 (0.4)
Long QT syndrome	-	-	1 (0.1)	1 (5.0)	-	-	-	2 (0.1)
Respiratory acidosis	-	-	1 (0.1)	1 (5.0)	-	-	-	2 (0.1)
Respiratory depression	-	2 (0.7)	1 (0.1)	1 (5.0)	-	1 (0.9)	-	5 (0.3)
Respiratory failure	-	3 (1.1)	2 (0.2)	1 (5.0)	-	-	-	6 (0.3)
Seizure	-	4 (1.5)	2 (0.2)	1 (5.0)	-	-	-	7 (0.4)
Shock	-	-	4 (0.3)	1 (5.0)	-	-	-	5 (0.3)
Sinus bradycardia	-	1 (0.4)	1 (0.1)	1 (5.0)	-	-	-	3 (0.2)

	DEX (N=66)	MID (N=270)	PRO (N=1326)	DEX/MID (N=20)	DEX/PRO (N=18)	MID/PRO (N=107)	DEX/MID/PRO (N=3)	Overall (N=1810)
Sinus node dysfunction	-	-	-	1 (5.0)	-	-	-	1 (0.1)
Renal failure	-	2 (0.7)	18 (1.4)	-	-	1 (0.9)	1 (33.3)	22 (1.2)
Cardio-respiratory arrest	-	-	-	-	1 (5.6)	-	-	1 (0.1)
Circulatory collapse	-	1 (0.4)	10 (0.8)	-	1 (5.6)	-	-	12 (0.7)
Multiple organ dysfunction syndrome	-	4 (1.5)	19 (1.4)	-	1 (5.6)	1 (0.9)	-	25 (1.4)
Acidosis	-	1 (0.4)	6 (0.5)	-	-	-	-	7 (0.4)
Acute respiratory distress syndrome	-	1 (0.4)	4 (0.3)	-	-	1 (0.9)	-	6 (0.3)
Adrenal cortex necrosis	-	1 (0.4)	-	-	-	-	-	1 (0.1)
Aggression	-	1 (0.4)	-	-	-	-	-	1 (0.1)
Agitation	-	1 (0.4)	10 (0.8)	-	-	-	-	11 (0.6)
Alanine aminotransferase increased	-	1 (0.4)	4 (0.3)	-	-	1 (0.9)	-	6 (0.3)
Altered state of consciousness	-	2 (0.7)	-	-	-	-	-	2 (0.1)
Anuria	-	1 (0.4)	3 (0.2)	-	-	-	-	4 (0.2)
Aspartate aminotransferase increased	-	1 (0.4)	5 (0.4)	-	-	1 (0.9)	-	7 (0.4)
Bacterial infection	-	1 (0.4)	-	-	-	-	-	1 (0.1)
Bedridden	-	1 (0.4)	-	-	-	-	-	1 (0.1)
Blood folate decreased	-	1 (0.4)	-	-	-	-	-	1 (0.1)
Blood glucose increased	-	1 (0.4)	-	-	-	-	-	1 (0.1)
Blood urea increased	-	1 (0.4)	-	-	-	1 (0.9)	-	2 (0.1)
Bradycardia	-	1 (0.4)	10 (0.8)	-	-	1 (0.9)	-	12 (0.7)
Bradypnoea	-	2 (0.7)	-	-	-	-	-	2 (0.1)
C-reactive protein increased	-	1 (0.4)	-	-	-	1 (0.9)	-	2 (0.1)
Cardiac arrest	-	2 (0.7)	16 (1.2)	-	-	-	-	18 (1.0)
Chills	-	1 (0.4)	8 (0.6)	-	-	-	-	9 (0.5)
Chromaturia	-	1 (0.4)	15 (1.1)	-	-	-	-	16 (0.9)
Colitis	-	1 (0.4)	-	-	-	-	-	1 (0.1)
Coma	-	2 (0.7)	4 (0.3)	-	-	-	-	6 (0.3)

	DEX (N=66)	MID (N=270)	PRO (N=1326)	DEX/MID (N=20)	DEX/PRO (N=18)	MID/PRO (N=107)	DEX/MID/PRO (N=3)	Overall (N=1810)
Completed suicide	-	1 (0.4)	-	-	-	-	-	1 (0.1)
Condition aggravated	-	1 (0.4)	8 (0.6)	-	-	-	-	9 (0.5)
Confusional state	-	2 (0.7)	-	-	-	-	-	2 (0.1)
Conjunctival hyperaemia	-	1 (0.4)	-	-	-	-	-	1 (0.1)
Coordination abnormal	-	1 (0.4)	-	-	-	-	-	1 (0.1)
Cyanosis	-	1 (0.4)	-	-	-	-	-	1 (0.1)
Decreased appetite	-	1 (0.4)	1 (0.1)	-	-	-	-	2 (0.1)
Decreased interest	-	1 (0.4)	-	-	-	-	-	1 (0.1)
Deep vein thrombosis	-	1 (0.4)	-	-	-	-	-	1 (0.1)
Depressed mood	-	1 (0.4)	-	-	-	-	-	1 (0.1)
Diabetic ketoacidosis	-	1 (0.4)	-	-	-	-	-	1 (0.1)
Disseminated intravascular coagulation	-	5 (1.9)	5 (0.4)	-	-	1 (0.9)	-	11 (0.6)
Dissociative disorder	-	1 (0.4)	-	-	-	-	-	1 (0.1)
Drug abuse	-	3 (1.1)	1 (0.1)	-	-	-	-	4 (0.2)
Drug level increased	-	2 (0.7)	-	-	-	-	-	2 (0.1)
Dyskinesia	-	1 (0.4)	-	-	-	-	-	1 (0.1)
Ecchymosis	-	1 (0.4)	-	-	-	-	-	1 (0.1)
Electrocardiogram abnormal	-	1 (0.4)	3 (0.2)	-	-	-	-	4 (0.2)
Electrocardiogram QRS complex prolonged	-	1 (0.4)	1 (0.1)	-	-	1 (0.9)	-	3 (0.2)
Epilepsy	-	1 (0.4)	1 (0.1)	-	-	-	-	2 (0.1)
Extensor plantar response	-	1 (0.4)	-	-	-	-	-	1 (0.1)
Fibrin D dimer increased	-	1 (0.4)	-	-	-	-	-	1 (0.1)
Generalised tonic-clonic seizure	-	2 (0.7)	-	-	-	-	-	2 (0.1)
Haemorrhagic transformation stroke	-	1 (0.4)	-	-	-	-	-	1 (0.1)
Hallucination, auditory	-	1 (0.4)	-	-	-	-	-	1 (0.1)
Heart rate decreased	-	1 (0.4)	-	-	-	-	-	1 (0.1)
Hepatic enzyme increased	-	2 (0.7)	5 (0.4)	-	-	-	-	7 (0.4)

	DEX (N=66)	MID (N=270)	PRO (N=1326)	DEX/MID (N=20)	DEX/PRO (N=18)	MID/PRO (N=107)	DEX/MID/PRO (N=3)	Overall (N=1810)
Hepatic failure	-	1 (0.4)	5 (0.4)	-	-	-	-	6 (0.3)
Hepatic function abnormal	-	2 (0.7)	5 (0.4)	-	-	-	-	7 (0.4)
Hyperglycaemia	-	1 (0.4)	1 (0.1)	-	-	-	-	2 (0.1)
Hyperhidrosis	-	3 (1.1)	-	-	-	-	-	3 (0.2)
Hyperkalaemia	-	2 (0.7)	23 (1.7)	-	-	1 (0.9)	-	26 (1.4)
Hyperreflexia	-	2 (0.7)	-	-	-	-	-	2 (0.1)
Hypertonia	-	3 (1.1)	-	-	-	1 (0.9)	-	4 (0.2)
Hypertransaminasaemia	-	1 (0.4)	5 (0.4)	-	-	-	-	6 (0.3)
Hyponatraemia	-	1 (0.4)	-	-	-	-	-	1 (0.1)
Hypotension	-	2 (0.7)	22 (1.7)	-	-	-	-	24 (1.3)
Hypotonia	-	1 (0.4)	-	-	-	-	-	1 (0.1)
Hypoxia	-	1 (0.4)	5 (0.4)	-	-	-	-	6 (0.3)
Inhibitory drug interaction	-	1 (0.4)	-	-	-	-	-	1 (0.1)
Insomnia	-	1 (0.4)	-	-	-	-	-	1 (0.1)
Intentional overdose	-	5 (1.9)	-	-	-	-	-	5 (0.3)
Intentional product misuse	-	1 (0.4)	-	-	-	-	-	1 (0.1)
Lagophthalmos	-	1 (0.4)	-	-	-	-	-	1 (0.1)
Loss of consciousness	-	1 (0.4)	1 (0.1)	-	-	-	-	2 (0.1)
Mental status changes	-	1 (0.4)	1 (0.1)	-	-	-	-	2 (0.1)
Metabolic acidosis	-	3 (1.1)	41 (3.1)	-	-	3 (2.8)	-	47 (2.6)
Mouth haemorrhage	-	1 (0.4)	-	-	-	-	-	1 (0.1)
Muscle atrophy	-	2 (0.7)	-	-	-	-	-	2 (0.1)
Muscle rigidity	-	1 (0.4)	1 (0.1)	-	-	1 (0.9)	-	3 (0.2)
Muscle twitching	-	1 (0.4)	-	-	-	-	-	1 (0.1)
Myalgia	-	1 (0.4)	12 (0.9)	-	-	1 (0.9)	-	14 (0.8)
Mydriasis	-	2 (0.7)	-	-	-	-	-	2 (0.1)
Myelosuppression	-	1 (0.4)	-	-	-	-	-	1 (0.1)

	DEX (N=66)	MID (N=270)	PRO (N=1326)	DEX/MID (N=20)	DEX/PRO (N=18)	MID/PRO (N=107)	DEX/MID/PRO (N=3)	Overall (N=1810)
Myoclonus	-	6 (2.2)	1 (0.1)	-	-	-	-	7 (0.4)
Myoglobinuria	-	1 (0.4)	3 (0.2)	-	-	-	-	4 (0.2)
Nausea	-	1 (0.4)	7 (0.5)	-	-	-	-	8 (0.4)
Negative thoughts	-	1 (0.4)	-	-	-	-	-	1 (0.1)
Oliguria	-	1 (0.4)	5 (0.4)	-	-	1 (0.9)	-	7 (0.4)
Osmotic demyelination syndrome	-	1 (0.4)	-	-	-	-	-	1 (0.1)
Pancreatitis necrotising	-	1 (0.4)	-	-	-	-	-	1 (0.1)
Persecutory delusion	-	1 (0.4)	-	-	-	-	-	1 (0.1)
Pigmentation disorder	-	1 (0.4)	-	-	-	-	-	1 (0.1)
Pneumonia	-	2 (0.7)	3 (0.2)	-	-	-	-	5 (0.3)
Pneumonia aspiration	-	1 (0.4)	-	-	-	2 (1.9)	-	3 (0.2)
Pneumonia pseudomonal	-	1 (0.4)	-	-	-	-	-	1 (0.1)
Pneumonitis aspiration	-	1 (0.4)	-	-	-	-	-	1 (0.1)
Pupillary reflex impaired	-	2 (0.7)	-	-	-	-	-	2 (0.1)
Rash	-	1 (0.4)	-	-	-	-	-	1 (0.1)
Respiratory rate decreased	-	1 (0.4)	1 (0.1)	-	-	-	-	2 (0.1)
Respiratory rate increased	-	1 (0.4)	-	-	-	-	-	1 (0.1)
Sepsis	-	1 (0.4)	3 (0.2)	-	-	-	-	4 (0.2)
Septic shock	-	1 (0.4)	2 (0.2)	-	-	-	-	3 (0.2)
Serotonin syndrome	-	4 (1.5)	4 (0.3)	-	-	1 (0.9)	-	9 (0.5)
Sinus tachycardia	-	2 (0.7)	-	-	-	-	-	2 (0.1)
Social avoidant behaviour	-	1 (0.4)	-	-	-	-	-	1 (0.1)
Staphylococcal infection	-	1 (0.4)	-	-	-	-	-	1 (0.1)
Staphylococcus test positive	-	1 (0.4)	1 (0.1)	-	-	-	-	2 (0.1)
Status epilepticus	-	2 (0.7)	1 (0.1)	-	-	-	-	3 (0.2)
Suicidal ideation	-	1 (0.4)	-	-	-	-	-	1 (0.1)
Suicide attempt	-	5 (1.9)	-	-	-	-	-	5 (0.3)

	DEX (N=66)	MID (N=270)	PRO (N=1326)	DEX/MID (N=20)	DEX/PRO (N=18)	MID/PRO (N=107)	DEX/MID/PRO (N=3)	Overall (N=1810)
Supraventricular tachycardia	-	2 (0.7)	2 (0.2)	-	-	-	-	4 (0.2)
Syncope	-	1 (0.4)	-	-	-	-	-	1 (0.1)
Tachycardia	-	1 (0.4)	4 (0.3)	-	-	1 (0.9)	-	6 (0.3)
Toxic epidermal necrolysis	-	1 (0.4)	-	-	-	-	-	1 (0.1)
Toxicity to various agents	-	4 (1.5)	3 (0.2)	-	-	-	-	7 (0.4)
Urine output decreased	-	1 (0.4)	-	-	-	-	-	1 (0.1)
Vomiting	-	1 (0.4)	3 (0.2)	-	-	-	-	4 (0.2)
Weight increased	-	1 (0.4)	1 (0.1)	-	-	-	-	2 (0.1)
Acute hepatic failure	-	-	3 (0.2)	-	-	1 (0.9)	-	4 (0.2)
Arthralgia	-	-	-	-	-	1 (0.9)	-	1 (0.1)
Arthropathy	-	-	-	-	-	1 (0.9)	-	1 (0.1)
Blister	-	-	-	-	-	1 (0.9)	-	1 (0.1)
Body temperature increased	-	-	1 (0.1)	-	-	1 (0.9)	-	2 (0.1)
Bundle branch block left	-	-	-	-	-	1 (0.9)	-	1 (0.1)
Carbon dioxide increased	-	-	-	-	-	1 (0.9)	-	1 (0.1)
Cardiac failure	-	-	6 (0.5)	-	-	1 (0.9)	-	7 (0.4)
Drug effect less than expected	-	-	-	-	-	1 (0.9)	-	1 (0.1)
End-tidal CO2 increased	-	-	-	-	-	1 (0.9)	-	1 (0.1)
Extrasystoles	-	-	-	-	-	1 (0.9)	-	1 (0.1)
Extravasation	-	-	-	-	-	1 (0.9)	-	1 (0.1)
Haematuria	-	-	3 (0.2)	-	-	1 (0.9)	-	4 (0.2)
Heart rate increased	-	-	-	-	-	1 (0.9)	-	1 (0.1)
Hepatotoxicity	-	-	1 (0.1)	-	-	1 (0.9)	-	2 (0.1)
Hyperammonaemia	-	-	-	-	-	1 (0.9)	-	1 (0.1)
Hypercapnia	-	-	1 (0.1)	-	-	1 (0.9)	-	2 (0.1)
Hypertension	-	-	1 (0.1)	-	-	1 (0.9)	-	2 (0.1)
Lactic acidosis	-	-	18 (1.4)	-	-	1 (0.9)	-	19 (1.0)

	DEX (N=66)	MID (N=270)	PRO (N=1326)	DEX/MID (N=20)	DEX/PRO (N=18)	MID/PRO (N=107)	DEX/MID/PRO (N=3)	Overall (N=1810)
Multiple-drug resistance	-	-	-	-	-	1 (0.9)	-	1 (0.1)
Muscle disorder	-	-	-	-	-	1 (0.9)	-	1 (0.1)
Neuromyopathy	-	-	-	-	-	1 (0.9)	-	1 (0.1)
Pain in extremity	-	-	5 (0.4)	-	-	1 (0.9)	-	6 (0.3)
Phlebitis	-	-	-	-	-	1 (0.9)	-	1 (0.1)
Propofol infusion syndrome	-	-	91 (6.9)	-	-	2 (1.9)	-	93 (5.1)
Proteinuria	-	-	-	-	-	1 (0.9)	-	1 (0.1)
Pulmonary haemorrhage	-	-	-	-	-	1 (0.9)	-	1 (0.1)
Pyogenic granuloma	-	-	-	-	-	1 (0.9)	-	1 (0.1)
Rash maculo-papular	-	-	1 (0.1)	-	-	2 (1.9)	-	3 (0.2)
Respiratory distress	-	-	1 (0.1)	-	-	1 (0.9)	-	2 (0.1)
Somnolence	-	-	-	-	-	1 (0.9)	-	1 (0.1)
Thrombocytopenia	-	-	2 (0.2)	-	-	1 (0.9)	-	3 (0.2)
Transaminases increased	-	-	5 (0.4)	-	-	1 (0.9)	-	6 (0.3)
Abdominal compartment syndrome	-	-	1 (0.1)	-	-	-	-	1 (0.1)
Alopecia	-	-	1 (0.1)	-	-	-	-	1 (0.1)
Anaemia	-	-	1 (0.1)	-	-	-	-	1 (0.1)
Anaesthetic complication	-	-	2 (0.2)	-	-	-	-	2 (0.1)
Ascites	-	-	2 (0.2)	-	-	-	-	2 (0.1)
Asthenia	-	-	7 (0.5)	-	-	-	-	7 (0.4)
Atelectasis	-	-	2 (0.2)	-	-	-	-	2 (0.1)
Atrial fibrillation	-	-	2 (0.2)	-	-	-	-	2 (0.1)
Atrioventricular block	-	-	3 (0.2)	-	-	-	-	3 (0.2)
Atrioventricular dissociation	-	-	1 (0.1)	-	-	-	-	1 (0.1)
Back pain	-	-	11 (0.8)	-	-	-	-	11 (0.6)
Biliary cast syndrome	-	-	1 (0.1)	-	-	-	-	1 (0.1)
Blood albumin decreased	-	-	1 (0.1)	-	-	-	-	1 (0.1)

	DEX (N=66)	MID (N=270)	PRO (N=1326)	DEX/MID (N=20)	DEX/PRO (N=18)	MID/PRO (N=107)	DEX/MID/PRO (N=3)	Overall (N=1810)
Blood creatine increased	-	-	1 (0.1)	-	-	-	-	1 (0.1)
Blood creatine phosphokinase abnormal	-	-	1 (0.1)	-	-	-	-	1 (0.1)
Blood lactate dehydrogenase increased	-	-	2 (0.2)	-	-	-	-	2 (0.1)
Blood lactic acid increased	-	-	1 (0.1)	-	-	-	-	1 (0.1)
Blood pressure decreased	-	-	4 (0.3)	-	-	-	-	4 (0.2)
Blood pressure fluctuation	-	-	1 (0.1)	-	-	-	-	1 (0.1)
Blood triglycerides increased	-	-	6 (0.5)	-	-	-	-	6 (0.3)
Brain oedema	-	-	2 (0.2)	-	-	-	-	2 (0.1)
Brain operation	-	-	1 (0.1)	-	-	-	-	1 (0.1)
Bronchospasm	-	-	1 (0.1)	-	-	-	-	1 (0.1)
Brugada syndrome	-	-	1 (0.1)	-	-	-	-	1 (0.1)
Bundle branch block right	-	-	2 (0.2)	-	-	-	-	2 (0.1)
Calcium ionised decreased	-	-	1 (0.1)	-	-	-	-	1 (0.1)
Cardiac contractility decreased	-	-	1 (0.1)	-	-	-	-	1 (0.1)
Cardiac disorder	-	-	1 (0.1)	-	-	-	-	1 (0.1)
Cardiac dysfunction	-	-	2 (0.2)	-	-	-	-	2 (0.1)
Cardiac massage	-	-	1 (0.1)	-	-	-	-	1 (0.1)
Cardiogenic shock	-	-	3 (0.2)	-	-	-	-	3 (0.2)
Cardiovascular disorder	-	-	2 (0.2)	-	-	-	-	2 (0.1)
Cardioversion	-	-	1 (0.1)	-	-	-	-	1 (0.1)
Carotid artery occlusion	-	-	1 (0.1)	-	-	-	-	1 (0.1)
Cerebral disorder	-	-	1 (0.1)	-	-	-	-	1 (0.1)
Cerebral haemorrhage	-	-	1 (0.1)	-	-	-	-	1 (0.1)
Cerebral infarction	-	-	2 (0.2)	-	-	-	-	2 (0.1)
Cheyne-Stokes respiration	-	-	1 (0.1)	-	-	-	-	1 (0.1)
Cholestasis	-	-	1 (0.1)	-	-	-	-	1 (0.1)
Cholestatic liver injury	-	-	1 (0.1)	-	-	-	-	1 (0.1)

	DEX (N=66)	MID (N=270)	PRO (N=1326)	DEX/MID (N=20)	DEX/PRO (N=18)	MID/PRO (N=107)	DEX/MID/PRO (N=3)	Overall (N=1810)
Choreoathetosis	-	-	1 (0.1)	-	-	-	-	1 (0.1)
Clonic convulsion	-	-	1 (0.1)	-	-	-	-	1 (0.1)
Coagulopathy	-	-	2 (0.2)	-	-	-	-	2 (0.1)
Compartment syndrome	-	-	2 (0.2)	-	-	-	-	2 (0.1)
Cough	-	-	1 (0.1)	-	-	-	-	1 (0.1)
Cytomegalovirus infection	-	-	1 (0.1)	-	-	-	-	1 (0.1)
Deafness unilateral	-	-	1 (0.1)	-	-	-	-	1 (0.1)
Death	-	-	1 (0.1)	-	-	-	-	1 (0.1)
Dehydration	-	-	1 (0.1)	-	-	-	-	1 (0.1)
Depressed level of consciousness	-	-	2 (0.2)	-	-	-	-	2 (0.1)
Diabetes insipidus	-	-	1 (0.1)	-	-	-	-	1 (0.1)
Diplegia	-	-	1 (0.1)	-	-	-	-	1 (0.1)
Distributive shock	-	-	1 (0.1)	-	-	-	-	1 (0.1)
Drug intolerance	-	-	1 (0.1)	-	-	-	-	1 (0.1)
Drug reaction with eosinophilia and systemic symptoms	-	-	1 (0.1)	-	-	-	-	1 (0.1)
Dyslipidaemia	-	-	1 (0.1)	-	-	-	-	1 (0.1)
Dyspnoea	-	-	1 (0.1)	-	-	-	-	1 (0.1)
Ejection fraction decreased	-	-	1 (0.1)	-	-	-	-	1 (0.1)
Electrocardiogram ST segment elevation	-	-	2 (0.2)	-	-	-	-	2 (0.1)
Electroencephalogram abnormal	-	-	1 (0.1)	-	-	-	-	1 (0.1)
Encephalitis	-	-	1 (0.1)	-	-	-	-	1 (0.1)
Encephalopathy	-	-	2 (0.2)	-	-	-	-	2 (0.1)
Familial risk factor	-	-	1 (0.1)	-	-	-	-	1 (0.1)
Fatigue	-	-	1 (0.1)	-	-	-	-	1 (0.1)
Flushing	-	-	1 (0.1)	-	-	-	-	1 (0.1)
Gait disturbance	-	-	4 (0.3)	-	-	-	-	4 (0.2)
Gait inability	-	-	4 (0.3)	-	-	-	-	4 (0.2)

	DEX (N=66)	MID (N=270)	PRO (N=1326)	DEX/MID (N=20)	DEX/PRO (N=18)	MID/PRO (N=107)	DEX/MID/PRO (N=3)	Overall (N=1810)
Gallbladder rupture	-	-	1 (0.1)	-	-	-	-	1 (0.1)
Gastrointestinal haemorrhage	-	-	1 (0.1)	-	-	-	-	1 (0.1)
Haemodynamic instability	-	-	5 (0.4)	-	-	-	-	5 (0.3)
Haemofiltration	-	-	2 (0.2)	-	-	-	-	2 (0.1)
Haemolysis	-	-	2 (0.2)	-	-	-	-	2 (0.1)
Haemorrhage	-	-	1 (0.1)	-	-	-	-	1 (0.1)
Hallucination	-	-	1 (0.1)	-	-	-	-	1 (0.1)
Hepatic cytolysis	-	-	4 (0.3)	-	-	-	-	4 (0.2)
Hepatic necrosis	-	-	1 (0.1)	-	-	-	-	1 (0.1)
Hepatic steatosis	-	-	1 (0.1)	-	-	-	-	1 (0.1)
Hepatomegaly	-	-	1 (0.1)	-	-	-	-	1 (0.1)
High density lipoprotein decreased	-	-	1 (0.1)	-	-	-	-	1 (0.1)
Hip arthroplasty	-	-	1 (0.1)	-	-	-	-	1 (0.1)
Hot flush	-	-	1 (0.1)	-	-	-	-	1 (0.1)
Hyperbilirubinaemia	-	-	2 (0.2)	-	-	-	-	2 (0.1)
Hyperchloraemia	-	-	1 (0.1)	-	-	-	-	1 (0.1)
Hypercreatininaemia	-	-	1 (0.1)	-	-	-	-	1 (0.1)
Hyperkeratosis	-	-	1 (0.1)	-	-	-	-	1 (0.1)
Hyperlactacidaemia	-	-	1 (0.1)	-	-	-	-	1 (0.1)
Hyperlipidaemia	-	-	5 (0.4)	-	-	-	-	5 (0.3)
Hypermagnesaemia	-	-	1 (0.1)	-	-	-	-	1 (0.1)
Hyperphosphataemia	-	-	2 (0.2)	-	-	-	-	2 (0.1)
Hypertensive crisis	-	-	1 (0.1)	-	-	-	-	1 (0.1)
Hypertriglyceridaemia	-	-	13 (1.0)	-	-	-	-	13 (0.7)
Hypoaesthesia	-	-	1 (0.1)	-	-	-	-	1 (0.1)
Hypokalaemia	-	-	6 (0.5)	-	-	-	-	6 (0.3)
Hypomagnesaemia	-	-	1 (0.1)	-	-	-	-	1 (0.1)

	DEX (N=66)	MID (N=270)	PRO (N=1326)	DEX/MID (N=20)	DEX/PRO (N=18)	MID/PRO (N=107)	DEX/MID/PRO (N=3)	Overall (N=1810)
Hypoparathyroidism	-	-	1 (0.1)	-	-	-	-	1 (0.1)
Hypophosphataemia	-	-	8 (0.6)	-	-	-	-	8 (0.4)
Hypothermia	-	-	2 (0.2)	-	-	-	-	2 (0.1)
Hypoxic-ischaemic encephalopathy	-	-	1 (0.1)	-	-	-	-	1 (0.1)
Immobile	-	-	1 (0.1)	-	-	-	-	1 (0.1)
Inborn error of lipid metabolism	-	-	1 (0.1)	-	-	-	-	1 (0.1)
Incorrect dose administered	-	-	2 (0.2)	-	-	-	-	2 (0.1)
Infection	-	-	2 (0.2)	-	-	-	-	2 (0.1)
Inflammation	-	-	1 (0.1)	-	-	-	-	1 (0.1)
Inflammatory marker increased	-	-	3 (0.2)	-	-	-	-	3 (0.2)
Infusion related reaction	-	-	1 (0.1)	-	-	-	-	1 (0.1)
International normalised ratio increased	-	-	1 (0.1)	-	-	-	-	1 (0.1)
Intestinal dilatation	-	-	1 (0.1)	-	-	-	-	1 (0.1)
Intestinal infarction	-	-	1 (0.1)	-	-	-	-	1 (0.1)
Intracranial pressure increased	-	-	3 (0.2)	-	-	-	-	3 (0.2)
Jugular vein distension	-	-	1 (0.1)	-	-	-	-	1 (0.1)
Knee arthroplasty	-	-	1 (0.1)	-	-	-	-	1 (0.1)
Lactate pyruvate ratio increased	-	-	2 (0.2)	-	-	-	-	2 (0.1)
Leukopenia	-	-	1 (0.1)	-	-	-	-	1 (0.1)
Life support	-	-	1 (0.1)	-	-	-	-	1 (0.1)
Lipids abnormal	-	-	1 (0.1)	-	-	-	-	1 (0.1)
Lipids increased	-	-	1 (0.1)	-	-	-	-	1 (0.1)
Liver disorder	-	-	4 (0.3)	-	-	-	-	4 (0.2)
Liver function test abnormal	-	-	1 (0.1)	-	-	-	-	1 (0.1)
Liver injury	-	-	2 (0.2)	-	-	-	-	2 (0.1)
Medication error	-	-	1 (0.1)	-	-	-	-	1 (0.1)
Melaena	-	-	1 (0.1)	-	-	-	-	1 (0.1)

	DEX (N=66)	MID (N=270)	PRO (N=1326)	DEX/MID (N=20)	DEX/PRO (N=18)	MID/PRO (N=107)	DEX/MID/PRO (N=3)	Overall (N=1810)
Methaemoglobinaemia	-	-	3 (0.2)	-	-	-	-	3 (0.2)
Mitochondrial cytopathy	-	-	1 (0.1)	-	-	-	-	1 (0.1)
Mitochondrial toxicity	-	-	2 (0.2)	-	-	-	-	2 (0.1)
Muscle necrosis	-	-	1 (0.1)	-	-	-	-	1 (0.1)
Muscle spasms	-	-	2 (0.2)	-	-	-	-	2 (0.1)
Muscle swelling	-	-	1 (0.1)	-	-	-	-	1 (0.1)
Muscular weakness	-	-	10 (0.8)	-	-	-	-	10 (0.6)
Musculoskeletal disorder	-	-	1 (0.1)	-	-	-	-	1 (0.1)
Musculoskeletal pain	-	-	1 (0.1)	-	-	-	-	1 (0.1)
Myocardial infarction	-	-	1 (0.1)	-	-	-	-	1 (0.1)
Myocardial injury	-	-	1 (0.1)	-	-	-	-	1 (0.1)
Myoglobin urine present	-	-	1 (0.1)	-	-	-	-	1 (0.1)
Myoglobinaemia	-	-	2 (0.2)	-	-	-	-	2 (0.1)
Myopathy	-	-	4 (0.3)	-	-	-	-	4 (0.2)
Myopathy toxic	-	-	1 (0.1)	-	-	-	-	1 (0.1)
Neck pain	-	-	3 (0.2)	-	-	-	-	3 (0.2)
Necrosis	-	-	1 (0.1)	-	-	-	-	1 (0.1)
Nephrogenic anaemia	-	-	2 (0.2)	-	-	-	-	2 (0.1)
Neutropenia	-	-	1 (0.1)	-	-	-	-	1 (0.1)
Odynophagia	-	-	1 (0.1)	-	-	-	-	1 (0.1)
Oedema	-	-	2 (0.2)	-	-	-	-	2 (0.1)
Oedema peripheral	-	-	1 (0.1)	-	-	-	-	1 (0.1)
Pancreatitis acute	-	-	4 (0.3)	-	-	-	-	4 (0.2)
Pancytopenia	-	-	1 (0.1)	-	-	-	-	1 (0.1)
Partial seizures	-	-	1 (0.1)	-	-	-	-	1 (0.1)
Pericardial effusion	-	-	1 (0.1)	-	-	-	-	1 (0.1)
Peripheral coldness	-	-	1 (0.1)	-	-	-	-	1 (0.1)

	DEX (N=66)	MID (N=270)	PRO (N=1326)	DEX/MID (N=20)	DEX/PRO (N=18)	MID/PRO (N=107)	DEX/MID/PRO (N=3)	Overall (N=1810)
Peripheral swelling	-	-	1 (0.1)	-	-	-	-	1 (0.1)
Platelet count decreased	-	-	1 (0.1)	-	-	-	-	1 (0.1)
Pleural effusion	-	-	2 (0.2)	-	-	-	-	2 (0.1)
Polyneuropathy	-	-	1 (0.1)	-	-	-	-	1 (0.1)
Poor peripheral circulation	-	-	1 (0.1)	-	-	-	-	1 (0.1)
Post procedural complication	-	-	1 (0.1)	-	-	-	-	1 (0.1)
Post procedural fever	-	-	1 (0.1)	-	-	-	-	1 (0.1)
Priapism	-	-	1 (0.1)	-	-	-	-	1 (0.1)
Procalcitonin increased	-	-	1 (0.1)	-	-	-	-	1 (0.1)
Procedural haemorrhage	-	-	1 (0.1)	-	-	-	-	1 (0.1)
Pruritus	-	-	1 (0.1)	-	-	-	-	1 (0.1)
Psychomotor hyperactivity	-	-	2 (0.2)	-	-	-	-	2 (0.1)
Pulmonary oedema	-	-	3 (0.2)	-	-	-	-	3 (0.2)
Pulseless electrical activity	-	-	1 (0.1)	-	-	-	-	1 (0.1)
Rash macular	-	-	1 (0.1)	-	-	-	-	1 (0.1)
Rash vesicular	-	-	1 (0.1)	-	-	-	-	1 (0.1)
Renal disorder	-	-	1 (0.1)	-	-	-	-	1 (0.1)
Renal tubular necrosis	-	-	1 (0.1)	-	-	-	-	1 (0.1)
Respiratory arrest	-	-	1 (0.1)	-	-	-	-	1 (0.1)
Restlessness	-	-	1 (0.1)	-	-	-	-	1 (0.1)
Resuscitation	-	-	1 (0.1)	-	-	-	-	1 (0.1)
Rhythm idioventricular	-	-	1 (0.1)	-	-	-	-	1 (0.1)
Right ventricular dysfunction	-	-	1 (0.1)	-	-	-	-	1 (0.1)
Right ventricular failure	-	-	1 (0.1)	-	-	-	-	1 (0.1)
Spinal cord infarction	-	-	1 (0.1)	-	-	-	-	1 (0.1)
Subarachnoid haemorrhage	-	-	1 (0.1)	-	-	-	-	1 (0.1)
Swelling face	-	-	1 (0.1)	-	-	-	-	1 (0.1)

	DEX (N=66)	MID (N=270)	PRO (N=1326)	DEX/MID (N=20)	DEX/PRO (N=18)	MID/PRO (N=107)	DEX/MID/PRO (N=3)	Overall (N=1810)
Swelling of eyelid	-	-	1 (0.1)	-	-	-	-	1 (0.1)
Tachypnoea	-	-	2 (0.2)	-	-	-	-	2 (0.1)
Tenderness	-	-	1 (0.1)	-	-	-	-	1 (0.1)
Thrombotic thrombocytopenic purpura	-	-	1 (0.1)	-	-	-	-	1 (0.1)
Toxic shock syndrome	-	-	1 (0.1)	-	-	-	-	1 (0.1)
Tremor	-	-	1 (0.1)	-	-	-	-	1 (0.1)
Troponin I increased	-	-	1 (0.1)	-	-	-	-	1 (0.1)
Troponin T increased	-	-	1 (0.1)	-	-	-	-	1 (0.1)
Unresponsive to stimuli	-	-	2 (0.2)	-	-	-	-	2 (0.1)
Urinary incontinence	-	-	1 (0.1)	-	-	-	-	1 (0.1)
Urine abnormality	-	-	1 (0.1)	-	-	-	-	1 (0.1)
Urine analysis abnormal	-	-	6 (0.5)	-	-	-	-	6 (0.3)
Ventricular extrasystoles	-	-	1 (0.1)	-	-	-	-	1 (0.1)
Ventricular fibrillation	-	-	4 (0.3)	-	-	-	-	4 (0.2)
Ventricular hypokinesia	-	-	1 (0.1)	-	-	-	-	1 (0.1)
Ventricular tachycardia	-	-	8 (0.6)	-	-	-	-	8 (0.4)
Weight decreased	-	-	6 (0.5)	-	-	-	-	6 (0.3)
Wheezing	-	-	1 (0.1)	-	-	-	-	1 (0.1)
White blood cell count decreased	-	-	1 (0.1)	-	-	-	-	1 (0.1)
White blood cell count increased	-	-	1 (0.1)	-	-	-	-	1 (0.1)

DEX: dexmedetomidine; MID: midazolam; PRO: propofol; DEX/MID: combinations of dexmedetomidine and midazolam; DEX/PRO: combinations of dexmedetomidine and propofol; MID/PRO: combinations of midazolam and propofol; DEX/MID/PRO: combinations of dexmedetomidine, midazolam and propofol.

Supplementary Table S3. List of all preferred terms (PTs) reported in Individual Case Safety Reports (ICSRs) with dexmedetomidine, midazolam, propofol or combinations of these sedatives as suspect drug retrieved from the EudraVigilance spontaneous reporting system from 1st January 2013 to 31th December 2023.

	DEX (N=3841)	MID (N=14967)	PRO (N=24959)	DEX/MID (N=335)	DEX/PRO (N=497)	MID/PRO (N=4690)	DEX/MID/PRO (N=265)	Overall (N=49554)
**PT**								
Abdominal compartment syndrome	-	2 (0.0)	5 (0.0)	-	-	1 (0.0)	-	8 (0.0)
Abdominal discomfort	-	9 (0.1)	8 (0.0)	-	-	-	-	17 (0.0)
Abdominal distension	1 (0.0)	19 (0.1)	11 (0.0)	1 (0.3)	-	4 (0.1)	-	36 (0.1)
Abdominal injury	-	-	1 (0.0)	-	-	-	-	1 (0.0)
Abdominal pain	3 (0.1)	56 (0.4)	40 (0.2)	-	1 (0.2)	10 (0.2)	-	110 (0.2)
Abdominal pain lower	-	2 (0.0)	-	-	-	1 (0.0)	-	3 (0.0)
Abdominal pain upper	1 (0.0)	17 (0.1)	8 (0.0)	-	-	3 (0.1)	-	29 (0.1)
Abdominal rigidity	-	-	-	-	-	2 (0.0)	-	2 (0.0)
Abdominal symptom	1 (0.0)	-	-	-	-	-	-	1 (0.0)
Abdominal tenderness	-	1 (0.0)	2 (0.0)	-	-	-	-	3 (0.0)
Abdominal wall anomaly	-	-	2 (0.0)	-	-	-	-	2 (0.0)
Abdominal wall haematoma	1 (0.0)	-	-	-	-	1 (0.0)	-	2 (0.0)
Abnormal behaviour	1 (0.0)	18 (0.1)	6 (0.0)	1 (0.3)	-	-	-	26 (0.1)
Abnormal dreams	-	8 (0.1)	-	-	-	1 (0.0)	-	9 (0.0)
Abnormal faeces	1 (0.0)	1 (0.0)	-	-	-	-	-	2 (0.0)
Abnormal loss of weight	-	-	1 (0.0)	-	-	-	-	1 (0.0)
Abnormal sleep-related event	-	1 (0.0)	-	-	-	-	-	1 (0.0)
Abortion	-	-	-	-	-	1 (0.0)	-	1 (0.0)
Abortion induced	-	1 (0.0)	12 (0.0)	-	-	3 (0.1)	-	16 (0.0)
Abortion spontaneous	-	3 (0.0)	4 (0.0)	-	-	3 (0.1)	-	10 (0.0)
Abscess	-	3 (0.0)	1 (0.0)	-	-	2 (0.0)	-	6 (0.0)
Abscess limb	-	1 (0.0)	1 (0.0)	-	-	-	-	2 (0.0)
Abscess rupture	-	1 (0.0)	-	-	-	-	-	1 (0.0)
Acanthosis nigricans	-	4 (0.0)	-	-	-	-	-	4 (0.0)
Accelerated hypertension	-	-	1 (0.0)	-	-	-	-	1 (0.0)

Accelerated idioventricular rhythm	-	-	5 (0.0)	-	-	-	-	5 (0.0)
Accident	-	3 (0.0)	1 (0.0)	-	-	-	-	4 (0.0)
Accidental death	-	5 (0.0)	4 (0.0)	-	-	-	-	9 (0.0)
Accidental exposure to product	-	5 (0.0)	8 (0.0)	-	-	-	-	13 (0.0)
Accidental exposure to product by child	1 (0.0)	2 (0.0)	-	-	-	-	-	3 (0.0)
Accidental exposure to product packaging	-	1 (0.0)	-	-	-	-	-	1 (0.0)
Accidental overdose	16 (0.4)	35 (0.2)	17 (0.1)	-	-	2 (0.0)	-	70 (0.1)
Accidental poisoning	-	1 (0.0)	1 (0.0)	-	-	-	-	2 (0.0)
Accidental underdose	-	-	1 (0.0)	-	-	-	-	1 (0.0)
Acid base balance abnormal	-	1 (0.0)	-	-	-	-	-	1 (0.0)
Acidosis	1 (0.0)	6 (0.0)	31 (0.1)	-	-	2 (0.0)	-	40 (0.1)
Acidosis hyperchloraemic	-	7 (0.0)	2 (0.0)	-	-	5 (0.1)	-	14 (0.0)
Acinetobacter infection	-	2 (0.0)	-	-	-	1 (0.0)	-	3 (0.0)
Acne conglobata	-	1 (0.0)	-	-	-	-	-	1 (0.0)
Acquired antithrombin III deficiency	-	-	1 (0.0)	-	-	-	-	1 (0.0)
Acquired diaphragmatic eventration	-	-	1 (0.0)	-	-	-	-	1 (0.0)
Acquired haemophilia	-	1 (0.0)	-	-	-	-	-	1 (0.0)
Acrochordon	-	2 (0.0)	-	-	-	-	-	2 (0.0)
Activated partial thromboplastin time prolonged	2 (0.1)	1 (0.0)	4 (0.0)	-	-	-	-	7 (0.0)
Acute abdomen	-	1 (0.0)	-	-	-	-	-	1 (0.0)
Acute chest syndrome	-	-	2 (0.0)	-	-	-	-	2 (0.0)
Acute coronary syndrome	1 (0.0)	4 (0.0)	8 (0.0)	-	-	-	1 (0.4)	14 (0.0)
Acute generalised exanthematous pustulosis	-	5 (0.0)	15 (0.1)	-	1 (0.2)	2 (0.0)	-	23 (0.0)
Acute graft versus host disease	-	-	-	-	-	1 (0.0)	-	1 (0.0)
Acute hepatic failure	1 (0.0)	5 (0.0)	23 (0.1)	-	-	5 (0.1)	-	34 (0.1)
Acute hepatitis B	-	-	2 (0.0)	-	-	-	-	2 (0.0)
Acute kidney injury	7 (0.2)	36 (0.2)	142 (0.6)	-	3 (0.6)	24 (0.5)	-	212 (0.4)
Acute left ventricular failure	2 (0.1)	-	1 (0.0)	-	-	-	-	3 (0.0)
Acute lung injury	-	1 (0.0)	2 (0.0)	-	-	2 (0.0)	-	5 (0.0)
Acute motor axonal neuropathy	1 (0.0)	-	-	-	-	-	-	1 (0.0)
Acute myeloid leukaemia	-	-	1 (0.0)	-	-	-	-	1 (0.0)
Acute myocardial infarction	1 (0.0)	3 (0.0)	13 (0.1)	-	-	1 (0.0)	1 (0.4)	19 (0.0)

Acute postoperative sialadenitis	-	2 (0.0)	8 (0.0)	-	-	1 (0.0)	-	11 (0.0)
Acute psychosis	2 (0.1)	-	1 (0.0)	-	-	-	-	3 (0.0)
Acute pulmonary oedema	-	2 (0.0)	25 (0.1)	-	-	7 (0.1)	-	34 (0.1)
Acute respiratory distress syndrome	7 (0.2)	12 (0.1)	25 (0.1)	1 (0.3)	1 (0.2)	6 (0.1)	-	52 (0.1)
Acute respiratory failure	3 (0.1)	16 (0.1)	30 (0.1)	-	1 (0.2)	-	-	50 (0.1)
Acute right ventricular failure	-	-	2 (0.0)	-	-	-	-	2 (0.0)
Acute sinusitis	-	-	1 (0.0)	-	-	-	-	1 (0.0)
Adaptive servo-ventilation	-	-	1 (0.0)	-	-	-	-	1 (0.0)
Adenocarcinoma gastric	-	1 (0.0)	-	-	-	-	-	1 (0.0)
Adenoidal hypertrophy	-	1 (0.0)	-	-	-	-	-	1 (0.0)
Adenoidectomy	-	-	1 (0.0)	-	-	-	-	1 (0.0)
Administration related reaction	-	-	-	-	-	1 (0.0)	-	1 (0.0)
Administration site erythema	-	-	1 (0.0)	-	-	-	-	1 (0.0)
Administration site extravasation	1 (0.0)	-	62 (0.2)	-	-	-	-	63 (0.1)
Administration site irritation	-	-	10 (0.0)	-	-	-	-	10 (0.0)
Administration site joint erythema	-	-	1 (0.0)	-	-	-	-	1 (0.0)
Administration site oedema	-	-	3 (0.0)	-	-	-	-	3 (0.0)
Administration site pain	-	2 (0.0)	11 (0.0)	-	-	-	-	13 (0.0)
Administration site reaction	-	-	1 (0.0)	-	-	-	-	1 (0.0)
Administration site swelling	-	-	5 (0.0)	-	-	-	-	5 (0.0)
Administration site warmth	-	-	1 (0.0)	-	-	-	-	1 (0.0)
Adrenal cortex necrosis	-	1 (0.0)	-	-	-	-	-	1 (0.0)
Adrenal disorder	-	-	1 (0.0)	-	-	-	-	1 (0.0)
Adrenal insufficiency	-	1 (0.0)	3 (0.0)	1 (0.3)	-	2 (0.0)	-	7 (0.0)
Adrenoleukodystrophy	-	-	1 (0.0)	-	-	-	-	1 (0.0)
Adverse drug reaction	2 (0.1)	9 (0.1)	8 (0.0)	-	-	4 (0.1)	-	23 (0.0)
Adverse event	-	4 (0.0)	3 (0.0)	-	-	-	-	7 (0.0)
Adverse reaction	-	-	1 (0.0)	-	-	-	-	1 (0.0)
Affect lability	-	-	1 (0.0)	-	-	-	-	1 (0.0)
Affective disorder	-	1 (0.0)	-	-	-	-	-	1 (0.0)
Ageusia	-	1 (0.0)	5 (0.0)	-	-	-	-	6 (0.0)
Aggression	5 (0.1)	55 (0.4)	21 (0.1)	1 (0.3)	-	3 (0.1)	2 (0.8)	87 (0.2)

Agitation	36 (0.9)	150 (1.0)	101 (0.4)	4 (1.2)	4 (0.8)	22 (0.5)	4 (1.5)	321 (0.6)
Agitation neonatal	1 (0.0)	-	-	-	-	-	-	1 (0.0)
Agitation postoperative	-	1 (0.0)	1 (0.0)	-	-	1 (0.0)	-	3 (0.0)
Agnosia	-	3 (0.0)	-	-	-	-	-	3 (0.0)
Agoraphobia	-	1 (0.0)	-	-	-	-	-	1 (0.0)
Agranulocytosis	-	5 (0.0)	9 (0.0)	-	-	1 (0.0)	-	15 (0.0)
Air embolism	-	2 (0.0)	3 (0.0)	-	-	1 (0.0)	-	6 (0.0)
Airway complication of anaesthesia	1 (0.0)	2 (0.0)	47 (0.2)	-	-	11 (0.2)	3 (1.1)	64 (0.1)
Airway peak pressure increased	-	1 (0.0)	12 (0.0)	-	-	5 (0.1)	-	18 (0.0)
Akathisia	1 (0.0)	6 (0.0)	5 (0.0)	-	-	2 (0.0)	-	14 (0.0)
Akinesia	-	1 (0.0)	1 (0.0)	-	-	1 (0.0)	-	3 (0.0)
Alanine aminotransferase increased	6 (0.2)	20 (0.1)	29 (0.1)	1 (0.3)	-	11 (0.2)	-	67 (0.1)
Alcohol abuse	1 (0.0)	-	-	-	-	-	-	1 (0.0)
Alcohol interaction	-	1 (0.0)	-	-	-	-	-	1 (0.0)
Alcohol poisoning	-	1 (0.0)	-	-	-	1 (0.0)	-	2 (0.0)
Alcohol withdrawal syndrome	1 (0.0)	-	-	-	-	-	-	1 (0.0)
Alcoholic hangover	-	-	1 (0.0)	-	-	-	-	1 (0.0)
Allergic cough	-	-	1 (0.0)	-	-	-	-	1 (0.0)
Allergic reaction to excipient	-	-	4 (0.0)	-	-	-	-	4 (0.0)
Allergy test negative	-	-	26 (0.1)	-	-	1 (0.0)	-	27 (0.1)
Allergy test positive	-	1 (0.0)	6 (0.0)	-	-	1 (0.0)	-	8 (0.0)
Allergy to vaccine	-	-	2 (0.0)	-	-	-	-	2 (0.0)
Alopecia	-	6 (0.0)	6 (0.0)	-	-	-	-	12 (0.0)
Alopecia universalis	-	-	1 (0.0)	-	-	-	-	1 (0.0)
Alpha haemolytic streptococcal infection	-	-	-	-	-	1 (0.0)	-	1 (0.0)
Altered state of consciousness	5 (0.1)	56 (0.4)	22 (0.1)	-	5 (1.0)	10 (0.2)	1 (0.4)	99 (0.2)
Alveolar lung disease	-	-	1 (0.0)	-	-	-	-	1 (0.0)
Alveolitis	-	-	1 (0.0)	-	-	-	-	1 (0.0)
Amaurosis	-	1 (0.0)	-	-	-	-	-	1 (0.0)
Amino acid level increased	-	3 (0.0)	-	-	-	-	-	3 (0.0)
Ammonia increased	-	2 (0.0)	4 (0.0)	-	-	2 (0.0)	-	8 (0.0)
Amnesia	1 (0.0)	40 (0.3)	15 (0.1)	-	-	3 (0.1)	-	59 (0.1)

Amnestic disorder	-	1 (0.0)	1 (0.0)	-	-	-	-	2 (0.0)
Amniotic cavity infection	1 (0.0)	-	1 (0.0)	-	-	-	-	2 (0.0)
Amniotic fluid index decreased	-	-	1 (0.0)	-	-	-	-	1 (0.0)
Amylase increased	-	-	6 (0.0)	-	-	2 (0.0)	-	8 (0.0)
Amyotrophy	-	-	1 (0.0)	-	-	-	-	1 (0.0)
Anaemia	2 (0.1)	36 (0.2)	18 (0.1)	-	1 (0.2)	14 (0.3)	-	71 (0.1)
Anaemia neonatal	-	1 (0.0)	2 (0.0)	-	-	-	-	3 (0.0)
Anaesthesia	-	-	2 (0.0)	-	-	1 (0.0)	-	3 (0.0)
Anaesthetic complication	1 (0.0)	10 (0.1)	81 (0.3)	1 (0.3)	1 (0.2)	6 (0.1)	-	100 (0.2)
Anaesthetic complication cardiac	-	-	1 (0.0)	-	-	1 (0.0)	-	2 (0.0)
Anaesthetic complication neurological	3 (0.1)	8 (0.1)	46 (0.2)	-	-	31 (0.7)	1 (0.4)	89 (0.2)
Anaesthetic complication pulmonary	-	-	2 (0.0)	-	-	1 (0.0)	-	3 (0.0)
Anaesthetic complication vascular	-	-	1 (0.0)	-	-	-	-	1 (0.0)
Anal fissure	-	1 (0.0)	-	-	-	-	-	1 (0.0)
Anal incontinence	-	7 (0.0)	5 (0.0)	-	-	-	-	12 (0.0)
Anal prolapse	-	1 (0.0)	-	-	-	-	-	1 (0.0)
Anal stenosis	-	1 (0.0)	-	-	-	-	-	1 (0.0)
Anaphylactic reaction	8 (0.2)	114 (0.8)	712 (2.9)	-	7 (1.4)	200 (4.3)	2 (0.8)	1043 (2.1)
Anaphylactic shock	8 (0.2)	113 (0.8)	1492 (6.0)	1 (0.3)	14 (2.8)	124 (2.6)	-	1752 (3.5)
Anaphylactoid reaction	-	9 (0.1)	62 (0.2)	-	1 (0.2)	14 (0.3)	-	86 (0.2)
Anaphylactoid shock	-	3 (0.0)	43 (0.2)	-	-	9 (0.2)	-	55 (0.1)
Anaphylactoid syndrome of pregnancy	-	-	1 (0.0)	-	-	-	-	1 (0.0)
Anaphylaxis treatment	-	-	-	-	-	1 (0.0)	-	1 (0.0)
Anembryonic gestation	-	1 (0.0)	-	-	-	-	-	1 (0.0)
Anencephaly	-	-	1 (0.0)	-	-	-	-	1 (0.0)
Anger	-	9 (0.1)	1 (0.0)	-	-	-	-	10 (0.0)
Angina pectoris	-	2 (0.0)	1 (0.0)	-	-	-	1 (0.4)	4 (0.0)
Angina unstable	-	-	1 (0.0)	-	-	-	-	1 (0.0)
Angioedema	4 (0.1)	33 (0.2)	70 (0.3)	-	1 (0.2)	17 (0.4)	-	125 (0.3)
Angiopathy	-	-	2 (0.0)	-	-	-	-	2 (0.0)
Angle closure glaucoma	-	2 (0.0)	6 (0.0)	-	1 (0.2)	-	1 (0.4)	10 (0.0)
Anion gap decreased	1 (0.0)	-	-	-	-	-	-	1 (0.0)

Anisocoria	-	1 (0.0)	9 (0.0)	-	-	3 (0.1)	-	13 (0.0)
Anorectal discomfort	-	2 (0.0)	-	-	-	-	-	2 (0.0)
Anorectal disorder	-	1 (0.0)	-	-	-	-	-	1 (0.0)
Anorectal ulcer	-	1 (0.0)	-	-	-	-	-	1 (0.0)
Anosmia	-	1 (0.0)	7 (0.0)	-	-	2 (0.0)	-	10 (0.0)
Anoxia	-	-	1 (0.0)	-	-	-	-	1 (0.0)
Anterior spinal artery syndrome	-	-	1 (0.0)	-	-	-	-	1 (0.0)
Anterograde amnesia	-	4 (0.0)	1 (0.0)	-	-	2 (0.0)	-	7 (0.0)
Anticholinergic syndrome	-	4 (0.0)	11 (0.0)	-	1 (0.2)	6 (0.1)	-	22 (0.0)
Anticonvulsant drug level abnormal	-	1 (0.0)	-	-	-	-	-	1 (0.0)
Anticonvulsant drug level above therapeutic	-	-	-	-	-	1 (0.0)	-	1 (0.0)
Anticonvulsant drug level decreased	-	3 (0.0)	-	-	-	-	-	3 (0.0)
Anticonvulsant drug level increased	-	-	4 (0.0)	-	-	1 (0.0)	-	5 (0.0)
Anticonvulsant drug level therapeutic	-	1 (0.0)	-	-	-	-	-	1 (0.0)
Anti-NMDA antibody positive	-	-	-	-	-	1 (0.0)	-	1 (0.0)
Antinuclear antibody positive	-	-	-	-	-	2 (0.0)	-	2 (0.0)
Antipyresis	1 (0.0)	-	-	-	-	-	-	1 (0.0)
Anuria	1 (0.0)	5 (0.0)	13 (0.1)	-	-	6 (0.1)	1 (0.4)	26 (0.1)
Anxiety	9 (0.2)	46 (0.3)	15 (0.1)	1 (0.3)	1 (0.2)	3 (0.1)	-	75 (0.2)
Anxiety disorder	-	1 (0.0)	-	-	-	-	-	1 (0.0)
Aorta hypoplasia	-	-	1 (0.0)	-	-	-	-	1 (0.0)
Aortic aneurysm	-	-	1 (0.0)	-	-	-	-	1 (0.0)
Aortic aneurysm rupture	1 (0.0)	-	2 (0.0)	-	-	-	-	3 (0.0)
Aortic dissection	-	-	1 (0.0)	-	-	-	-	1 (0.0)
Aortic intramural haematoma	-	-	1 (0.0)	-	-	-	-	1 (0.0)
Aortic stenosis	-	1 (0.0)	1 (0.0)	-	-	-	-	2 (0.0)
Aortic valve calcification	-	-	1 (0.0)	-	-	-	-	1 (0.0)
Aortic valve incompetence	-	-	3 (0.0)	-	-	-	-	3 (0.0)
Apallic syndrome	1 (0.0)	2 (0.0)	4 (0.0)	-	-	1 (0.0)	-	8 (0.0)
Apathy	-	6 (0.0)	1 (0.0)	-	-	1 (0.0)	-	8 (0.0)
Apgar score abnormal	-	-	2 (0.0)	-	-	-	-	2 (0.0)
Apgar score low	1 (0.0)	8 (0.1)	12 (0.0)	-	-	3 (0.1)	1 (0.4)	25 (0.1)

Aphasia	2 (0.1)	9 (0.1)	9 (0.0)	-	-	3 (0.1)	-	23 (0.0)
Aphonia	-	1 (0.0)	9 (0.0)	-	-	-	-	10 (0.0)
Aphthous ulcer	-	2 (0.0)	1 (0.0)	-	-	-	-	3 (0.0)
Aplasia	-	-	1 (0.0)	-	-	-	-	1 (0.0)
Apnoea	12 (0.3)	72 (0.5)	109 (0.4)	-	3 (0.6)	11 (0.2)	1 (0.4)	208 (0.4)
Apnoeic attack	-	1 (0.0)	4 (0.0)	-	-	1 (0.0)	-	6 (0.0)
Apparent death	-	1 (0.0)	-	-	-	-	-	1 (0.0)
Appendicitis	-	1 (0.0)	-	-	-	-	-	1 (0.0)
Application site erythema	-	2 (0.0)	12 (0.0)	-	-	-	-	14 (0.0)
Application site extravasation	-	-	8 (0.0)	-	-	-	-	8 (0.0)
Application site induration	-	-	1 (0.0)	-	-	-	-	1 (0.0)
Application site necrosis	-	-	1 (0.0)	-	-	-	-	1 (0.0)
Application site oedema	-	2 (0.0)	-	-	-	-	-	2 (0.0)
Application site pain	-	1 (0.0)	6 (0.0)	-	-	-	-	7 (0.0)
Application site swelling	-	-	9 (0.0)	-	-	-	-	9 (0.0)
Apraxia	-	-	-	-	-	1 (0.0)	-	1 (0.0)
Areflexia	-	6 (0.0)	7 (0.0)	-	-	1 (0.0)	-	14 (0.0)
Arrhythmia	12 (0.3)	24 (0.2)	83 (0.3)	1 (0.3)	1 (0.2)	10 (0.2)	-	131 (0.3)
Arrhythmia supraventricular	2 (0.1)	-	2 (0.0)	-	-	1 (0.0)	-	5 (0.0)
Arrhythmic storm	-	1 (0.0)	-	-	-	1 (0.0)	-	2 (0.0)
Arterial disorder	-	1 (0.0)	-	-	-	1 (0.0)	-	2 (0.0)
Arterial haemorrhage	1 (0.0)	-	-	-	-	-	-	1 (0.0)
Arterial injury	-	-	1 (0.0)	-	-	-	-	1 (0.0)
Arterial occlusive disease	-	-	1 (0.0)	-	-	-	-	1 (0.0)
Arterial thrombosis	-	-	-	-	-	1 (0.0)	-	1 (0.0)
Arteriosclerosis	-	1 (0.0)	2 (0.0)	-	-	-	-	3 (0.0)
Arteriosclerosis coronary artery	-	1 (0.0)	-	-	-	-	-	1 (0.0)
Arteriospasm coronary	24 (0.6)	3 (0.0)	26 (0.1)	1 (0.3)	2 (0.4)	1 (0.0)	-	57 (0.1)
Arteriovenous fistula	-	-	1 (0.0)	-	-	-	-	1 (0.0)
Arthralgia	-	20 (0.1)	11 (0.0)	-	-	3 (0.1)	-	34 (0.1)
Arthritis	-	1 (0.0)	-	-	-	-	-	1 (0.0)
Arthropathy	-	5 (0.0)	2 (0.0)	-	-	1 (0.0)	-	8 (0.0)

Arthropod bite	-	1 (0.0)	-	-	-	-	-	1 (0.0)
Ascites	2 (0.1)	6 (0.0)	5 (0.0)	-	-	1 (0.0)	-	14 (0.0)
Aspartate aminotransferase abnormal	-	1 (0.0)	-	-	-	-	-	1 (0.0)
Aspartate aminotransferase increased	3 (0.1)	19 (0.1)	27 (0.1)	-	-	7 (0.1)	-	56 (0.1)
Asphyxia	-	5 (0.0)	4 (0.0)	-	-	1 (0.0)	-	10 (0.0)
Aspiration	4 (0.1)	16 (0.1)	25 (0.1)	-	-	7 (0.1)	-	52 (0.1)
Aspiration bone marrow abnormal	-	-	-	-	-	1 (0.0)	-	1 (0.0)
Asterixis	-	1 (0.0)	1 (0.0)	-	-	-	-	2 (0.0)
Asthenia	3 (0.1)	42 (0.3)	61 (0.2)	-	-	6 (0.1)	-	112 (0.2)
Asthenopia	-	-	1 (0.0)	-	-	-	-	1 (0.0)
Asthma	-	4 (0.0)	16 (0.1)	-	-	2 (0.0)	-	22 (0.0)
Asthmatic crisis	-	1 (0.0)	6 (0.0)	-	-	1 (0.0)	-	8 (0.0)
Astigmatism	-	1 (0.0)	-	-	-	-	-	1 (0.0)
Ataxia	1 (0.0)	11 (0.1)	-	-	-	3 (0.1)	-	15 (0.0)
Atelectasis	2 (0.1)	2 (0.0)	19 (0.1)	-	1 (0.2)	2 (0.0)	-	26 (0.1)
Atelectasis neonatal	-	1 (0.0)	-	-	-	-	-	1 (0.0)
Athetosis	-	1 (0.0)	-	-	-	-	-	1 (0.0)
Atonic urinary bladder	-	1 (0.0)	-	-	-	-	1 (0.4)	2 (0.0)
Atrial fibrillation	6 (0.2)	10 (0.1)	63 (0.3)	-	1 (0.2)	8 (0.2)	-	88 (0.2)
Atrial flutter	-	1 (0.0)	2 (0.0)	-	-	-	-	3 (0.0)
Atrial septal defect	-	1 (0.0)	2 (0.0)	-	-	-	-	3 (0.0)
Atrioventricular block	19 (0.5)	7 (0.0)	17 (0.1)	-	-	7 (0.1)	-	50 (0.1)
Atrioventricular block complete	36 (0.9)	6 (0.0)	49 (0.2)	-	1 (0.2)	1 (0.0)	1 (0.4)	94 (0.2)
Atrioventricular block first degree	7 (0.2)	1 (0.0)	4 (0.0)	-	-	1 (0.0)	-	13 (0.0)
Atrioventricular block second degree	21 (0.5)	1 (0.0)	12 (0.0)	-	3 (0.6)	-	-	37 (0.1)
Atrioventricular dissociation	2 (0.1)	-	3 (0.0)	-	-	1 (0.0)	-	6 (0.0)
Atrophy	-	1 (0.0)	-	-	-	-	-	1 (0.0)
Attention deficit hyperactivity disorder	-	2 (0.0)	2 (0.0)	-	-	-	-	4 (0.0)
Auditory disorder	-	-	-	-	-	1 (0.0)	-	1 (0.0)
Autism spectrum disorder	-	1 (0.0)	-	-	-	-	-	1 (0.0)
Autoimmune disorder	-	1 (0.0)	-	-	-	1 (0.0)	-	2 (0.0)
Autoimmune hepatitis	-	1 (0.0)	2 (0.0)	-	-	-	-	3 (0.0)

Autoimmune thyroiditis	-	2 (0.0)	-	-	-	-	-	2 (0.0)
Automatism	-	-	-	-	-	1 (0.0)	-	1 (0.0)
Autonomic dysreflexia	-	1 (0.0)	1 (0.0)	-	-	-	-	2 (0.0)
Autonomic nervous system imbalance	1 (0.0)	3 (0.0)	1 (0.0)	-	1 (0.2)	5 (0.1)	-	11 (0.0)
Axillary pain	-	-	1 (0.0)	-	-	-	-	1 (0.0)
Axonal neuropathy	-	2 (0.0)	-	-	-	-	-	2 (0.0)
Azotaemia	-	1 (0.0)	1 (0.0)	-	-	-	-	2 (0.0)
Bacillus infection	-	1 (0.0)	-	-	-	-	-	1 (0.0)
Back injury	-	1 (0.0)	-	-	-	-	-	1 (0.0)
Back pain	-	7 (0.0)	31 (0.1)	-	-	4 (0.1)	-	42 (0.1)
Bacteraemia	-	7 (0.0)	10 (0.0)	-	-	2 (0.0)	-	19 (0.0)
Bacterial infection	-	6 (0.0)	1 (0.0)	-	-	-	-	7 (0.0)
Bacterial sepsis	-	-	5 (0.0)	-	-	-	-	5 (0.0)
Bacterial test positive	-	1 (0.0)	1 (0.0)	-	-	-	-	2 (0.0)
Balance disorder	-	11 (0.1)	5 (0.0)	-	-	-	-	16 (0.0)
Ballismus	-	-	1 (0.0)	-	-	-	-	1 (0.0)
Bandaemia	-	1 (0.0)	-	-	-	-	-	1 (0.0)
Barbiturates positive	1 (0.0)	-	-	-	-	-	-	1 (0.0)
Barotrauma	-	2 (0.0)	-	-	-	-	-	2 (0.0)
Basal ganglion degeneration	-	-	1 (0.0)	-	-	-	-	1 (0.0)
Base excess	-	-	1 (0.0)	-	-	-	-	1 (0.0)
Base excess negative	-	-	1 (0.0)	-	-	-	-	1 (0.0)
Baseline foetal heart rate variability disorder	1 (0.0)	1 (0.0)	7 (0.0)	-	1 (0.2)	-	-	10 (0.0)
Bedridden	-	1 (0.0)	2 (0.0)	-	-	-	-	3 (0.0)
Behaviour disorder	-	31 (0.2)	7 (0.0)	1 (0.3)	-	1 (0.0)	-	40 (0.1)
Benign familial neonatal convulsions	-	1 (0.0)	-	-	-	-	-	1 (0.0)
Benign prostatic hyperplasia	-	1 (0.0)	-	-	-	-	-	1 (0.0)
Beta haemolytic streptococcal infection	-	-	1 (0.0)	-	-	-	-	1 (0.0)
Bezoar	-	1 (0.0)	-	-	-	-	-	1 (0.0)
Bezold-Jarisch reflex	-	1 (0.0)	3 (0.0)	-	-	-	-	4 (0.0)
Bicytopenia	-	1 (0.0)	-	-	-	1 (0.0)	-	2 (0.0)
Bile duct stone	1 (0.0)	-	-	-	-	-	-	1 (0.0)

Biliary cast syndrome	-	-	1 (0.0)	-	-	-	-	1 (0.0)
Biliary colic	-	-	3 (0.0)	-	-	-	-	3 (0.0)
Biliary dilatation	-	-	-	-	-	1 (0.0)	-	1 (0.0)
Biliary obstruction	-	-	1 (0.0)	-	-	-	-	1 (0.0)
Biliary tract disorder	-	-	1 (0.0)	-	-	-	-	1 (0.0)
Bilirubin conjugated increased	-	1 (0.0)	1 (0.0)	-	-	-	-	2 (0.0)
Bipolar disorder	-	5 (0.0)	1 (0.0)	-	-	1 (0.0)	-	7 (0.0)
Bispectral index decreased	-	2 (0.0)	4 (0.0)	-	-	-	-	6 (0.0)
Bladder dilatation	-	1 (0.0)	-	-	-	-	-	1 (0.0)
Bladder disorder	-	5 (0.0)	1 (0.0)	-	-	-	-	6 (0.0)
Bladder diverticulum	-	1 (0.0)	-	-	-	-	-	1 (0.0)
Bladder dysfunction	-	1 (0.0)	1 (0.0)	-	-	-	-	2 (0.0)
Bladder pain	-	-	1 (0.0)	-	-	1 (0.0)	-	2 (0.0)
Bladder spasm	-	-	2 (0.0)	-	-	-	-	2 (0.0)
Blepharospasm	-	4 (0.0)	-	-	-	-	-	4 (0.0)
Blindness	-	-	4 (0.0)	-	-	1 (0.0)	1 (0.4)	6 (0.0)
Blindness cortical	-	-	-	-	-	-	1 (0.4)	1 (0.0)
Blindness transient	-	1 (0.0)	2 (0.0)	-	-	-	-	3 (0.0)
Blindness unilateral	-	1 (0.0)	2 (0.0)	-	-	-	-	3 (0.0)
Blister	1 (0.0)	10 (0.1)	9 (0.0)	-	-	5 (0.1)	-	25 (0.1)
Blood albumin decreased	-	4 (0.0)	4 (0.0)	-	-	-	-	8 (0.0)
Blood alkaline phosphatase abnormal	-	1 (0.0)	-	-	-	-	-	1 (0.0)
Blood alkaline phosphatase increased	3 (0.1)	6 (0.0)	10 (0.0)	-	-	1 (0.0)	-	20 (0.0)
Blood bicarbonate decreased	-	1 (0.0)	1 (0.0)	-	-	2 (0.0)	-	4 (0.0)
Blood bicarbonate increased	1 (0.0)	2 (0.0)	-	-	-	-	-	3 (0.0)
Blood bilirubin increased	1 (0.0)	7 (0.0)	9 (0.0)	-	-	1 (0.0)	1 (0.4)	19 (0.0)
Blood bilirubin unconjugated increased	1 (0.0)	-	2 (0.0)	-	-	-	-	3 (0.0)
Blood blister	-	-	1 (0.0)	-	-	-	-	1 (0.0)
Blood calcium abnormal	-	1 (0.0)	-	-	-	-	-	1 (0.0)
Blood calcium decreased	-	1 (0.0)	2 (0.0)	-	-	1 (0.0)	-	4 (0.0)
Blood calcium increased	-	-	1 (0.0)	-	-	-	-	1 (0.0)
Blood chloride decreased	-	-	1 (0.0)	-	-	-	-	1 (0.0)

Blood chloride increased	1 (0.0)	4 (0.0)	-	-	-	3 (0.1)	-	8 (0.0)
Blood cholesterol increased	-	1 (0.0)	2 (0.0)	-	-	-	-	3 (0.0)
Blood cholinesterase decreased	-	1 (0.0)	-	-	-	-	-	1 (0.0)
Blood creatine increased	-	1 (0.0)	4 (0.0)	-	-	1 (0.0)	-	6 (0.0)
Blood creatine phosphokinase abnormal	1 (0.0)	1 (0.0)	1 (0.0)	-	-	-	-	3 (0.0)
Blood creatine phosphokinase increased	9 (0.2)	20 (0.1)	96 (0.4)	-	2 (0.4)	13 (0.3)	-	140 (0.3)
Blood creatine phosphokinase MB increased	-	-	1 (0.0)	-	-	-	-	1 (0.0)
Blood creatine phosphokinase normal	-	-	1 (0.0)	-	-	-	-	1 (0.0)
Blood creatinine increased	3 (0.1)	14 (0.1)	21 (0.1)	-	-	5 (0.1)	-	43 (0.1)
Blood disorder	-	-	1 (0.0)	-	-	-	-	1 (0.0)
Blood fibrinogen increased	-	-	1 (0.0)	-	-	-	-	1 (0.0)
Blood folate decreased	-	1 (0.0)	-	-	-	-	-	1 (0.0)
Blood gases	-	-	1 (0.0)	-	-	-	-	1 (0.0)
Blood gases abnormal	-	-	1 (0.0)	-	-	2 (0.0)	-	3 (0.0)
Blood glucose abnormal	-	-	1 (0.0)	-	-	-	-	1 (0.0)
Blood glucose decreased	2 (0.1)	-	2 (0.0)	1 (0.3)	-	-	-	5 (0.0)
Blood glucose fluctuation	-	3 (0.0)	-	-	-	-	-	3 (0.0)
Blood glucose increased	1 (0.0)	8 (0.1)	5 (0.0)	-	-	-	-	14 (0.0)
Blood immunoglobulin E increased	-	-	1 (0.0)	-	-	-	-	1 (0.0)
Blood immunoglobulin G decreased	-	-	1 (0.0)	-	-	-	-	1 (0.0)
Blood immunoglobulin G increased	-	-	1 (0.0)	-	-	-	-	1 (0.0)
Blood lactate dehydrogenase abnormal	-	1 (0.0)	-	-	-	-	-	1 (0.0)
Blood lactate dehydrogenase increased	-	3 (0.0)	12 (0.0)	-	-	2 (0.0)	-	17 (0.0)
Blood lactic acid decreased	-	-	-	-	-	1 (0.0)	-	1 (0.0)
Blood lactic acid increased	1 (0.0)	4 (0.0)	14 (0.1)	-	-	4 (0.1)	-	23 (0.0)
Blood loss anaemia	-	1 (0.0)	-	-	-	-	-	1 (0.0)
Blood loss anaemia neonatal	-	-	1 (0.0)	-	-	-	-	1 (0.0)
Blood magnesium decreased	-	3 (0.0)	-	-	-	1 (0.0)	-	4 (0.0)
Blood osmolarity decreased	-	1 (0.0)	-	-	1 (0.2)	-	-	2 (0.0)
Blood osmolarity increased	-	1 (0.0)	-	-	-	-	-	1 (0.0)
Blood pH abnormal	-	1 (0.0)	-	-	-	-	-	1 (0.0)
Blood pH decreased	1 (0.0)	5 (0.0)	3 (0.0)	-	-	4 (0.1)	-	13 (0.0)

Blood pH increased	-	1 (0.0)	-	-	-	2 (0.0)	-	3 (0.0)
Blood phosphorus decreased	-	2 (0.0)	-	-	-	-	-	2 (0.0)
Blood phosphorus increased	-	-	1 (0.0)	-	-	-	-	1 (0.0)
Blood potassium decreased	-	1 (0.0)	1 (0.0)	-	-	1 (0.0)	-	3 (0.0)
Blood potassium increased	1 (0.0)	1 (0.0)	5 (0.0)	-	-	2 (0.0)	-	9 (0.0)
Blood pressure abnormal	1 (0.0)	2 (0.0)	1 (0.0)	-	-	-	-	4 (0.0)
Blood pressure ambulatory increased	-	-	1 (0.0)	-	-	-	-	1 (0.0)
Blood pressure decreased	77 (2.0)	104 (0.7)	292 (1.2)	2 (0.6)	4 (0.8)	33 (0.7)	-	512 (1.0)
Blood pressure diastolic decreased	1 (0.0)	-	2 (0.0)	-	-	-	-	3 (0.0)
Blood pressure fluctuation	2 (0.1)	3 (0.0)	7 (0.0)	-	-	3 (0.1)	1 (0.4)	16 (0.0)
Blood pressure immeasurable	-	-	10 (0.0)	-	2 (0.4)	2 (0.0)	-	14 (0.0)
Blood pressure increased	17 (0.4)	33 (0.2)	62 (0.2)	1 (0.3)	1 (0.2)	7 (0.1)	1 (0.4)	122 (0.2)
Blood pressure measurement	-	-	1 (0.0)	-	-	-	-	1 (0.0)
Blood pressure normal	-	-	1 (0.0)	-	-	-	-	1 (0.0)
Blood pressure systolic	-	-	2 (0.0)	-	-	-	-	2 (0.0)
Blood pressure systolic decreased	17 (0.4)	7 (0.0)	26 (0.1)	-	-	8 (0.2)	-	58 (0.1)
Blood pressure systolic increased	1 (0.0)	4 (0.0)	6 (0.0)	-	-	-	-	11 (0.0)
Blood prolactin increased	-	4 (0.0)	1 (0.0)	-	-	-	-	5 (0.0)
Blood sodium	1 (0.0)	-	-	-	-	-	-	1 (0.0)
Blood sodium decreased	-	2 (0.0)	3 (0.0)	-	-	-	-	5 (0.0)
Blood sodium increased	2 (0.1)	3 (0.0)	3 (0.0)	-	-	-	-	8 (0.0)
Blood test abnormal	-	1 (0.0)	1 (0.0)	1 (0.3)	-	-	-	3 (0.0)
Blood triglycerides abnormal	-	-	2 (0.0)	-	-	-	-	2 (0.0)
Blood triglycerides increased	-	-	31 (0.1)	-	-	1 (0.0)	-	32 (0.1)
Blood urea increased	-	4 (0.0)	4 (0.0)	-	-	2 (0.0)	-	10 (0.0)
Blood uric acid increased	2 (0.1)	1 (0.0)	1 (0.0)	-	-	-	-	4 (0.0)
Blood urine present	-	1 (0.0)	1 (0.0)	-	-	-	-	2 (0.0)
Bloody discharge	-	-	1 (0.0)	-	-	1 (0.0)	-	2 (0.0)
Blue toe syndrome	-	-	1 (0.0)	-	-	-	-	1 (0.0)
Body height decreased	-	1 (0.0)	-	-	-	-	-	1 (0.0)
Body temperature abnormal	-	-	1 (0.0)	-	-	-	-	1 (0.0)
Body temperature decreased	1 (0.0)	2 (0.0)	1 (0.0)	1 (0.3)	-	-	-	5 (0.0)

Body temperature fluctuation	-	-	3 (0.0)	-	-	-	-	3 (0.0)
Body temperature increased	5 (0.1)	13 (0.1)	44 (0.2)	-	1 (0.2)	5 (0.1)	-	68 (0.1)
Bone cement implantation syndrome	-	-	1 (0.0)	-	-	-	-	1 (0.0)
Bone cyst excision	-	-	1 (0.0)	-	-	-	-	1 (0.0)
Bone density decreased	-	1 (0.0)	-	-	-	-	-	1 (0.0)
Bone marrow failure	-	-	1 (0.0)	-	-	-	-	1 (0.0)
Bone pain	1 (0.0)	4 (0.0)	1 (0.0)	-	-	-	-	6 (0.0)
Boredom	-	1 (0.0)	-	-	-	-	-	1 (0.0)
Brachial plexopathy	-	-	1 (0.0)	-	-	-	-	1 (0.0)
Bradyarrhythmia	6 (0.2)	-	11 (0.0)	1 (0.3)	-	2 (0.0)	-	20 (0.0)
Bradycardia	544 (14.2)	105 (0.7)	341 (1.4)	12 (3.6)	33 (6.6)	43 (0.9)	4 (1.5)	1082 (2.2)
Bradycardia foetal	-	2 (0.0)	14 (0.1)	-	-	3 (0.1)	-	19 (0.0)
Bradycardia neonatal	1 (0.0)	-	-	-	-	2 (0.0)	-	3 (0.0)
Bradyphrenia	-	3 (0.0)	2 (0.0)	-	-	-	-	5 (0.0)
Bradypnoea	1 (0.0)	19 (0.1)	5 (0.0)	-	3 (0.6)	-	-	28 (0.1)
Brain death	-	10 (0.1)	5 (0.0)	-	-	3 (0.1)	-	18 (0.0)
Brain fog	-	-	2 (0.0)	-	-	-	-	2 (0.0)
Brain herniation	-	2 (0.0)	-	-	-	1 (0.0)	-	3 (0.0)
Brain hypoxia	-	4 (0.0)	2 (0.0)	-	-	-	-	6 (0.0)
Brain injury	1 (0.0)	6 (0.0)	23 (0.1)	-	-	2 (0.0)	-	32 (0.1)
Brain malformation	-	-	-	-	-	1 (0.0)	-	1 (0.0)
Brain natriuretic peptide increased	-	1 (0.0)	-	-	-	-	-	1 (0.0)
Brain neoplasm malignant	-	1 (0.0)	-	-	-	-	-	1 (0.0)
Brain oedema	-	16 (0.1)	20 (0.1)	-	-	2 (0.0)	1 (0.4)	39 (0.1)
Brain operation	-	-	1 (0.0)	-	-	-	-	1 (0.0)
Brain scan abnormal	-	-	1 (0.0)	-	-	-	-	1 (0.0)
Brain stem infarction	-	-	2 (0.0)	-	-	-	-	2 (0.0)
Brain stem syndrome	-	2 (0.0)	1 (0.0)	-	-	-	-	3 (0.0)
Breakthrough pain	-	1 (0.0)	-	-	-	-	-	1 (0.0)
Breast cancer	-	2 (0.0)	-	-	-	-	-	2 (0.0)
Breast cancer metastatic	-	5 (0.0)	-	-	-	-	-	5 (0.0)
Breast cancer recurrent	-	-	1 (0.0)	-	-	-	-	1 (0.0)

Breast discharge	-	-	1 (0.0)	-	-	-	-	1 (0.0)
Breast feeding	-	-	2 (0.0)	-	-	-	-	2 (0.0)
Breast haematoma	-	-	1 (0.0)	-	-	-	-	1 (0.0)
Breast milk discolouration	-	-	5 (0.0)	-	-	-	-	5 (0.0)
Breast operation	-	1 (0.0)	-	-	-	-	-	1 (0.0)
Breast pain	-	-	2 (0.0)	-	-	-	-	2 (0.0)
Breath odour	-	-	1 (0.0)	-	-	-	-	1 (0.0)
Breath sounds abnormal	1 (0.0)	3 (0.0)	5 (0.0)	-	-	1 (0.0)	-	10 (0.0)
Breath sounds absent	-	1 (0.0)	1 (0.0)	-	-	-	-	2 (0.0)
Brief resolved unexplained event	-	1 (0.0)	-	-	-	-	-	1 (0.0)
Bronchial aspiration procedure	-	1 (0.0)	-	-	-	-	-	1 (0.0)
Bronchial disorder	-	1 (0.0)	-	-	-	-	-	1 (0.0)
Bronchial hyperreactivity	-	-	1 (0.0)	-	-	-	-	1 (0.0)
Bronchial obstruction	-	-	3 (0.0)	-	-	-	-	3 (0.0)
Bronchial oedema	-	-	1 (0.0)	-	-	-	-	1 (0.0)
Bronchial secretion retention	1 (0.0)	-	1 (0.0)	-	-	-	-	2 (0.0)
Bronchiectasis	-	1 (0.0)	-	-	-	-	-	1 (0.0)
Bronchiolitis	-	1 (0.0)	-	-	-	-	-	1 (0.0)
Bronchitis	-	1 (0.0)	3 (0.0)	-	-	-	-	4 (0.0)
Bronchoalveolar lavage abnormal	-	1 (0.0)	-	-	-	-	-	1 (0.0)
Bronchopulmonary aspergillosis	-	-	1 (0.0)	-	-	-	-	1 (0.0)
Bronchopulmonary dysplasia	1 (0.0)	1 (0.0)	-	-	-	-	-	2 (0.0)
Bronchospasm	5 (0.1)	22 (0.1)	418 (1.7)	-	-	71 (1.5)	-	516 (1.0)
Bronchospasm paradoxical	-	1 (0.0)	5 (0.0)	-	-	1 (0.0)	-	7 (0.0)
Bronchostenosis	-	2 (0.0)	4 (0.0)	-	-	-	-	6 (0.0)
Brugada syndrome	-	1 (0.0)	8 (0.0)	-	1 (0.2)	1 (0.0)	-	11 (0.0)
Bruxism	-	3 (0.0)	1 (0.0)	-	-	-	-	4 (0.0)
Bulimia nervosa	-	2 (0.0)	-	-	-	-	-	2 (0.0)
Bundle branch block	-	-	2 (0.0)	-	-	-	-	2 (0.0)
Bundle branch block left	-	2 (0.0)	11 (0.0)	-	-	1 (0.0)	-	14 (0.0)
Bundle branch block right	1 (0.0)	6 (0.0)	9 (0.0)	-	2 (0.4)	-	-	18 (0.0)
Burning sensation	-	7 (0.0)	12 (0.0)	-	-	-	-	19 (0.0)

Burns first degree	-	-	1 (0.0)	-	-	-	-	1 (0.0)
Bursitis	-	-	1 (0.0)	-	-	-	-	1 (0.0)
CADASIL	-	-	1 (0.0)	-	-	-	-	1 (0.0)
Caesarean section	-	-	8 (0.0)	-	-	1 (0.0)	-	9 (0.0)
Cafe au lait spots	-	1 (0.0)	-	-	-	-	-	1 (0.0)
Calcinosis	-	2 (0.0)	-	-	-	-	-	2 (0.0)
Calcium ionised decreased	1 (0.0)	-	1 (0.0)	-	-	-	-	2 (0.0)
Candida infection	1 (0.0)	1 (0.0)	-	-	-	1 (0.0)	-	3 (0.0)
Candida pneumonia	-	1 (0.0)	-	-	-	-	-	1 (0.0)
Candida test positive	-	1 (0.0)	-	-	-	-	-	1 (0.0)
Capillary disorder	-	-	-	-	-	1 (0.0)	-	1 (0.0)
Capillary nail refill test abnormal	-	-	1 (0.0)	-	-	-	-	1 (0.0)
Capillary permeability increased	-	-	1 (0.0)	-	-	-	-	1 (0.0)
Capnogram abnormal	-	-	2 (0.0)	-	-	-	-	2 (0.0)
Carbohydrate antigen 125 increased	-	-	-	-	-	1 (0.0)	-	1 (0.0)
Carbon dioxide abnormal	-	-	1 (0.0)	-	-	-	-	1 (0.0)
Carbon dioxide decreased	-	-	4 (0.0)	-	-	-	-	4 (0.0)
Carbon dioxide increased	1 (0.0)	-	2 (0.0)	-	-	1 (0.0)	-	4 (0.0)
Carcinoid crisis	-	-	1 (0.0)	-	-	3 (0.1)	-	4 (0.0)
Carcinoid heart disease	-	-	-	-	-	1 (0.0)	-	1 (0.0)
Carcinoid syndrome	-	-	-	-	-	1 (0.0)	-	1 (0.0)
Carcinoid tumour	-	2 (0.0)	-	-	-	-	-	2 (0.0)
Cardiac arrest	187 (4.9)	117 (0.8)	429 (1.7)	5 (1.5)	20 (4.0)	57 (1.2)	6 (2.3)	821 (1.7)
Cardiac arrest neonatal	2 (0.1)	-	-	-	-	-	-	2 (0.0)
Cardiac contractility decreased	-	-	4 (0.0)	-	-	1 (0.0)	-	5 (0.0)
Cardiac death	-	1 (0.0)	-	-	-	-	-	1 (0.0)
Cardiac disorder	-	7 (0.0)	15 (0.1)	-	1 (0.2)	-	-	23 (0.0)
Cardiac dysfunction	-	2 (0.0)	4 (0.0)	-	-	1 (0.0)	-	7 (0.0)
Cardiac failure	2 (0.1)	15 (0.1)	48 (0.2)	2 (0.6)	2 (0.4)	11 (0.2)	-	80 (0.2)
Cardiac failure acute	4 (0.1)	3 (0.0)	10 (0.0)	-	-	-	-	17 (0.0)
Cardiac failure congestive	2 (0.1)	-	6 (0.0)	-	-	-	-	8 (0.0)
Cardiac fibrillation	-	3 (0.0)	-	-	-	-	-	3 (0.0)

Cardiac flutter	-	-	2 (0.0)	-	-	-	-	2 (0.0)
Cardiac function disturbance postoperative	-	-	1 (0.0)	-	-	-	-	1 (0.0)
Cardiac index decreased	-	-	2 (0.0)	-	-	-	-	2 (0.0)
Cardiac massage	-	-	3 (0.0)	-	-	-	-	3 (0.0)
Cardiac murmur	-	3 (0.0)	-	-	-	-	-	3 (0.0)
Cardiac output decreased	3 (0.1)	1 (0.0)	3 (0.0)	-	-	2 (0.0)	-	9 (0.0)
Cardiac pacemaker insertion	-	-	2 (0.0)	-	-	-	-	2 (0.0)
Cardiac perforation	-	1 (0.0)	1 (0.0)	-	-	-	-	2 (0.0)
Cardiac septal hypertrophy	-	1 (0.0)	-	-	-	-	-	1 (0.0)
Cardiac tamponade	3 (0.1)	-	1 (0.0)	-	-	3 (0.1)	-	7 (0.0)
Cardiac valve abscess	-	-	-	-	-	1 (0.0)	-	1 (0.0)
Cardiac valve rupture	-	-	1 (0.0)	-	-	-	-	1 (0.0)
Cardiac valve vegetation	-	-	-	-	-	1 (0.0)	-	1 (0.0)
Cardiac ventricular disorder	-	1 (0.0)	-	-	-	-	-	1 (0.0)
Cardiac ventricular thrombosis	-	-	1 (0.0)	-	-	-	-	1 (0.0)
Cardioactive drug level increased	-	-	1 (0.0)	-	-	-	-	1 (0.0)
Cardiogenic shock	5 (0.1)	8 (0.1)	44 (0.2)	3 (0.9)	-	5 (0.1)	1 (0.4)	66 (0.1)
Cardiomegaly	-	6 (0.0)	5 (0.0)	-	-	4 (0.1)	-	15 (0.0)
Cardiomyopathy	1 (0.0)	1 (0.0)	16 (0.1)	1 (0.3)	-	-	-	19 (0.0)
Cardiopulmonary bypass	-	-	-	-	-	1 (0.0)	-	1 (0.0)
Cardiopulmonary failure	2 (0.1)	4 (0.0)	4 (0.0)	-	1 (0.2)	2 (0.0)	-	13 (0.0)
Cardio-respiratory arrest	15 (0.4)	57 (0.4)	111 (0.4)	1 (0.3)	4 (0.8)	18 (0.4)	3 (1.1)	209 (0.4)
Cardio-respiratory distress	-	-	2 (0.0)	-	-	1 (0.0)	-	3 (0.0)
Cardiotoxicity	-	3 (0.0)	2 (0.0)	-	-	2 (0.0)	-	7 (0.0)
Cardiovascular disorder	1 (0.0)	4 (0.0)	13 (0.1)	-	-	1 (0.0)	-	19 (0.0)
Cardiovascular insufficiency	-	5 (0.0)	8 (0.0)	-	-	1 (0.0)	-	14 (0.0)
Cardioversion	-	1 (0.0)	8 (0.0)	-	-	-	-	9 (0.0)
Carnitine decreased	-	-	1 (0.0)	-	-	-	-	1 (0.0)
Carotid artery occlusion	1 (0.0)	-	1 (0.0)	-	-	-	-	2 (0.0)
Carotid artery stenosis	-	-	1 (0.0)	-	-	-	-	1 (0.0)
Carpal tunnel syndrome	-	-	2 (0.0)	-	-	-	-	2 (0.0)
Cat scratch disease	-	1 (0.0)	-	-	-	-	-	1 (0.0)

Catabolic state	-	-	1 (0.0)	-	-	-	-	1 (0.0)
Cataract	-	1 (0.0)	-	-	-	-	-	1 (0.0)
Catatonia	3 (0.1)	8 (0.1)	3 (0.0)	-	-	-	1 (0.4)	15 (0.0)
Catecholamine crisis	-	1 (0.0)	-	-	-	-	-	1 (0.0)
Catheter site erythema	-	1 (0.0)	-	-	-	-	-	1 (0.0)
Catheter site extravasation	1 (0.0)	-	1 (0.0)	-	-	-	-	2 (0.0)
Catheter site haemorrhage	-	-	1 (0.0)	-	-	-	-	1 (0.0)
Catheter site pain	-	-	3 (0.0)	-	-	-	-	3 (0.0)
Catheter site thrombosis	-	1 (0.0)	-	-	-	-	-	1 (0.0)
Catheter site vesicles	-	1 (0.0)	-	-	-	-	-	1 (0.0)
Cauda equina syndrome	-	-	1 (0.0)	-	-	-	-	1 (0.0)
Cell death	-	1 (0.0)	2 (0.0)	-	-	-	-	3 (0.0)
Cellulitis	-	4 (0.0)	2 (0.0)	-	-	-	-	6 (0.0)
Cementoplasty	-	-	1 (0.0)	-	-	-	-	1 (0.0)
Central nervous system lesion	-	2 (0.0)	2 (0.0)	-	-	-	-	4 (0.0)
Central nervous system stimulation	-	-	1 (0.0)	-	-	-	-	1 (0.0)
Central sleep apnoea syndrome	3 (0.1)	-	-	-	-	-	-	3 (0.0)
Central venous pressure decreased	-	-	1 (0.0)	-	-	-	-	1 (0.0)
Central venous pressure increased	1 (0.0)	-	1 (0.0)	-	-	1 (0.0)	-	3 (0.0)
Central-alveolar hypoventilation	-	-	2 (0.0)	-	-	1 (0.0)	-	3 (0.0)
Cerebellar atrophy	-	1 (0.0)	1 (0.0)	-	-	-	-	2 (0.0)
Cerebellar hypoplasia	-	1 (0.0)	-	-	-	1 (0.0)	-	2 (0.0)
Cerebellar infarction	1 (0.0)	-	3 (0.0)	-	-	-	-	4 (0.0)
Cerebellar ischaemia	-	-	1 (0.0)	-	-	-	-	1 (0.0)
Cerebral artery embolism	-	-	1 (0.0)	-	-	-	-	1 (0.0)
Cerebral artery occlusion	3 (0.1)	-	-	-	-	-	-	3 (0.0)
Cerebral atrophy	-	13 (0.1)	-	-	-	-	-	13 (0.0)
Cerebral congestion	-	1 (0.0)	-	-	-	-	-	1 (0.0)
Cerebral disorder	1 (0.0)	4 (0.0)	2 (0.0)	-	-	-	-	7 (0.0)
Cerebral haematoma	-	-	1 (0.0)	-	-	-	-	1 (0.0)
Cerebral haemorrhage	-	5 (0.0)	6 (0.0)	1 (0.3)	-	1 (0.0)	-	13 (0.0)
Cerebral hypoperfusion	1 (0.0)	-	4 (0.0)	-	-	1 (0.0)	-	6 (0.0)

Cerebral infarction	8 (0.2)	5 (0.0)	15 (0.1)	-	-	-	1 (0.4)	29 (0.1)
Cerebral ischaemia	-	4 (0.0)	7 (0.0)	-	-	2 (0.0)	-	13 (0.0)
Cerebral palsy	1 (0.0)	-	-	-	-	-	-	1 (0.0)
Cerebral salt-wasting syndrome	-	1 (0.0)	-	-	-	-	-	1 (0.0)
Cerebral thrombosis	1 (0.0)	-	-	-	-	-	-	1 (0.0)
Cerebral venous sinus thrombosis	-	-	2 (0.0)	-	-	-	-	2 (0.0)
Cerebral venous thrombosis	-	-	2 (0.0)	-	-	-	-	2 (0.0)
Cerebral ventricle dilatation	-	1 (0.0)	-	-	1 (0.2)	-	-	2 (0.0)
Cerebrospinal fluid leakage	-	1 (0.0)	1 (0.0)	-	-	-	-	2 (0.0)
Cerebrovascular accident	3 (0.1)	8 (0.1)	8 (0.0)	1 (0.3)	-	-	-	20 (0.0)
Cerebrovascular arteriovenous malformation	2 (0.1)	-	-	-	-	-	-	2 (0.0)
Cerebrovascular insufficiency	2 (0.1)	-	1 (0.0)	-	-	-	-	3 (0.0)
Cervical cord compression	-	-	1 (0.0)	-	-	-	-	1 (0.0)
Cervix disorder	-	1 (0.0)	-	-	-	-	-	1 (0.0)
Cervix oedema	-	-	1 (0.0)	-	-	-	-	1 (0.0)
Change in seizure presentation	-	3 (0.0)	-	-	-	1 (0.0)	-	4 (0.0)
Cheilitis	-	-	1 (0.0)	-	-	1 (0.0)	-	2 (0.0)
Chemical burn	-	-	1 (0.0)	-	-	-	-	1 (0.0)
Chemical poisoning	-	1 (0.0)	-	-	-	-	-	1 (0.0)
Chemical submission	-	1 (0.0)	-	-	-	-	-	1 (0.0)
Chest discomfort	-	18 (0.1)	13 (0.1)	1 (0.3)	-	4 (0.1)	-	36 (0.1)
Chest expansion decreased	-	-	-	-	-	1 (0.0)	-	1 (0.0)
Chest pain	2 (0.1)	23 (0.2)	13 (0.1)	-	-	1 (0.0)	-	39 (0.1)
Chest X-ray abnormal	-	1 (0.0)	2 (0.0)	-	-	-	-	3 (0.0)
Cheyne-Stokes respiration	-	3 (0.0)	2 (0.0)	-	-	-	-	5 (0.0)
Childhood depression	-	-	-	-	-	1 (0.0)	-	1 (0.0)
Chills	4 (0.1)	35 (0.2)	230 (0.9)	-	-	22 (0.5)	-	291 (0.6)
Choking	-	8 (0.1)	4 (0.0)	-	-	-	-	12 (0.0)
Choking sensation	-	1 (0.0)	-	-	-	-	-	1 (0.0)
Cholangitis	1 (0.0)	1 (0.0)	-	-	-	-	-	2 (0.0)
Cholangitis sclerosing	-	-	1 (0.0)	-	-	-	-	1 (0.0)
Cholecystectomy	-	1 (0.0)	1 (0.0)	-	-	-	-	2 (0.0)

Cholecystitis	-	-	4 (0.0)	-	-	-	-	4 (0.0)
Cholecystitis acute	-	1 (0.0)	-	-	-	1 (0.0)	-	2 (0.0)
Cholelithiasis	-	3 (0.0)	2 (0.0)	-	-	-	-	5 (0.0)
Cholestasis	-	15 (0.1)	12 (0.0)	-	-	5 (0.1)	-	32 (0.1)
Cholestatic liver injury	-	-	5 (0.0)	-	-	-	-	5 (0.0)
Chorea	-	2 (0.0)	2 (0.0)	-	-	-	-	4 (0.0)
Choreoathetosis	-	-	2 (0.0)	-	-	1 (0.0)	-	3 (0.0)
Chromaturia	1 (0.0)	4 (0.0)	87 (0.3)	-	-	-	-	92 (0.2)
Chronic hepatitis	-	1 (0.0)	-	-	-	-	-	1 (0.0)
Chronic kidney disease	-	-	-	1 (0.3)	-	-	-	1 (0.0)
Chronic obstructive pulmonary disease	1 (0.0)	2 (0.0)	2 (0.0)	-	-	-	-	5 (0.0)
Chronic sinusitis	-	14 (0.1)	-	-	-	-	-	14 (0.0)
Circadian rhythm sleep disorder	-	2 (0.0)	-	1 (0.3)	-	1 (0.0)	-	4 (0.0)
Circulatory collapse	8 (0.2)	36 (0.2)	151 (0.6)	-	2 (0.4)	26 (0.6)	1 (0.4)	224 (0.5)
Circumoral oedema	-	-	3 (0.0)	-	-	-	-	3 (0.0)
Circumstance or information capable of leading to medication error	2 (0.1)	3 (0.0)	2 (0.0)	-	-	-	-	7 (0.0)
Clinical death	-	-	1 (0.0)	-	-	-	-	1 (0.0)
Clitoral engorgement	-	-	-	-	-	1 (0.0)	-	1 (0.0)
Clonic convulsion	-	4 (0.0)	11 (0.0)	-	-	2 (0.0)	-	17 (0.0)
Clonus	-	7 (0.0)	19 (0.1)	-	-	8 (0.2)	-	34 (0.1)
Clostridial infection	-	-	3 (0.0)	-	-	-	-	3 (0.0)
Clostridium colitis	-	-	1 (0.0)	-	-	-	-	1 (0.0)
Clostridium difficile colitis	-	1 (0.0)	1 (0.0)	-	-	1 (0.0)	-	3 (0.0)
Clostridium difficile infection	-	-	1 (0.0)	-	-	-	-	1 (0.0)
Clubbing	-	1 (0.0)	-	-	-	-	-	1 (0.0)
Clumsiness	-	-	1 (0.0)	-	-	-	-	1 (0.0)
CNS ventriculitis	-	1 (0.0)	-	-	-	-	-	1 (0.0)
Coagulation test abnormal	-	-	1 (0.0)	-	-	-	-	1 (0.0)
Coagulation time prolonged	-	1 (0.0)	-	-	-	2 (0.0)	-	3 (0.0)
Coagulopathy	-	5 (0.0)	16 (0.1)	-	-	11 (0.2)	-	32 (0.1)
Cognitive disorder	-	17 (0.1)	13 (0.1)	2 (0.6)	-	10 (0.2)	-	42 (0.1)
Cogwheel rigidity	-	-	4 (0.0)	-	-	1 (0.0)	-	5 (0.0)

Cold sweat	-	7 (0.0)	3 (0.0)	-	-	1 (0.0)	-	11 (0.0)
Colitis	-	27 (0.2)	-	-	-	-	-	27 (0.1)
Colitis ischaemic	1 (0.0)	2 (0.0)	1 (0.0)	-	-	1 (0.0)	-	5 (0.0)
Colitis ulcerative	-	23 (0.2)	4 (0.0)	-	-	-	-	27 (0.1)
Colonoscopy	-	-	1 (0.0)	-	-	-	-	1 (0.0)
Colorectal cancer metastatic	-	-	-	-	-	1 (0.0)	-	1 (0.0)
Colour blindness	-	-	-	-	-	-	1 (0.4)	1 (0.0)
Coma	4 (0.1)	102 (0.7)	56 (0.2)	-	-	8 (0.2)	1 (0.4)	171 (0.3)
Coma blister	-	-	3 (0.0)	-	-	2 (0.0)	-	5 (0.0)
Coma hepatic	-	1 (0.0)	2 (0.0)	-	-	-	-	3 (0.0)
Coma neonatal	-	1 (0.0)	-	-	-	-	-	1 (0.0)
Coma scale abnormal	-	10 (0.1)	2 (0.0)	-	-	-	-	12 (0.0)
Communication disorder	-	1 (0.0)	-	-	-	1 (0.0)	-	2 (0.0)
Compartment syndrome	-	2 (0.0)	10 (0.0)	-	-	-	-	12 (0.0)
Completed suicide	6 (0.2)	30 (0.2)	47 (0.2)	-	-	5 (0.1)	-	88 (0.2)
Complex regional pain syndrome	-	-	2 (0.0)	-	-	-	-	2 (0.0)
Complication associated with device	-	-	5 (0.0)	-	-	1 (0.0)	-	6 (0.0)
Complication of delivery	-	-	1 (0.0)	-	-	-	-	1 (0.0)
Complication of device insertion	-	-	1 (0.0)	-	-	-	-	1 (0.0)
Complication of device removal	-	-	1 (0.0)	-	-	-	-	1 (0.0)
Complication of pregnancy	-	1 (0.0)	-	-	-	-	-	1 (0.0)
Compulsive shopping	-	3 (0.0)	-	-	-	-	-	3 (0.0)
Concomitant disease aggravated	-	3 (0.0)	4 (0.0)	-	-	-	-	7 (0.0)
Concomitant disease progression	-	1 (0.0)	-	-	-	-	-	1 (0.0)
Concussion	1 (0.0)	-	-	-	-	-	-	1 (0.0)
Condition aggravated	9 (0.2)	51 (0.3)	77 (0.3)	1 (0.3)	2 (0.4)	22 (0.5)	2 (0.8)	164 (0.3)
Conduction disorder	-	1 (0.0)	3 (0.0)	1 (0.3)	-	-	-	5 (0.0)
Confusion postoperative	-	1 (0.0)	3 (0.0)	-	-	1 (0.0)	-	5 (0.0)
Confusional arousal	1 (0.0)	1 (0.0)	-	-	-	-	-	2 (0.0)
Confusional state	6 (0.2)	83 (0.6)	36 (0.1)	-	1 (0.2)	9 (0.2)	1 (0.4)	136 (0.3)
Congenital anomaly	-	2 (0.0)	-	-	-	-	-	2 (0.0)
Congenital brain damage	-	1 (0.0)	-	-	-	-	-	1 (0.0)

Congenital central hypoventilation syndrome	-	-	1 (0.0)	-	-	-	-	1 (0.0)
Congenital central nervous system anomaly	-	-	1 (0.0)	-	-	-	-	1 (0.0)
Congenital clavicular agenesis	-	-	-	-	-	2 (0.0)	-	2 (0.0)
Congenital infection	-	1 (0.0)	-	-	-	-	-	1 (0.0)
Congenital renal cyst	-	-	1 (0.0)	-	-	-	-	1 (0.0)
Congenital tracheomalacia	-	-	-	-	-	1 (0.0)	-	1 (0.0)
Congenital umbilical hernia	-	-	1 (0.0)	-	-	-	-	1 (0.0)
Congestive hepatopathy	-	-	13 (0.1)	-	-	-	-	13 (0.0)
Conjunctival disorder	-	1 (0.0)	1 (0.0)	-	-	-	-	2 (0.0)
Conjunctival erosion	-	1 (0.0)	-	-	-	-	-	1 (0.0)
Conjunctival haemorrhage	-	-	2 (0.0)	-	-	-	-	2 (0.0)
Conjunctival hyperaemia	-	2 (0.0)	6 (0.0)	-	-	-	-	8 (0.0)
Conjunctival oedema	-	-	2 (0.0)	-	-	2 (0.0)	-	4 (0.0)
Conjunctivitis	-	1 (0.0)	8 (0.0)	-	-	1 (0.0)	-	10 (0.0)
Consciousness fluctuating	-	2 (0.0)	2 (0.0)	-	-	-	-	4 (0.0)
Constipation	6 (0.2)	29 (0.2)	10 (0.0)	1 (0.3)	-	1 (0.0)	-	47 (0.1)
Contraindicated product administered	1 (0.0)	1 (0.0)	3 (0.0)	-	-	4 (0.1)	-	9 (0.0)
Contraindication to medical treatment	-	-	3 (0.0)	-	-	-	-	3 (0.0)
Contusion	2 (0.1)	4 (0.0)	-	-	-	1 (0.0)	-	7 (0.0)
Conversion disorder	-	2 (0.0)	3 (0.0)	-	-	2 (0.0)	-	7 (0.0)
Convulsions local	-	-	16 (0.1)	-	-	-	-	16 (0.0)
Convulsive threshold lowered	-	-	-	-	-	1 (0.0)	-	1 (0.0)
Coordination abnormal	-	6 (0.0)	2 (0.0)	-	-	1 (0.0)	-	9 (0.0)
Coprolalia	-	-	1 (0.0)	-	-	-	-	1 (0.0)
Corneal abrasion	-	-	2 (0.0)	-	-	-	-	2 (0.0)
Corneal disorder	-	-	1 (0.0)	-	-	-	-	1 (0.0)
Corneal endothelial cell loss	-	1 (0.0)	-	-	-	-	-	1 (0.0)
Corneal irritation	-	-	1 (0.0)	-	-	-	-	1 (0.0)
Corneal light reflex test abnormal	-	-	-	-	-	1 (0.0)	-	1 (0.0)
Corneal oedema	-	1 (0.0)	-	-	-	-	-	1 (0.0)
Corneal reflex decreased	-	4 (0.0)	-	-	-	-	-	4 (0.0)
Corneal warpage	-	-	1 (0.0)	-	-	-	-	1 (0.0)

Coronary artery bypass	-	-	-	-	-	1 (0.0)	-	1 (0.0)
Coronary artery compression	-	1 (0.0)	-	-	-	-	-	1 (0.0)
Coronary artery disease	-	1 (0.0)	-	-	-	3 (0.1)	-	4 (0.0)
Coronary artery embolism	-	-	-	-	-	1 (0.0)	-	1 (0.0)
Coronary artery occlusion	-	-	2 (0.0)	-	-	-	-	2 (0.0)
Coronary artery stenosis	1 (0.0)	2 (0.0)	1 (0.0)	-	-	-	-	4 (0.0)
Coronary artery thrombosis	-	-	2 (0.0)	-	-	-	-	2 (0.0)
Cortisol abnormal	-	-	-	-	-	1 (0.0)	-	1 (0.0)
Cough	5 (0.1)	28 (0.2)	97 (0.4)	1 (0.3)	-	3 (0.1)	-	134 (0.3)
COVID-19	1 (0.0)	8 (0.1)	2 (0.0)	1 (0.3)	-	1 (0.0)	-	13 (0.0)
COVID-19 pneumonia	1 (0.0)	2 (0.0)	3 (0.0)	-	-	-	-	6 (0.0)
Cranial nerve disorder	1 (0.0)	-	1 (0.0)	-	-	1 (0.0)	-	3 (0.0)
Cranial nerve paralysis	-	-	-	-	-	1 (0.0)	-	1 (0.0)
Cranial operation	2 (0.1)	-	-	-	-	-	-	2 (0.0)
Craniocerebral injury	-	-	-	-	-	1 (0.0)	-	1 (0.0)
Craniofacial fracture	1 (0.0)	-	-	-	-	-	-	1 (0.0)
C-reactive protein abnormal	-	1 (0.0)	-	-	-	-	-	1 (0.0)
C-reactive protein decreased	-	1 (0.0)	-	-	-	-	-	1 (0.0)
C-reactive protein increased	2 (0.1)	8 (0.1)	10 (0.0)	-	-	5 (0.1)	-	25 (0.1)
Creatinine renal clearance decreased	-	-	8 (0.0)	-	-	1 (0.0)	-	9 (0.0)
Creatinine renal clearance increased	-	-	1 (0.0)	-	-	-	-	1 (0.0)
Crepitations	-	-	1 (0.0)	-	-	2 (0.0)	-	3 (0.0)
Critical illness	-	1 (0.0)	-	-	-	-	-	1 (0.0)
Crohn's disease	-	9 (0.1)	-	-	-	-	-	9 (0.0)
Cross sensitivity reaction	-	1 (0.0)	1 (0.0)	-	-	1 (0.0)	-	3 (0.0)
Croup infectious	-	-	1 (0.0)	-	-	-	-	1 (0.0)
Crying	-	12 (0.1)	6 (0.0)	1 (0.3)	-	-	-	19 (0.0)
Cryptococcosis	-	1 (0.0)	-	-	-	-	-	1 (0.0)
Cryptorchism	-	1 (0.0)	-	-	-	-	-	1 (0.0)
Crystal urine present	-	-	3 (0.0)	-	-	-	-	3 (0.0)
CSF oligoclonal band present	-	-	-	-	-	2 (0.0)	-	2 (0.0)
CSF pressure increased	-	1 (0.0)	-	-	-	-	1 (0.4)	2 (0.0)

Cutaneous symptom	-	-	1 (0.0)	-	-	-	-	1 (0.0)
Cutaneous vasculitis	1 (0.0)	-	-	-	-	-	-	1 (0.0)
Cyanosis	-	22 (0.1)	44 (0.2)	-	-	13 (0.3)	-	79 (0.2)
Cyanosis central	-	-	1 (0.0)	-	-	-	-	1 (0.0)
Cyanosis neonatal	-	1 (0.0)	1 (0.0)	-	-	1 (0.0)	-	3 (0.0)
CYP2C19 polymorphism	-	1 (0.0)	-	-	-	-	-	1 (0.0)
Cyst	-	-	1 (0.0)	-	-	-	-	1 (0.0)
Cystic fibrosis	-	2 (0.0)	-	-	-	-	-	2 (0.0)
Cystitis	-	1 (0.0)	-	-	-	-	-	1 (0.0)
Cystitis interstitial	-	-	-	-	-	1 (0.0)	-	1 (0.0)
Cytokine release syndrome	-	5 (0.0)	1 (0.0)	-	-	-	-	6 (0.0)
Cytokine storm	-	1 (0.0)	2 (0.0)	-	-	1 (0.0)	-	4 (0.0)
Cytomegalovirus infection	-	1 (0.0)	2 (0.0)	-	-	-	-	3 (0.0)
Cytotoxic oedema	-	2 (0.0)	1 (0.0)	-	-	-	1 (0.4)	4 (0.0)
Dacryostenosis acquired	-	1 (0.0)	-	-	-	-	-	1 (0.0)
Daydreaming	-	-	1 (0.0)	-	-	-	-	1 (0.0)
Deafness	1 (0.0)	-	-	-	-	-	-	1 (0.0)
Deafness bilateral	1 (0.0)	1 (0.0)	-	-	-	-	-	2 (0.0)
Deafness neurosensory	-	-	1 (0.0)	-	-	-	-	1 (0.0)
Deafness unilateral	1 (0.0)	-	1 (0.0)	-	-	1 (0.0)	-	3 (0.0)
Death	23 (0.6)	140 (0.9)	47 (0.2)	-	-	19 (0.4)	-	229 (0.5)
Death neonatal	-	2 (0.0)	1 (0.0)	-	1 (0.2)	-	-	4 (0.0)
Decerebrate posture	-	1 (0.0)	1 (0.0)	-	-	-	-	2 (0.0)
Decerebration	2 (0.1)	-	2 (0.0)	-	-	-	-	4 (0.0)
Decompensated hypothyroidism	-	-	5 (0.0)	-	-	-	-	5 (0.0)
Decorticate posture	-	-	1 (0.0)	-	-	-	-	1 (0.0)
Decreased activity	-	1 (0.0)	3 (0.0)	-	-	-	-	4 (0.0)
Decreased appetite	-	20 (0.1)	14 (0.1)	-	-	2 (0.0)	-	36 (0.1)
Decreased eye contact	-	1 (0.0)	-	1 (0.3)	-	-	-	2 (0.0)
Decreased interest	-	1 (0.0)	4 (0.0)	-	-	-	-	5 (0.0)
Decreased ventricular afterload	-	1 (0.0)	-	-	-	-	-	1 (0.0)
Decubitus ulcer	-	2 (0.0)	1 (0.0)	-	-	1 (0.0)	-	4 (0.0)

Deep vein thrombosis	1 (0.0)	9 (0.1)	3 (0.0)	-	-	7 (0.1)	-	20 (0.0)
Defaecation disorder	-	1 (0.0)	-	-	-	-	-	1 (0.0)
Defaecation urgency	-	1 (0.0)	-	-	-	-	-	1 (0.0)
Defect conduction intraventricular	-	1 (0.0)	-	-	-	-	-	1 (0.0)
Deficiency of bile secretion	-	-	1 (0.0)	-	-	-	-	1 (0.0)
Deformity	-	1 (0.0)	-	-	-	-	-	1 (0.0)
Dehydration	1 (0.0)	24 (0.2)	7 (0.0)	-	5 (1.0)	6 (0.1)	-	43 (0.1)
Delayed graft function	-	1 (0.0)	-	-	-	-	-	1 (0.0)
Delayed recovery from anaesthesia	6 (0.2)	43 (0.3)	73 (0.3)	3 (0.9)	4 (0.8)	10 (0.2)	1 (0.4)	140 (0.3)
Delirium	25 (0.7)	93 (0.6)	37 (0.1)	8 (2.4)	5 (1.0)	13 (0.3)	7 (2.6)	188 (0.4)
Delirium tremens	-	1 (0.0)	1 (0.0)	-	-	-	-	2 (0.0)
Delusion	1 (0.0)	5 (0.0)	20 (0.1)	-	-	1 (0.0)	-	27 (0.1)
Delusion of grandeur	-	1 (0.0)	-	-	-	-	-	1 (0.0)
Dementia	-	-	-	-	-	1 (0.0)	-	1 (0.0)
Dental caries	-	-	1 (0.0)	-	-	-	-	1 (0.0)
Dental plaque	-	1 (0.0)	-	-	-	-	-	1 (0.0)
Dependence	-	5 (0.0)	5 (0.0)	-	-	-	-	10 (0.0)
Dependence on respirator	-	1 (0.0)	-	-	-	-	-	1 (0.0)
Depersonalisation/derealisation disorder	-	3 (0.0)	-	-	-	-	-	3 (0.0)
Depressed level of consciousness	7 (0.2)	125 (0.8)	67 (0.3)	1 (0.3)	1 (0.2)	17 (0.4)	1 (0.4)	219 (0.4)
Depressed mood	-	5 (0.0)	4 (0.0)	-	-	1 (0.0)	-	10 (0.0)
Depression	-	13 (0.1)	8 (0.0)	-	-	4 (0.1)	-	25 (0.1)
Derealisation	-	1 (0.0)	-	-	-	-	-	1 (0.0)
Dermatitis	-	-	2 (0.0)	-	-	-	-	2 (0.0)
Dermatitis allergic	1 (0.0)	7 (0.0)	11 (0.0)	-	-	6 (0.1)	-	25 (0.1)
Dermatitis bullous	-	2 (0.0)	7 (0.0)	-	-	2 (0.0)	-	11 (0.0)
Dermatitis contact	-	1 (0.0)	-	-	-	-	-	1 (0.0)
Dermatitis exfoliative generalised	-	1 (0.0)	7 (0.0)	-	1 (0.2)	1 (0.0)	-	10 (0.0)
Dermatitis herpetiformis	-	1 (0.0)	-	-	-	-	-	1 (0.0)
Developmental delay	-	1 (0.0)	2 (0.0)	-	-	-	-	3 (0.0)
Developmental hip dysplasia	-	1 (0.0)	-	-	-	-	-	1 (0.0)
Developmental regression	-	1 (0.0)	-	-	-	-	-	1 (0.0)

Device connection issue	-	1 (0.0)	2 (0.0)	-	-	-	-	3 (0.0)
Device delivery system issue	-	-	1 (0.0)	-	-	-	-	1 (0.0)
Device dislocation	-	-	2 (0.0)	-	-	-	-	2 (0.0)
Device dispensing error	-	-	1 (0.0)	-	-	-	-	1 (0.0)
Device electrical finding	-	-	1 (0.0)	-	-	-	-	1 (0.0)
Device extrusion	-	-	-	-	-	1 (0.0)	-	1 (0.0)
Device failure	-	-	3 (0.0)	-	-	-	-	3 (0.0)
Device infusion issue	-	2 (0.0)	7 (0.0)	-	-	-	-	9 (0.0)
Device issue	-	-	9 (0.0)	-	-	-	-	9 (0.0)
Device kink	-	-	-	1 (0.3)	-	-	-	1 (0.0)
Device leakage	-	-	1 (0.0)	-	-	-	-	1 (0.0)
Device malfunction	-	2 (0.0)	5 (0.0)	-	-	-	-	7 (0.0)
Device occlusion	-	1 (0.0)	-	-	-	-	-	1 (0.0)
Device placement issue	-	-	1 (0.0)	-	-	-	-	1 (0.0)
Device programming error	-	12 (0.1)	6 (0.0)	-	-	-	-	18 (0.0)
Device related infection	1 (0.0)	-	1 (0.0)	-	-	-	-	2 (0.0)
Device related sepsis	-	2 (0.0)	-	-	-	-	-	2 (0.0)
Diabetes insipidus	27 (0.7)	-	23 (0.1)	1 (0.3)	5 (1.0)	3 (0.1)	-	59 (0.1)
Diabetes mellitus	-	2 (0.0)	-	-	-	1 (0.0)	-	3 (0.0)
Diabetic ketoacidosis	-	2 (0.0)	2 (0.0)	-	-	-	-	4 (0.0)
Diabetic macroangiopathy	-	1 (0.0)	-	-	-	-	-	1 (0.0)
Diabetic neuropathy	-	-	1 (0.0)	-	-	-	-	1 (0.0)
Dialysis	-	-	2 (0.0)	-	-	-	-	2 (0.0)
Diaphragmatic paralysis	1 (0.0)	1 (0.0)	1 (0.0)	-	-	-	-	3 (0.0)
Diaphragmatic spasm	-	-	3 (0.0)	-	-	-	-	3 (0.0)
Diarrhoea	3 (0.1)	42 (0.3)	35 (0.1)	1 (0.3)	-	6 (0.1)	1 (0.4)	88 (0.2)
Diastolic dysfunction	-	-	2 (0.0)	-	-	1 (0.0)	-	3 (0.0)
Diastolic hypotension	-	1 (0.0)	-	-	-	-	-	1 (0.0)
Diencephalic syndrome of infancy	-	-	1 (0.0)	-	-	-	-	1 (0.0)
Differentiation syndrome	1 (0.0)	-	1 (0.0)	-	-	-	-	2 (0.0)
Diffuse alveolar damage	-	1 (0.0)	-	-	-	-	-	1 (0.0)
Diffuse axonal injury	-	-	-	-	-	1 (0.0)	-	1 (0.0)

Diffuse large B-cell lymphoma refractory	-	5 (0.0)	-	-	-	-	-	5 (0.0)
Dilatation ventricular	-	-	1 (0.0)	-	-	-	-	1 (0.0)
Dilated cardiomyopathy	-	1 (0.0)	-	-	-	-	-	1 (0.0)
Diplegia	-	-	2 (0.0)	-	-	-	-	2 (0.0)
Diplopia	-	3 (0.0)	2 (0.0)	-	-	2 (0.0)	-	7 (0.0)
Disability	-	-	1 (0.0)	-	-	-	-	1 (0.0)
Discomfort	1 (0.0)	5 (0.0)	4 (0.0)	-	-	-	-	10 (0.0)
Disease complication	-	1 (0.0)	-	-	-	-	-	1 (0.0)
Disease progression	-	14 (0.1)	2 (0.0)	-	-	3 (0.1)	-	19 (0.0)
Disease recurrence	-	7 (0.0)	1 (0.0)	-	-	2 (0.0)	-	10 (0.0)
Disinhibition	-	6 (0.0)	-	-	-	-	-	6 (0.0)
Disorganised speech	-	-	-	-	-	1 (0.0)	-	1 (0.0)
Disorientation	-	33 (0.2)	17 (0.1)	1 (0.3)	1 (0.2)	5 (0.1)	-	57 (0.1)
Disseminated intravascular coagulation	1 (0.0)	13 (0.1)	33 (0.1)	-	1 (0.2)	7 (0.1)	-	55 (0.1)
Disseminated mycobacterium avium complex infection	-	1 (0.0)	-	-	-	-	-	1 (0.0)
Dissociation	-	3 (0.0)	-	-	-	-	-	3 (0.0)
Dissociative amnesia	-	1 (0.0)	4 (0.0)	-	-	1 (0.0)	-	6 (0.0)
Dissociative disorder	-	4 (0.0)	2 (0.0)	-	-	-	-	6 (0.0)
Distractibility	-	1 (0.0)	-	-	-	-	-	1 (0.0)
Distributive shock	-	2 (0.0)	2 (0.0)	-	-	-	-	4 (0.0)
Disturbance in attention	-	27 (0.2)	10 (0.0)	-	-	1 (0.0)	1 (0.4)	39 (0.1)
Diverticulitis	-	1 (0.0)	1 (0.0)	-	-	2 (0.0)	-	4 (0.0)
Dizziness	3 (0.1)	72 (0.5)	50 (0.2)	-	-	3 (0.1)	-	128 (0.3)
Dizziness postural	-	1 (0.0)	1 (0.0)	-	-	1 (0.0)	-	3 (0.0)
Documented hypersensitivity to administered product	-	4 (0.0)	-	-	-	-	-	4 (0.0)
Dose calculation error	1 (0.0)	1 (0.0)	-	-	-	-	-	2 (0.0)
Double inlet left ventricle	-	-	-	-	1 (0.2)	-	-	1 (0.0)
Double outlet right ventricle	-	-	-	-	1 (0.2)	-	-	1 (0.0)
Drooling	1 (0.0)	1 (0.0)	2 (0.0)	-	-	-	-	4 (0.0)
Drop attacks	-	-	1 (0.0)	-	-	-	-	1 (0.0)
Drowning	-	-	2 (0.0)	-	-	-	-	2 (0.0)
Drug abuse	1 (0.0)	87 (0.6)	54 (0.2)	-	-	6 (0.1)	-	148 (0.3)

Drug abuser	-	2 (0.0)	-	-	-	-	-	2 (0.0)
Drug chemical incompatibility	-	4 (0.0)	2 (0.0)	-	-	-	-	6 (0.0)
Drug clearance decreased	1 (0.0)	2 (0.0)	-	-	-	1 (0.0)	-	4 (0.0)
Drug dependence	1 (0.0)	56 (0.4)	19 (0.1)	-	-	1 (0.0)	-	77 (0.2)
Drug detoxification	-	2 (0.0)	-	-	-	-	-	2 (0.0)
Drug dispensed to wrong patient	-	2 (0.0)	-	-	-	-	-	2 (0.0)
Drug diversion	-	5 (0.0)	-	-	-	-	-	5 (0.0)
Drug dose omission by device	-	-	1 (0.0)	-	-	-	-	1 (0.0)
Drug dose titration not performed	1 (0.0)	-	-	-	-	-	-	1 (0.0)
Drug effect less than expected	-	1 (0.0)	8 (0.0)	-	-	1 (0.0)	-	10 (0.0)
Drug effective for unapproved indication	-	1 (0.0)	2 (0.0)	-	-	-	-	3 (0.0)
Drug eruption	1 (0.0)	11 (0.1)	8 (0.0)	-	-	5 (0.1)	1 (0.4)	26 (0.1)
Drug half-life increased	-	-	-	1 (0.3)	-	-	-	1 (0.0)
Drug hypersensitivity	4 (0.1)	29 (0.2)	57 (0.2)	-	-	13 (0.3)	2 (0.8)	105 (0.2)
Drug ineffective	28 (0.7)	425 (2.8)	467 (1.9)	9 (2.7)	4 (0.8)	111 (2.4)	2 (0.8)	1046 (2.1)
Drug ineffective for unapproved indication	8 (0.2)	30 (0.2)	16 (0.1)	1 (0.3)	2 (0.4)	11 (0.2)	1 (0.4)	69 (0.1)
Drug interaction	43 (1.1)	164 (1.1)	151 (0.6)	8 (2.4)	9 (1.8)	46 (1.0)	1 (0.4)	422 (0.9)
Drug intolerance	-	8 (0.1)	2 (0.0)	-	-	-	-	10 (0.0)
Drug level above therapeutic	-	2 (0.0)	-	-	-	1 (0.0)	-	3 (0.0)
Drug level below therapeutic	-	3 (0.0)	-	-	-	1 (0.0)	-	4 (0.0)
Drug level decreased	-	2 (0.0)	1 (0.0)	-	-	-	-	3 (0.0)
Drug level increased	2 (0.1)	17 (0.1)	9 (0.0)	1 (0.3)	-	1 (0.0)	-	30 (0.1)
Drug monitoring procedure incorrectly performed	-	-	-	-	-	1 (0.0)	-	1 (0.0)
Drug monitoring procedure not performed	-	1 (0.0)	1 (0.0)	-	-	-	-	2 (0.0)
Drug reaction with eosinophilia and systemic symptoms	-	12 (0.1)	14 (0.1)	1 (0.3)	-	7 (0.1)	2 (0.8)	36 (0.1)
Drug resistance	3 (0.1)	27 (0.2)	3 (0.0)	-	-	4 (0.1)	-	37 (0.1)
Drug screen positive	-	2 (0.0)	-	-	-	-	-	2 (0.0)
Drug specific antibody present	-	4 (0.0)	-	-	-	-	-	4 (0.0)
Drug therapy	-	1 (0.0)	-	-	-	-	-	1 (0.0)
Drug tolerance	1 (0.0)	4 (0.0)	1 (0.0)	-	-	2 (0.0)	-	8 (0.0)
Drug use disorder	-	2 (0.0)	-	-	-	-	-	2 (0.0)
Drug withdrawal convulsions	8 (0.2)	3 (0.0)	1 (0.0)	-	-	2 (0.0)	-	14 (0.0)

Drug withdrawal syndrome	17 (0.4)	63 (0.4)	10 (0.0)	2 (0.6)	1 (0.2)	2 (0.0)	2 (0.8)	97 (0.2)
Drug withdrawal syndrome neonatal	1 (0.0)	9 (0.1)	-	2 (0.6)	-	1 (0.0)	-	13 (0.0)
Drug-disease interaction	-	-	1 (0.0)	-	-	-	-	1 (0.0)
Drug-induced liver injury	-	9 (0.1)	36 (0.1)	1 (0.3)	1 (0.2)	2 (0.0)	-	49 (0.1)
Dry eye	-	5 (0.0)	-	-	-	-	-	5 (0.0)
Dry gangrene	-	1 (0.0)	-	-	-	1 (0.0)	-	2 (0.0)
Dry mouth	2 (0.1)	15 (0.1)	3 (0.0)	-	-	-	-	20 (0.0)
Dry skin	-	3 (0.0)	-	-	1 (0.2)	-	-	4 (0.0)
Duchenne muscular dystrophy	-	-	1 (0.0)	-	-	-	-	1 (0.0)
Duodenal ulcer	-	1 (0.0)	-	-	-	2 (0.0)	-	3 (0.0)
Dural abscess	-	1 (0.0)	-	-	-	-	-	1 (0.0)
Dysaesthesia	-	-	2 (0.0)	-	-	-	-	2 (0.0)
Dysarthria	4 (0.1)	11 (0.1)	7 (0.0)	-	-	3 (0.1)	-	25 (0.1)
Dyschezia	-	4 (0.0)	-	-	-	-	-	4 (0.0)
Dysdiadochokinesis	-	1 (0.0)	-	-	-	-	-	1 (0.0)
Dysgeusia	-	-	4 (0.0)	-	-	-	-	4 (0.0)
Dysgraphia	-	2 (0.0)	-	-	-	-	-	2 (0.0)
Dyskinesia	1 (0.0)	45 (0.3)	56 (0.2)	1 (0.3)	1 (0.2)	11 (0.2)	7 (2.6)	122 (0.2)
Dyslalia	1 (0.0)	-	-	-	-	-	-	1 (0.0)
Dyslipidaemia	-	1 (0.0)	12 (0.0)	-	-	-	-	13 (0.0)
Dysmorphism	-	-	-	-	-	2 (0.0)	-	2 (0.0)
Dyspepsia	-	16 (0.1)	2 (0.0)	-	-	1 (0.0)	-	19 (0.0)
Dysphagia	2 (0.1)	10 (0.1)	17 (0.1)	1 (0.3)	-	3 (0.1)	-	33 (0.1)
Dysphemia	-	2 (0.0)	1 (0.0)	-	-	-	-	3 (0.0)
Dysphonia	2 (0.1)	5 (0.0)	8 (0.0)	-	-	1 (0.0)	-	16 (0.0)
Dysphoria	-	2 (0.0)	9 (0.0)	-	-	-	1 (0.4)	12 (0.0)
Dyspnoea	11 (0.3)	110 (0.7)	136 (0.5)	1 (0.3)	-	26 (0.6)	1 (0.4)	285 (0.6)
Dyspnoea exertional	-	2 (0.0)	2 (0.0)	-	-	-	-	4 (0.0)
Dyspnoea paroxysmal nocturnal	-	1 (0.0)	1 (0.0)	-	-	-	-	2 (0.0)
Dyspraxia	-	2 (0.0)	-	-	-	-	-	2 (0.0)
Dysstasia	1 (0.0)	2 (0.0)	1 (0.0)	-	-	-	-	4 (0.0)
Dystonia	-	14 (0.1)	35 (0.1)	-	-	7 (0.1)	-	56 (0.1)

Dystonic tremor	-	1 (0.0)	-	-	-	-	-	1 (0.0)
Dysuria	-	4 (0.0)	5 (0.0)	-	-	1 (0.0)	-	10 (0.0)
Ear disorder	-	1 (0.0)	-	-	-	-	-	1 (0.0)
Ear haemorrhage	-	-	1 (0.0)	-	-	-	-	1 (0.0)
Ear infection	-	1 (0.0)	-	-	-	-	-	1 (0.0)
Ear malformation	-	1 (0.0)	-	-	-	-	-	1 (0.0)
Ear swelling	-	1 (0.0)	-	-	-	1 (0.0)	-	2 (0.0)
Eating disorder	-	5 (0.0)	2 (0.0)	-	-	-	-	7 (0.0)
Ecchymosis	-	1 (0.0)	1 (0.0)	-	-	-	-	2 (0.0)
ECG signs of myocardial ischaemia	-	-	1 (0.0)	-	-	-	-	1 (0.0)
Echocardiogram abnormal	1 (0.0)	-	-	-	-	-	-	1 (0.0)
Echolalia	-	2 (0.0)	-	-	-	-	-	2 (0.0)
Eclampsia	-	1 (0.0)	-	-	-	-	-	1 (0.0)
Economic problem	-	-	1 (0.0)	-	-	-	-	1 (0.0)
Eczema	-	3 (0.0)	2 (0.0)	-	-	-	-	5 (0.0)
Eczema asteatotic	-	1 (0.0)	-	-	-	-	-	1 (0.0)
Eczema eyelids	-	2 (0.0)	-	-	-	-	-	2 (0.0)
Ejection fraction abnormal	-	1 (0.0)	1 (0.0)	-	-	-	-	2 (0.0)
Ejection fraction decreased	1 (0.0)	3 (0.0)	12 (0.0)	-	-	3 (0.1)	-	19 (0.0)
Electric shock	-	-	1 (0.0)	-	-	-	-	1 (0.0)
Electric shock sensation	-	-	1 (0.0)	-	-	-	-	1 (0.0)
Electrocardiogram	-	-	1 (0.0)	-	-	-	-	1 (0.0)
Electrocardiogram abnormal	-	12 (0.1)	23 (0.1)	-	-	5 (0.1)	-	40 (0.1)
Electrocardiogram change	-	-	2 (0.0)	-	-	-	-	2 (0.0)
Electrocardiogram P wave abnormal	-	2 (0.0)	1 (0.0)	-	-	1 (0.0)	1 (0.4)	5 (0.0)
Electrocardiogram PR prolongation	2 (0.1)	-	3 (0.0)	-	-	-	-	5 (0.0)
Electrocardiogram QRS complex	-	-	1 (0.0)	-	-	-	-	1 (0.0)
Electrocardiogram QRS complex abnormal	-	-	-	-	-	1 (0.0)	-	1 (0.0)
Electrocardiogram QRS complex prolonged	1 (0.0)	5 (0.0)	9 (0.0)	-	-	4 (0.1)	-	19 (0.0)
Electrocardiogram QRS complex shortened	-	-	1 (0.0)	-	-	1 (0.0)	-	2 (0.0)
Electrocardiogram QT prolonged	13 (0.3)	21 (0.1)	35 (0.1)	3 (0.9)	2 (0.4)	10 (0.2)	-	84 (0.2)
Electrocardiogram R on T phenomenon	-	-	2 (0.0)	-	-	-	-	2 (0.0)

Electrocardiogram repolarisation abnormality	-	-	1 (0.0)	1 (0.3)	-	1 (0.0)	-	3 (0.0)
Electrocardiogram ST segment	-	-	1 (0.0)	-	-	-	-	1 (0.0)
Electrocardiogram ST segment abnormal	1 (0.0)	-	5 (0.0)	-	-	-	-	6 (0.0)
Electrocardiogram ST segment depression	-	5 (0.0)	18 (0.1)	-	-	1 (0.0)	1 (0.4)	25 (0.1)
Electrocardiogram ST segment elevation	7 (0.2)	2 (0.0)	48 (0.2)	-	1 (0.2)	7 (0.1)	-	65 (0.1)
Electrocardiogram ST-T change	-	1 (0.0)	1 (0.0)	-	-	1 (0.0)	-	3 (0.0)
Electrocardiogram T wave abnormal	-	1 (0.0)	-	-	-	1 (0.0)	-	2 (0.0)
Electrocardiogram T wave alternans	-	1 (0.0)	-	-	-	-	-	1 (0.0)
Electrocardiogram T wave amplitude decreased	-	-	-	-	-	-	1 (0.4)	1 (0.0)
Electrocardiogram T wave biphasic	1 (0.0)	1 (0.0)	1 (0.0)	-	-	-	-	3 (0.0)
Electrocardiogram T wave inversion	1 (0.0)	1 (0.0)	7 (0.0)	-	-	1 (0.0)	1 (0.4)	11 (0.0)
Electrocardiogram T wave peaked	-	1 (0.0)	1 (0.0)	-	-	2 (0.0)	-	4 (0.0)
Electrocardiogram U wave present	-	-	-	-	-	-	1 (0.4)	1 (0.0)
Electrocution	-	1 (0.0)	-	-	-	-	-	1 (0.0)
Electroencephalogram abnormal	-	19 (0.1)	10 (0.0)	-	-	4 (0.1)	-	33 (0.1)
Electrolyte imbalance	2 (0.1)	8 (0.1)	7 (0.0)	-	1 (0.2)	1 (0.0)	1 (0.4)	20 (0.0)
Electromyogram abnormal	-	-	2 (0.0)	-	-	-	-	2 (0.0)
Embolic stroke	1 (0.0)	-	2 (0.0)	-	-	1 (0.0)	-	4 (0.0)
Embolism	-	3 (0.0)	1 (0.0)	-	-	1 (0.0)	1 (0.4)	6 (0.0)
Embolism arterial	-	-	1 (0.0)	-	-	-	1 (0.4)	2 (0.0)
Embolism venous	3 (0.1)	-	2 (0.0)	-	-	-	-	5 (0.0)
Emotional disorder	-	4 (0.0)	-	-	-	-	-	4 (0.0)
Emotional distress	-	8 (0.1)	1 (0.0)	-	-	1 (0.0)	-	10 (0.0)
Emphysema	-	1 (0.0)	1 (0.0)	-	-	2 (0.0)	-	4 (0.0)
Empyema	-	2 (0.0)	-	-	-	-	-	2 (0.0)
Encephalitis	-	2 (0.0)	1 (0.0)	-	-	2 (0.0)	-	5 (0.0)
Encephalitis autoimmune	-	1 (0.0)	3 (0.0)	-	-	2 (0.0)	-	6 (0.0)
Encephalitis viral	-	1 (0.0)	1 (0.0)	-	-	-	-	2 (0.0)
Encephalomalacia	-	1 (0.0)	1 (0.0)	-	-	-	-	2 (0.0)
Encephalopathy	4 (0.1)	31 (0.2)	26 (0.1)	-	1 (0.2)	4 (0.1)	1 (0.4)	67 (0.1)
Encephalopathy neonatal	-	2 (0.0)	-	-	-	-	-	2 (0.0)
Endocarditis	-	-	-	-	-	1 (0.0)	-	1 (0.0)

Endometrial cancer	-	-	2 (0.0)	-	-	-	-	2 (0.0)
Endoscopic retrograde cholangiopancreatography	-	-	1 (0.0)	-	-	-	-	1 (0.0)
Endotoxic shock	-	-	5 (0.0)	-	-	-	-	5 (0.0)
Endotracheal intubation	1 (0.0)	6 (0.0)	9 (0.0)	-	-	2 (0.0)	-	18 (0.0)
Endotracheal intubation complication	1 (0.0)	1 (0.0)	11 (0.0)	-	-	3 (0.1)	-	16 (0.0)
End-tidal CO2 abnormal	-	-	-	-	-	1 (0.0)	-	1 (0.0)
End-tidal CO2 decreased	-	-	6 (0.0)	-	-	1 (0.0)	-	7 (0.0)
End-tidal CO2 increased	-	-	5 (0.0)	-	-	1 (0.0)	-	6 (0.0)
Enlarged cerebral perivascular spaces	-	1 (0.0)	-	-	-	-	-	1 (0.0)
Enlarged uvula	-	-	1 (0.0)	-	-	1 (0.0)	-	2 (0.0)
Enterobacter infection	-	1 (0.0)	1 (0.0)	-	-	-	-	2 (0.0)
Enterococcal infection	-	-	4 (0.0)	-	-	-	-	4 (0.0)
Enterocolitis	-	-	1 (0.0)	-	-	-	-	1 (0.0)
Enzyme level abnormal	-	-	1 (0.0)	-	-	-	-	1 (0.0)
Enzyme level decreased	-	-	1 (0.0)	-	-	-	-	1 (0.0)
Eosinophil count increased	-	1 (0.0)	-	-	-	-	-	1 (0.0)
Eosinophil percentage decreased	1 (0.0)	-	-	-	-	-	-	1 (0.0)
Eosinophilia	-	14 (0.1)	14 (0.1)	-	-	9 (0.2)	-	37 (0.1)
Eosinophilic pneumonia	1 (0.0)	-	-	-	-	-	-	1 (0.0)
Eosinophilic pneumonia acute	-	-	1 (0.0)	-	-	1 (0.0)	-	2 (0.0)
Epidermal necrosis	-	1 (0.0)	-	-	-	-	-	1 (0.0)
Epidermolysis bullosa	-	-	1 (0.0)	-	-	1 (0.0)	-	2 (0.0)
Epigastric discomfort	-	-	-	-	-	1 (0.0)	-	1 (0.0)
Epiglottic oedema	-	-	1 (0.0)	-	-	-	-	1 (0.0)
Epilepsy	2 (0.1)	41 (0.3)	34 (0.1)	-	-	2 (0.0)	1 (0.4)	80 (0.2)
Epilepsy with myoclonic-atonic seizures	-	-	-	-	-	1 (0.0)	-	1 (0.0)
Epileptic aura	-	1 (0.0)	-	-	-	-	-	1 (0.0)
Epistaxis	1 (0.0)	6 (0.0)	5 (0.0)	-	-	3 (0.1)	-	15 (0.0)
Erectile dysfunction	-	1 (0.0)	2 (0.0)	-	-	-	-	3 (0.0)
Erection increased	-	-	-	-	-	1 (0.0)	-	1 (0.0)
Erosive duodenitis	-	-	1 (0.0)	-	-	-	-	1 (0.0)
Eructation	-	-	2 (0.0)	-	-	-	-	2 (0.0)

Erythema	5 (0.1)	86 (0.6)	340 (1.4)	-	2 (0.4)	78 (1.7)	-	511 (1.0)
Erythema multiforme	-	4 (0.0)	3 (0.0)	-	-	-	-	7 (0.0)
Erythema nodosum	-	1 (0.0)	-	-	-	-	-	1 (0.0)
Erythema of eyelid	-	1 (0.0)	1 (0.0)	-	-	-	-	2 (0.0)
Erythrosis	-	-	2 (0.0)	-	-	-	-	2 (0.0)
Escherichia bacteraemia	-	1 (0.0)	-	-	-	-	-	1 (0.0)
Escherichia infection	-	1 (0.0)	-	-	-	-	-	1 (0.0)
Escherichia sepsis	-	1 (0.0)	1 (0.0)	-	-	-	-	2 (0.0)
Escherichia urinary tract infection	-	1 (0.0)	-	-	-	1 (0.0)	-	2 (0.0)
Euglycaemic diabetic ketoacidosis	-	-	2 (0.0)	-	-	-	-	2 (0.0)
Euphoric mood	-	9 (0.1)	9 (0.0)	-	-	-	-	18 (0.0)
Euthanasia	-	2 (0.0)	2 (0.0)	-	-	-	-	4 (0.0)
Euthyroid sick syndrome	-	-	-	-	-	1 (0.0)	-	1 (0.0)
Evacuation of retained products of conception	-	-	1 (0.0)	-	-	-	-	1 (0.0)
Exaggerated startle response	1 (0.0)	-	-	-	-	-	-	1 (0.0)
Exanthema subitum	-	1 (0.0)	-	-	-	-	-	1 (0.0)
Excessive dynamic airway collapse	-	-	3 (0.0)	-	-	-	-	3 (0.0)
Exfoliative rash	-	-	1 (0.0)	-	-	-	-	1 (0.0)
Exophthalmos	-	1 (0.0)	2 (0.0)	-	-	-	-	3 (0.0)
Exostosis	-	1 (0.0)	-	-	-	-	-	1 (0.0)
Expiratory reserve volume decreased	-	-	1 (0.0)	-	-	-	-	1 (0.0)
Expired device used	-	-	1 (0.0)	-	-	-	-	1 (0.0)
Expired product administered	1 (0.0)	2 (0.0)	-	-	-	5 (0.1)	-	8 (0.0)
Exposure during pregnancy	5 (0.1)	24 (0.2)	72 (0.3)	-	2 (0.4)	21 (0.4)	2 (0.8)	126 (0.3)
Exposure keratitis	-	-	1 (0.0)	-	-	-	-	1 (0.0)
Exposure via breast milk	-	1 (0.0)	2 (0.0)	-	-	-	-	3 (0.0)
Extensor plantar response	-	3 (0.0)	4 (0.0)	-	-	-	-	7 (0.0)
External vagal nerve stimulation	-	-	1 (0.0)	-	-	-	-	1 (0.0)
Extra dose administered	-	7 (0.0)	13 (0.1)	-	-	-	-	20 (0.0)
Extradural haematoma	-	-	2 (0.0)	-	-	-	-	2 (0.0)
Extrapyramidal disorder	1 (0.0)	2 (0.0)	7 (0.0)	-	1 (0.2)	5 (0.1)	1 (0.4)	17 (0.0)
Extrasystoles	-	5 (0.0)	19 (0.1)	-	-	3 (0.1)	-	27 (0.1)

Extravasation	3 (0.1)	4 (0.0)	51 (0.2)	-	-	1 (0.0)	-	59 (0.1)
Extremity necrosis	-	2 (0.0)	-	-	-	-	-	2 (0.0)
Extubation	-	-	1 (0.0)	1 (0.3)	1 (0.2)	-	-	3 (0.0)
Eye colour change	-	-	1 (0.0)	-	-	-	-	1 (0.0)
Eye disorder	-	-	4 (0.0)	-	-	-	-	4 (0.0)
Eye irritation	-	-	2 (0.0)	-	-	-	-	2 (0.0)
Eye movement disorder	4 (0.1)	5 (0.0)	5 (0.0)	-	-	4 (0.1)	-	18 (0.0)
Eye oedema	-	1 (0.0)	3 (0.0)	-	-	-	-	4 (0.0)
Eye pain	-	3 (0.0)	7 (0.0)	-	-	2 (0.0)	-	12 (0.0)
Eye pruritus	-	-	1 (0.0)	-	-	-	-	1 (0.0)
Eye swelling	-	3 (0.0)	5 (0.0)	-	-	1 (0.0)	-	9 (0.0)
Eyelid disorder	-	2 (0.0)	1 (0.0)	-	-	-	-	3 (0.0)
Eyelid function disorder	-	-	1 (0.0)	-	-	-	-	1 (0.0)
Eyelid oedema	-	8 (0.1)	26 (0.1)	-	1 (0.2)	4 (0.1)	-	39 (0.1)
Eyelid pain	-	-	1 (0.0)	-	-	-	-	1 (0.0)
Eyelid ptosis	1 (0.0)	1 (0.0)	5 (0.0)	-	-	-	-	7 (0.0)
Eyelids pruritus	-	-	1 (0.0)	-	-	-	-	1 (0.0)
Face oedema	-	16 (0.1)	37 (0.1)	-	-	10 (0.2)	-	63 (0.1)
Facial nerve disorder	-	-	1 (0.0)	-	-	-	-	1 (0.0)
Facial pain	-	1 (0.0)	1 (0.0)	-	-	-	-	2 (0.0)
Facial paralysis	-	2 (0.0)	6 (0.0)	-	-	-	-	8 (0.0)
Facial paresis	-	1 (0.0)	1 (0.0)	-	-	-	-	2 (0.0)
Facial spasm	-	1 (0.0)	3 (0.0)	-	-	-	-	4 (0.0)
Faecaloma	-	1 (0.0)	-	-	-	-	-	1 (0.0)
Faeces discoloured	-	6 (0.0)	1 (0.0)	-	-	-	-	7 (0.0)
Faeces hard	-	-	1 (0.0)	-	-	-	-	1 (0.0)
Faeces soft	-	1 (0.0)	-	-	-	-	-	1 (0.0)
Failure to thrive	-	1 (0.0)	-	-	-	-	-	1 (0.0)
Fall	5 (0.1)	41 (0.3)	5 (0.0)	-	-	2 (0.0)	-	53 (0.1)
Familial risk factor	-	-	1 (0.0)	-	-	-	-	1 (0.0)
Fanconi syndrome	-	-	-	-	-	1 (0.0)	-	1 (0.0)
Fascicular block	-	-	1 (0.0)	-	-	-	-	1 (0.0)

Fat embolism	-	-	2 (0.0)	-	-	-	-	2 (0.0)
Fatigue	4 (0.1)	51 (0.3)	46 (0.2)	-	-	2 (0.0)	-	103 (0.2)
Fear	-	9 (0.1)	1 (0.0)	-	-	3 (0.1)	-	13 (0.0)
Fear of death	-	-	1 (0.0)	-	-	-	-	1 (0.0)
Fear of falling	1 (0.0)	-	-	-	-	-	-	1 (0.0)
Febrile bone marrow aplasia	-	1 (0.0)	-	-	-	-	-	1 (0.0)
Febrile convulsion	-	8 (0.1)	-	-	-	-	-	8 (0.0)
Febrile infection-related epilepsy syndrome	-	1 (0.0)	1 (0.0)	-	-	-	-	2 (0.0)
Febrile neutropenia	-	4 (0.0)	1 (0.0)	-	-	-	-	5 (0.0)
Feeding disorder	-	-	1 (0.0)	1 (0.3)	-	-	-	2 (0.0)
Feeling abnormal	6 (0.2)	24 (0.2)	12 (0.0)	-	-	2 (0.0)	-	44 (0.1)
Feeling cold	1 (0.0)	4 (0.0)	19 (0.1)	-	-	-	-	24 (0.0)
Feeling drunk	-	5 (0.0)	1 (0.0)	-	-	-	-	6 (0.0)
Feeling hot	-	6 (0.0)	8 (0.0)	-	-	-	-	14 (0.0)
Feeling jittery	1 (0.0)	6 (0.0)	-	-	-	-	-	7 (0.0)
Feeling of despair	-	1 (0.0)	1 (0.0)	-	-	-	-	2 (0.0)
Female genital tract fistula	-	15 (0.1)	-	-	-	-	-	15 (0.0)
Female sterilisation	-	1 (0.0)	-	-	-	-	-	1 (0.0)
Femoral nerve palsy	-	-	1 (0.0)	-	-	-	-	1 (0.0)
Femur fracture	1 (0.0)	1 (0.0)	-	-	-	1 (0.0)	-	3 (0.0)
Fibrin D dimer increased	-	12 (0.1)	3 (0.0)	-	-	1 (0.0)	-	16 (0.0)
Fibrinolysis	-	-	-	-	-	5 (0.1)	-	5 (0.0)
Fibromyalgia	-	1 (0.0)	4 (0.0)	-	-	-	-	5 (0.0)
Fibrosis	-	1 (0.0)	-	-	-	-	-	1 (0.0)
Fine motor skill dysfunction	-	1 (0.0)	1 (0.0)	-	-	-	-	2 (0.0)
Finger deformity	-	-	1 (0.0)	-	-	-	-	1 (0.0)
Fistula	-	1 (0.0)	-	-	-	-	-	1 (0.0)
Fistula discharge	-	-	2 (0.0)	-	-	1 (0.0)	-	3 (0.0)
Fixed eruption	-	-	4 (0.0)	-	-	-	-	4 (0.0)
Flail chest	-	-	1 (0.0)	-	-	-	-	1 (0.0)
Flashback	-	-	1 (0.0)	1 (0.3)	-	-	-	2 (0.0)
Flat affect	-	1 (0.0)	-	-	-	-	-	1 (0.0)

Flatulence	-	2 (0.0)	5 (0.0)	-	-	-	-	7 (0.0)
Floppy iris syndrome	5 (0.1)	-	-	-	-	-	-	5 (0.0)
Fluid imbalance	-	-	1 (0.0)	-	-	-	-	1 (0.0)
Fluid intake reduced	-	-	1 (0.0)	-	-	-	-	1 (0.0)
Fluid retention	-	-	9 (0.0)	-	1 (0.2)	-	-	10 (0.0)
Flushing	3 (0.1)	18 (0.1)	67 (0.3)	-	-	9 (0.2)	-	97 (0.2)
Foaming at mouth	-	3 (0.0)	-	-	-	1 (0.0)	-	4 (0.0)
Focal dyscognitive seizures	-	3 (0.0)	4 (0.0)	-	-	-	-	7 (0.0)
Foetal arrhythmia	-	1 (0.0)	-	-	-	-	-	1 (0.0)
Foetal cardiac disorder	-	-	1 (0.0)	-	-	-	-	1 (0.0)
Foetal death	-	4 (0.0)	13 (0.1)	-	-	7 (0.1)	-	24 (0.0)
Foetal disorder	-	-	1 (0.0)	-	-	-	-	1 (0.0)
Foetal distress syndrome	-	3 (0.0)	3 (0.0)	-	-	1 (0.0)	-	7 (0.0)
Foetal exposure during delivery	1 (0.0)	-	7 (0.0)	-	-	6 (0.1)	-	14 (0.0)
Foetal exposure during pregnancy	2 (0.1)	30 (0.2)	82 (0.3)	2 (0.6)	6 (1.2)	37 (0.8)	4 (1.5)	163 (0.3)
Foetal exposure timing unspecified	-	-	2 (0.0)	-	-	-	-	2 (0.0)
Foetal growth restriction	-	7 (0.0)	6 (0.0)	-	2 (0.4)	4 (0.1)	-	19 (0.0)
Foetal heart rate abnormal	-	3 (0.0)	2 (0.0)	-	-	-	-	5 (0.0)
Foetal heart rate acceleration abnormality	-	-	1 (0.0)	-	-	-	-	1 (0.0)
Foetal heart rate deceleration abnormality	1 (0.0)	-	1 (0.0)	-	-	6 (0.1)	-	8 (0.0)
Foetal heart rate decreased	-	1 (0.0)	8 (0.0)	-	1 (0.2)	-	-	10 (0.0)
Foetal heart rate disorder	-	1 (0.0)	-	-	-	-	-	1 (0.0)
Foetal heart rate increased	-	1 (0.0)	-	-	-	-	-	1 (0.0)
Foetal hypokinesia	-	2 (0.0)	2 (0.0)	-	-	-	-	4 (0.0)
Foetal malformation	-	-	1 (0.0)	-	-	-	-	1 (0.0)
Foetal megacystis	-	-	-	-	-	-	1 (0.4)	1 (0.0)
Foetal non-stress test abnormal	-	1 (0.0)	-	-	-	-	-	1 (0.0)
Fontanelle bulging	-	1 (0.0)	-	-	-	-	-	1 (0.0)
Food craving	-	2 (0.0)	-	-	-	-	-	2 (0.0)
Food interaction	-	1 (0.0)	2 (0.0)	-	-	-	-	3 (0.0)
Foreign body aspiration	-	1 (0.0)	1 (0.0)	-	-	-	-	2 (0.0)
Foreign body embolism	-	1 (0.0)	-	-	-	-	-	1 (0.0)

Foreign body in gastrointestinal tract	-	1 (0.0)	-	-	-	-	-	1 (0.0)
Foreign body in respiratory tract	-	2 (0.0)	-	-	-	-	-	2 (0.0)
Foreign body in throat	-	1 (0.0)	-	-	-	-	-	1 (0.0)
Formication	-	-	-	-	-	1 (0.0)	-	1 (0.0)
Frequent bowel movements	-	20 (0.1)	-	-	-	-	-	20 (0.0)
Frustration tolerance decreased	-	1 (0.0)	-	-	-	-	-	1 (0.0)
Full blood count abnormal	-	1 (0.0)	-	-	-	-	-	1 (0.0)
Full blood count decreased	-	-	1 (0.0)	-	-	-	-	1 (0.0)
Functional gastrointestinal disorder	-	1 (0.0)	1 (0.0)	-	-	-	-	2 (0.0)
Fungal infection	1 (0.0)	3 (0.0)	-	-	-	1 (0.0)	-	5 (0.0)
Fungal sepsis	-	-	1 (0.0)	-	-	-	-	1 (0.0)
Furuncle	-	-	1 (0.0)	-	-	-	-	1 (0.0)
Gait disturbance	-	17 (0.1)	24 (0.1)	2 (0.6)	-	2 (0.0)	-	45 (0.1)
Gait inability	-	7 (0.0)	6 (0.0)	-	-	1 (0.0)	-	14 (0.0)
Gallbladder disorder	-	-	2 (0.0)	-	-	-	-	2 (0.0)
Gallbladder enlargement	-	-	1 (0.0)	-	-	-	-	1 (0.0)
Gallbladder operation	-	1 (0.0)	-	-	-	-	-	1 (0.0)
Gallbladder rupture	-	-	1 (0.0)	-	-	-	-	1 (0.0)
Gamma-glutamyltransferase increased	1 (0.0)	10 (0.1)	10 (0.0)	-	-	2 (0.0)	-	23 (0.0)
Gangrene	-	1 (0.0)	1 (0.0)	-	-	-	-	2 (0.0)
Gastric haemorrhage	1 (0.0)	3 (0.0)	1 (0.0)	-	-	-	-	5 (0.0)
Gastric perforation	-	1 (0.0)	-	-	-	-	-	1 (0.0)
Gastric polyps	-	1 (0.0)	-	-	-	-	-	1 (0.0)
Gastric residual increased	-	1 (0.0)	-	-	-	-	-	1 (0.0)
Gastric ulcer	-	-	1 (0.0)	-	-	-	-	1 (0.0)
Gastric varices haemorrhage	-	-	1 (0.0)	-	-	-	-	1 (0.0)
Gastritis	-	3 (0.0)	-	-	-	-	-	3 (0.0)
Gastritis erosive	-	1 (0.0)	1 (0.0)	-	-	-	-	2 (0.0)
Gastritis haemorrhagic	-	-	-	-	-	1 (0.0)	-	1 (0.0)
Gastroenteritis	-	-	1 (0.0)	-	-	-	-	1 (0.0)
Gastrointestinal disorder	-	10 (0.1)	3 (0.0)	-	-	-	-	13 (0.0)
Gastrointestinal haemorrhage	1 (0.0)	11 (0.1)	6 (0.0)	-	1 (0.2)	2 (0.0)	-	21 (0.0)

Gastrointestinal hypomotility	-	1 (0.0)	2 (0.0)	1 (0.3)	-	1 (0.0)	-	5 (0.0)
Gastrointestinal infection	-	1 (0.0)	-	-	-	1 (0.0)	-	2 (0.0)
Gastrointestinal inflammation	-	2 (0.0)	-	-	-	-	-	2 (0.0)
Gastrointestinal ischaemia	-	-	2 (0.0)	-	-	-	-	2 (0.0)
Gastrointestinal motility disorder	-	3 (0.0)	2 (0.0)	-	-	2 (0.0)	-	7 (0.0)
Gastrointestinal mucosa hyperaemia	-	3 (0.0)	-	-	-	-	-	3 (0.0)
Gastrointestinal mucosal disorder	-	1 (0.0)	-	-	-	-	-	1 (0.0)
Gastrointestinal necrosis	-	-	1 (0.0)	-	-	-	-	1 (0.0)
Gastrointestinal obstruction	-	-	1 (0.0)	-	-	-	-	1 (0.0)
Gastrointestinal oedema	-	1 (0.0)	-	-	-	-	-	1 (0.0)
Gastrointestinal pain	-	1 (0.0)	-	-	-	-	-	1 (0.0)
Gastrointestinal perforation	1 (0.0)	-	-	-	-	-	-	1 (0.0)
Gastrointestinal scarring	-	2 (0.0)	-	-	-	-	-	2 (0.0)
Gastrointestinal sounds abnormal	2 (0.1)	2 (0.0)	-	-	-	1 (0.0)	-	5 (0.0)
Gastrooesophageal reflux disease	-	1 (0.0)	4 (0.0)	-	-	1 (0.0)	-	6 (0.0)
Gastrooesophageal sphincter insufficiency	-	-	1 (0.0)	-	-	-	-	1 (0.0)
Gaze palsy	-	2 (0.0)	11 (0.0)	-	-	-	-	13 (0.0)
Gene mutation	-	-	-	-	-	2 (0.0)	-	2 (0.0)
General anaesthesia	-	1 (0.0)	-	-	-	-	-	1 (0.0)
General physical condition abnormal	-	-	1 (0.0)	-	-	-	-	1 (0.0)
General physical health deterioration	4 (0.1)	20 (0.1)	24 (0.1)	2 (0.6)	-	1 (0.0)	-	51 (0.1)
General symptom	-	1 (0.0)	-	-	-	-	-	1 (0.0)
Generalised oedema	-	3 (0.0)	5 (0.0)	-	-	-	-	8 (0.0)
Generalised onset non-motor seizure	-	1 (0.0)	-	-	-	-	-	1 (0.0)
Generalised tonic-clonic seizure	4 (0.1)	50 (0.3)	54 (0.2)	2 (0.6)	-	13 (0.3)	5 (1.9)	128 (0.3)
Genital erosion	-	2 (0.0)	-	-	-	-	-	2 (0.0)
Genital haemorrhage	-	6 (0.0)	-	-	-	-	-	6 (0.0)
Genito-pelvic pain/penetration disorder	-	-	-	-	-	1 (0.0)	-	1 (0.0)
Gestational diabetes	-	1 (0.0)	-	1 (0.3)	-	-	-	2 (0.0)
Gestational hypertension	-	-	3 (0.0)	-	-	-	-	3 (0.0)
Gingival cancer	-	-	1 (0.0)	-	-	-	-	1 (0.0)
Gingival disorder	-	1 (0.0)	-	-	-	-	-	1 (0.0)

Gingival pain	-	1 (0.0)	1 (0.0)	-	-	-	-	2 (0.0)
Gingival recession	-	1 (0.0)	-	-	-	-	-	1 (0.0)
Gingivitis	-	1 (0.0)	-	-	-	-	-	1 (0.0)
Glaucoma	-	1 (0.0)	1 (0.0)	-	-	-	-	2 (0.0)
Glomerular filtration rate decreased	-	-	1 (0.0)	-	-	-	-	1 (0.0)
Glomerulonephritis membranous	-	-	1 (0.0)	-	-	-	-	1 (0.0)
Glossitis	-	-	1 (0.0)	-	-	-	-	1 (0.0)
Glossodynia	-	-	2 (0.0)	-	-	-	-	2 (0.0)
Glossoptosis	8 (0.2)	9 (0.1)	6 (0.0)	3 (0.9)	-	-	1 (0.4)	27 (0.1)
Gluten sensitivity	-	1 (0.0)	-	-	-	-	-	1 (0.0)
Gout	-	-	1 (0.0)	-	-	-	-	1 (0.0)
Gouty tophus	-	-	1 (0.0)	-	-	-	-	1 (0.0)
Graft versus host disease in gastrointestinal tract	1 (0.0)	1 (0.0)	-	-	-	1 (0.0)	-	3 (0.0)
Graft versus host disease in liver	-	1 (0.0)	-	-	-	-	-	1 (0.0)
Graft versus host disease in skin	-	1 (0.0)	-	-	-	1 (0.0)	-	2 (0.0)
Granulocyte count increased	-	-	1 (0.0)	-	-	-	-	1 (0.0)
Granulocytopenia	-	1 (0.0)	-	-	-	-	-	1 (0.0)
Granuloma	-	2 (0.0)	-	-	-	-	-	2 (0.0)
Grimacing	-	3 (0.0)	1 (0.0)	-	-	-	-	4 (0.0)
Grip strength decreased	-	-	1 (0.0)	-	-	-	-	1 (0.0)
Groin pain	-	2 (0.0)	-	-	-	-	-	2 (0.0)
Gross motor delay	-	1 (0.0)	-	-	-	-	-	1 (0.0)
Guillain-Barre syndrome	-	1 (0.0)	1 (0.0)	-	-	1 (0.0)	-	3 (0.0)
H1N1 influenza	-	-	1 (0.0)	-	-	-	-	1 (0.0)
Haemangioma	-	1 (0.0)	3 (0.0)	-	-	1 (0.0)	-	5 (0.0)
Haemangioma congenital	-	1 (0.0)	-	-	-	-	-	1 (0.0)
Haemangioma of liver	-	1 (0.0)	-	-	-	-	-	1 (0.0)
Haematemesis	-	2 (0.0)	6 (0.0)	1 (0.3)	-	3 (0.1)	-	12 (0.0)
Haematochezia	1 (0.0)	19 (0.1)	-	-	-	-	-	20 (0.0)
Haematocrit decreased	4 (0.1)	3 (0.0)	2 (0.0)	-	-	-	-	9 (0.0)
Haematoma	-	4 (0.0)	5 (0.0)	-	-	-	-	9 (0.0)
Haematuria	1 (0.0)	2 (0.0)	5 (0.0)	-	-	1 (0.0)	-	9 (0.0)

Haematuria traumatic	-	-	-	-	-	1 (0.0)	-	1 (0.0)
Haemobilia	1 (0.0)	-	-	-	-	-	-	1 (0.0)
Haemodialysis	-	-	1 (0.0)	-	-	-	-	1 (0.0)
Haemodilution	-	-	2 (0.0)	-	-	-	-	2 (0.0)
Haemodynamic instability	4 (0.1)	20 (0.1)	62 (0.2)	-	3 (0.6)	18 (0.4)	-	107 (0.2)
Haemodynamic test abnormal	-	1 (0.0)	2 (0.0)	-	-	-	-	3 (0.0)
Haemofiltration	-	-	2 (0.0)	-	-	-	-	2 (0.0)
Haemoglobin decreased	6 (0.2)	9 (0.1)	5 (0.0)	-	-	3 (0.1)	-	23 (0.0)
Haemoglobinuria	-	1 (0.0)	2 (0.0)	-	-	-	-	3 (0.0)
Haemolysis	-	1 (0.0)	3 (0.0)	-	-	-	-	4 (0.0)
Haemolytic anaemia	-	4 (0.0)	4 (0.0)	-	-	2 (0.0)	-	10 (0.0)
Haemoperitoneum	-	-	4 (0.0)	-	-	4 (0.1)	-	8 (0.0)
Haemophagocytic lymphohistiocytosis	-	1 (0.0)	2 (0.0)	-	-	2 (0.0)	2 (0.8)	7 (0.0)
Haemophilia	1 (0.0)	-	-	-	-	-	-	1 (0.0)
Haemoptysis	-	2 (0.0)	10 (0.0)	-	-	2 (0.0)	-	14 (0.0)
Haemorrhage	2 (0.1)	17 (0.1)	36 (0.1)	-	-	3 (0.1)	-	58 (0.1)
Haemorrhage intracranial	-	1 (0.0)	1 (0.0)	-	-	-	-	2 (0.0)
Haemorrhage subcutaneous	2 (0.1)	1 (0.0)	-	-	-	-	-	3 (0.0)
Haemorrhagic diathesis	1 (0.0)	-	-	-	-	-	-	1 (0.0)
Haemorrhagic necrotic pancreatitis	-	-	2 (0.0)	-	-	-	-	2 (0.0)
Haemorrhagic stroke	-	1 (0.0)	2 (0.0)	-	-	-	-	3 (0.0)
Haemorrhagic transformation stroke	-	1 (0.0)	-	-	-	-	-	1 (0.0)
Haemorrhoids	-	8 (0.1)	1 (0.0)	-	-	-	-	9 (0.0)
Haemothorax	1 (0.0)	-	2 (0.0)	-	-	-	-	3 (0.0)
Hair colour changes	-	-	5 (0.0)	-	-	-	-	5 (0.0)
Hallucination	5 (0.1)	64 (0.4)	27 (0.1)	1 (0.3)	1 (0.2)	4 (0.1)	-	102 (0.2)
Hallucination, auditory	1 (0.0)	5 (0.0)	2 (0.0)	-	-	-	-	8 (0.0)
Hallucination, olfactory	-	1 (0.0)	-	-	-	-	-	1 (0.0)
Hallucination, visual	2 (0.1)	20 (0.1)	4 (0.0)	-	1 (0.2)	1 (0.0)	-	28 (0.1)
Hallucinations, mixed	1 (0.0)	1 (0.0)	2 (0.0)	-	-	-	-	4 (0.0)
Harlequin skin reaction	-	1 (0.0)	-	-	-	-	-	1 (0.0)
Harlequin syndrome	-	-	3 (0.0)	-	-	-	-	3 (0.0)

Hashimoto's encephalopathy	-	-	-	-	-	1 (0.0)	-	1 (0.0)
Head deformity	1 (0.0)	-	-	-	-	-	-	1 (0.0)
Head discomfort	-	2 (0.0)	2 (0.0)	-	-	-	-	4 (0.0)
Head injury	2 (0.1)	6 (0.0)	-	-	-	1 (0.0)	-	9 (0.0)
Head titubation	-	-	2 (0.0)	-	-	-	-	2 (0.0)
Headache	3 (0.1)	92 (0.6)	124 (0.5)	-	-	17 (0.4)	-	236 (0.5)
Heart rate abnormal	1 (0.0)	4 (0.0)	2 (0.0)	-	-	-	-	7 (0.0)
Heart rate decreased	40 (1.0)	28 (0.2)	41 (0.2)	-	2 (0.4)	9 (0.2)	-	120 (0.2)
Heart rate increased	8 (0.2)	20 (0.1)	59 (0.2)	-	-	13 (0.3)	-	100 (0.2)
Heart rate irregular	-	6 (0.0)	1 (0.0)	-	-	1 (0.0)	-	8 (0.0)
Heart sounds abnormal	-	-	2 (0.0)	-	-	-	-	2 (0.0)
Heart valve incompetence	-	1 (0.0)	-	-	-	-	-	1 (0.0)
Heart valve stenosis	-	1 (0.0)	-	-	-	-	-	1 (0.0)
Heavy menstrual bleeding	-	-	1 (0.0)	-	-	-	-	1 (0.0)
Helicobacter infection	-	1 (0.0)	-	-	-	-	-	1 (0.0)
HELLP syndrome	-	-	1 (0.0)	-	-	-	-	1 (0.0)
Hemiparesis	1 (0.0)	2 (0.0)	6 (0.0)	-	-	1 (0.0)	-	10 (0.0)
Hemiplegia	1 (0.0)	2 (0.0)	9 (0.0)	-	2 (0.4)	-	-	14 (0.0)
Hemiplegic migraine	-	2 (0.0)	1 (0.0)	-	-	4 (0.1)	-	7 (0.0)
Heparin-induced thrombocytopenia	-	1 (0.0)	1 (0.0)	-	-	-	-	2 (0.0)
Hepatic cytolysis	1 (0.0)	14 (0.1)	44 (0.2)	-	-	11 (0.2)	-	70 (0.1)
Hepatic encephalopathy	-	11 (0.1)	10 (0.0)	-	-	-	-	21 (0.0)
Hepatic enzyme abnormal	-	-	1 (0.0)	-	-	-	-	1 (0.0)
Hepatic enzyme increased	-	9 (0.1)	27 (0.1)	-	-	2 (0.0)	1 (0.4)	39 (0.1)
Hepatic failure	-	19 (0.1)	31 (0.1)	-	-	5 (0.1)	-	55 (0.1)
Hepatic function abnormal	7 (0.2)	24 (0.2)	34 (0.1)	1 (0.3)	2 (0.4)	3 (0.1)	-	71 (0.1)
Hepatic ischaemia	-	-	-	-	-	1 (0.0)	-	1 (0.0)
Hepatic necrosis	-	1 (0.0)	5 (0.0)	-	-	-	-	6 (0.0)
Hepatic steatosis	-	1 (0.0)	5 (0.0)	-	-	1 (0.0)	-	7 (0.0)
Hepatitis	-	5 (0.0)	30 (0.1)	-	-	3 (0.1)	-	38 (0.1)
Hepatitis acute	-	1 (0.0)	12 (0.0)	-	-	5 (0.1)	-	18 (0.0)
Hepatitis B	-	-	1 (0.0)	-	-	-	-	1 (0.0)

Hepatitis C	-	-	1 (0.0)	-	-	-	-	1 (0.0)
Hepatitis cholestatic	-	-	6 (0.0)	-	-	-	-	6 (0.0)
Hepatitis fulminant	1 (0.0)	7 (0.0)	4 (0.0)	-	-	4 (0.1)	-	16 (0.0)
Hepatitis toxic	-	-	4 (0.0)	-	-	-	-	4 (0.0)
Hepatobiliary disease	-	-	3 (0.0)	-	-	-	-	3 (0.0)
Hepatocellular injury	-	4 (0.0)	10 (0.0)	-	-	1 (0.0)	-	15 (0.0)
Hepatomegaly	-	3 (0.0)	7 (0.0)	-	-	-	-	10 (0.0)
Hepatorenal failure	-	-	9 (0.0)	-	-	-	-	9 (0.0)
Hepatorenal syndrome	-	-	1 (0.0)	-	-	1 (0.0)	-	2 (0.0)
Hepatosplenomegaly	-	1 (0.0)	-	-	-	-	-	1 (0.0)
Hepatotoxicity	-	1 (0.0)	15 (0.1)	-	-	1 (0.0)	-	17 (0.0)
Herpes simplex test positive	-	-	1 (0.0)	-	-	-	-	1 (0.0)
Herpes virus infection	-	1 (0.0)	-	-	-	-	-	1 (0.0)
Herpes zoster	1 (0.0)	1 (0.0)	-	-	-	-	-	2 (0.0)
Hiatus hernia	-	2 (0.0)	-	-	-	-	-	2 (0.0)
Hiccups	3 (0.1)	9 (0.1)	21 (0.1)	-	-	-	-	33 (0.1)
High density lipoprotein decreased	-	-	1 (0.0)	-	-	-	-	1 (0.0)
High risk sexual behaviour	-	1 (0.0)	-	-	-	-	-	1 (0.0)
Hip arthroplasty	-	1 (0.0)	1 (0.0)	-	-	-	-	2 (0.0)
Histamine abnormal	-	-	-	-	-	1 (0.0)	-	1 (0.0)
Histamine level increased	-	1 (0.0)	4 (0.0)	-	-	-	-	5 (0.0)
HIV infection	-	1 (0.0)	-	-	-	-	-	1 (0.0)
HIV-associated neurocognitive disorder	-	-	1 (0.0)	-	-	-	-	1 (0.0)
Hodgkin's disease stage IV	-	-	1 (0.0)	-	-	-	-	1 (0.0)
Homicide	-	4 (0.0)	-	-	-	1 (0.0)	-	5 (0.0)
Horner's syndrome	-	1 (0.0)	2 (0.0)	-	-	1 (0.0)	-	4 (0.0)
Hospitalisation	-	6 (0.0)	1 (0.0)	-	-	-	-	7 (0.0)
Hostility	-	1 (0.0)	-	-	-	-	-	1 (0.0)
Hot flush	-	5 (0.0)	4 (0.0)	-	-	-	-	9 (0.0)
Humerus fracture	-	2 (0.0)	-	-	-	-	-	2 (0.0)
Hunger	-	1 (0.0)	1 (0.0)	-	-	-	-	2 (0.0)
Hydrocephalus	-	-	1 (0.0)	-	-	-	-	1 (0.0)

Hydrops foetalis	-	-	-	-	-	1 (0.0)	-	1 (0.0)
Hydrothorax	-	-	1 (0.0)	-	-	2 (0.0)	-	3 (0.0)
Hyperacusis	-	1 (0.0)	1 (0.0)	-	-	-	-	2 (0.0)
Hyperaemia	-	7 (0.0)	10 (0.0)	-	-	-	-	17 (0.0)
Hyperaesthesia	1 (0.0)	11 (0.1)	5 (0.0)	-	-	-	-	17 (0.0)
Hyperammonaemia	-	1 (0.0)	5 (0.0)	-	-	2 (0.0)	-	8 (0.0)
Hyperammonaemic encephalopathy	-	2 (0.0)	3 (0.0)	-	-	1 (0.0)	-	6 (0.0)
Hyperamylasaemia	-	-	2 (0.0)	-	-	-	-	2 (0.0)
Hyperbilirubinaemia	-	4 (0.0)	11 (0.0)	1 (0.3)	-	1 (0.0)	-	17 (0.0)
Hypercalcaemia	1 (0.0)	-	1 (0.0)	-	-	2 (0.0)	-	4 (0.0)
Hypercapnia	4 (0.1)	16 (0.1)	23 (0.1)	1 (0.3)	-	8 (0.2)	-	52 (0.1)
Hypercapnic coma	-	1 (0.0)	-	-	-	-	-	1 (0.0)
Hyperchloraemia	-	2 (0.0)	1 (0.0)	-	-	-	-	3 (0.0)
Hypercholesterolaemia	-	-	2 (0.0)	-	-	-	-	2 (0.0)
Hypercoagulation	-	1 (0.0)	1 (0.0)	-	-	-	-	2 (0.0)
Hypercreatininaemia	-	-	3 (0.0)	-	-	-	-	3 (0.0)
Hyperdynamic left ventricle	-	2 (0.0)	-	-	-	2 (0.0)	1 (0.4)	5 (0.0)
Hyperexplexia	1 (0.0)	1 (0.0)	-	-	-	-	-	2 (0.0)
Hyperfibrinolysis	-	-	1 (0.0)	-	-	-	-	1 (0.0)
Hyperglycaemia	1 (0.0)	4 (0.0)	11 (0.0)	-	-	3 (0.1)	-	19 (0.0)
Hyperhidrosis	6 (0.2)	25 (0.2)	34 (0.1)	2 (0.6)	-	12 (0.3)	2 (0.8)	81 (0.2)
Hyperkalaemia	2 (0.1)	14 (0.1)	71 (0.3)	-	1 (0.2)	14 (0.3)	-	102 (0.2)
Hyperkeratosis	-	-	1 (0.0)	-	-	-	-	1 (0.0)
Hyperkinesia	-	2 (0.0)	2 (0.0)	1 (0.3)	-	-	-	5 (0.0)
Hyperlactacidaemia	-	1 (0.0)	14 (0.1)	-	1 (0.2)	-	-	16 (0.0)
Hyperleukocytosis	-	-	3 (0.0)	-	-	-	-	3 (0.0)
Hyperlipasaemia	-	1 (0.0)	1 (0.0)	-	-	-	-	2 (0.0)
Hyperlipidaemia	-	-	17 (0.1)	-	-	-	-	17 (0.0)
Hypermagnesaemia	-	-	2 (0.0)	-	-	-	-	2 (0.0)
Hypermetabolism	-	-	-	-	-	1 (0.0)	-	1 (0.0)
Hypernatraemia	11 (0.3)	4 (0.0)	2 (0.0)	-	-	4 (0.1)	-	21 (0.0)
Hyperparathyroidism	-	-	1 (0.0)	-	-	-	-	1 (0.0)

Hyperparathyroidism secondary	-	1 (0.0)	-	-	-	-	-	1 (0.0)
Hyperphagia	-	1 (0.0)	-	-	-	-	-	1 (0.0)
Hyperphosphataemia	-	-	7 (0.0)	-	-	-	-	7 (0.0)
Hyperprolactinaemia	-	1 (0.0)	-	-	-	-	-	1 (0.0)
Hyperpyrexia	23 (0.6)	2 (0.0)	8 (0.0)	-	-	2 (0.0)	-	35 (0.1)
Hyperreflexia	1 (0.0)	6 (0.0)	10 (0.0)	-	-	6 (0.1)	-	23 (0.0)
Hypersensitivity	3 (0.1)	52 (0.3)	201 (0.8)	1 (0.3)	2 (0.4)	28 (0.6)	1 (0.4)	288 (0.6)
Hypersensitivity pneumonitis	-	1 (0.0)	-	-	-	-	-	1 (0.0)
Hypersexuality	-	4 (0.0)	-	-	-	-	-	4 (0.0)
Hypersomnia	2 (0.1)	9 (0.1)	1 (0.0)	-	-	1 (0.0)	-	13 (0.0)
Hypertension	31 (0.8)	53 (0.4)	74 (0.3)	2 (0.6)	2 (0.4)	19 (0.4)	3 (1.1)	184 (0.4)
Hypertensive crisis	2 (0.1)	5 (0.0)	5 (0.0)	-	-	4 (0.1)	-	16 (0.0)
Hypertensive emergency	-	-	-	1 (0.3)	-	-	-	1 (0.0)
Hypertensive heart disease	-	-	1 (0.0)	-	-	-	-	1 (0.0)
Hyperthermia	65 (1.7)	13 (0.1)	46 (0.2)	1 (0.3)	2 (0.4)	2 (0.0)	1 (0.4)	130 (0.3)
Hyperthermia malignant	11 (0.3)	13 (0.1)	145 (0.6)	2 (0.6)	6 (1.2)	20 (0.4)	1 (0.4)	198 (0.4)
Hyperthyroidism	-	1 (0.0)	1 (0.0)	-	-	2 (0.0)	-	4 (0.0)
Hypertonia	-	9 (0.1)	20 (0.1)	-	-	4 (0.1)	1 (0.4)	34 (0.1)
Hypertransaminasaemia	1 (0.0)	4 (0.0)	19 (0.1)	-	-	-	-	24 (0.0)
Hypertriglyceridaemia	-	2 (0.0)	62 (0.2)	-	-	6 (0.1)	-	70 (0.1)
Hypertrophic cardiomyopathy	-	-	1 (0.0)	-	-	5 (0.1)	-	6 (0.0)
Hypertrophy	-	-	1 (0.0)	-	-	-	-	1 (0.0)
Hyperuricaemia	-	-	2 (0.0)	-	-	-	-	2 (0.0)
Hyperuricosuria	-	-	2 (0.0)	-	-	-	-	2 (0.0)
Hyperventilation	2 (0.1)	9 (0.1)	10 (0.0)	-	-	2 (0.0)	-	23 (0.0)
Hypervigilance	-	1 (0.0)	-	-	-	-	-	1 (0.0)
Hyperviscosity syndrome	-	-	2 (0.0)	-	-	-	-	2 (0.0)
Hypervolaemia	-	2 (0.0)	3 (0.0)	-	-	2 (0.0)	-	7 (0.0)
Hypnagogic hallucination	-	-	1 (0.0)	-	-	-	-	1 (0.0)
Hypoacusis	-	1 (0.0)	7 (0.0)	-	-	-	-	8 (0.0)
Hypoaesthesia	-	20 (0.1)	17 (0.1)	-	-	3 (0.1)	1 (0.4)	41 (0.1)
Hypoaesthesia oral	-	8 (0.1)	2 (0.0)	-	-	-	-	10 (0.0)

Hypoalbuminaemia	2 (0.1)	2 (0.0)	1 (0.0)	-	-	-	-	5 (0.0)
Hypocalcaemia	-	4 (0.0)	12 (0.0)	1 (0.3)	-	2 (0.0)	-	19 (0.0)
Hypocapnia	1 (0.0)	-	4 (0.0)	-	-	-	-	5 (0.0)
Hypocoagulable state	-	-	2 (0.0)	-	-	-	-	2 (0.0)
Hypofibrinogenaemia	-	-	1 (0.0)	-	-	1 (0.0)	-	2 (0.0)
Hypogeusia	-	-	4 (0.0)	-	-	1 (0.0)	-	5 (0.0)
Hypoglossal nerve paralysis	-	-	1 (0.0)	-	-	-	-	1 (0.0)
Hypoglycaemia	5 (0.1)	4 (0.0)	5 (0.0)	-	-	2 (0.0)	1 (0.4)	17 (0.0)
Hypoglycaemia neonatal	-	-	1 (0.0)	-	-	1 (0.0)	-	2 (0.0)
Hypokalaemia	4 (0.1)	5 (0.0)	27 (0.1)	-	-	7 (0.1)	1 (0.4)	44 (0.1)
Hypokinesia	1 (0.0)	6 (0.0)	7 (0.0)	-	-	2 (0.0)	-	16 (0.0)
Hypomagnesaemia	-	-	5 (0.0)	-	-	3 (0.1)	-	8 (0.0)
Hypomenorrhoea	-	6 (0.0)	-	-	-	-	-	6 (0.0)
Hypometabolism	-	-	-	-	-	1 (0.0)	-	1 (0.0)
Hyponatraemia	-	11 (0.1)	4 (0.0)	-	1 (0.2)	5 (0.1)	-	21 (0.0)
Hyponatraemic encephalopathy	-	-	1 (0.0)	-	-	-	-	1 (0.0)
Hypoparathyroidism	-	-	1 (0.0)	-	-	-	-	1 (0.0)
Hypoperfusion	-	-	4 (0.0)	-	-	-	-	4 (0.0)
Hypophagia	1 (0.0)	3 (0.0)	1 (0.0)	-	-	-	-	5 (0.0)
Hypophosphataemia	-	1 (0.0)	8 (0.0)	-	-	1 (0.0)	-	10 (0.0)
Hypoplastic left heart syndrome	-	1 (0.0)	-	-	-	-	-	1 (0.0)
Hypopnoea	7 (0.2)	14 (0.1)	6 (0.0)	-	1 (0.2)	5 (0.1)	-	33 (0.1)
Hypoproteinaemia	-	-	-	-	1 (0.2)	-	-	1 (0.0)
Hyporeflexia	-	-	4 (0.0)	-	-	2 (0.0)	-	6 (0.0)
Hyporesponsive to stimuli	1 (0.0)	5 (0.0)	2 (0.0)	-	-	3 (0.1)	-	11 (0.0)
Hyposmia	-	1 (0.0)	-	-	-	-	-	1 (0.0)
Hypotension	176 (4.6)	294 (2.0)	1011 (4.1)	9 (2.7)	18 (3.6)	187 (4.0)	6 (2.3)	1701 (3.4)
Hypothermia	3 (0.1)	6 (0.0)	24 (0.1)	1 (0.3)	-	4 (0.1)	2 (0.8)	40 (0.1)
Hypothermia neonatal	-	-	1 (0.0)	-	-	-	-	1 (0.0)
Hypothyroidism	-	2 (0.0)	-	-	-	2 (0.0)	-	4 (0.0)
Hypotonia	6 (0.2)	24 (0.2)	19 (0.1)	1 (0.3)	-	3 (0.1)	2 (0.8)	55 (0.1)
Hypotonia neonatal	-	3 (0.0)	1 (0.0)	-	-	3 (0.1)	-	7 (0.0)

Hypotonic-hyporesponsive episode	-	-	3 (0.0)	-	-	-	-	3 (0.0)
Hypotony of eye	-	-	2 (0.0)	-	-	-	-	2 (0.0)
Hypoventilation	2 (0.1)	16 (0.1)	50 (0.2)	-	1 (0.2)	10 (0.2)	-	79 (0.2)
Hypoventilation neonatal	-	-	1 (0.0)	-	-	-	-	1 (0.0)
Hypovitaminosis	-	1 (0.0)	-	-	-	-	-	1 (0.0)
Hypovolaemia	3 (0.1)	-	6 (0.0)	-	1 (0.2)	5 (0.1)	-	15 (0.0)
Hypovolaemic shock	1 (0.0)	3 (0.0)	8 (0.0)	-	-	8 (0.2)	-	20 (0.0)
Hypoxia	18 (0.5)	59 (0.4)	100 (0.4)	2 (0.6)	3 (0.6)	19 (0.4)	2 (0.8)	203 (0.4)
Hypoxic-ischaemic encephalopathy	1 (0.0)	25 (0.2)	26 (0.1)	1 (0.3)	-	2 (0.0)	-	55 (0.1)
Iatrogenic injury	-	-	2 (0.0)	-	-	-	-	2 (0.0)
Idiopathic interstitial pneumonia	-	-	7 (0.0)	-	-	-	-	7 (0.0)
Idiopathic intracranial hypertension	-	1 (0.0)	-	-	-	-	-	1 (0.0)
Idiopathic pulmonary fibrosis	2 (0.1)	-	-	-	-	-	-	2 (0.0)
Idiosyncratic drug reaction	-	-	2 (0.0)	-	-	-	-	2 (0.0)
IIIrd nerve paralysis	-	-	1 (0.0)	-	-	-	-	1 (0.0)
Ileus	4 (0.1)	5 (0.0)	4 (0.0)	-	-	4 (0.1)	-	17 (0.0)
Ileus paralytic	1 (0.0)	4 (0.0)	-	-	-	1 (0.0)	-	6 (0.0)
Ill-defined disorder	-	5 (0.0)	2 (0.0)	-	-	-	-	7 (0.0)
Illness	-	5 (0.0)	3 (0.0)	-	-	-	-	8 (0.0)
Illusion	-	1 (0.0)	-	-	-	-	-	1 (0.0)
Imaging procedure abnormal	-	-	1 (0.0)	-	-	-	-	1 (0.0)
Immobile	1 (0.0)	1 (0.0)	1 (0.0)	-	-	-	-	3 (0.0)
Immune effector cell-associated neurotoxicity syndrome	-	5 (0.0)	-	-	-	-	-	5 (0.0)
Immune reconstitution inflammatory syndrome	-	1 (0.0)	-	-	-	-	-	1 (0.0)
Immune system disorder	-	-	1 (0.0)	-	-	-	-	1 (0.0)
Immunodeficiency	-	1 (0.0)	2 (0.0)	-	-	-	-	3 (0.0)
Immunosuppressant drug level increased	-	2 (0.0)	1 (0.0)	-	-	-	-	3 (0.0)
Immunosuppression	-	2 (0.0)	-	-	-	-	-	2 (0.0)
Impaired driving ability	-	1 (0.0)	1 (0.0)	-	-	-	-	2 (0.0)
Impaired gastric emptying	2 (0.1)	4 (0.0)	4 (0.0)	-	-	2 (0.0)	-	12 (0.0)
Impaired quality of life	-	-	1 (0.0)	-	-	-	-	1 (0.0)
Impaired reasoning	-	1 (0.0)	-	-	-	-	-	1 (0.0)

Impaired work ability	-	2 (0.0)	2 (0.0)	-	-	-	-	4 (0.0)
Impatience	-	5 (0.0)	-	-	-	-	-	5 (0.0)
Implant site extravasation	-	1 (0.0)	-	-	-	-	-	1 (0.0)
Implant site infection	-	-	-	-	-	1 (0.0)	-	1 (0.0)
Impulsive behaviour	-	4 (0.0)	1 (0.0)	-	-	1 (0.0)	-	6 (0.0)
Inadequate analgesia	-	1 (0.0)	5 (0.0)	-	-	-	-	6 (0.0)
Inadequate aseptic technique in use of product	-	-	6 (0.0)	-	-	-	-	6 (0.0)
Inappropriate affect	-	1 (0.0)	1 (0.0)	-	-	-	-	2 (0.0)
Inappropriate antidiuretic hormone secretion	-	2 (0.0)	1 (0.0)	-	1 (0.2)	1 (0.0)	-	5 (0.0)
Inappropriate schedule of product administration	-	3 (0.0)	1 (0.0)	-	1 (0.2)	-	-	5 (0.0)
Inborn error of lipid metabolism	-	-	1 (0.0)	-	-	-	-	1 (0.0)
Incision site haemorrhage	-	-	-	-	-	1 (0.0)	-	1 (0.0)
Incisional hernia	-	1 (0.0)	-	-	-	-	-	1 (0.0)
Incoherent	1 (0.0)	4 (0.0)	2 (0.0)	-	-	-	-	7 (0.0)
Incontinence	-	1 (0.0)	-	-	-	-	1 (0.4)	2 (0.0)
Incorrect dosage administered	1 (0.0)	3 (0.0)	2 (0.0)	-	-	1 (0.0)	-	7 (0.0)
Incorrect dose administered	8 (0.2)	54 (0.4)	22 (0.1)	-	1 (0.2)	1 (0.0)	3 (1.1)	89 (0.2)
Incorrect dose administered by device	-	1 (0.0)	-	-	-	-	-	1 (0.0)
Incorrect dose administered by product	-	1 (0.0)	-	-	-	-	-	1 (0.0)
Incorrect drug administration rate	10 (0.3)	12 (0.1)	8 (0.0)	-	1 (0.2)	-	-	31 (0.1)
Incorrect product administration duration	2 (0.1)	5 (0.0)	3 (0.0)	-	-	-	-	10 (0.0)
Incorrect product dosage form administered	-	1 (0.0)	-	-	-	-	-	1 (0.0)
Incorrect product formulation administered	-	1 (0.0)	-	-	-	-	-	1 (0.0)
Incorrect route of product administration	9 (0.2)	44 (0.3)	32 (0.1)	-	-	1 (0.0)	-	86 (0.2)
Increased appetite	-	3 (0.0)	-	-	-	-	-	3 (0.0)
Increased bronchial secretion	-	7 (0.0)	6 (0.0)	1 (0.3)	-	5 (0.1)	-	19 (0.0)
Increased upper airway secretion	-	2 (0.0)	5 (0.0)	1 (0.3)	-	-	-	8 (0.0)
Induration	-	2 (0.0)	1 (0.0)	-	-	-	-	3 (0.0)
Infantile apnoea	2 (0.1)	2 (0.0)	3 (0.0)	-	-	1 (0.0)	-	8 (0.0)
Infarction	-	2 (0.0)	-	-	-	-	-	2 (0.0)
Infection	2 (0.1)	7 (0.0)	7 (0.0)	-	-	9 (0.2)	-	25 (0.1)
Infection reactivation	1 (0.0)	-	-	-	-	-	-	1 (0.0)

Infective pulmonary exacerbation of cystic fibrosis	-	2 (0.0)	-	-	-	-	-	2 (0.0)
Inflammation	-	9 (0.1)	10 (0.0)	-	-	2 (0.0)	-	21 (0.0)
Inflammation of wound	-	1 (0.0)	-	-	-	-	-	1 (0.0)
Inflammatory marker increased	-	-	5 (0.0)	-	-	-	-	5 (0.0)
Influenza	-	2 (0.0)	2 (0.0)	-	-	-	-	4 (0.0)
Influenza A virus test positive	-	2 (0.0)	-	-	-	-	-	2 (0.0)
Influenza B virus test positive	-	1 (0.0)	-	-	-	-	-	1 (0.0)
Influenza like illness	-	5 (0.0)	3 (0.0)	-	-	-	-	8 (0.0)
Infrequent bowel movements	-	2 (0.0)	-	-	-	-	-	2 (0.0)
Infusion related hypersensitivity reaction	-	-	3 (0.0)	-	-	-	-	3 (0.0)
Infusion related reaction	-	15 (0.1)	4 (0.0)	-	-	-	-	19 (0.0)
Infusion site cellulitis	-	-	1 (0.0)	-	-	-	-	1 (0.0)
Infusion site discharge	-	-	1 (0.0)	-	-	-	-	1 (0.0)
Infusion site erythema	2 (0.1)	7 (0.0)	11 (0.0)	-	-	2 (0.0)	-	22 (0.0)
Infusion site extravasation	4 (0.1)	4 (0.0)	21 (0.1)	-	-	1 (0.0)	-	30 (0.1)
Infusion site induration	1 (0.0)	-	1 (0.0)	-	-	-	-	2 (0.0)
Infusion site inflammation	-	-	1 (0.0)	-	-	-	-	1 (0.0)
Infusion site ischaemia	-	-	1 (0.0)	-	-	-	-	1 (0.0)
Infusion site oedema	2 (0.1)	4 (0.0)	3 (0.0)	-	-	-	-	9 (0.0)
Infusion site pain	-	3 (0.0)	12 (0.0)	-	-	1 (0.0)	-	16 (0.0)
Infusion site phlebitis	-	-	4 (0.0)	-	-	-	-	4 (0.0)
Infusion site pruritus	-	1 (0.0)	-	-	-	-	-	1 (0.0)
Infusion site rash	-	-	-	-	1 (0.2)	-	-	1 (0.0)
Infusion site reaction	-	2 (0.0)	3 (0.0)	-	-	-	-	5 (0.0)
Infusion site swelling	1 (0.0)	2 (0.0)	5 (0.0)	-	-	-	-	8 (0.0)
Infusion site ulcer	1 (0.0)	-	-	-	-	-	-	1 (0.0)
Infusion site urticaria	-	-	2 (0.0)	-	-	-	-	2 (0.0)
Infusion site vesicles	-	-	3 (0.0)	-	-	-	-	3 (0.0)
Infusion site warmth	1 (0.0)	-	1 (0.0)	-	-	-	-	2 (0.0)
Inguinal hernia	-	3 (0.0)	-	-	-	-	-	3 (0.0)
Inhibitory drug interaction	-	3 (0.0)	3 (0.0)	-	-	-	-	6 (0.0)
Initial insomnia	-	3 (0.0)	1 (0.0)	-	-	-	-	4 (0.0)

Injection related reaction	-	3 (0.0)	-	-	-	-	-	3 (0.0)
Injection site bruising	-	-	2 (0.0)	-	-	-	-	2 (0.0)
Injection site coldness	-	-	1 (0.0)	-	-	-	-	1 (0.0)
Injection site dysaesthesia	-	-	1 (0.0)	-	-	-	-	1 (0.0)
Injection site erythema	-	11 (0.1)	23 (0.1)	-	-	-	-	34 (0.1)
Injection site extravasation	1 (0.0)	1 (0.0)	11 (0.0)	-	-	-	-	13 (0.0)
Injection site haemorrhage	-	-	2 (0.0)	-	-	1 (0.0)	-	3 (0.0)
Injection site hypoaesthesia	-	1 (0.0)	3 (0.0)	-	-	-	-	4 (0.0)
Injection site induration	-	3 (0.0)	-	-	-	-	-	3 (0.0)
Injection site infection	-	1 (0.0)	-	-	-	-	-	1 (0.0)
Injection site inflammation	-	-	3 (0.0)	-	-	-	-	3 (0.0)
Injection site irritation	-	2 (0.0)	-	-	-	-	-	2 (0.0)
Injection site mass	-	2 (0.0)	1 (0.0)	-	-	-	-	3 (0.0)
Injection site necrosis	-	1 (0.0)	2 (0.0)	-	-	-	-	3 (0.0)
Injection site oedema	-	-	1 (0.0)	-	-	-	-	1 (0.0)
Injection site pain	-	19 (0.1)	62 (0.2)	-	-	3 (0.1)	1 (0.4)	85 (0.2)
Injection site phlebitis	-	-	2 (0.0)	-	-	-	-	2 (0.0)
Injection site rash	-	4 (0.0)	-	-	-	2 (0.0)	-	6 (0.0)
Injection site reaction	-	4 (0.0)	2 (0.0)	-	-	-	-	6 (0.0)
Injection site swelling	-	2 (0.0)	12 (0.0)	-	-	-	-	14 (0.0)
Injection site thrombosis	-	-	5 (0.0)	-	-	-	-	5 (0.0)
Injection site urticaria	-	9 (0.1)	1 (0.0)	-	-	-	-	10 (0.0)
Injection site vasculitis	-	1 (0.0)	-	-	-	-	-	1 (0.0)
Injection site vesicles	-	1 (0.0)	2 (0.0)	-	-	-	-	3 (0.0)
Injection site warmth	-	1 (0.0)	3 (0.0)	-	-	-	-	4 (0.0)
Injury	-	5 (0.0)	7 (0.0)	-	-	-	-	12 (0.0)
Injury associated with device	2 (0.1)	-	-	-	-	-	-	2 (0.0)
Insomnia	1 (0.0)	44 (0.3)	11 (0.0)	2 (0.6)	-	3 (0.1)	-	61 (0.1)
Inspiratory capacity abnormal	-	-	1 (0.0)	-	-	-	-	1 (0.0)
Inspiratory capacity decreased	-	-	1 (0.0)	-	-	-	-	1 (0.0)
Insurance issue	-	-	1 (0.0)	-	-	-	-	1 (0.0)
Intellectual disability	-	2 (0.0)	1 (0.0)	-	-	1 (0.0)	-	4 (0.0)

Intensive care	-	3 (0.0)	3 (0.0)	-	-	-	-	6 (0.0)
Intensive care unit acquired weakness	2 (0.1)	-	2 (0.0)	-	-	9 (0.2)	-	13 (0.0)
Intensive care unit delirium	-	-	-	-	-	1 (0.0)	-	1 (0.0)
Intention tremor	-	-	2 (0.0)	-	-	-	-	2 (0.0)
Intentional overdose	2 (0.1)	44 (0.3)	20 (0.1)	1 (0.3)	1 (0.2)	4 (0.1)	-	72 (0.1)
Intentional product misuse	1 (0.0)	70 (0.5)	18 (0.1)	-	-	11 (0.2)	-	100 (0.2)
Intentional product misuse to child	-	1 (0.0)	1 (0.0)	-	-	-	-	2 (0.0)
Intentional product use issue	12 (0.3)	39 (0.3)	15 (0.1)	-	-	7 (0.1)	-	73 (0.1)
Intentional self-injury	-	3 (0.0)	-	-	-	-	-	3 (0.0)
Intentional underdose	-	2 (0.0)	-	-	-	1 (0.0)	-	3 (0.0)
Intercepted product administration error	-	-	1 (0.0)	-	-	-	-	1 (0.0)
Interleukin level increased	-	-	4 (0.0)	-	-	-	-	4 (0.0)
Interleukin-2 receptor increased	-	-	-	-	-	1 (0.0)	-	1 (0.0)
Intermenstrual bleeding	-	-	1 (0.0)	-	-	-	-	1 (0.0)
Intermittent claudication	-	1 (0.0)	1 (0.0)	-	-	-	-	2 (0.0)
Intermittent explosive disorder	-	1 (0.0)	-	-	-	-	-	1 (0.0)
International normalised ratio increased	-	2 (0.0)	10 (0.0)	-	-	-	-	12 (0.0)
Interruption of aortic arch	-	-	-	-	1 (0.2)	-	-	1 (0.0)
Interstitial lung disease	1 (0.0)	4 (0.0)	6 (0.0)	-	2 (0.4)	-	-	13 (0.0)
Intervertebral disc degeneration	-	1 (0.0)	-	-	-	-	-	1 (0.0)
Intervertebral disc protrusion	-	2 (0.0)	2 (0.0)	-	-	-	-	4 (0.0)
Intestinal atresia	-	-	-	-	-	2 (0.0)	-	2 (0.0)
Intestinal dilatation	2 (0.1)	-	1 (0.0)	-	-	-	-	3 (0.0)
Intestinal infarction	-	1 (0.0)	2 (0.0)	-	-	1 (0.0)	-	4 (0.0)
Intestinal ischaemia	-	3 (0.0)	2 (0.0)	-	-	7 (0.1)	-	12 (0.0)
Intestinal obstruction	-	1 (0.0)	2 (0.0)	-	-	-	-	3 (0.0)
Intestinal perforation	-	-	-	-	-	5 (0.1)	-	5 (0.0)
Intestinal pseudo-obstruction	7 (0.2)	-	-	-	-	-	-	7 (0.0)
Intestinal stenosis	-	3 (0.0)	-	-	-	-	-	3 (0.0)
Intra-abdominal pressure increased	-	-	-	-	-	4 (0.1)	-	4 (0.0)
Intracranial pressure increased	-	5 (0.0)	5 (0.0)	-	-	3 (0.1)	-	13 (0.0)
Intraocular pressure increased	-	1 (0.0)	2 (0.0)	-	-	-	-	3 (0.0)

Intraventricular haemorrhage	3 (0.1)	-	1 (0.0)	-	-	-	-	4 (0.0)
Intraventricular haemorrhage neonatal	-	-	1 (0.0)	-	-	-	-	1 (0.0)
Iris atrophy	-	1 (0.0)	-	-	-	-	-	1 (0.0)
Iron deficiency	-	-	-	-	-	1 (0.0)	-	1 (0.0)
Iron deficiency anaemia	-	3 (0.0)	-	-	-	-	-	3 (0.0)
Irregular breathing	1 (0.0)	4 (0.0)	9 (0.0)	-	-	-	-	14 (0.0)
Irregular sleep wake rhythm disorder	-	1 (0.0)	-	-	-	-	-	1 (0.0)
Irritability	5 (0.1)	14 (0.1)	3 (0.0)	2 (0.6)	-	2 (0.0)	-	26 (0.1)
Ischaemia	1 (0.0)	3 (0.0)	1 (0.0)	-	-	-	-	5 (0.0)
Ischaemic cerebral infarction	-	-	1 (0.0)	-	-	-	-	1 (0.0)
Ischaemic stroke	1 (0.0)	1 (0.0)	6 (0.0)	1 (0.3)	-	-	-	9 (0.0)
Jaundice	1 (0.0)	6 (0.0)	19 (0.1)	-	-	3 (0.1)	-	29 (0.1)
Jaundice cholestatic	-	-	2 (0.0)	-	-	1 (0.0)	-	3 (0.0)
Jaundice neonatal	-	-	1 (0.0)	-	-	-	-	1 (0.0)
Jaw disorder	1 (0.0)	-	2 (0.0)	-	-	-	-	3 (0.0)
Joint arthroplasty	-	-	1 (0.0)	-	-	-	-	1 (0.0)
Joint dislocation	-	-	4 (0.0)	-	-	1 (0.0)	-	5 (0.0)
Joint injury	-	1 (0.0)	-	-	-	-	-	1 (0.0)
Joint stiffness	-	1 (0.0)	5 (0.0)	-	-	-	-	6 (0.0)
Joint swelling	1 (0.0)	2 (0.0)	1 (0.0)	-	-	-	-	4 (0.0)
Judgement impaired	-	5 (0.0)	1 (0.0)	-	-	-	-	6 (0.0)
Jugular vein distension	-	-	1 (0.0)	-	-	-	-	1 (0.0)
Ketonuria	-	-	1 (0.0)	-	-	-	-	1 (0.0)
Ketosis	-	-	1 (0.0)	-	-	-	-	1 (0.0)
Kidney duplex	-	1 (0.0)	-	-	-	-	-	1 (0.0)
Kidney enlargement	-	-	2 (0.0)	-	-	-	-	2 (0.0)
Kidney transplant rejection	-	-	1 (0.0)	-	-	-	-	1 (0.0)
Klebsiella infection	-	2 (0.0)	3 (0.0)	-	-	2 (0.0)	-	7 (0.0)
Klebsiella sepsis	-	1 (0.0)	-	-	-	-	-	1 (0.0)
Knee arthroplasty	-	1 (0.0)	1 (0.0)	-	-	-	-	2 (0.0)
Knee operation	-	1 (0.0)	-	-	-	-	-	1 (0.0)
Kounis syndrome	-	7 (0.0)	11 (0.0)	-	-	1 (0.0)	-	19 (0.0)

Kyphosis	-	-	2 (0.0)	-	-	-	-	2 (0.0)
Labelled drug-drug interaction issue	-	1 (0.0)	-	-	-	-	-	1 (0.0)
Labelled drug-drug interaction medication error	-	5 (0.0)	4 (0.0)	-	-	-	-	9 (0.0)
Labile blood pressure	-	-	1 (0.0)	-	-	3 (0.1)	-	4 (0.0)
Laboratory test	1 (0.0)	-	-	-	-	-	-	1 (0.0)
Laboratory test abnormal	-	2 (0.0)	1 (0.0)	-	-	-	-	3 (0.0)
Laboratory test interference	-	-	2 (0.0)	-	-	-	-	2 (0.0)
Labyrinthitis	-	1 (0.0)	-	-	-	-	-	1 (0.0)
Lacrimal haemorrhage	-	-	1 (0.0)	-	-	-	-	1 (0.0)
Lacrimation increased	-	1 (0.0)	6 (0.0)	-	-	-	-	7 (0.0)
Lactate pyruvate ratio increased	-	-	2 (0.0)	-	-	-	-	2 (0.0)
Lactescent serum	-	-	1 (0.0)	-	-	-	-	1 (0.0)
Lactic acidosis	2 (0.1)	11 (0.1)	57 (0.2)	-	-	5 (0.1)	-	75 (0.2)
Lagophthalmos	-	1 (0.0)	-	-	-	-	-	1 (0.0)
Language disorder	-	3 (0.0)	4 (0.0)	-	-	-	-	7 (0.0)
Large intestinal stenosis	-	1 (0.0)	-	-	-	-	-	1 (0.0)
Large intestinal ulcer	-	1 (0.0)	-	-	-	-	-	1 (0.0)
Large intestine perforation	1 (0.0)	-	-	-	-	1 (0.0)	-	2 (0.0)
Large intestine polyp	-	-	4 (0.0)	-	-	-	-	4 (0.0)
Laryngeal discomfort	-	1 (0.0)	-	-	-	-	-	1 (0.0)
Laryngeal disorder	-	2 (0.0)	-	-	-	-	-	2 (0.0)
Laryngeal dyspnoea	-	1 (0.0)	2 (0.0)	-	-	-	-	3 (0.0)
Laryngeal obstruction	-	-	2 (0.0)	-	-	-	-	2 (0.0)
Laryngeal oedema	2 (0.1)	11 (0.1)	39 (0.2)	-	1 (0.2)	3 (0.1)	-	56 (0.1)
Laryngeal stenosis	-	1 (0.0)	-	-	-	-	-	1 (0.0)
Laryngitis	-	-	1 (0.0)	-	-	-	-	1 (0.0)
Laryngomalacia	-	-	1 (0.0)	2 (0.6)	-	-	-	3 (0.0)
Laryngospasm	2 (0.1)	10 (0.1)	59 (0.2)	1 (0.3)	1 (0.2)	12 (0.3)	1 (0.4)	86 (0.2)
Lateral medullary syndrome	-	1 (0.0)	-	-	-	-	-	1 (0.0)
Learning disability	-	1 (0.0)	-	-	-	-	-	1 (0.0)
Learning disorder	-	2 (0.0)	-	-	-	-	-	2 (0.0)
Left atrial dilatation	-	-	1 (0.0)	-	-	-	-	1 (0.0)

Left ventricle outflow tract obstruction	-	2 (0.0)	3 (0.0)	-	-	-	-	5 (0.0)
Left ventricular dilatation	-	1 (0.0)	-	-	-	-	-	1 (0.0)
Left ventricular dysfunction	-	2 (0.0)	7 (0.0)	1 (0.3)	-	2 (0.0)	-	12 (0.0)
Left ventricular failure	-	1 (0.0)	3 (0.0)	-	-	1 (0.0)	-	5 (0.0)
Left ventricular hypertrophy	-	3 (0.0)	1 (0.0)	-	-	3 (0.1)	-	7 (0.0)
Leg amputation	-	-	1 (0.0)	-	-	-	-	1 (0.0)
Lethargy	1 (0.0)	10 (0.1)	12 (0.0)	-	1 (0.2)	1 (0.0)	-	25 (0.1)
Leukaemia	1 (0.0)	-	-	-	-	-	-	1 (0.0)
Leukocytosis	2 (0.1)	9 (0.1)	21 (0.1)	-	-	4 (0.1)	-	36 (0.1)
Leukocyturia	-	-	1 (0.0)	-	-	-	-	1 (0.0)
Leukoencephalopathy	-	1 (0.0)	4 (0.0)	-	-	-	-	5 (0.0)
Leukopenia	1 (0.0)	5 (0.0)	9 (0.0)	-	1 (0.2)	1 (0.0)	-	17 (0.0)
Libido decreased	-	2 (0.0)	-	-	-	-	-	2 (0.0)
Lichen planus	-	-	3 (0.0)	-	-	-	-	3 (0.0)
Lichenoid keratosis	-	-	1 (0.0)	-	-	-	-	1 (0.0)
Life expectancy shortened	-	1 (0.0)	-	-	-	-	-	1 (0.0)
Life support	-	-	2 (0.0)	-	-	-	-	2 (0.0)
Limb amputation	-	-	1 (0.0)	-	-	-	-	1 (0.0)
Limb deformity	-	1 (0.0)	2 (0.0)	-	-	-	-	3 (0.0)
Limb discomfort	-	10 (0.1)	1 (0.0)	-	-	1 (0.0)	-	12 (0.0)
Limb injury	-	-	1 (0.0)	-	-	-	-	1 (0.0)
Limb mass	-	-	1 (0.0)	-	-	-	-	1 (0.0)
Limb operation	-	-	1 (0.0)	-	-	-	-	1 (0.0)
Lip discolouration	-	-	1 (0.0)	-	-	-	-	1 (0.0)
Lip disorder	-	-	1 (0.0)	-	-	-	-	1 (0.0)
Lip erosion	-	3 (0.0)	-	-	-	-	-	3 (0.0)
Lip oedema	1 (0.0)	3 (0.0)	13 (0.1)	-	-	3 (0.1)	-	20 (0.0)
Lip pain	-	-	1 (0.0)	-	-	-	-	1 (0.0)
Lip swelling	-	6 (0.0)	11 (0.0)	-	-	13 (0.3)	-	30 (0.1)
Lipase increased	-	-	6 (0.0)	-	-	2 (0.0)	-	8 (0.0)
Lipids abnormal	-	-	1 (0.0)	-	-	-	-	1 (0.0)
Lipids increased	-	-	2 (0.0)	-	-	-	-	2 (0.0)

Lipoedema	-	-	2 (0.0)	-	-	-	-	2 (0.0)
Liquid product physical issue	-	-	1 (0.0)	-	-	-	-	1 (0.0)
Lissencephaly	-	1 (0.0)	-	-	-	-	-	1 (0.0)
Listless	-	-	1 (0.0)	-	-	-	-	1 (0.0)
Live birth	-	1 (0.0)	26 (0.1)	-	1 (0.2)	1 (0.0)	-	29 (0.1)
Livedo reticularis	1 (0.0)	2 (0.0)	3 (0.0)	-	-	-	-	6 (0.0)
Liver disorder	3 (0.1)	8 (0.1)	18 (0.1)	-	-	1 (0.0)	5 (1.9)	35 (0.1)
Liver function test abnormal	1 (0.0)	4 (0.0)	13 (0.1)	-	-	2 (0.0)	-	20 (0.0)
Liver function test increased	-	4 (0.0)	5 (0.0)	-	-	1 (0.0)	-	10 (0.0)
Liver injury	-	3 (0.0)	36 (0.1)	1 (0.3)	-	5 (0.1)	-	45 (0.1)
Lividity	-	-	-	-	-	1 (0.0)	-	1 (0.0)
Local anaesthetic systemic toxicity	3 (0.1)	1 (0.0)	2 (0.0)	-	-	-	-	6 (0.0)
Local reaction	-	1 (0.0)	2 (0.0)	-	-	-	-	3 (0.0)
Localised oedema	-	1 (0.0)	8 (0.0)	-	-	1 (0.0)	-	10 (0.0)
Locked-in syndrome	-	-	1 (0.0)	-	-	-	-	1 (0.0)
Logorrhoea	-	3 (0.0)	3 (0.0)	-	-	1 (0.0)	-	7 (0.0)
Long QT syndrome	1 (0.0)	-	4 (0.0)	1 (0.3)	-	2 (0.0)	-	8 (0.0)
Loss of consciousness	16 (0.4)	105 (0.7)	60 (0.2)	3 (0.9)	-	11 (0.2)	1 (0.4)	196 (0.4)
Loss of control of legs	-	-	-	-	-	1 (0.0)	-	1 (0.0)
Loss of libido	-	1 (0.0)	1 (0.0)	-	-	-	-	2 (0.0)
Loss of personal independence in daily activities	-	1 (0.0)	2 (0.0)	-	-	1 (0.0)	-	4 (0.0)
Low birth weight baby	-	10 (0.1)	21 (0.1)	-	3 (0.6)	6 (0.1)	-	40 (0.1)
Low cardiac output syndrome	-	-	1 (0.0)	-	-	-	-	1 (0.0)
Lower limb fracture	-	1 (0.0)	-	-	-	-	-	1 (0.0)
Lower respiratory tract infection	-	2 (0.0)	-	-	-	1 (0.0)	-	3 (0.0)
Lung abscess	1 (0.0)	-	-	-	-	-	-	1 (0.0)
Lung assist device therapy	-	-	1 (0.0)	-	-	-	-	1 (0.0)
Lung consolidation	-	-	1 (0.0)	-	-	-	-	1 (0.0)
Lung disorder	-	5 (0.0)	6 (0.0)	-	-	-	-	11 (0.0)
Lung hyperinflation	-	2 (0.0)	-	-	-	-	-	2 (0.0)
Lung infiltration	-	3 (0.0)	3 (0.0)	-	-	1 (0.0)	-	7 (0.0)
Lymphadenopathy	-	5 (0.0)	1 (0.0)	-	-	1 (0.0)	-	7 (0.0)

Lymphangitis	-	-	1 (0.0)	-	-	-	-	1 (0.0)
Lymphocyte count decreased	-	1 (0.0)	-	-	-	-	-	1 (0.0)
Lymphocyte percentage increased	3 (0.1)	-	-	-	-	-	-	3 (0.0)
Lymphocyte transformation test positive	-	-	1 (0.0)	-	-	-	-	1 (0.0)
Lymphocytic infiltration	-	2 (0.0)	1 (0.0)	-	-	-	-	3 (0.0)
Lymphocytosis	-	3 (0.0)	-	-	-	-	-	3 (0.0)
Lymphoedema	-	1 (0.0)	2 (0.0)	-	-	-	-	3 (0.0)
Lymphoma	-	1 (0.0)	-	-	-	-	-	1 (0.0)
Lymphopenia	-	-	2 (0.0)	-	-	1 (0.0)	-	3 (0.0)
Macrocephaly	-	1 (0.0)	-	-	-	-	-	1 (0.0)
Macroglossia	-	-	5 (0.0)	-	-	-	-	5 (0.0)
Macular degeneration	-	15 (0.1)	-	-	-	-	-	15 (0.0)
Macule	-	-	1 (0.0)	-	-	-	-	1 (0.0)
Madarosis	-	-	2 (0.0)	-	-	-	-	2 (0.0)
Magnesium deficiency	-	-	1 (0.0)	-	-	-	-	1 (0.0)
Magnetic resonance imaging head abnormal	-	2 (0.0)	3 (0.0)	-	-	-	-	5 (0.0)
Malabsorption	1 (0.0)	2 (0.0)	-	-	-	5 (0.1)	-	8 (0.0)
Malaise	-	61 (0.4)	25 (0.1)	-	-	5 (0.1)	-	91 (0.2)
Malignant hypertension	-	-	1 (0.0)	-	-	1 (0.0)	-	2 (0.0)
Malignant neoplasm progression	-	5 (0.0)	-	-	-	-	-	5 (0.0)
Mallory-Weiss syndrome	-	-	2 (0.0)	-	-	-	-	2 (0.0)
Malnutrition	-	1 (0.0)	-	-	-	-	-	1 (0.0)
Mania	1 (0.0)	1 (0.0)	3 (0.0)	-	-	1 (0.0)	1 (0.4)	7 (0.0)
Marrow hyperplasia	-	-	-	-	-	1 (0.0)	-	1 (0.0)
Mass	-	2 (0.0)	-	-	-	-	-	2 (0.0)
Mast cell activation syndrome	-	-	1 (0.0)	-	-	-	-	1 (0.0)
Mastication disorder	-	1 (0.0)	-	-	-	-	-	1 (0.0)
Mastocytosis	-	-	1 (0.0)	-	-	-	-	1 (0.0)
Mastoid disorder	1 (0.0)	-	-	-	-	-	-	1 (0.0)
Maternal exposure before pregnancy	-	-	2 (0.0)	-	-	1 (0.0)	-	3 (0.0)
Maternal exposure during delivery	1 (0.0)	2 (0.0)	11 (0.0)	-	3 (0.6)	4 (0.1)	-	21 (0.0)
Maternal exposure during pregnancy	4 (0.1)	29 (0.2)	59 (0.2)	1 (0.3)	2 (0.4)	27 (0.6)	2 (0.8)	124 (0.3)

Maternal exposure timing unspecified	-	1 (0.0)	10 (0.0)	-	-	-	-	11 (0.0)
Mean arterial pressure	-	-	-	-	-	1 (0.0)	-	1 (0.0)
Mean arterial pressure decreased	3 (0.1)	-	10 (0.0)	-	-	1 (0.0)	-	14 (0.0)
Mean arterial pressure increased	2 (0.1)	-	-	-	-	-	-	2 (0.0)
Mechanical ventilation	-	4 (0.0)	6 (0.0)	-	-	1 (0.0)	-	11 (0.0)
Mechanical ventilation complication	-	-	4 (0.0)	-	-	3 (0.1)	-	7 (0.0)
Meconium aspiration syndrome	-	1 (0.0)	-	-	-	-	-	1 (0.0)
Meconium in amniotic fluid	-	-	1 (0.0)	-	-	-	-	1 (0.0)
Mediastinal haematoma	1 (0.0)	-	-	-	-	-	-	1 (0.0)
Mediastinitis	-	1 (0.0)	-	-	-	-	-	1 (0.0)
Medical device change	-	1 (0.0)	-	-	-	-	-	1 (0.0)
Medical device site extravasation	-	-	1 (0.0)	-	-	-	-	1 (0.0)
Medical procedure	-	-	1 (0.0)	-	-	-	-	1 (0.0)
Medication error	1 (0.0)	52 (0.3)	33 (0.1)	-	-	-	-	86 (0.2)
Medullary thyroid cancer	-	-	-	-	-	1 (0.0)	-	1 (0.0)
Melaena	2 (0.1)	1 (0.0)	2 (0.0)	-	-	-	-	5 (0.0)
MELAS syndrome	-	-	1 (0.0)	-	-	1 (0.0)	-	2 (0.0)
Memory impairment	-	21 (0.1)	15 (0.1)	-	-	3 (0.1)	-	39 (0.1)
Meniere's disease	-	1 (0.0)	-	-	-	-	-	1 (0.0)
Meningism	-	-	2 (0.0)	-	-	-	-	2 (0.0)
Meningitis aseptic	-	1 (0.0)	1 (0.0)	-	-	-	-	2 (0.0)
Meningitis bacterial	-	1 (0.0)	1 (0.0)	-	1 (0.2)	-	-	3 (0.0)
Meningitis viral	-	-	1 (0.0)	-	-	-	-	1 (0.0)
Meningomyelocele	-	-	2 (0.0)	-	-	-	-	2 (0.0)
Meniscus injury	-	1 (0.0)	-	-	-	-	-	1 (0.0)
Menometrorrhagia	-	-	1 (0.0)	-	-	-	-	1 (0.0)
Menopausal symptoms	-	-	1 (0.0)	-	-	-	-	1 (0.0)
Mental disorder	-	7 (0.0)	4 (0.0)	-	-	1 (0.0)	-	12 (0.0)
Mental impairment	-	10 (0.1)	4 (0.0)	-	-	-	-	14 (0.0)
Mental status changes	3 (0.1)	11 (0.1)	13 (0.1)	1 (0.3)	-	3 (0.1)	-	31 (0.1)
Mental status changes postoperative	-	-	1 (0.0)	-	-	-	-	1 (0.0)
Mesenteric artery embolism	-	1 (0.0)	-	-	-	-	-	1 (0.0)

Mesenteric haemorrhage	-	1 (0.0)	-	-	-	-	-	1 (0.0)
Mesenteric traction syndrome	-	1 (0.0)	-	-	-	-	-	1 (0.0)
Metabolic acidosis	4 (0.1)	20 (0.1)	130 (0.5)	-	-	17 (0.4)	-	171 (0.3)
Metabolic alkalosis	-	-	1 (0.0)	-	-	-	-	1 (0.0)
Metabolic disorder	-	1 (0.0)	4 (0.0)	-	-	-	-	5 (0.0)
Metabolic encephalopathy	-	3 (0.0)	1 (0.0)	-	-	-	1 (0.4)	5 (0.0)
Metabolic surgery	-	1 (0.0)	-	-	-	-	-	1 (0.0)
Metamorphopsia	-	1 (0.0)	-	-	-	-	-	1 (0.0)
Metastases to bone	-	2 (0.0)	-	-	-	-	-	2 (0.0)
Metastases to liver	-	2 (0.0)	-	-	-	-	-	2 (0.0)
Metastases to meninges	-	1 (0.0)	-	-	-	-	-	1 (0.0)
Metastasis	-	3 (0.0)	1 (0.0)	-	-	-	-	4 (0.0)
Metastatic neoplasm	-	2 (0.0)	-	-	-	-	-	2 (0.0)
Methaemoglobinaemia	-	2 (0.0)	5 (0.0)	-	-	-	-	7 (0.0)
Microangiopathy	-	-	1 (0.0)	-	-	-	-	1 (0.0)
Microcephaly	-	2 (0.0)	4 (0.0)	-	-	1 (0.0)	-	7 (0.0)
Microcytic anaemia	-	-	1 (0.0)	-	-	-	-	1 (0.0)
Micturition disorder	-	-	1 (0.0)	-	-	-	-	1 (0.0)
Micturition urgency	1 (0.0)	1 (0.0)	1 (0.0)	-	-	-	-	3 (0.0)
Middle cerebral artery stroke	-	-	1 (0.0)	-	-	-	-	1 (0.0)
Middle insomnia	-	-	1 (0.0)	-	-	-	-	1 (0.0)
Migraine	-	3 (0.0)	6 (0.0)	-	-	-	-	9 (0.0)
Miosis	6 (0.2)	19 (0.1)	7 (0.0)	-	-	1 (0.0)	-	33 (0.1)
Mitochondrial cytopathy	-	-	4 (0.0)	-	-	-	-	4 (0.0)
Mitochondrial DNA mutation	-	1 (0.0)	-	-	-	3 (0.1)	-	4 (0.0)
Mitochondrial enzyme deficiency	-	-	1 (0.0)	-	-	-	-	1 (0.0)
Mitochondrial myopathy	-	-	1 (0.0)	-	-	-	-	1 (0.0)
Mitochondrial myopathy acquired	-	-	1 (0.0)	-	-	-	-	1 (0.0)
Mitochondrial toxicity	-	-	4 (0.0)	-	-	-	-	4 (0.0)
Mitral valve calcification	-	-	2 (0.0)	-	-	-	-	2 (0.0)
Mitral valve disease	-	-	1 (0.0)	-	-	1 (0.0)	-	2 (0.0)
Mitral valve disease mixed	-	-	1 (0.0)	-	-	-	-	1 (0.0)

Mitral valve incompetence	-	-	5 (0.0)	-	-	2 (0.0)	-	7 (0.0)
Mitral valve stenosis	-	-	2 (0.0)	-	-	-	-	2 (0.0)
Mixed liver injury	-	4 (0.0)	11 (0.0)	-	-	2 (0.0)	-	17 (0.0)
Moaning	-	-	-	-	-	2 (0.0)	-	2 (0.0)
Mobility decreased	-	1 (0.0)	-	1 (0.3)	-	1 (0.0)	-	3 (0.0)
Mononucleosis syndrome	-	4 (0.0)	-	-	-	-	-	4 (0.0)
Monoparesis	-	2 (0.0)	1 (0.0)	-	-	-	-	3 (0.0)
Monoplegia	-	1 (0.0)	5 (0.0)	-	-	-	-	6 (0.0)
Mood altered	-	4 (0.0)	1 (0.0)	-	-	-	-	5 (0.0)
Motor developmental delay	-	1 (0.0)	-	-	-	-	-	1 (0.0)
Motor dysfunction	-	1 (0.0)	5 (0.0)	2 (0.6)	1 (0.2)	1 (0.0)	-	10 (0.0)
Mouth haemorrhage	-	2 (0.0)	2 (0.0)	-	-	1 (0.0)	-	5 (0.0)
Mouth swelling	-	-	1 (0.0)	-	-	-	-	1 (0.0)
Mouth ulceration	-	4 (0.0)	-	-	-	-	-	4 (0.0)
Movement disorder	1 (0.0)	15 (0.1)	7 (0.0)	1 (0.3)	1 (0.2)	1 (0.0)	-	26 (0.1)
Mucocutaneous rash	-	-	3 (0.0)	-	-	-	-	3 (0.0)
Mucosal discolouration	-	-	1 (0.0)	-	-	-	-	1 (0.0)
Mucosal disorder	-	1 (0.0)	1 (0.0)	-	-	-	-	2 (0.0)
Mucosal dryness	-	-	-	-	1 (0.2)	-	-	1 (0.0)
Mucosal erosion	-	4 (0.0)	-	-	-	-	-	4 (0.0)
Mucosal hyperaemia	-	1 (0.0)	-	-	-	-	-	1 (0.0)
Mucosal inflammation	-	2 (0.0)	-	-	-	-	-	2 (0.0)
Mucous stools	-	2 (0.0)	-	-	-	-	-	2 (0.0)
Multi-organ disorder	-	1 (0.0)	-	-	-	-	-	1 (0.0)
Multiple allergies	-	2 (0.0)	-	-	-	-	-	2 (0.0)
Multiple injuries	-	1 (0.0)	-	-	-	-	-	1 (0.0)
Multiple organ dysfunction syndrome	10 (0.3)	30 (0.2)	91 (0.4)	1 (0.3)	5 (1.0)	16 (0.3)	1 (0.4)	154 (0.3)
Multiple sclerosis	-	-	-	-	-	2 (0.0)	1 (0.4)	3 (0.0)
Multiple sclerosis relapse	-	-	2 (0.0)	-	-	1 (0.0)	-	3 (0.0)
Multiple system atrophy	-	-	2 (0.0)	-	-	-	-	2 (0.0)
Multiple use of single-use product	-	-	21 (0.1)	-	-	-	-	21 (0.0)
Multiple-drug resistance	-	42 (0.3)	6 (0.0)	-	-	23 (0.5)	-	71 (0.1)

Multisystem inflammatory syndrome in children	-	1 (0.0)	-	-	-	-	-	1 (0.0)
Mumps	-	4 (0.0)	-	-	-	-	-	4 (0.0)
Munchausen's syndrome	-	1 (0.0)	-	-	-	-	-	1 (0.0)
Muscle atrophy	-	2 (0.0)	1 (0.0)	-	-	-	-	3 (0.0)
Muscle contractions involuntary	-	3 (0.0)	8 (0.0)	-	1 (0.2)	-	-	12 (0.0)
Muscle contracture	-	-	4 (0.0)	-	-	2 (0.0)	-	6 (0.0)
Muscle disorder	1 (0.0)	1 (0.0)	1 (0.0)	-	-	1 (0.0)	-	4 (0.0)
Muscle fatigue	-	-	1 (0.0)	-	-	-	-	1 (0.0)
Muscle injury	-	1 (0.0)	1 (0.0)	-	-	-	-	2 (0.0)
Muscle necrosis	-	-	4 (0.0)	-	-	-	-	4 (0.0)
Muscle relaxant therapy	-	-	-	-	-	1 (0.0)	-	1 (0.0)
Muscle rigidity	1 (0.0)	9 (0.1)	23 (0.1)	-	-	14 (0.3)	1 (0.4)	48 (0.1)
Muscle spasms	2 (0.1)	14 (0.1)	39 (0.2)	1 (0.3)	-	8 (0.2)	-	64 (0.1)
Muscle spasticity	2 (0.1)	3 (0.0)	5 (0.0)	-	-	1 (0.0)	-	11 (0.0)
Muscle strength abnormal	-	-	1 (0.0)	-	-	-	-	1 (0.0)
Muscle swelling	-	-	1 (0.0)	-	-	-	-	1 (0.0)
Muscle tightness	-	5 (0.0)	5 (0.0)	-	-	-	1 (0.4)	11 (0.0)
Muscle tone disorder	-	-	1 (0.0)	-	-	-	-	1 (0.0)
Muscle twitching	-	13 (0.1)	32 (0.1)	-	-	6 (0.1)	-	51 (0.1)
Muscular weakness	1 (0.0)	17 (0.1)	40 (0.2)	2 (0.6)	-	2 (0.0)	-	62 (0.1)
Musculoskeletal complication associated with device	-	1 (0.0)	-	-	-	-	-	1 (0.0)
Musculoskeletal discomfort	-	1 (0.0)	-	-	-	-	-	1 (0.0)
Musculoskeletal disorder	-	-	1 (0.0)	-	-	-	-	1 (0.0)
Musculoskeletal pain	-	-	1 (0.0)	-	-	1 (0.0)	-	2 (0.0)
Musculoskeletal stiffness	2 (0.1)	9 (0.1)	19 (0.1)	-	-	1 (0.0)	-	31 (0.1)
Mutism	1 (0.0)	1 (0.0)	6 (0.0)	-	-	1 (0.0)	1 (0.4)	10 (0.0)
Myalgia	-	18 (0.1)	62 (0.2)	-	-	2 (0.0)	-	82 (0.2)
Myasthenia gravis	1 (0.0)	-	2 (0.0)	-	-	-	-	3 (0.0)
Myasthenia gravis crisis	-	1 (0.0)	1 (0.0)	-	-	-	-	2 (0.0)
Myasthenic syndrome	-	-	2 (0.0)	-	-	-	-	2 (0.0)
Mycobacterium tuberculosis complex test positive	-	-	3 (0.0)	-	-	-	-	3 (0.0)
Mydriasis	4 (0.1)	13 (0.1)	19 (0.1)	-	-	7 (0.1)	-	43 (0.1)

Myelitis	-	-	1 (0.0)	-	-	-	-	1 (0.0)
Myelitis transverse	-	-	1 (0.0)	-	-	-	-	1 (0.0)
Myelodysplastic syndrome	-	1 (0.0)	-	-	-	-	-	1 (0.0)
Myelopathy	-	-	3 (0.0)	-	-	-	-	3 (0.0)
Myelosuppression	-	3 (0.0)	-	-	-	-	-	3 (0.0)
Myocardial bridging	-	1 (0.0)	-	-	-	-	-	1 (0.0)
Myocardial depression	-	-	3 (0.0)	-	-	1 (0.0)	-	4 (0.0)
Myocardial haemorrhage	-	-	1 (0.0)	-	-	-	-	1 (0.0)
Myocardial infarction	3 (0.1)	16 (0.1)	25 (0.1)	-	-	-	-	44 (0.1)
Myocardial injury	-	-	2 (0.0)	-	-	-	-	2 (0.0)
Myocardial ischaemia	4 (0.1)	2 (0.0)	11 (0.0)	-	-	6 (0.1)	-	23 (0.0)
Myocardial necrosis	-	1 (0.0)	-	-	-	-	-	1 (0.0)
Myocardial necrosis marker increased	-	1 (0.0)	5 (0.0)	-	-	1 (0.0)	-	7 (0.0)
Myocardial oedema	-	-	2 (0.0)	-	-	-	-	2 (0.0)
Myocardial stunning	-	-	2 (0.0)	-	-	-	-	2 (0.0)
Myocarditis	-	3 (0.0)	13 (0.1)	-	-	-	-	16 (0.0)
Myoclonic epilepsy	-	4 (0.0)	9 (0.0)	-	-	-	-	13 (0.0)
Myoclonus	2 (0.1)	43 (0.3)	76 (0.3)	1 (0.3)	3 (0.6)	16 (0.3)	3 (1.1)	144 (0.3)
Myoglobin blood increased	2 (0.1)	-	12 (0.0)	-	1 (0.2)	1 (0.0)	-	16 (0.0)
Myoglobin urine present	-	-	2 (0.0)	-	-	2 (0.0)	-	4 (0.0)
Myoglobinaemia	-	1 (0.0)	5 (0.0)	-	-	-	-	6 (0.0)
Myoglobinuria	-	1 (0.0)	5 (0.0)	-	-	-	-	6 (0.0)
Myokymia	-	-	-	-	-	1 (0.0)	-	1 (0.0)
Myopathy	-	1 (0.0)	8 (0.0)	-	-	-	-	9 (0.0)
Myopathy toxic	-	-	3 (0.0)	-	-	-	-	3 (0.0)
Myopia	-	1 (0.0)	-	-	-	-	-	1 (0.0)
Myositis	-	-	1 (0.0)	-	-	-	-	1 (0.0)
Myotonia	-	-	-	-	-	1 (0.0)	-	1 (0.0)
Myotonic dystrophy	-	-	-	-	-	1 (0.0)	-	1 (0.0)
Myxoedema	-	-	1 (0.0)	-	-	-	-	1 (0.0)
Nail disorder	-	-	1 (0.0)	-	-	-	-	1 (0.0)
Narcissistic personality disorder	-	-	1 (0.0)	-	-	-	-	1 (0.0)

Nasal congestion	-	7 (0.0)	1 (0.0)	-	-	-	-	8 (0.0)
Nasal discomfort	-	2 (0.0)	-	-	-	-	-	2 (0.0)
Nasal injury	-	-	1 (0.0)	-	-	-	-	1 (0.0)
Nasal oedema	-	1 (0.0)	1 (0.0)	-	-	1 (0.0)	-	3 (0.0)
Nasal operation	-	-	2 (0.0)	-	-	-	-	2 (0.0)
Nasal septum perforation	-	1 (0.0)	-	-	-	-	-	1 (0.0)
Nasogastric output high	1 (0.0)	-	-	-	-	-	-	1 (0.0)
Nasopharyngitis	-	3 (0.0)	2 (0.0)	-	-	-	-	5 (0.0)
Natural killer cell activity abnormal	-	1 (0.0)	-	-	-	-	-	1 (0.0)
Nausea	16 (0.4)	123 (0.8)	226 (0.9)	1 (0.3)	1 (0.2)	26 (0.6)	5 (1.9)	398 (0.8)
Near death experience	-	1 (0.0)	1 (0.0)	-	-	-	-	2 (0.0)
Neck pain	-	4 (0.0)	8 (0.0)	-	-	1 (0.0)	-	13 (0.0)
Necrosis	1 (0.0)	4 (0.0)	4 (0.0)	-	-	1 (0.0)	-	10 (0.0)
Necrosis ischaemic	-	1 (0.0)	-	-	-	4 (0.1)	-	5 (0.0)
Necrotising colitis	-	1 (0.0)	-	-	-	-	-	1 (0.0)
Necrotising fasciitis	-	-	1 (0.0)	-	-	-	-	1 (0.0)
Necrotising myositis	-	-	1 (0.0)	-	-	-	-	1 (0.0)
Negative pressure pulmonary oedema	-	2 (0.0)	10 (0.0)	-	3 (0.6)	5 (0.1)	-	20 (0.0)
Negative thoughts	-	3 (0.0)	-	-	-	-	-	3 (0.0)
Neonatal anuria	-	1 (0.0)	-	-	-	-	-	1 (0.0)
Neonatal asphyxia	-	2 (0.0)	8 (0.0)	-	-	4 (0.1)	-	14 (0.0)
Neonatal disorder	-	1 (0.0)	-	-	-	-	-	1 (0.0)
Neonatal hypotension	-	1 (0.0)	-	-	-	2 (0.0)	-	3 (0.0)
Neonatal hypoxia	9 (0.2)	2 (0.0)	3 (0.0)	-	-	-	-	14 (0.0)
Neonatal oversedation	-	1 (0.0)	2 (0.0)	-	-	1 (0.0)	-	4 (0.0)
Neonatal respiratory acidosis	-	-	2 (0.0)	-	-	1 (0.0)	-	3 (0.0)
Neonatal respiratory depression	2 (0.1)	4 (0.0)	6 (0.0)	-	-	7 (0.1)	-	19 (0.0)
Neonatal respiratory distress	-	1 (0.0)	-	-	-	1 (0.0)	-	2 (0.0)
Neonatal respiratory distress syndrome	-	2 (0.0)	1 (0.0)	-	1 (0.2)	-	-	4 (0.0)
Neonatal respiratory failure	-	2 (0.0)	1 (0.0)	-	-	-	-	3 (0.0)
Neonatal seizure	-	7 (0.0)	1 (0.0)	-	-	-	-	8 (0.0)
Neoplasm malignant	-	1 (0.0)	1 (0.0)	-	-	-	-	2 (0.0)

Neoplasm progression	-	1 (0.0)	-	-	-	-	-	1 (0.0)
Neoplasm recurrence	1 (0.0)	-	-	-	-	-	-	1 (0.0)
Nephrogenic anaemia	-	-	2 (0.0)	-	-	-	-	2 (0.0)
Nephrogenic diabetes insipidus	4 (0.1)	2 (0.0)	-	-	-	-	-	6 (0.0)
Nephrolithiasis	-	7 (0.0)	3 (0.0)	-	-	1 (0.0)	-	11 (0.0)
Nephropathy	-	-	4 (0.0)	-	-	1 (0.0)	-	5 (0.0)
Nephropathy toxic	-	1 (0.0)	4 (0.0)	-	-	-	-	5 (0.0)
Nephrosclerosis	-	1 (0.0)	-	-	-	-	-	1 (0.0)
Nerve block	-	1 (0.0)	-	-	-	-	-	1 (0.0)
Nerve conduction studies abnormal	1 (0.0)	-	-	-	1 (0.2)	-	-	2 (0.0)
Nerve injury	-	2 (0.0)	1 (0.0)	-	-	1 (0.0)	-	4 (0.0)
Nerve stimulation test abnormal	-	-	3 (0.0)	-	-	-	-	3 (0.0)
Nervous system disorder	1 (0.0)	12 (0.1)	11 (0.0)	-	-	6 (0.1)	-	30 (0.1)
Nervousness	1 (0.0)	2 (0.0)	4 (0.0)	-	-	-	-	7 (0.0)
Neuralgia	1 (0.0)	2 (0.0)	1 (0.0)	-	-	-	-	4 (0.0)
Neuritis cranial	-	1 (0.0)	-	-	-	-	-	1 (0.0)
Neurodevelopmental disorder	-	1 (0.0)	-	-	-	-	-	1 (0.0)
Neurogenic bladder	-	-	1 (0.0)	-	-	-	-	1 (0.0)
Neurogenic shock	-	-	2 (0.0)	-	-	-	-	2 (0.0)
Neuroleptic malignant syndrome	8 (0.2)	29 (0.2)	20 (0.1)	3 (0.9)	7 (1.4)	7 (0.1)	1 (0.4)	75 (0.2)
Neurological decompensation	-	3 (0.0)	4 (0.0)	-	-	-	-	7 (0.0)
Neurological symptom	-	1 (0.0)	25 (0.1)	-	-	4 (0.1)	-	30 (0.1)
Neuromuscular block prolonged	-	2 (0.0)	16 (0.1)	-	-	1 (0.0)	-	19 (0.0)
Neuromuscular blockade	-	-	6 (0.0)	-	-	1 (0.0)	-	7 (0.0)
Neuromuscular blocking therapy	-	1 (0.0)	-	-	-	-	-	1 (0.0)
Neuromuscular toxicity	-	1 (0.0)	-	-	-	-	-	1 (0.0)
Neuromyopathy	-	1 (0.0)	-	-	-	3 (0.1)	-	4 (0.0)
Neuropathy peripheral	1 (0.0)	4 (0.0)	1 (0.0)	-	-	-	-	6 (0.0)
Neurosis	-	1 (0.0)	-	-	-	-	-	1 (0.0)
Neurotoxicity	-	9 (0.1)	4 (0.0)	-	-	-	-	13 (0.0)
Neutropenia	-	7 (0.0)	7 (0.0)	-	-	11 (0.2)	-	25 (0.1)
Neutropenic sepsis	-	1 (0.0)	-	-	-	-	-	1 (0.0)

Neutrophil count abnormal	-	-	-	1 (0.3)	-	-	-	1 (0.0)
Neutrophil count decreased	1 (0.0)	-	-	-	-	-	-	1 (0.0)
Neutrophil count increased	-	1 (0.0)	-	-	-	-	-	1 (0.0)
Neutrophil percentage decreased	1 (0.0)	-	-	-	-	-	-	1 (0.0)
Neutrophilia	-	1 (0.0)	1 (0.0)	-	-	-	-	2 (0.0)
New onset refractory status epilepticus	-	-	-	-	-	4 (0.1)	-	4 (0.0)
Night sweats	-	2 (0.0)	3 (0.0)	-	-	-	-	5 (0.0)
Nightmare	-	17 (0.1)	1 (0.0)	-	-	-	-	18 (0.0)
Nikolsky's sign	-	1 (0.0)	-	-	-	1 (0.0)	-	2 (0.0)
No adverse event	-	39 (0.3)	8 (0.0)	-	1 (0.2)	1 (0.0)	-	49 (0.1)
No reaction on previous exposure to drug	-	-	2 (0.0)	-	-	-	-	2 (0.0)
Nocturia	-	1 (0.0)	1 (0.0)	-	-	-	-	2 (0.0)
Nodal arrhythmia	2 (0.1)	-	2 (0.0)	-	-	-	-	4 (0.0)
Nodal rhythm	3 (0.1)	2 (0.0)	8 (0.0)	-	1 (0.2)	1 (0.0)	-	15 (0.0)
Nodule	-	2 (0.0)	-	-	-	-	-	2 (0.0)
Non-cardiogenic pulmonary oedema	-	1 (0.0)	4 (0.0)	-	-	1 (0.0)	-	6 (0.0)
Non-consummation	-	2 (0.0)	-	-	-	-	-	2 (0.0)
Non-pitting oedema	-	1 (0.0)	-	-	-	-	-	1 (0.0)
Nonreassuring foetal heart rate pattern	-	-	3 (0.0)	-	-	-	-	3 (0.0)
Noonan syndrome	-	-	-	-	-	1 (0.0)	-	1 (0.0)
Normal newborn	-	1 (0.0)	9 (0.0)	-	1 (0.2)	-	-	11 (0.0)
Nosocomial infection	-	1 (0.0)	1 (0.0)	-	-	-	-	2 (0.0)
N-terminal prohormone brain natriuretic peptide increased	-	-	1 (0.0)	-	-	-	-	1 (0.0)
Nuchal rigidity	-	-	2 (0.0)	-	-	-	-	2 (0.0)
Nystagmus	-	4 (0.0)	5 (0.0)	-	-	4 (0.1)	-	13 (0.0)
Obesity	-	1 (0.0)	1 (0.0)	-	-	1 (0.0)	-	3 (0.0)
Obsessive-compulsive disorder	-	1 (0.0)	-	-	-	-	-	1 (0.0)
Obsessive-compulsive personality disorder	-	1 (0.0)	-	-	-	-	-	1 (0.0)
Obstruction	-	1 (0.0)	1 (0.0)	-	-	-	-	2 (0.0)
Obstructive airways disorder	3 (0.1)	18 (0.1)	40 (0.2)	-	-	9 (0.2)	-	70 (0.1)
Obstructive sleep apnoea syndrome	-	2 (0.0)	-	-	-	-	-	2 (0.0)
Occupational exposure to product	-	-	8 (0.0)	-	-	-	-	8 (0.0)

Ocular discomfort	-	-	1 (0.0)	-	-	-	-	1 (0.0)
Ocular hyperaemia	-	1 (0.0)	1 (0.0)	-	-	-	-	2 (0.0)
Ocular icterus	-	-	1 (0.0)	-	-	-	-	1 (0.0)
Oculogyric crisis	-	3 (0.0)	10 (0.0)	-	-	1 (0.0)	-	14 (0.0)
Odynophagia	-	7 (0.0)	2 (0.0)	-	-	-	-	9 (0.0)
Oedema	2 (0.1)	19 (0.1)	27 (0.1)	-	-	15 (0.3)	-	63 (0.1)
Oedema mouth	-	1 (0.0)	4 (0.0)	-	-	-	-	5 (0.0)
Oedema mucosal	-	-	1 (0.0)	-	-	2 (0.0)	-	3 (0.0)
Oedema peripheral	-	12 (0.1)	26 (0.1)	-	-	5 (0.1)	-	43 (0.1)
Oedematous pancreatitis	-	-	2 (0.0)	-	-	-	-	2 (0.0)
Oesophageal carcinoma	-	-	1 (0.0)	-	-	-	-	1 (0.0)
Oesophageal dilatation	-	1 (0.0)	-	-	-	-	-	1 (0.0)
Oesophageal disorder	-	-	1 (0.0)	-	-	-	-	1 (0.0)
Oesophageal oedema	-	-	1 (0.0)	-	-	-	-	1 (0.0)
Oesophageal pain	-	-	1 (0.0)	-	-	-	-	1 (0.0)
Oesophageal spasm	-	-	1 (0.0)	-	-	-	-	1 (0.0)
Oesophagitis	-	-	1 (0.0)	-	-	-	-	1 (0.0)
Off label use	125 (3.3)	345 (2.3)	163 (0.7)	4 (1.2)	11 (2.2)	65 (1.4)	1 (0.4)	714 (1.4)
Oligohydramnios	-	-	2 (0.0)	-	-	2 (0.0)	-	4 (0.0)
Oliguria	1 (0.0)	4 (0.0)	16 (0.1)	1 (0.3)	-	3 (0.1)	-	25 (0.1)
Onychoclasis	-	-	2 (0.0)	-	-	-	-	2 (0.0)
Open angle glaucoma	-	-	-	-	-	1 (0.0)	-	1 (0.0)
Ophthalmoplegia	1 (0.0)	1 (0.0)	4 (0.0)	-	-	-	-	6 (0.0)
Opisthotonus	-	4 (0.0)	20 (0.1)	-	-	1 (0.0)	-	25 (0.1)
Opsoclonus myoclonus	-	1 (0.0)	1 (0.0)	-	-	-	-	2 (0.0)
Optic ischaemic neuropathy	-	-	1 (0.0)	-	-	-	-	1 (0.0)
Optic nerve disorder	-	-	1 (0.0)	-	-	-	-	1 (0.0)
Optic neuritis	-	-	2 (0.0)	-	-	-	-	2 (0.0)
Optic neuropathy	-	-	1 (0.0)	-	-	1 (0.0)	-	2 (0.0)
Oral administration complication	-	1 (0.0)	-	-	-	-	-	1 (0.0)
Oral blood blister	-	1 (0.0)	-	-	-	-	-	1 (0.0)
Oral candidiasis	-	14 (0.1)	-	-	-	-	-	14 (0.0)

Oral discharge	-	-	2 (0.0)	1 (0.3)	-	-	-	3 (0.0)
Oral disorder	-	1 (0.0)	-	-	-	-	-	1 (0.0)
Oral dysaesthesia	-	-	1 (0.0)	-	-	-	-	1 (0.0)
Oral mucosa erosion	-	1 (0.0)	1 (0.0)	-	-	-	-	2 (0.0)
Oral mucosal eruption	-	1 (0.0)	-	-	-	-	-	1 (0.0)
Oral pain	-	1 (0.0)	-	-	-	-	-	1 (0.0)
Oral pruritus	-	1 (0.0)	-	-	-	-	-	1 (0.0)
Orbital oedema	-	-	1 (0.0)	-	-	-	-	1 (0.0)
Organ failure	-	2 (0.0)	-	-	-	-	-	2 (0.0)
Oromandibular dystonia	1 (0.0)	-	5 (0.0)	-	-	-	1 (0.4)	7 (0.0)
Oropharyngeal discomfort	-	2 (0.0)	3 (0.0)	-	-	-	-	5 (0.0)
Oropharyngeal oedema	1 (0.0)	-	-	-	-	-	-	1 (0.0)
Oropharyngeal pain	-	4 (0.0)	7 (0.0)	-	1 (0.2)	-	-	12 (0.0)
Oropharyngeal spasm	-	-	1 (0.0)	-	-	-	-	1 (0.0)
Oropharyngeal swelling	-	-	1 (0.0)	-	-	-	-	1 (0.0)
Orthopnoea	-	1 (0.0)	1 (0.0)	-	-	1 (0.0)	-	3 (0.0)
Orthostatic hypotension	1 (0.0)	1 (0.0)	1 (0.0)	-	-	2 (0.0)	-	5 (0.0)
Osmotic demyelination syndrome	-	1 (0.0)	-	-	-	-	-	1 (0.0)
Osteoarthritis	-	3 (0.0)	2 (0.0)	-	-	-	-	5 (0.0)
Osteoporosis	-	1 (0.0)	-	-	-	-	-	1 (0.0)
Otorrhoea	-	-	-	-	-	1 (0.0)	-	1 (0.0)
Ovarian cyst	-	2 (0.0)	-	-	-	1 (0.0)	-	3 (0.0)
Overdose	35 (0.9)	136 (0.9)	79 (0.3)	6 (1.8)	4 (0.8)	16 (0.3)	1 (0.4)	277 (0.6)
Overweight	-	2 (0.0)	-	-	-	-	-	2 (0.0)
Oxidative stress	-	1 (0.0)	-	-	-	-	-	1 (0.0)
Oxygen consumption decreased	-	3 (0.0)	2 (0.0)	-	-	-	-	5 (0.0)
Oxygen saturation	-	1 (0.0)	-	-	-	-	-	1 (0.0)
Oxygen saturation abnormal	-	5 (0.0)	7 (0.0)	-	-	2 (0.0)	-	14 (0.0)
Oxygen saturation decreased	26 (0.7)	189 (1.3)	249 (1.0)	6 (1.8)	1 (0.2)	38 (0.8)	2 (0.8)	511 (1.0)
Oxygen saturation immeasurable	-	4 (0.0)	2 (0.0)	-	-	-	-	6 (0.0)
Oxygen therapy	-	7 (0.0)	-	-	-	-	-	7 (0.0)
Pain	5 (0.1)	47 (0.3)	51 (0.2)	-	-	7 (0.1)	-	110 (0.2)

Pain in extremity	4 (0.1)	7 (0.0)	31 (0.1)	-	-	3 (0.1)	-	45 (0.1)
Pain in jaw	-	1 (0.0)	-	-	-	2 (0.0)	-	3 (0.0)
Pain of skin	-	-	2 (0.0)	-	-	-	-	2 (0.0)
Painful respiration	-	1 (0.0)	1 (0.0)	-	-	-	-	2 (0.0)
Palatal oedema	-	2 (0.0)	1 (0.0)	-	-	3 (0.1)	-	6 (0.0)
Pallor	1 (0.0)	23 (0.2)	10 (0.0)	-	-	3 (0.1)	-	37 (0.1)
Palmar erythema	-	-	1 (0.0)	-	-	-	-	1 (0.0)
Palmar-plantar erythrodysaesthesia syndrome	-	2 (0.0)	2 (0.0)	-	-	-	-	4 (0.0)
Palpitations	-	10 (0.1)	15 (0.1)	-	-	1 (0.0)	1 (0.4)	27 (0.1)
Pancreatic abscess	-	1 (0.0)	1 (0.0)	-	-	-	-	2 (0.0)
Pancreatic cyst	-	-	1 (0.0)	-	-	-	-	1 (0.0)
Pancreatic disorder	-	1 (0.0)	1 (0.0)	-	-	-	-	2 (0.0)
Pancreatic enzymes increased	-	1 (0.0)	2 (0.0)	-	-	-	-	3 (0.0)
Pancreatic haemorrhage	-	-	1 (0.0)	-	-	-	-	1 (0.0)
Pancreatitis	2 (0.1)	3 (0.0)	40 (0.2)	-	1 (0.2)	5 (0.1)	-	51 (0.1)
Pancreatitis acute	1 (0.0)	7 (0.0)	68 (0.3)	-	-	3 (0.1)	-	79 (0.2)
Pancreatitis haemorrhagic	-	-	3 (0.0)	-	-	1 (0.0)	-	4 (0.0)
Pancreatitis necrotising	-	1 (0.0)	8 (0.0)	-	-	1 (0.0)	-	10 (0.0)
Pancreatitis relapsing	-	-	2 (0.0)	-	-	-	-	2 (0.0)
Pancytopenia	-	6 (0.0)	7 (0.0)	-	-	1 (0.0)	-	14 (0.0)
Panic attack	-	4 (0.0)	4 (0.0)	-	-	1 (0.0)	-	9 (0.0)
Panic disorder	-	1 (0.0)	-	-	-	-	-	1 (0.0)
Panic reaction	-	2 (0.0)	-	-	-	1 (0.0)	-	3 (0.0)
Pantoea agglomerans infection	-	-	4 (0.0)	-	-	-	-	4 (0.0)
Papilloedema	-	7 (0.0)	1 (0.0)	-	-	-	-	8 (0.0)
Papilloma	-	1 (0.0)	-	-	-	-	-	1 (0.0)
Papule	-	1 (0.0)	7 (0.0)	-	-	1 (0.0)	-	9 (0.0)
Paradoxical drug reaction	2 (0.1)	50 (0.3)	3 (0.0)	-	-	2 (0.0)	-	57 (0.1)
Paradoxical pain	-	-	2 (0.0)	-	-	-	-	2 (0.0)
Paraesthesia	2 (0.1)	16 (0.1)	18 (0.1)	-	-	-	-	36 (0.1)
Paraesthesia oral	-	19 (0.1)	1 (0.0)	-	-	1 (0.0)	-	21 (0.0)
Paraganglion neoplasm	-	-	-	-	-	1 (0.0)	-	1 (0.0)

Parainfluenzae virus infection	-	-	-	-	-	1 (0.0)	-	1 (0.0)
Paralysis	-	9 (0.1)	19 (0.1)	-	-	-	-	28 (0.1)
Paranoia	-	5 (0.0)	1 (0.0)	-	-	1 (0.0)	-	7 (0.0)
Paraparesis	-	3 (0.0)	4 (0.0)	-	-	-	-	7 (0.0)
Paraplegia	-	2 (0.0)	6 (0.0)	-	-	-	-	8 (0.0)
Parasomnia	-	1 (0.0)	-	-	-	-	-	1 (0.0)
Paravalvular regurgitation	-	-	1 (0.0)	-	-	-	-	1 (0.0)
Paravenous drug administration	-	-	6 (0.0)	-	-	-	-	6 (0.0)
Paresis	-	2 (0.0)	5 (0.0)	-	-	1 (0.0)	-	8 (0.0)
Parkinsonism	-	3 (0.0)	-	-	-	-	-	3 (0.0)
Parkinsonism hyperpyrexia syndrome	-	-	1 (0.0)	-	-	-	-	1 (0.0)
Parkinson's disease	-	-	1 (0.0)	-	-	1 (0.0)	-	2 (0.0)
Parosmia	-	-	2 (0.0)	-	-	-	-	2 (0.0)
Parotid duct obstruction	-	-	1 (0.0)	-	-	-	-	1 (0.0)
Parotid gland enlargement	-	1 (0.0)	3 (0.0)	-	-	1 (0.0)	-	5 (0.0)
Parotitis	-	1 (0.0)	1 (0.0)	-	-	1 (0.0)	-	3 (0.0)
Paroxysmal atrioventricular block	-	-	2 (0.0)	-	-	-	-	2 (0.0)
Paroxysmal sympathetic hyperactivity	-	-	6 (0.0)	-	-	-	-	6 (0.0)
Partial seizures	1 (0.0)	28 (0.2)	4 (0.0)	-	-	8 (0.2)	1 (0.4)	42 (0.1)
Patella fracture	-	1 (0.0)	-	-	-	-	-	1 (0.0)
Patent ductus arteriosus	1 (0.0)	2 (0.0)	2 (0.0)	-	-	3 (0.1)	-	8 (0.0)
Pathogen resistance	-	2 (0.0)	1 (0.0)	-	-	-	-	3 (0.0)
Pathological fracture	-	-	-	-	-	2 (0.0)	-	2 (0.0)
Patient elopement	-	1 (0.0)	-	-	-	-	-	1 (0.0)
Patient uncooperative	1 (0.0)	1 (0.0)	1 (0.0)	-	-	-	-	3 (0.0)
PCO2	-	-	1 (0.0)	-	-	-	-	1 (0.0)
PCO2 increased	1 (0.0)	2 (0.0)	4 (0.0)	-	-	2 (0.0)	-	9 (0.0)
Peak expiratory flow rate decreased	-	-	2 (0.0)	-	-	-	-	2 (0.0)
Peau d'orange	-	-	1 (0.0)	-	-	-	-	1 (0.0)
Pelvic venous thrombosis	-	-	2 (0.0)	-	-	1 (0.0)	-	3 (0.0)
Penile erosion	-	-	1 (0.0)	-	-	-	-	1 (0.0)
Penile oedema	-	-	3 (0.0)	-	-	-	-	3 (0.0)

Penile swelling	-	-	2 (0.0)	-	-	-	-	2 (0.0)
Perforation	-	-	-	-	-	1 (0.0)	-	1 (0.0)
Performance status decreased	-	1 (0.0)	2 (0.0)	-	-	-	-	3 (0.0)
Pericardial effusion	-	-	12 (0.0)	-	-	5 (0.1)	-	17 (0.0)
Pericardial haemorrhage	-	-	1 (0.0)	-	-	1 (0.0)	-	2 (0.0)
Pericarditis uraemic	-	1 (0.0)	-	-	-	-	-	1 (0.0)
Pericoronitis	-	-	-	-	-	1 (0.0)	-	1 (0.0)
Periodic limb movement disorder	-	1 (0.0)	-	-	-	1 (0.0)	-	2 (0.0)
Periorbital haemorrhage	-	-	1 (0.0)	-	-	-	-	1 (0.0)
Periorbital oedema	-	5 (0.0)	8 (0.0)	-	-	2 (0.0)	-	15 (0.0)
Periorbital pain	-	-	1 (0.0)	-	-	-	-	1 (0.0)
Periorbital swelling	-	3 (0.0)	1 (0.0)	-	-	8 (0.2)	-	12 (0.0)
Peripancreatic fluid collection	-	-	2 (0.0)	-	-	-	-	2 (0.0)
Peripartum cardiomyopathy	-	-	2 (0.0)	-	-	-	-	2 (0.0)
Peripheral circulatory failure	1 (0.0)	-	1 (0.0)	-	-	-	-	2 (0.0)
Peripheral coldness	-	9 (0.1)	3 (0.0)	-	-	2 (0.0)	-	14 (0.0)
Peripheral ischaemia	-	3 (0.0)	4 (0.0)	-	-	-	-	7 (0.0)
Peripheral nerve decompression	-	-	1 (0.0)	-	-	-	-	1 (0.0)
Peripheral nerve injury	-	-	1 (0.0)	-	-	-	-	1 (0.0)
Peripheral nerve palsy	-	-	2 (0.0)	-	-	-	-	2 (0.0)
Peripheral paralysis	-	-	1 (0.0)	-	-	-	-	1 (0.0)
Peripheral swelling	1 (0.0)	9 (0.1)	9 (0.0)	-	-	1 (0.0)	-	20 (0.0)
Peripheral vascular disorder	-	-	1 (0.0)	-	-	-	-	1 (0.0)
Peripheral venous disease	-	1 (0.0)	-	-	-	-	-	1 (0.0)
Periprocedural myocardial infarction	-	-	1 (0.0)	-	-	-	-	1 (0.0)
Peritoneal haematoma	-	-	1 (0.0)	-	-	-	-	1 (0.0)
Peritoneal sarcoma	-	1 (0.0)	-	-	-	-	-	1 (0.0)
Peritonitis	-	1 (0.0)	1 (0.0)	-	-	-	-	2 (0.0)
Periventricular haemorrhage neonatal	-	-	-	-	-	1 (0.0)	-	1 (0.0)
Periventricular leukomalacia	-	1 (0.0)	-	-	-	-	-	1 (0.0)
Peroneal nerve palsy	-	-	3 (0.0)	-	-	-	-	3 (0.0)
Persecutory delusion	-	2 (0.0)	2 (0.0)	-	-	-	-	4 (0.0)

Perseveration	-	-	1 (0.0)	-	-	-	-	1 (0.0)
Personality change	-	-	2 (0.0)	-	-	1 (0.0)	-	3 (0.0)
Personality disorder	-	1 (0.0)	-	-	-	-	-	1 (0.0)
Petechiae	-	1 (0.0)	6 (0.0)	-	-	-	-	7 (0.0)
Petit mal epilepsy	-	5 (0.0)	1 (0.0)	-	-	-	-	6 (0.0)
Phaeochromocytoma	-	-	1 (0.0)	-	-	-	-	1 (0.0)
Phaeochromocytoma crisis	-	1 (0.0)	1 (0.0)	-	2 (0.4)	-	-	4 (0.0)
Pharyngeal cancer	-	-	1 (0.0)	-	-	-	-	1 (0.0)
Pharyngeal disorder	-	-	3 (0.0)	-	-	-	-	3 (0.0)
Pharyngeal haemorrhage	-	-	-	-	-	1 (0.0)	-	1 (0.0)
Pharyngeal hypoaesthesia	-	5 (0.0)	-	-	-	-	-	5 (0.0)
Pharyngeal oedema	-	6 (0.0)	4 (0.0)	-	1 (0.2)	1 (0.0)	-	12 (0.0)
Pharyngeal paraesthesia	-	5 (0.0)	-	-	-	-	-	5 (0.0)
Pharyngeal stenosis	-	1 (0.0)	-	-	-	-	-	1 (0.0)
Pharyngeal swelling	-	1 (0.0)	2 (0.0)	-	-	-	-	3 (0.0)
Phimosis	-	1 (0.0)	-	-	-	-	-	1 (0.0)
Phlebitis	1 (0.0)	3 (0.0)	13 (0.1)	-	-	3 (0.1)	-	20 (0.0)
Phlebitis superficial	-	1 (0.0)	-	-	-	-	-	1 (0.0)
Phobia	-	2 (0.0)	-	-	-	-	-	2 (0.0)
Photophobia	-	2 (0.0)	2 (0.0)	-	-	-	-	4 (0.0)
Photosensitivity reaction	-	2 (0.0)	-	-	-	-	-	2 (0.0)
Phrenic nerve paralysis	2 (0.1)	-	2 (0.0)	-	-	1 (0.0)	-	5 (0.0)
Physical assault	-	1 (0.0)	2 (0.0)	-	-	-	-	3 (0.0)
Physical disability	-	-	1 (0.0)	-	-	-	-	1 (0.0)
Physical product label issue	-	1 (0.0)	2 (0.0)	-	-	-	-	3 (0.0)
Pigmentation disorder	-	1 (0.0)	1 (0.0)	-	-	-	-	2 (0.0)
Piloerection	-	-	6 (0.0)	-	-	1 (0.0)	-	7 (0.0)
Pituitary haemorrhage	-	-	1 (0.0)	-	-	-	-	1 (0.0)
Pituitary tumour benign	-	-	2 (0.0)	-	-	-	-	2 (0.0)
Platelet count abnormal	-	1 (0.0)	-	-	-	-	-	1 (0.0)
Platelet count decreased	7 (0.2)	6 (0.0)	15 (0.1)	-	-	2 (0.0)	-	30 (0.1)
Platelet count increased	-	1 (0.0)	1 (0.0)	-	-	-	-	2 (0.0)

Pleocytosis	-	1 (0.0)	1 (0.0)	-	-	2 (0.0)	-	4 (0.0)
Pleural adhesion	-	1 (0.0)	-	-	-	-	-	1 (0.0)
Pleural effusion	1 (0.0)	7 (0.0)	25 (0.1)	-	2 (0.4)	4 (0.1)	-	39 (0.1)
Pleurisy	-	1 (0.0)	-	-	-	-	-	1 (0.0)
Pneumatosis	-	-	-	-	-	2 (0.0)	-	2 (0.0)
Pneumatosis intestinalis	-	1 (0.0)	-	-	-	-	-	1 (0.0)
Pneumocephalus	-	-	1 (0.0)	-	-	-	-	1 (0.0)
Pneumoconiosis	-	1 (0.0)	-	-	-	-	-	1 (0.0)
Pneumocystis jirovecii pneumonia	-	1 (0.0)	-	-	-	-	-	1 (0.0)
Pneumomediastinum	1 (0.0)	2 (0.0)	2 (0.0)	-	-	1 (0.0)	-	6 (0.0)
Pneumonia	10 (0.3)	42 (0.3)	30 (0.1)	1 (0.3)	3 (0.6)	11 (0.2)	2 (0.8)	99 (0.2)
Pneumonia acinetobacter	-	1 (0.0)	-	-	-	-	-	1 (0.0)
Pneumonia aspiration	4 (0.1)	11 (0.1)	17 (0.1)	-	1 (0.2)	6 (0.1)	-	39 (0.1)
Pneumonia escherichia	1 (0.0)	-	-	-	-	-	-	1 (0.0)
Pneumonia fungal	-	1 (0.0)	-	-	-	-	-	1 (0.0)
Pneumonia haemophilus	1 (0.0)	-	-	-	-	-	-	1 (0.0)
Pneumonia klebsiella	-	1 (0.0)	1 (0.0)	-	-	1 (0.0)	-	3 (0.0)
Pneumonia measles	-	-	1 (0.0)	-	-	-	-	1 (0.0)
Pneumonia pseudomonal	-	1 (0.0)	1 (0.0)	-	-	-	-	2 (0.0)
Pneumonia staphylococcal	-	1 (0.0)	2 (0.0)	-	1 (0.2)	3 (0.1)	-	7 (0.0)
Pneumonitis	-	6 (0.0)	1 (0.0)	-	-	-	-	7 (0.0)
Pneumonitis aspiration	-	2 (0.0)	4 (0.0)	-	-	1 (0.0)	-	7 (0.0)
Pneumopericardium	1 (0.0)	-	-	-	-	-	-	1 (0.0)
Pneumoperitoneum	-	2 (0.0)	2 (0.0)	-	-	-	-	4 (0.0)
Pneumothorax	1 (0.0)	8 (0.1)	17 (0.1)	-	-	10 (0.2)	-	36 (0.1)
PO2 decreased	1 (0.0)	2 (0.0)	2 (0.0)	-	-	1 (0.0)	-	6 (0.0)
PO2 increased	1 (0.0)	-	-	-	-	1 (0.0)	-	2 (0.0)
Poisoning	-	16 (0.1)	7 (0.0)	-	-	-	-	23 (0.0)
Poisoning deliberate	-	1 (0.0)	2 (0.0)	-	-	6 (0.1)	-	9 (0.0)
Pollakiuria	-	4 (0.0)	2 (0.0)	-	-	-	-	6 (0.0)
Polyarthritis	-	-	2 (0.0)	-	-	-	-	2 (0.0)
Polyhydramnios	-	-	1 (0.0)	-	-	-	-	1 (0.0)

Polymyalgia rheumatica	-	-	1 (0.0)	-	-	-	-	1 (0.0)
Polyneuropathy	2 (0.1)	3 (0.0)	5 (0.0)	-	-	3 (0.1)	-	13 (0.0)
Polyp	-	-	1 (0.0)	-	-	-	-	1 (0.0)
Polyuria	37 (1.0)	3 (0.0)	9 (0.0)	-	-	-	-	49 (0.1)
Poor feeding infant	1 (0.0)	2 (0.0)	-	2 (0.6)	-	-	-	5 (0.0)
Poor peripheral circulation	-	2 (0.0)	2 (0.0)	-	-	-	-	4 (0.0)
Poor quality product administered	-	1 (0.0)	8 (0.0)	-	-	-	-	9 (0.0)
Poor quality sleep	-	2 (0.0)	1 (0.0)	-	-	-	-	3 (0.0)
Porphyria	-	-	-	-	-	1 (0.0)	-	1 (0.0)
Porphyria acute	2 (0.1)	-	-	-	-	-	-	2 (0.0)
Portopulmonary hypertension	-	-	1 (0.0)	-	-	-	-	1 (0.0)
Post polio syndrome	-	-	1 (0.0)	-	-	-	-	1 (0.0)
Post procedural complication	1 (0.0)	-	10 (0.0)	-	-	4 (0.1)	1 (0.4)	16 (0.0)
Post procedural discomfort	-	-	1 (0.0)	-	-	-	-	1 (0.0)
Post procedural fever	-	-	2 (0.0)	-	-	-	-	2 (0.0)
Post procedural haematoma	-	-	2 (0.0)	-	-	-	-	2 (0.0)
Post procedural haemorrhage	-	2 (0.0)	10 (0.0)	-	-	4 (0.1)	-	16 (0.0)
Post procedural hypotension	-	1 (0.0)	1 (0.0)	-	-	2 (0.0)	-	4 (0.0)
Post procedural infection	-	-	3 (0.0)	-	-	-	-	3 (0.0)
Post procedural pneumonia	-	1 (0.0)	-	-	-	-	-	1 (0.0)
Post procedural stroke	-	2 (0.0)	1 (0.0)	-	-	-	-	3 (0.0)
Posterior reversible encephalopathy syndrome	1 (0.0)	5 (0.0)	6 (0.0)	1 (0.3)	-	5 (0.1)	-	18 (0.0)
Postictal paralysis	-	1 (0.0)	1 (0.0)	-	-	-	-	2 (0.0)
Postictal state	-	1 (0.0)	1 (0.0)	-	-	-	-	2 (0.0)
Post-injection delirium sedation syndrome	-	-	15 (0.1)	-	-	-	-	15 (0.0)
Postoperative delirium	22 (0.6)	6 (0.0)	29 (0.1)	-	1 (0.2)	3 (0.1)	1 (0.4)	62 (0.1)
Postoperative ileus	1 (0.0)	-	-	-	-	-	-	1 (0.0)
Postoperative renal failure	-	-	1 (0.0)	-	-	-	-	1 (0.0)
Postoperative respiratory distress	-	-	2 (0.0)	-	-	-	-	2 (0.0)
Postoperative respiratory failure	-	-	4 (0.0)	-	-	-	-	4 (0.0)
Postoperative thrombosis	-	-	1 (0.0)	-	-	-	-	1 (0.0)
Postoperative wound infection	-	1 (0.0)	1 (0.0)	-	-	-	-	2 (0.0)

Postpartum haemorrhage	1 (0.0)	-	8 (0.0)	-	-	-	-	9 (0.0)
Postresuscitation encephalopathy	-	1 (0.0)	1 (0.0)	2 (0.6)	-	-	-	4 (0.0)
Post-traumatic osteoporosis	-	1 (0.0)	-	-	-	-	-	1 (0.0)
Post-traumatic stress disorder	-	7 (0.0)	5 (0.0)	1 (0.3)	-	1 (0.0)	1 (0.4)	15 (0.0)
Postural tremor	-	-	-	-	-	1 (0.0)	-	1 (0.0)
Posture abnormal	-	2 (0.0)	5 (0.0)	-	-	1 (0.0)	-	8 (0.0)
Posturing	-	1 (0.0)	-	-	-	-	-	1 (0.0)
Potentiating drug interaction	-	4 (0.0)	10 (0.0)	-	1 (0.2)	4 (0.1)	6 (2.3)	25 (0.1)
Pre-eclampsia	-	2 (0.0)	4 (0.0)	-	1 (0.2)	1 (0.0)	-	8 (0.0)
Pregnancy	-	2 (0.0)	1 (0.0)	-	-	-	-	3 (0.0)
Pregnancy on oral contraceptive	-	2 (0.0)	-	-	-	-	-	2 (0.0)
Premature baby	-	14 (0.1)	46 (0.2)	-	5 (1.0)	21 (0.4)	2 (0.8)	88 (0.2)
Premature delivery	-	7 (0.0)	18 (0.1)	-	3 (0.6)	13 (0.3)	2 (0.8)	43 (0.1)
Premature labour	-	1 (0.0)	3 (0.0)	-	1 (0.2)	2 (0.0)	-	7 (0.0)
Premature recovery from anaesthesia	-	-	17 (0.1)	-	-	-	-	17 (0.0)
Premature separation of placenta	-	-	1 (0.0)	-	-	1 (0.0)	-	2 (0.0)
Prerenal failure	-	1 (0.0)	-	-	-	-	-	1 (0.0)
Prescribed overdose	2 (0.1)	12 (0.1)	7 (0.0)	1 (0.3)	-	-	-	22 (0.0)
Prescribed underdose	-	1 (0.0)	-	-	-	-	-	1 (0.0)
Prescription drug used without a prescription	2 (0.1)	3 (0.0)	-	-	-	-	-	5 (0.0)
Prescription form tampering	-	1 (0.0)	-	-	-	-	-	1 (0.0)
Presyncope	1 (0.0)	7 (0.0)	3 (0.0)	-	-	2 (0.0)	-	13 (0.0)
Preterm premature rupture of membranes	-	-	-	-	-	1 (0.0)	-	1 (0.0)
Priapism	-	1 (0.0)	17 (0.1)	-	-	1 (0.0)	-	19 (0.0)
Prinzmetal angina	7 (0.2)	2 (0.0)	14 (0.1)	-	-	-	-	23 (0.0)
Procalcitonin	-	-	1 (0.0)	-	-	-	-	1 (0.0)
Procalcitonin increased	-	1 (0.0)	5 (0.0)	-	-	-	-	6 (0.0)
Procedural anxiety	-	1 (0.0)	-	-	-	-	-	1 (0.0)
Procedural complication	-	1 (0.0)	7 (0.0)	-	1 (0.2)	4 (0.1)	-	13 (0.0)
Procedural haemorrhage	-	-	7 (0.0)	-	-	1 (0.0)	-	8 (0.0)
Procedural headache	-	-	2 (0.0)	-	-	-	-	2 (0.0)
Procedural hypertension	1 (0.0)	1 (0.0)	1 (0.0)	-	-	-	-	3 (0.0)

Procedural hypotension	1 (0.0)	3 (0.0)	40 (0.2)	-	-	3 (0.1)	-	47 (0.1)
Procedural nausea	-	2 (0.0)	21 (0.1)	-	-	4 (0.1)	1 (0.4)	28 (0.1)
Procedural pain	1 (0.0)	16 (0.1)	-	1 (0.3)	-	-	-	18 (0.0)
Procedural shock	-	-	17 (0.1)	-	-	-	-	17 (0.0)
Procedural vomiting	-	1 (0.0)	17 (0.1)	1 (0.3)	-	3 (0.1)	1 (0.4)	23 (0.0)
Proctalgia	-	3 (0.0)	-	-	-	-	-	3 (0.0)
Proctitis	-	16 (0.1)	-	-	-	-	-	16 (0.0)
Product administered to patient of inappropriate age	98 (2.6)	8 (0.1)	7 (0.0)	15 (4.5)	7 (1.4)	5 (0.1)	1 (0.4)	141 (0.3)
Product administration error	6 (0.2)	38 (0.3)	15 (0.1)	-	-	7 (0.1)	-	66 (0.1)
Product administration interrupted	-	1 (0.0)	1 (0.0)	-	-	-	-	2 (0.0)
Product appearance confusion	3 (0.1)	-	-	-	-	-	-	3 (0.0)
Product availability issue	-	4 (0.0)	1 (0.0)	-	-	-	-	5 (0.0)
Product closure removal difficult	-	1 (0.0)	-	-	-	-	-	1 (0.0)
Product colour issue	-	1 (0.0)	1 (0.0)	-	-	-	-	2 (0.0)
Product commingling	-	1 (0.0)	-	-	-	-	-	1 (0.0)
Product communication issue	-	1 (0.0)	1 (0.0)	-	-	-	-	2 (0.0)
Product complaint	4 (0.1)	3 (0.0)	12 (0.0)	-	-	2 (0.0)	-	21 (0.0)
Product confusion	-	3 (0.0)	-	-	-	-	-	3 (0.0)
Product container issue	-	-	2 (0.0)	-	-	-	-	2 (0.0)
Product container seal issue	-	1 (0.0)	-	-	-	-	-	1 (0.0)
Product contamination	-	1 (0.0)	10 (0.0)	-	-	-	-	11 (0.0)
Product contamination microbial	-	-	6 (0.0)	-	-	-	-	6 (0.0)
Product contamination physical	-	2 (0.0)	-	-	-	-	-	2 (0.0)
Product delivery mechanism issue	-	3 (0.0)	-	-	-	-	-	3 (0.0)
Product deposit	-	2 (0.0)	-	-	-	-	-	2 (0.0)
Product dispensing error	-	6 (0.0)	5 (0.0)	-	-	-	-	11 (0.0)
Product dispensing issue	-	1 (0.0)	1 (0.0)	-	-	-	-	2 (0.0)
Product dosage form confusion	1 (0.0)	-	-	-	-	-	-	1 (0.0)
Product dose confusion	-	1 (0.0)	-	-	-	-	-	1 (0.0)
Product dose omission in error	-	1 (0.0)	-	-	-	-	-	1 (0.0)
Product dose omission issue	-	8 (0.1)	3 (0.0)	-	-	-	-	11 (0.0)
Product formulation issue	-	-	3 (0.0)	-	-	-	-	3 (0.0)

Product impurity	-	4 (0.0)	1 (0.0)	-	-	-	-	5 (0.0)
Product label confusion	1 (0.0)	2 (0.0)	-	-	-	-	-	3 (0.0)
Product label issue	-	2 (0.0)	1 (0.0)	-	-	-	-	3 (0.0)
Product leakage	-	-	1 (0.0)	-	-	-	-	1 (0.0)
Product lot number issue	-	1 (0.0)	-	-	-	-	-	1 (0.0)
Product measured potency issue	-	4 (0.0)	9 (0.0)	-	-	-	-	13 (0.0)
Product monitoring error	1 (0.0)	1 (0.0)	1 (0.0)	-	-	-	-	3 (0.0)
Product name confusion	-	1 (0.0)	-	-	-	-	-	1 (0.0)
Product odour abnormal	-	1 (0.0)	2 (0.0)	-	-	-	-	3 (0.0)
Product package associated injury	-	3 (0.0)	2 (0.0)	-	-	-	-	5 (0.0)
Product packaging confusion	2 (0.1)	1 (0.0)	-	-	-	-	-	3 (0.0)
Product packaging issue	-	1 (0.0)	-	-	-	-	-	1 (0.0)
Product packaging quantity issue	-	1 (0.0)	-	-	-	-	-	1 (0.0)
Product physical issue	-	2 (0.0)	2 (0.0)	-	-	-	-	4 (0.0)
Product preparation error	2 (0.1)	6 (0.0)	4 (0.0)	-	-	-	-	12 (0.0)
Product preparation issue	1 (0.0)	2 (0.0)	14 (0.1)	-	-	-	-	17 (0.0)
Product prescribing error	2 (0.1)	15 (0.1)	4 (0.0)	-	-	-	-	21 (0.0)
Product prescribing issue	1 (0.0)	3 (0.0)	-	-	-	-	-	4 (0.0)
Product quality issue	1 (0.0)	13 (0.1)	41 (0.2)	-	-	4 (0.1)	-	59 (0.1)
Product residue present	-	2 (0.0)	-	-	-	-	-	2 (0.0)
Product selection error	1 (0.0)	3 (0.0)	-	-	-	-	-	4 (0.0)
Product shape issue	-	1 (0.0)	-	-	-	-	-	1 (0.0)
Product solubility abnormal	-	1 (0.0)	-	-	-	-	-	1 (0.0)
Product storage error	-	3 (0.0)	4 (0.0)	-	-	-	-	7 (0.0)
Product substitution issue	1 (0.0)	4 (0.0)	1 (0.0)	-	-	1 (0.0)	-	7 (0.0)
Product tampering	-	1 (0.0)	-	-	-	-	-	1 (0.0)
Product taste abnormal	-	3 (0.0)	1 (0.0)	-	-	-	-	4 (0.0)
Product temperature excursion issue	-	1 (0.0)	-	-	-	-	-	1 (0.0)
Product use complaint	-	3 (0.0)	1 (0.0)	-	-	-	-	4 (0.0)
Product use in unapproved indication	49 (1.3)	77 (0.5)	61 (0.2)	3 (0.9)	7 (1.4)	37 (0.8)	4 (1.5)	238 (0.5)
Product use in unapproved therapeutic environment	8 (0.2)	-	-	1 (0.3)	-	-	-	9 (0.0)
Product use issue	26 (0.7)	80 (0.5)	32 (0.1)	3 (0.9)	2 (0.4)	8 (0.2)	2 (0.8)	153 (0.3)

Product used for unknown indication	1 (0.0)	-	-	-	-	-	-	1 (0.0)
Productive cough	-	2 (0.0)	2 (0.0)	1 (0.3)	-	-	-	5 (0.0)
Propofol infusion syndrome	-	-	436 (1.7)	-	1 (0.2)	11 (0.2)	1 (0.4)	449 (0.9)
Prostate cancer	-	1 (0.0)	-	-	-	-	-	1 (0.0)
Protein total decreased	1 (0.0)	1 (0.0)	-	-	-	-	-	2 (0.0)
Protein urine present	-	1 (0.0)	1 (0.0)	-	-	-	-	2 (0.0)
Proteinuria	-	3 (0.0)	-	-	-	1 (0.0)	-	4 (0.0)
Prothrombin time prolonged	-	-	10 (0.0)	-	-	-	-	10 (0.0)
Prothrombin time shortened	-	1 (0.0)	1 (0.0)	-	-	-	-	2 (0.0)
Protrusion tongue	-	1 (0.0)	1 (0.0)	-	-	-	1 (0.4)	3 (0.0)
Pruritus	2 (0.1)	65 (0.4)	80 (0.3)	3 (0.9)	2 (0.4)	15 (0.3)	-	167 (0.3)
Pruritus allergic	-	1 (0.0)	-	-	-	-	-	1 (0.0)
Pseudocholinesterase deficiency	-	-	2 (0.0)	-	-	-	-	2 (0.0)
Pseudomembranous colitis	-	-	-	-	-	1 (0.0)	-	1 (0.0)
Pseudomonal sepsis	-	1 (0.0)	-	-	-	1 (0.0)	-	2 (0.0)
Pseudomonas infection	-	2 (0.0)	1 (0.0)	-	-	1 (0.0)	-	4 (0.0)
Pseudopolyp	-	1 (0.0)	-	-	-	-	-	1 (0.0)
Psychiatric symptom	-	2 (0.0)	4 (0.0)	-	-	-	-	6 (0.0)
Psychogenic seizure	-	1 (0.0)	1 (0.0)	-	-	1 (0.0)	-	3 (0.0)
Psychogenic tremor	-	-	1 (0.0)	-	-	-	-	1 (0.0)
Psychomotor hyperactivity	3 (0.1)	47 (0.3)	19 (0.1)	-	-	-	1 (0.4)	70 (0.1)
Psychomotor retardation	-	1 (0.0)	1 (0.0)	1 (0.3)	-	-	-	3 (0.0)
Psychomotor skills impaired	-	1 (0.0)	1 (0.0)	-	-	-	-	2 (0.0)
Psychotherapy	-	-	1 (0.0)	-	-	-	-	1 (0.0)
Psychotic behaviour	-	-	1 (0.0)	-	-	-	-	1 (0.0)
Psychotic disorder	1 (0.0)	26 (0.2)	6 (0.0)	-	-	-	-	33 (0.1)
Psychotic symptom	-	2 (0.0)	-	-	-	-	-	2 (0.0)
Pulmonary air leakage	1 (0.0)	-	-	-	-	1 (0.0)	-	2 (0.0)
Pulmonary alveolar haemorrhage	1 (0.0)	5 (0.0)	8 (0.0)	-	-	2 (0.0)	-	16 (0.0)
Pulmonary arterial hypertension	2 (0.1)	1 (0.0)	1 (0.0)	-	-	-	-	4 (0.0)
Pulmonary arterial pressure decreased	-	2 (0.0)	-	-	-	-	-	2 (0.0)
Pulmonary arterial pressure increased	-	-	1 (0.0)	-	-	-	-	1 (0.0)

Pulmonary artery dilatation	-	1 (0.0)	-	-	-	-	-	1 (0.0)
Pulmonary artery stenosis	-	1 (0.0)	-	-	-	-	-	1 (0.0)
Pulmonary congestion	1 (0.0)	5 (0.0)	3 (0.0)	-	-	-	-	9 (0.0)
Pulmonary embolism	2 (0.1)	9 (0.1)	19 (0.1)	-	-	2 (0.0)	1 (0.4)	33 (0.1)
Pulmonary fibrosis	-	1 (0.0)	1 (0.0)	-	-	-	-	2 (0.0)
Pulmonary function test abnormal	-	-	1 (0.0)	-	-	-	-	1 (0.0)
Pulmonary function test decreased	-	5 (0.0)	1 (0.0)	-	1 (0.2)	1 (0.0)	-	8 (0.0)
Pulmonary haemorrhage	-	1 (0.0)	10 (0.0)	-	-	2 (0.0)	-	13 (0.0)
Pulmonary haemorrhage neonatal	-	1 (0.0)	-	-	-	1 (0.0)	-	2 (0.0)
Pulmonary hypertension	-	2 (0.0)	4 (0.0)	1 (0.3)	-	-	-	7 (0.0)
Pulmonary hypertensive crisis	-	5 (0.0)	-	-	-	1 (0.0)	-	6 (0.0)
Pulmonary interstitial emphysema syndrome	-	1 (0.0)	-	-	-	-	-	1 (0.0)
Pulmonary mass	-	2 (0.0)	-	-	-	-	-	2 (0.0)
Pulmonary oedema	7 (0.2)	17 (0.1)	67 (0.3)	1 (0.3)	2 (0.4)	9 (0.2)	-	103 (0.2)
Pulmonary pain	-	-	-	-	-	1 (0.0)	-	1 (0.0)
Pulmonary physical examination abnormal	-	-	1 (0.0)	-	-	-	-	1 (0.0)
Pulmonary sequestration	-	-	-	-	-	1 (0.0)	-	1 (0.0)
Pulmonary thrombosis	-	-	3 (0.0)	-	-	-	-	3 (0.0)
Pulmonary toxicity	-	1 (0.0)	1 (0.0)	-	-	-	-	2 (0.0)
Pulmonary valve incompetence	-	-	1 (0.0)	-	-	-	-	1 (0.0)
Pulmonary valve stenosis	-	1 (0.0)	-	-	-	-	-	1 (0.0)
Pulmonary vascular disorder	-	1 (0.0)	-	-	-	-	-	1 (0.0)
Pulmonary vasculitis	-	1 (0.0)	-	-	-	-	-	1 (0.0)
Pulse abnormal	-	5 (0.0)	4 (0.0)	-	-	-	-	9 (0.0)
Pulse absent	-	8 (0.1)	15 (0.1)	-	-	4 (0.1)	-	27 (0.1)
Pulse pressure increased	-	-	1 (0.0)	-	-	-	-	1 (0.0)
Pulseless electrical activity	2 (0.1)	10 (0.1)	42 (0.2)	-	1 (0.2)	8 (0.2)	-	63 (0.1)
Puncture site haemorrhage	-	-	1 (0.0)	-	-	-	-	1 (0.0)
Pupil fixed	-	3 (0.0)	3 (0.0)	-	-	2 (0.0)	-	8 (0.0)
Pupillary disorder	-	1 (0.0)	1 (0.0)	-	-	-	-	2 (0.0)
Pupillary light reflex tests abnormal	1 (0.0)	2 (0.0)	-	-	-	-	-	3 (0.0)
Pupillary reflex impaired	-	7 (0.0)	4 (0.0)	-	-	2 (0.0)	-	13 (0.0)

Purpura	1 (0.0)	3 (0.0)	2 (0.0)	-	-	-	-	6 (0.0)
Purpura non-thrombocytopenic	-	2 (0.0)	-	-	-	-	-	2 (0.0)
Purulent pericarditis	-	1 (0.0)	-	-	-	-	-	1 (0.0)
Pustule	-	1 (0.0)	2 (0.0)	-	-	-	-	3 (0.0)
Pyelocaliectasis	-	1 (0.0)	-	-	-	-	-	1 (0.0)
Pyelonephritis	-	1 (0.0)	1 (0.0)	-	-	-	-	2 (0.0)
Pyelonephritis acute	-	-	1 (0.0)	-	-	-	-	1 (0.0)
Pyoderma gangrenosum	-	1 (0.0)	-	-	-	-	-	1 (0.0)
Pyogenic granuloma	-	-	-	-	-	1 (0.0)	-	1 (0.0)
Pyopneumothorax	-	-	1 (0.0)	-	-	-	-	1 (0.0)
Pyramidal tract syndrome	-	-	2 (0.0)	-	-	-	-	2 (0.0)
Pyrexia	148 (3.9)	100 (0.7)	298 (1.2)	3 (0.9)	15 (3.0)	26 (0.6)	1 (0.4)	591 (1.2)
Quadriparesis	1 (0.0)	3 (0.0)	2 (0.0)	-	-	2 (0.0)	-	8 (0.0)
Quadriplegia	1 (0.0)	10 (0.1)	7 (0.0)	-	-	-	-	18 (0.0)
Quality of life decreased	2 (0.1)	1 (0.0)	2 (0.0)	-	-	-	-	5 (0.0)
Radial pulse abnormal	-	-	1 (0.0)	-	-	-	-	1 (0.0)
Radial pulse decreased	-	1 (0.0)	-	-	-	-	-	1 (0.0)
Radiculopathy	1 (0.0)	14 (0.1)	-	-	-	-	-	15 (0.0)
Radius fracture	-	1 (0.0)	-	-	-	-	-	1 (0.0)
Rales	-	1 (0.0)	10 (0.0)	-	-	-	-	11 (0.0)
Rapid eye movements sleep abnormal	-	-	-	-	-	1 (0.0)	-	1 (0.0)
Rash	10 (0.3)	105 (0.7)	433 (1.7)	1 (0.3)	5 (1.0)	77 (1.6)	-	631 (1.3)
Rash erythematous	-	32 (0.2)	76 (0.3)	-	1 (0.2)	12 (0.3)	-	121 (0.2)
Rash macular	1 (0.0)	11 (0.1)	14 (0.1)	-	-	1 (0.0)	-	27 (0.1)
Rash maculo-papular	5 (0.1)	44 (0.3)	62 (0.2)	-	-	17 (0.4)	-	128 (0.3)
Rash morbilliform	2 (0.1)	3 (0.0)	6 (0.0)	-	-	-	-	11 (0.0)
Rash papular	-	5 (0.0)	17 (0.1)	-	1 (0.2)	2 (0.0)	-	25 (0.1)
Rash pruritic	1 (0.0)	9 (0.1)	8 (0.0)	-	-	-	-	18 (0.0)
Rash pustular	-	1 (0.0)	7 (0.0)	-	-	1 (0.0)	-	9 (0.0)
Rash vesicular	-	1 (0.0)	5 (0.0)	-	-	-	-	6 (0.0)
Rasmussen encephalitis	-	1 (0.0)	-	-	-	-	-	1 (0.0)
Reaction to excipient	-	1 (0.0)	3 (0.0)	-	-	1 (0.0)	-	5 (0.0)

Reaction to preservatives	-	-	1 (0.0)	-	-	-	-	1 (0.0)
Rebound effect	-	6 (0.0)	-	-	-	-	-	6 (0.0)
Rebound psychosis	1 (0.0)	-	1 (0.0)	-	-	-	-	2 (0.0)
Rebound tachycardia	1 (0.0)	-	-	-	-	-	-	1 (0.0)
Recalled product administered	-	-	2 (0.0)	-	-	-	-	2 (0.0)
Rectal haemorrhage	-	16 (0.1)	2 (0.0)	-	-	-	-	18 (0.0)
Recurrence of neuromuscular blockade	2 (0.1)	-	7 (0.0)	-	1 (0.2)	1 (0.0)	-	11 (0.0)
Red blood cell count decreased	3 (0.1)	2 (0.0)	1 (0.0)	-	-	-	-	6 (0.0)
Red blood cell transfusion	-	-	-	-	-	1 (0.0)	-	1 (0.0)
Reduced facial expression	-	2 (0.0)	1 (0.0)	-	-	-	-	3 (0.0)
Reexpansion pulmonary oedema	-	-	2 (0.0)	-	-	-	-	2 (0.0)
Reflexes abnormal	-	1 (0.0)	-	-	-	-	-	1 (0.0)
Reflux gastritis	-	-	1 (0.0)	-	-	-	-	1 (0.0)
Refusal of treatment by patient	1 (0.0)	2 (0.0)	-	-	-	-	-	3 (0.0)
Regurgitation	-	1 (0.0)	2 (0.0)	-	-	-	-	3 (0.0)
Renal disorder	-	3 (0.0)	8 (0.0)	-	-	-	-	11 (0.0)
Renal failure	2 (0.1)	18 (0.1)	43 (0.2)	-	1 (0.2)	5 (0.1)	2 (0.8)	71 (0.1)
Renal failure neonatal	-	1 (0.0)	-	-	-	-	-	1 (0.0)
Renal function test abnormal	-	-	4 (0.0)	-	-	-	-	4 (0.0)
Renal impairment	2 (0.1)	11 (0.1)	22 (0.1)	-	2 (0.4)	4 (0.1)	-	41 (0.1)
Renal infarct	-	2 (0.0)	-	-	-	-	-	2 (0.0)
Renal injury	-	-	1 (0.0)	-	-	-	-	1 (0.0)
Renal ischaemia	-	-	-	-	-	1 (0.0)	-	1 (0.0)
Renal oncocytoma	-	1 (0.0)	-	-	-	-	-	1 (0.0)
Renal pain	-	2 (0.0)	4 (0.0)	-	-	-	-	6 (0.0)
Renal tubular disorder	-	-	3 (0.0)	-	-	1 (0.0)	-	4 (0.0)
Renal tubular dysfunction	-	-	1 (0.0)	-	-	1 (0.0)	-	2 (0.0)
Renal tubular necrosis	-	1 (0.0)	11 (0.0)	-	-	-	-	12 (0.0)
Renin increased	-	-	8 (0.0)	-	-	-	-	8 (0.0)
Re-opening of ductus arteriosus	-	1 (0.0)	-	-	-	-	-	1 (0.0)
Reperfusion injury	-	-	-	-	-	1 (0.0)	-	1 (0.0)
Repetitive speech	-	1 (0.0)	-	-	-	-	-	1 (0.0)

Respiration abnormal	1 (0.0)	9 (0.1)	3 (0.0)	-	-	-	-	13 (0.0)
Respiratory acidosis	4 (0.1)	14 (0.1)	31 (0.1)	1 (0.3)	-	11 (0.2)	-	61 (0.1)
Respiratory alkalosis	-	1 (0.0)	2 (0.0)	-	-	1 (0.0)	1 (0.4)	5 (0.0)
Respiratory arrest	9 (0.2)	100 (0.7)	79 (0.3)	1 (0.3)	1 (0.2)	17 (0.4)	1 (0.4)	208 (0.4)
Respiratory depression	36 (0.9)	222 (1.5)	131 (0.5)	7 (2.1)	12 (2.4)	40 (0.9)	3 (1.1)	451 (0.9)
Respiratory disorder	2 (0.1)	20 (0.1)	19 (0.1)	1 (0.3)	-	4 (0.1)	1 (0.4)	47 (0.1)
Respiratory disorder neonatal	-	-	2 (0.0)	-	-	-	-	2 (0.0)
Respiratory distress	2 (0.1)	43 (0.3)	19 (0.1)	-	1 (0.2)	9 (0.2)	-	74 (0.1)
Respiratory failure	13 (0.3)	76 (0.5)	76 (0.3)	2 (0.6)	5 (1.0)	18 (0.4)	2 (0.8)	192 (0.4)
Respiratory fatigue	1 (0.0)	-	-	-	-	-	-	1 (0.0)
Respiratory gas exchange disorder	-	-	-	-	-	2 (0.0)	-	2 (0.0)
Respiratory muscle weakness	-	-	4 (0.0)	-	-	1 (0.0)	-	5 (0.0)
Respiratory paralysis	-	-	1 (0.0)	-	-	-	-	1 (0.0)
Respiratory rate decreased	6 (0.2)	14 (0.1)	4 (0.0)	-	-	3 (0.1)	-	27 (0.1)
Respiratory rate increased	1 (0.0)	9 (0.1)	5 (0.0)	-	-	-	-	15 (0.0)
Respiratory symptom	-	1 (0.0)	2 (0.0)	-	-	3 (0.1)	-	6 (0.0)
Respiratory syncytial virus infection	-	1 (0.0)	-	-	-	-	-	1 (0.0)
Respiratory therapy	-	-	1 (0.0)	-	-	-	-	1 (0.0)
Respiratory tract haemorrhage	-	-	1 (0.0)	-	-	-	-	1 (0.0)
Respiratory tract infection	-	2 (0.0)	-	-	-	-	-	2 (0.0)
Respiratory tract irritation	-	1 (0.0)	-	-	-	-	-	1 (0.0)
Respiratory tract oedema	-	-	4 (0.0)	-	-	-	-	4 (0.0)
Restless legs syndrome	3 (0.1)	-	3 (0.0)	-	1 (0.2)	-	-	7 (0.0)
Restlessness	7 (0.2)	46 (0.3)	39 (0.2)	6 (1.8)	-	4 (0.1)	2 (0.8)	104 (0.2)
Resuscitation	1 (0.0)	8 (0.1)	8 (0.0)	-	-	-	-	17 (0.0)
Retained products of conception	-	-	-	-	-	1 (0.0)	-	1 (0.0)
Retching	-	10 (0.1)	5 (0.0)	-	-	3 (0.1)	-	18 (0.0)
Retinal degeneration	-	-	1 (0.0)	-	-	-	-	1 (0.0)
Retinal haemorrhage	-	-	2 (0.0)	-	-	-	-	2 (0.0)
Retinal migraine	1 (0.0)	-	-	-	-	-	-	1 (0.0)
Retinopathy	-	-	1 (0.0)	-	-	-	-	1 (0.0)
Retinopathy haemorrhagic	-	-	3 (0.0)	-	-	-	-	3 (0.0)

Retrograde amnesia	1 (0.0)	7 (0.0)	6 (0.0)	-	-	3 (0.1)	-	17 (0.0)
Retroperitoneal haematoma	-	-	1 (0.0)	-	-	1 (0.0)	-	2 (0.0)
Retroperitoneal lymphadenopathy	-	-	1 (0.0)	-	-	1 (0.0)	-	2 (0.0)
Reversal of opiate activity	-	1 (0.0)	-	-	-	-	-	1 (0.0)
Reversal of sedation	-	2 (0.0)	-	-	-	-	-	2 (0.0)
Reversible airways obstruction	-	-	1 (0.0)	-	-	-	-	1 (0.0)
Reversible cerebral vasoconstriction syndrome	-	2 (0.0)	1 (0.0)	-	-	-	-	3 (0.0)
Reye's syndrome	-	-	1 (0.0)	-	-	-	-	1 (0.0)
Rhabdomyolysis	14 (0.4)	37 (0.2)	280 (1.1)	2 (0.6)	9 (1.8)	21 (0.5)	1 (0.4)	365 (0.7)
Rheumatoid arthritis	-	1 (0.0)	2 (0.0)	-	-	-	-	3 (0.0)
Rhinalgia	-	1 (0.0)	-	-	-	-	-	1 (0.0)
Rhinitis	-	1 (0.0)	2 (0.0)	-	-	-	-	3 (0.0)
Rhinitis allergic	-	1 (0.0)	-	-	-	-	-	1 (0.0)
Rhinorrhoea	-	2 (0.0)	7 (0.0)	-	-	-	-	9 (0.0)
Rhonchi	-	1 (0.0)	-	-	-	-	-	1 (0.0)
Rhythm idioventricular	2 (0.1)	-	1 (0.0)	-	-	-	-	3 (0.0)
Right ventricle outflow tract obstruction	-	1 (0.0)	1 (0.0)	-	-	-	-	2 (0.0)
Right ventricular dysfunction	-	-	4 (0.0)	-	-	2 (0.0)	-	6 (0.0)
Right ventricular enlargement	-	-	1 (0.0)	-	-	-	-	1 (0.0)
Right ventricular failure	-	-	3 (0.0)	-	-	-	-	3 (0.0)
Right ventricular hypertension	-	1 (0.0)	-	-	-	-	-	1 (0.0)
Right ventricular hypertrophy	-	-	1 (0.0)	-	-	-	-	1 (0.0)
Road traffic accident	1 (0.0)	6 (0.0)	3 (0.0)	-	-	-	-	10 (0.0)
Rubber sensitivity	-	-	1 (0.0)	-	-	-	-	1 (0.0)
Ruptured cerebral aneurysm	-	-	1 (0.0)	-	-	-	-	1 (0.0)
Salivary gland enlargement	-	-	1 (0.0)	-	-	-	-	1 (0.0)
Salivary hypersecretion	-	8 (0.1)	30 (0.1)	-	-	1 (0.0)	-	39 (0.1)
Salt craving	-	2 (0.0)	-	-	-	-	-	2 (0.0)
Scar	-	1 (0.0)	-	-	-	-	-	1 (0.0)
Scar pain	-	4 (0.0)	-	-	-	-	-	4 (0.0)
Schizencephaly	-	1 (0.0)	-	-	-	-	-	1 (0.0)
Schizoaffective disorder	-	4 (0.0)	-	-	-	-	-	4 (0.0)

Scintillating scotoma	1 (0.0)	-	-	-	-	-	-	1 (0.0)
Scoliosis	-	-	1 (0.0)	-	-	-	-	1 (0.0)
Scratch	-	-	2 (0.0)	-	-	-	-	2 (0.0)
Screaming	1 (0.0)	8 (0.1)	3 (0.0)	-	-	1 (0.0)	-	13 (0.0)
Seasonal allergy	-	1 (0.0)	-	-	-	-	-	1 (0.0)
Secretion discharge	-	3 (0.0)	2 (0.0)	-	-	-	-	5 (0.0)
Sedation	5 (0.1)	86 (0.6)	22 (0.1)	1 (0.3)	-	1 (0.0)	-	115 (0.2)
Sedation complication	34 (0.9)	96 (0.6)	18 (0.1)	5 (1.5)	3 (0.6)	6 (0.1)	1 (0.4)	163 (0.3)
Sedative therapy	-	2 (0.0)	-	-	-	-	-	2 (0.0)
Seizure	27 (0.7)	203 (1.4)	174 (0.7)	4 (1.2)	-	40 (0.9)	1 (0.4)	449 (0.9)
Seizure anoxic	-	-	1 (0.0)	-	-	-	-	1 (0.0)
Seizure cluster	-	6 (0.0)	-	-	-	1 (0.0)	-	7 (0.0)
Seizure like phenomena	-	2 (0.0)	13 (0.1)	-	-	1 (0.0)	9 (3.4)	25 (0.1)
Selective abortion	-	-	1 (0.0)	-	-	-	-	1 (0.0)
Self-injurious ideation	-	-	1 (0.0)	-	-	-	-	1 (0.0)
Self-medication	-	1 (0.0)	2 (0.0)	-	-	-	-	3 (0.0)
Sensation of foreign body	-	1 (0.0)	1 (0.0)	-	-	1 (0.0)	-	3 (0.0)
Sense of oppression	-	2 (0.0)	-	-	-	-	-	2 (0.0)
Sensitisation	-	1 (0.0)	-	-	-	-	-	1 (0.0)
Sensitive skin	-	1 (0.0)	-	-	-	1 (0.0)	-	2 (0.0)
Sensorimotor disorder	-	-	1 (0.0)	-	-	-	-	1 (0.0)
Sensory disturbance	-	3 (0.0)	2 (0.0)	-	-	-	-	5 (0.0)
Sensory level abnormal	-	-	2 (0.0)	-	-	2 (0.0)	-	4 (0.0)
Sensory loss	-	5 (0.0)	3 (0.0)	-	-	-	-	8 (0.0)
Sepsis	3 (0.1)	22 (0.1)	84 (0.3)	-	-	16 (0.3)	-	125 (0.3)
Sepsis syndrome	-	-	3 (0.0)	-	-	-	-	3 (0.0)
Septic shock	5 (0.1)	10 (0.1)	43 (0.2)	1 (0.3)	1 (0.2)	6 (0.1)	-	66 (0.1)
Septo-optic dysplasia	-	-	-	-	1 (0.2)	-	-	1 (0.0)
Serotonin syndrome	1 (0.0)	13 (0.1)	35 (0.1)	1 (0.3)	-	7 (0.1)	-	57 (0.1)
Serratia bacteraemia	-	-	2 (0.0)	-	-	-	-	2 (0.0)
Serratia infection	-	-	4 (0.0)	-	-	-	-	4 (0.0)
Serratia sepsis	-	-	2 (0.0)	-	-	-	-	2 (0.0)

Serum ferritin increased	-	1 (0.0)	-	-	-	1 (0.0)	-	2 (0.0)
Severe myoclonic epilepsy of infancy	-	2 (0.0)	-	-	-	-	-	2 (0.0)
Sex chromosome abnormality	-	-	1 (0.0)	-	-	-	-	1 (0.0)
Shock	12 (0.3)	14 (0.1)	76 (0.3)	1 (0.3)	-	30 (0.6)	-	133 (0.3)
Shock haemorrhagic	-	-	4 (0.0)	-	-	3 (0.1)	-	7 (0.0)
Shock symptom	-	-	3 (0.0)	-	-	1 (0.0)	-	4 (0.0)
Shoshin beriberi	-	2 (0.0)	-	-	-	-	-	2 (0.0)
Shunt infection	1 (0.0)	-	-	-	-	-	-	1 (0.0)
Shunt occlusion	1 (0.0)	-	-	-	-	-	-	1 (0.0)
Sialoadenitis	-	2 (0.0)	2 (0.0)	-	-	-	-	4 (0.0)
Similar reaction on previous exposure to drug	-	-	1 (0.0)	-	-	-	-	1 (0.0)
Simple partial seizures	-	2 (0.0)	-	-	-	-	-	2 (0.0)
Sinoatrial block	4 (0.1)	-	1 (0.0)	-	-	-	-	5 (0.0)
Sinus arrest	30 (0.8)	1 (0.0)	15 (0.1)	-	3 (0.6)	-	-	49 (0.1)
Sinus arrhythmia	-	1 (0.0)	3 (0.0)	-	-	-	-	4 (0.0)
Sinus bradycardia	14 (0.4)	6 (0.0)	23 (0.1)	1 (0.3)	2 (0.4)	4 (0.1)	1 (0.4)	51 (0.1)
Sinus congestion	-	1 (0.0)	-	-	-	-	-	1 (0.0)
Sinus disorder	1 (0.0)	-	-	-	-	-	-	1 (0.0)
Sinus headache	-	-	1 (0.0)	-	-	-	-	1 (0.0)
Sinus node dysfunction	5 (0.1)	2 (0.0)	10 (0.0)	1 (0.3)	1 (0.2)	-	-	19 (0.0)
Sinus rhythm	-	-	2 (0.0)	-	-	1 (0.0)	-	3 (0.0)
Sinus tachycardia	5 (0.1)	7 (0.0)	36 (0.1)	-	2 (0.4)	5 (0.1)	1 (0.4)	56 (0.1)
Sinusitis	-	1 (0.0)	1 (0.0)	-	1 (0.2)	-	-	3 (0.0)
Skin abrasion	1 (0.0)	-	2 (0.0)	-	-	-	-	3 (0.0)
Skin burning sensation	-	-	5 (0.0)	-	-	-	-	5 (0.0)
Skin cancer	1 (0.0)	-	-	-	-	-	-	1 (0.0)
Skin discolouration	1 (0.0)	4 (0.0)	13 (0.1)	-	-	2 (0.0)	-	20 (0.0)
Skin disorder	-	7 (0.0)	3 (0.0)	-	-	1 (0.0)	-	11 (0.0)
Skin erosion	-	1 (0.0)	-	-	-	2 (0.0)	-	3 (0.0)
Skin exfoliation	-	2 (0.0)	3 (0.0)	-	-	2 (0.0)	-	7 (0.0)
Skin fissures	-	1 (0.0)	-	-	-	-	-	1 (0.0)
Skin hypertrophy	-	-	1 (0.0)	-	-	-	-	1 (0.0)

Skin infection	1 (0.0)	1 (0.0)	-	-	-	-	-	2 (0.0)
Skin injury	-	-	1 (0.0)	-	-	-	-	1 (0.0)
Skin irritation	-	-	5 (0.0)	-	-	-	-	5 (0.0)
Skin laceration	3 (0.1)	-	2 (0.0)	-	-	-	-	5 (0.0)
Skin lesion	3 (0.1)	4 (0.0)	5 (0.0)	-	-	2 (0.0)	-	14 (0.0)
Skin necrosis	3 (0.1)	4 (0.0)	4 (0.0)	-	-	-	-	11 (0.0)
Skin oedema	-	-	1 (0.0)	-	-	1 (0.0)	-	2 (0.0)
Skin plaque	1 (0.0)	-	-	-	-	1 (0.0)	-	2 (0.0)
Skin reaction	-	1 (0.0)	15 (0.1)	-	-	1 (0.0)	-	17 (0.0)
Skin swelling	-	1 (0.0)	1 (0.0)	-	-	-	-	2 (0.0)
Skin test negative	-	-	3 (0.0)	-	-	1 (0.0)	-	4 (0.0)
Skin test positive	-	-	4 (0.0)	-	-	-	-	4 (0.0)
Skin tightness	-	-	1 (0.0)	-	-	-	-	1 (0.0)
Skin turgor decreased	-	-	1 (0.0)	-	-	-	-	1 (0.0)
Skin ulcer	-	-	1 (0.0)	-	-	-	-	1 (0.0)
Skin warm	1 (0.0)	2 (0.0)	-	-	-	1 (0.0)	-	4 (0.0)
Sleep apnoea syndrome	-	4 (0.0)	-	-	-	-	-	4 (0.0)
Sleep disorder	2 (0.1)	6 (0.0)	9 (0.0)	1 (0.3)	-	-	-	18 (0.0)
Sleep disorder due to a general medical condition	-	1 (0.0)	-	-	-	-	-	1 (0.0)
Sleep terror	-	1 (0.0)	-	-	-	-	-	1 (0.0)
Slow response to stimuli	-	4 (0.0)	1 (0.0)	-	-	-	-	5 (0.0)
Slow speech	-	1 (0.0)	-	-	-	-	-	1 (0.0)
Sluggishness	-	3 (0.0)	1 (0.0)	-	-	-	-	4 (0.0)
Small cell lung cancer	-	1 (0.0)	-	-	-	-	-	1 (0.0)
Small for dates baby	-	-	3 (0.0)	-	2 (0.4)	-	-	5 (0.0)
Small intestinal resection	-	2 (0.0)	-	-	-	-	-	2 (0.0)
Smoke sensitivity	-	1 (0.0)	-	-	-	-	-	1 (0.0)
Sneezing	-	3 (0.0)	4 (0.0)	-	-	-	-	7 (0.0)
Snoring	1 (0.0)	6 (0.0)	1 (0.0)	-	-	3 (0.1)	-	11 (0.0)
Social	-	1 (0.0)	-	-	-	-	-	1 (0.0)
Social avoidant behaviour	-	3 (0.0)	1 (0.0)	-	-	-	-	4 (0.0)
Social fear	-	3 (0.0)	-	-	-	-	-	3 (0.0)

Sodium retention	-	-	8 (0.0)	-	-	-	-	8 (0.0)
Soft tissue injury	-	-	2 (0.0)	-	-	-	-	2 (0.0)
Soft tissue necrosis	-	-	4 (0.0)	-	-	-	-	4 (0.0)
Somatic dysfunction	-	-	1 (0.0)	-	-	-	-	1 (0.0)
Somatic symptom disorder	-	4 (0.0)	-	-	-	-	-	4 (0.0)
Somatosensory evoked potentials abnormal	-	-	5 (0.0)	-	-	-	-	5 (0.0)
Somnambulism	-	10 (0.1)	-	-	-	-	-	10 (0.0)
Somnolence	16 (0.4)	233 (1.6)	60 (0.2)	4 (1.2)	1 (0.2)	10 (0.2)	2 (0.8)	326 (0.7)
Somnolence neonatal	1 (0.0)	1 (0.0)	3 (0.0)	-	-	-	-	5 (0.0)
Sopor	-	11 (0.1)	2 (0.0)	-	-	2 (0.0)	-	15 (0.0)
Spasmodic dysphonia	-	-	2 (0.0)	-	-	-	-	2 (0.0)
Specific gravity urine decreased	1 (0.0)	-	-	-	-	-	-	1 (0.0)
Speech disorder	1 (0.0)	19 (0.1)	10 (0.0)	-	1 (0.2)	1 (0.0)	-	32 (0.1)
Speech disorder developmental	-	-	-	1 (0.3)	-	-	-	1 (0.0)
Sphenoid bone dehiscence	-	-	-	-	1 (0.2)	-	-	1 (0.0)
Sphincter of Oddi dysfunction	-	-	1 (0.0)	-	-	-	-	1 (0.0)
Spina bifida	-	-	1 (0.0)	-	-	-	-	1 (0.0)
Spinal column injury	-	-	-	-	-	1 (0.0)	-	1 (0.0)
Spinal cord compression	-	-	-	-	-	1 (0.0)	-	1 (0.0)
Spinal cord disorder	-	-	3 (0.0)	-	-	-	-	3 (0.0)
Spinal cord infarction	-	-	7 (0.0)	-	-	-	-	7 (0.0)
Spinal cord ischaemia	-	1 (0.0)	-	-	-	-	-	1 (0.0)
Spinal cord oedema	-	1 (0.0)	3 (0.0)	-	-	1 (0.0)	-	5 (0.0)
Spinal disorder	-	3 (0.0)	-	-	-	1 (0.0)	-	4 (0.0)
Spinal fracture	-	-	1 (0.0)	-	-	-	-	1 (0.0)
Spinal pain	-	-	3 (0.0)	-	-	1 (0.0)	-	4 (0.0)
Spinal stenosis	-	1 (0.0)	-	-	-	-	-	1 (0.0)
Splenic abscess	-	1 (0.0)	-	-	-	-	-	1 (0.0)
Splenic haematoma	-	-	1 (0.0)	-	-	-	-	1 (0.0)
Splenic infarction	-	2 (0.0)	-	-	-	-	-	2 (0.0)
Splenic injury	-	-	1 (0.0)	-	-	-	-	1 (0.0)
Splenomegaly	-	2 (0.0)	3 (0.0)	-	-	-	-	5 (0.0)

Splinter	-	-	1 (0.0)	-	-	-	-	1 (0.0)
Spontaneous haemorrhage	-	1 (0.0)	1 (0.0)	-	-	-	-	2 (0.0)
Sputum abnormal	-	-	1 (0.0)	-	-	1 (0.0)	-	2 (0.0)
Sputum discoloured	-	1 (0.0)	-	-	-	-	-	1 (0.0)
Sputum increased	2 (0.1)	-	1 (0.0)	-	-	-	-	3 (0.0)
Sputum purulent	-	1 (0.0)	-	-	-	-	-	1 (0.0)
Sputum retention	-	2 (0.0)	5 (0.0)	1 (0.3)	-	-	-	8 (0.0)
Staphylococcal abscess	-	1 (0.0)	-	-	-	-	-	1 (0.0)
Staphylococcal bacteraemia	1 (0.0)	-	-	-	-	1 (0.0)	-	2 (0.0)
Staphylococcal infection	-	6 (0.0)	3 (0.0)	-	-	1 (0.0)	-	10 (0.0)
Staphylococcal scalded skin syndrome	-	1 (0.0)	-	-	-	-	-	1 (0.0)
Staphylococcus test positive	-	1 (0.0)	2 (0.0)	-	-	-	-	3 (0.0)
Staring	-	3 (0.0)	1 (0.0)	-	-	-	-	4 (0.0)
Status epilepticus	1 (0.0)	84 (0.6)	34 (0.1)	-	-	31 (0.7)	-	150 (0.3)
Stereotypy	-	3 (0.0)	1 (0.0)	1 (0.3)	-	1 (0.0)	-	6 (0.0)
Sternotomy	1 (0.0)	-	-	-	-	-	-	1 (0.0)
Steroid diabetes	-	-	1 (0.0)	-	-	-	-	1 (0.0)
Stevens-Johnson syndrome	-	11 (0.1)	6 (0.0)	-	-	3 (0.1)	-	20 (0.0)
Stillbirth	-	2 (0.0)	2 (0.0)	-	-	3 (0.1)	-	7 (0.0)
Stomatitis	-	-	1 (0.0)	-	-	-	-	1 (0.0)
Strabismus	-	3 (0.0)	-	-	-	-	-	3 (0.0)
Streptococcal infection	-	2 (0.0)	-	-	-	-	-	2 (0.0)
Streptococcal sepsis	-	1 (0.0)	-	-	-	-	-	1 (0.0)
Stress	-	1 (0.0)	-	-	-	-	-	1 (0.0)
Stress cardiomyopathy	1 (0.0)	4 (0.0)	65 (0.3)	1 (0.3)	3 (0.6)	8 (0.2)	-	82 (0.2)
Stridor	3 (0.1)	5 (0.0)	17 (0.1)	-	-	6 (0.1)	-	31 (0.1)
Stroke volume increased	-	-	2 (0.0)	-	-	-	-	2 (0.0)
Stupor	-	8 (0.1)	7 (0.0)	-	-	4 (0.1)	-	19 (0.0)
Subacute combined cord degeneration	-	-	1 (0.0)	-	-	-	-	1 (0.0)
Subacute cutaneous lupus erythematosus	-	-	2 (0.0)	-	-	-	-	2 (0.0)
Subarachnoid haematoma	-	1 (0.0)	-	-	-	-	-	1 (0.0)
Subarachnoid haemorrhage	-	2 (0.0)	3 (0.0)	-	-	-	-	5 (0.0)

Subcutaneous emphysema	1 (0.0)	-	4 (0.0)	-	-	2 (0.0)	-	7 (0.0)
Subcutaneous haematoma	1 (0.0)	-	-	-	-	-	-	1 (0.0)
Subdural effusion	-	1 (0.0)	-	-	-	-	-	1 (0.0)
Subdural haematoma	-	-	1 (0.0)	-	-	-	-	1 (0.0)
Subdural haemorrhage	-	1 (0.0)	-	-	-	-	-	1 (0.0)
Subileus	1 (0.0)	-	-	-	-	1 (0.0)	-	2 (0.0)
Substance abuse	-	11 (0.1)	-	-	1 (0.2)	-	-	12 (0.0)
Substance-induced mood disorder	-	-	-	-	-	1 (0.0)	-	1 (0.0)
Substance-induced psychotic disorder	-	1 (0.0)	1 (0.0)	-	-	-	-	2 (0.0)
Sudden death	-	6 (0.0)	4 (0.0)	-	-	1 (0.0)	-	11 (0.0)
Sudden onset of sleep	-	4 (0.0)	-	-	-	-	-	4 (0.0)
Suffocation feeling	-	2 (0.0)	1 (0.0)	-	-	-	-	3 (0.0)
Suicidal ideation	-	15 (0.1)	2 (0.0)	-	-	1 (0.0)	-	18 (0.0)
Suicide attempt	-	33 (0.2)	16 (0.1)	1 (0.3)	-	-	-	50 (0.1)
Superficial inflammatory dermatosis	-	1 (0.0)	-	-	-	-	-	1 (0.0)
Superficial vein thrombosis	-	1 (0.0)	4 (0.0)	-	-	2 (0.0)	-	7 (0.0)
Superinfection bacterial	1 (0.0)	-	-	-	-	-	-	1 (0.0)
Superior vena cava occlusion	-	1 (0.0)	-	-	-	-	-	1 (0.0)
Superior vena cava syndrome	-	-	1 (0.0)	-	-	-	-	1 (0.0)
Supine hypertension	-	-	-	-	-	1 (0.0)	-	1 (0.0)
Supraventricular extrasystoles	1 (0.0)	2 (0.0)	3 (0.0)	1 (0.3)	-	2 (0.0)	-	9 (0.0)
Supraventricular tachyarrhythmia	-	-	1 (0.0)	-	-	-	-	1 (0.0)
Supraventricular tachycardia	3 (0.1)	9 (0.1)	25 (0.1)	-	-	9 (0.2)	-	46 (0.1)
Surgery	-	-	2 (0.0)	-	1 (0.2)	-	-	3 (0.0)
Suspected counterfeit product	-	3 (0.0)	1 (0.0)	-	-	1 (0.0)	-	5 (0.0)
Suspected product contamination	-	-	11 (0.0)	-	-	1 (0.0)	-	12 (0.0)
Suspected product quality issue	-	2 (0.0)	15 (0.1)	-	-	-	-	17 (0.0)
Suspected product tampering	-	2 (0.0)	-	-	-	-	-	2 (0.0)
Suspected suicide	-	2 (0.0)	2 (0.0)	-	-	-	-	4 (0.0)
Suspected transmission of an infectious agent via product	-	-	2 (0.0)	-	-	-	-	2 (0.0)
Suspiciousness	-	2 (0.0)	-	-	-	-	-	2 (0.0)
Swelling	1 (0.0)	7 (0.0)	30 (0.1)	-	-	3 (0.1)	-	41 (0.1)

Swelling face	-	9 (0.1)	13 (0.1)	-	-	6 (0.1)	-	28 (0.1)
Swelling of eyelid	-	6 (0.0)	3 (0.0)	-	-	1 (0.0)	-	10 (0.0)
Swollen tongue	-	6 (0.0)	14 (0.1)	-	-	3 (0.1)	-	23 (0.0)
Symmetrical drug-related intertriginous and flexural exanthema	-	-	3 (0.0)	-	-	-	-	3 (0.0)
Sympatholysis	1 (0.0)	-	-	-	-	-	-	1 (0.0)
Sympathomimetic effect	1 (0.0)	-	-	-	-	-	-	1 (0.0)
Symptom masked	-	1 (0.0)	4 (0.0)	-	-	-	-	5 (0.0)
Symptom recurrence	-	1 (0.0)	2 (0.0)	-	-	-	-	3 (0.0)
Syncope	15 (0.4)	29 (0.2)	6 (0.0)	-	-	4 (0.1)	-	54 (0.1)
Syndactyly	-	-	-	-	-	2 (0.0)	-	2 (0.0)
Syringe issue	-	5 (0.0)	2 (0.0)	-	-	-	-	7 (0.0)
Systemic candida	1 (0.0)	-	1 (0.0)	-	-	-	-	2 (0.0)
Systemic immune activation	-	-	1 (0.0)	-	-	-	-	1 (0.0)
Systemic infection	-	-	1 (0.0)	-	-	-	-	1 (0.0)
Systemic inflammatory response syndrome	-	2 (0.0)	9 (0.0)	-	1 (0.2)	4 (0.1)	-	16 (0.0)
Systemic lupus erythematosus	-	1 (0.0)	-	-	-	-	-	1 (0.0)
Systemic mastocytosis	-	-	-	-	-	1 (0.0)	-	1 (0.0)
Systemic toxicity	-	-	2 (0.0)	-	-	-	-	2 (0.0)
Systolic anterior motion of mitral valve	-	3 (0.0)	3 (0.0)	-	-	-	-	6 (0.0)
Systolic dysfunction	-	-	1 (0.0)	-	-	1 (0.0)	-	2 (0.0)
Systolic hypertension	-	-	3 (0.0)	-	-	-	-	3 (0.0)
Tachyarrhythmia	-	-	5 (0.0)	-	-	1 (0.0)	-	6 (0.0)
Tachycardia	28 (0.7)	105 (0.7)	357 (1.4)	4 (1.2)	3 (0.6)	77 (1.6)	7 (2.6)	581 (1.2)
Tachycardia foetal	-	-	-	-	-	1 (0.0)	-	1 (0.0)
Tachyphylaxis	-	3 (0.0)	2 (0.0)	2 (0.6)	-	3 (0.1)	-	10 (0.0)
Tachypnoea	8 (0.2)	14 (0.1)	26 (0.1)	-	-	5 (0.1)	8 (3.0)	61 (0.1)
Tardive dyskinesia	-	2 (0.0)	-	-	-	-	-	2 (0.0)
Taste disorder	-	2 (0.0)	3 (0.0)	-	-	-	-	5 (0.0)
Tearfulness	-	1 (0.0)	1 (0.0)	-	-	1 (0.0)	-	3 (0.0)
Temperature intolerance	-	1 (0.0)	-	-	-	-	-	1 (0.0)
Temperature regulation disorder	-	-	6 (0.0)	-	-	-	-	6 (0.0)
Tenderness	-	-	2 (0.0)	-	-	-	-	2 (0.0)

Tension headache	-	-	1 (0.0)	-	-	-	-	1 (0.0)
Terminal insomnia	-	1 (0.0)	-	-	-	-	-	1 (0.0)
Terminal state	-	2 (0.0)	-	-	-	-	-	2 (0.0)
Tetanus	-	2 (0.0)	-	-	-	-	-	2 (0.0)
Tetany	-	1 (0.0)	1 (0.0)	-	-	-	-	2 (0.0)
Tethered cord syndrome	-	1 (0.0)	-	-	-	-	-	1 (0.0)
Theft	-	2 (0.0)	-	-	-	-	-	2 (0.0)
Therapeutic product effect decreased	1 (0.0)	21 (0.1)	56 (0.2)	-	-	-	-	78 (0.2)
Therapeutic product effect delayed	-	10 (0.1)	8 (0.0)	-	-	-	-	18 (0.0)
Therapeutic product effect incomplete	8 (0.2)	30 (0.2)	22 (0.1)	2 (0.6)	5 (1.0)	2 (0.0)	1 (0.4)	70 (0.1)
Therapeutic product effect increased	-	7 (0.0)	1 (0.0)	-	-	-	-	8 (0.0)
Therapeutic product effect prolonged	-	8 (0.1)	7 (0.0)	-	-	-	1 (0.4)	16 (0.0)
Therapeutic product effect variable	-	3 (0.0)	1 (0.0)	-	1 (0.2)	-	-	5 (0.0)
Therapeutic product ineffective	-	-	6 (0.0)	-	-	-	-	6 (0.0)
Therapeutic product ineffective for unapproved indication	-	-	1 (0.0)	-	-	3 (0.1)	-	4 (0.0)
Therapeutic reaction time decreased	-	1 (0.0)	-	-	-	-	-	1 (0.0)
Therapeutic response decreased	3 (0.1)	17 (0.1)	21 (0.1)	-	1 (0.2)	1 (0.0)	-	43 (0.1)
Therapeutic response delayed	-	1 (0.0)	1 (0.0)	-	-	1 (0.0)	-	3 (0.0)
Therapeutic response prolonged	-	-	2 (0.0)	-	-	-	-	2 (0.0)
Therapeutic response shortened	-	1 (0.0)	1 (0.0)	-	-	-	-	2 (0.0)
Therapeutic response unexpected	2 (0.1)	5 (0.0)	3 (0.0)	-	-	-	-	10 (0.0)
Therapy cessation	-	2 (0.0)	-	-	-	1 (0.0)	-	3 (0.0)
Therapy change	-	8 (0.1)	3 (0.0)	-	-	-	-	11 (0.0)
Therapy non-responder	2 (0.1)	11 (0.1)	19 (0.1)	-	-	6 (0.1)	-	38 (0.1)
Therapy partial responder	2 (0.1)	-	-	-	-	-	-	2 (0.0)
Thermal burn	-	-	1 (0.0)	-	-	-	-	1 (0.0)
Thinking abnormal	-	3 (0.0)	1 (0.0)	-	-	-	-	4 (0.0)
Thirst	-	4 (0.0)	6 (0.0)	-	-	-	-	10 (0.0)
Thoracic haemorrhage	-	-	-	-	-	1 (0.0)	-	1 (0.0)
Throat irritation	-	-	1 (0.0)	-	-	1 (0.0)	-	2 (0.0)
Throat tightness	-	8 (0.1)	3 (0.0)	-	-	1 (0.0)	-	12 (0.0)
Thrombocytopenia	2 (0.1)	24 (0.2)	28 (0.1)	-	2 (0.4)	11 (0.2)	-	67 (0.1)

Thrombocytopenia neonatal	-	-	1 (0.0)	-	-	-	-	1 (0.0)
Thrombocytosis	-	2 (0.0)	-	-	-	-	-	2 (0.0)
Thrombophlebitis	-	3 (0.0)	13 (0.1)	-	-	1 (0.0)	-	17 (0.0)
Thrombophlebitis septic	1 (0.0)	1 (0.0)	-	-	-	-	-	2 (0.0)
Thrombosis	-	-	9 (0.0)	-	-	3 (0.1)	-	12 (0.0)
Thrombosis in device	1 (0.0)	-	5 (0.0)	-	-	-	-	6 (0.0)
Thrombotic microangiopathy	-	1 (0.0)	1 (0.0)	-	-	5 (0.1)	-	7 (0.0)
Thrombotic thrombocytopenic purpura	-	-	2 (0.0)	-	-	-	-	2 (0.0)
Thyroid cyst	-	-	-	-	-	1 (0.0)	-	1 (0.0)
Thyroid disorder	-	3 (0.0)	-	-	-	-	-	3 (0.0)
Thyroid function test abnormal	-	-	1 (0.0)	-	-	1 (0.0)	-	2 (0.0)
Thyrotoxic crisis	-	-	3 (0.0)	-	1 (0.2)	2 (0.0)	-	6 (0.0)
Tic	-	1 (0.0)	2 (0.0)	-	-	-	-	3 (0.0)
Tidal volume decreased	-	-	-	-	1 (0.2)	-	-	1 (0.0)
Time perception altered	-	-	1 (0.0)	-	-	-	-	1 (0.0)
Tinnitus	-	7 (0.0)	7 (0.0)	-	-	1 (0.0)	-	15 (0.0)
Tissue infiltration	-	-	1 (0.0)	-	-	-	-	1 (0.0)
Tissue irritation	-	-	2 (0.0)	-	-	-	-	2 (0.0)
Tobacco user	-	1 (0.0)	-	-	-	-	-	1 (0.0)
Tocolysis	-	-	-	-	-	1 (0.0)	-	1 (0.0)
Tongue biting	-	2 (0.0)	-	-	-	-	-	2 (0.0)
Tongue coated	-	-	1 (0.0)	-	-	-	-	1 (0.0)
Tongue discomfort	-	-	1 (0.0)	-	-	-	-	1 (0.0)
Tongue disorder	1 (0.0)	3 (0.0)	1 (0.0)	-	1 (0.2)	-	-	6 (0.0)
Tongue injury	-	-	1 (0.0)	-	-	-	-	1 (0.0)
Tongue movement disturbance	-	-	1 (0.0)	-	-	1 (0.0)	-	2 (0.0)
Tongue oedema	-	5 (0.0)	18 (0.1)	-	1 (0.2)	3 (0.1)	-	27 (0.1)
Tonic clonic movements	-	4 (0.0)	14 (0.1)	-	-	5 (0.1)	-	23 (0.0)
Tonic convulsion	1 (0.0)	12 (0.1)	9 (0.0)	-	-	2 (0.0)	-	24 (0.0)
Tonic posturing	-	-	2 (0.0)	-	-	-	-	2 (0.0)
Tonsillar hypertrophy	-	1 (0.0)	-	-	-	-	-	1 (0.0)
Tonsillectomy	-	-	1 (0.0)	-	-	-	-	1 (0.0)

Tonsillitis	-	-	1 (0.0)	-	-	-	-	1 (0.0)
Tooth discolouration	-	2 (0.0)	1 (0.0)	-	-	-	-	3 (0.0)
Tooth fracture	-	-	-	-	-	1 (0.0)	-	1 (0.0)
Toothache	-	2 (0.0)	1 (0.0)	-	-	-	-	3 (0.0)
Torsade de pointes	10 (0.3)	5 (0.0)	25 (0.1)	-	-	3 (0.1)	-	43 (0.1)
Torticollis	-	1 (0.0)	8 (0.0)	-	-	-	-	9 (0.0)
Total lung capacity decreased	-	-	1 (0.0)	-	-	-	-	1 (0.0)
Toxic cardiomyopathy	-	1 (0.0)	-	-	-	-	-	1 (0.0)
Toxic encephalopathy	-	3 (0.0)	2 (0.0)	-	-	1 (0.0)	-	6 (0.0)
Toxic epidermal necrolysis	1 (0.0)	15 (0.1)	9 (0.0)	-	2 (0.4)	1 (0.0)	-	28 (0.1)
Toxic leukoencephalopathy	-	-	-	1 (0.3)	-	-	-	1 (0.0)
Toxic shock syndrome	-	-	13 (0.1)	-	-	-	-	13 (0.0)
Toxic skin eruption	-	7 (0.0)	5 (0.0)	-	-	3 (0.1)	-	15 (0.0)
Toxicity to various agents	5 (0.1)	131 (0.9)	88 (0.4)	2 (0.6)	1 (0.2)	15 (0.3)	1 (0.4)	243 (0.5)
Tracheal disorder	-	1 (0.0)	-	-	-	-	-	1 (0.0)
Tracheal haemorrhage	1 (0.0)	-	-	-	-	-	-	1 (0.0)
Tracheal injury	-	-	1 (0.0)	-	-	1 (0.0)	-	2 (0.0)
Tracheal oedema	-	-	2 (0.0)	-	-	-	-	2 (0.0)
Tracheal pain	-	-	1 (0.0)	-	-	-	-	1 (0.0)
Tracheal stenosis	-	1 (0.0)	1 (0.0)	-	-	-	-	2 (0.0)
Tracheitis	1 (0.0)	-	-	-	-	-	-	1 (0.0)
Tracheobronchitis	1 (0.0)	1 (0.0)	1 (0.0)	-	-	-	-	3 (0.0)
Tracheostomy	1 (0.0)	-	-	-	-	-	-	1 (0.0)
Trance	-	-	1 (0.0)	-	-	-	-	1 (0.0)
Transaminases increased	-	4 (0.0)	22 (0.1)	-	-	3 (0.1)	-	29 (0.1)
Transcranial electrical motor evoked potential monitoring abnormal	2 (0.1)	-	11 (0.0)	-	1 (0.2)	-	-	14 (0.0)
Transfusion	-	-	1 (0.0)	-	-	-	-	1 (0.0)
Transient global amnesia	-	-	1 (0.0)	-	-	-	-	1 (0.0)
Transient ischaemic attack	1 (0.0)	2 (0.0)	3 (0.0)	-	-	-	-	6 (0.0)
Transient psychosis	-	-	-	-	-	-	1 (0.4)	1 (0.0)
Transmission of an infectious agent via product	-	-	4 (0.0)	-	-	-	-	4 (0.0)
Transposition of the great vessels	-	-	-	-	1 (0.2)	-	-	1 (0.0)

Transurethral resection syndrome	-	-	1 (0.0)	-	-	-	-	1 (0.0)
Traumatic haemothorax	-	1 (0.0)	-	-	-	-	-	1 (0.0)
Traumatic lung injury	-	-	1 (0.0)	-	-	-	-	1 (0.0)
Treatment delayed	-	-	1 (0.0)	-	-	-	-	1 (0.0)
Treatment failure	-	23 (0.2)	2 (0.0)	-	-	7 (0.1)	-	32 (0.1)
Treatment noncompliance	-	1 (0.0)	8 (0.0)	-	-	-	-	9 (0.0)
Tremor	3 (0.1)	44 (0.3)	77 (0.3)	1 (0.3)	-	14 (0.3)	-	139 (0.3)
Trichorrhexis	-	-	2 (0.0)	-	-	-	-	2 (0.0)
Trichotillomania	-	-	-	-	-	1 (0.0)	-	1 (0.0)
Tricuspid valve incompetence	1 (0.0)	-	1 (0.0)	-	-	-	-	2 (0.0)
Trigeminal nerve disorder	-	-	-	-	-	1 (0.0)	-	1 (0.0)
Trigeminal neuralgia	-	1 (0.0)	-	-	-	-	-	1 (0.0)
Trigemino-cardiac reflex	1 (0.0)	-	7 (0.0)	-	1 (0.2)	2 (0.0)	-	11 (0.0)
Tri-iodothyronine free decreased	-	-	-	-	-	1 (0.0)	-	1 (0.0)
Trismus	1 (0.0)	4 (0.0)	16 (0.1)	-	-	4 (0.1)	-	25 (0.1)
Trisomy 21	-	1 (0.0)	-	-	-	-	-	1 (0.0)
Troponin I increased	-	-	6 (0.0)	-	-	-	-	6 (0.0)
Troponin increased	-	6 (0.0)	8 (0.0)	-	-	5 (0.1)	-	19 (0.0)
Troponin T increased	-	1 (0.0)	3 (0.0)	-	-	-	-	4 (0.0)
Tryptase decreased	-	-	1 (0.0)	-	-	-	-	1 (0.0)
Tryptase increased	-	2 (0.0)	5 (0.0)	-	-	3 (0.1)	-	10 (0.0)
Tuberous sclerosis complex	-	-	1 (0.0)	-	-	-	-	1 (0.0)
Tubulointerstitial nephritis	-	2 (0.0)	1 (0.0)	-	-	-	-	3 (0.0)
Tumour haemorrhage	1 (0.0)	-	2 (0.0)	-	-	-	-	3 (0.0)
Tumour lysis syndrome	-	1 (0.0)	2 (0.0)	-	-	-	-	3 (0.0)
Tunnel vision	-	1 (0.0)	-	-	-	1 (0.0)	-	2 (0.0)
Twin pregnancy	-	1 (0.0)	2 (0.0)	-	-	-	-	3 (0.0)
Tympanic membrane perforation	-	-	1 (0.0)	-	-	-	-	1 (0.0)
Type I hypersensitivity	-	9 (0.1)	82 (0.3)	-	1 (0.2)	9 (0.2)	-	101 (0.2)
Type III immune complex mediated reaction	-	-	3 (0.0)	-	-	1 (0.0)	-	4 (0.0)
Type IV hypersensitivity reaction	-	1 (0.0)	5 (0.0)	-	-	1 (0.0)	-	7 (0.0)
Ulcer	-	3 (0.0)	1 (0.0)	-	-	1 (0.0)	-	5 (0.0)

Ulcer haemorrhage	1 (0.0)	-	-	-	-	-	-	1 (0.0)
Ulcerative keratitis	-	-	1 (0.0)	-	-	-	-	1 (0.0)
Ulna fracture	1 (0.0)	1 (0.0)	-	-	-	-	-	2 (0.0)
Ultrasound ovary abnormal	-	-	-	-	-	1 (0.0)	-	1 (0.0)
Underdose	-	6 (0.0)	12 (0.0)	-	-	-	-	18 (0.0)
Unevaluable event	-	7 (0.0)	3 (0.0)	-	-	1 (0.0)	1 (0.4)	12 (0.0)
Unilateral bronchospasm	-	-	3 (0.0)	-	-	-	-	3 (0.0)
Univentricular heart	-	-	1 (0.0)	-	-	-	-	1 (0.0)
Unmasking of previously unidentified disease	-	2 (0.0)	8 (0.0)	-	-	1 (0.0)	-	11 (0.0)
Unresponsive to stimuli	11 (0.3)	82 (0.5)	46 (0.2)	1 (0.3)	-	12 (0.3)	1 (0.4)	153 (0.3)
Unwanted awareness during anaesthesia	-	3 (0.0)	16 (0.1)	-	-	3 (0.1)	-	22 (0.0)
Upper airway obstruction	6 (0.2)	7 (0.0)	13 (0.1)	5 (1.5)	-	4 (0.1)	4 (1.5)	39 (0.1)
Upper gastrointestinal haemorrhage	-	3 (0.0)	1 (0.0)	-	-	-	-	4 (0.0)
Upper limb fracture	-	-	1 (0.0)	-	-	-	-	1 (0.0)
Upper respiratory tract infection	-	3 (0.0)	2 (0.0)	-	1 (0.2)	-	-	6 (0.0)
Ureterolithiasis	-	1 (0.0)	-	-	-	-	-	1 (0.0)
Urethral injury	-	1 (0.0)	-	-	-	-	-	1 (0.0)
Urethral stenosis	-	1 (0.0)	-	-	-	-	-	1 (0.0)
Urinary ascites	-	-	-	-	-	-	1 (0.4)	1 (0.0)
Urinary bladder rupture	-	1 (0.0)	-	-	-	-	1 (0.4)	2 (0.0)
Urinary hesitation	-	2 (0.0)	-	-	-	-	-	2 (0.0)
Urinary incontinence	2 (0.1)	3 (0.0)	4 (0.0)	1 (0.3)	-	-	-	10 (0.0)
Urinary retention	4 (0.1)	12 (0.1)	9 (0.0)	-	-	4 (0.1)	-	29 (0.1)
Urinary retention postoperative	-	1 (0.0)	-	-	-	-	-	1 (0.0)
Urinary sediment abnormal	-	-	1 (0.0)	-	-	-	-	1 (0.0)
Urinary tract candidiasis	-	2 (0.0)	-	-	-	-	-	2 (0.0)
Urinary tract infection	1 (0.0)	5 (0.0)	3 (0.0)	-	1 (0.2)	1 (0.0)	-	11 (0.0)
Urinary tract infection enterococcal	-	-	2 (0.0)	-	-	-	-	2 (0.0)
Urine abnormality	-	-	5 (0.0)	-	-	-	-	5 (0.0)
Urine alcohol test positive	1 (0.0)	-	-	-	-	-	-	1 (0.0)
Urine analysis abnormal	-	-	6 (0.0)	-	-	-	-	6 (0.0)
Urine osmolarity	1 (0.0)	-	-	-	-	-	-	1 (0.0)

Urine osmolarity decreased	-	-	1 (0.0)	-	-	-	-	1 (0.0)
Urine osmolarity increased	-	-	-	-	1 (0.2)	-	-	1 (0.0)
Urine output decreased	-	6 (0.0)	12 (0.0)	-	-	-	-	18 (0.0)
Urine output increased	1 (0.0)	-	1 (0.0)	-	-	-	-	2 (0.0)
Urine sodium increased	-	-	1 (0.0)	-	1 (0.2)	-	-	2 (0.0)
Urine uric acid increased	-	-	1 (0.0)	-	-	-	-	1 (0.0)
Urobilinogen urine increased	-	1 (0.0)	-	-	-	-	-	1 (0.0)
Urosepsis	-	-	-	-	-	1 (0.0)	-	1 (0.0)
Urticaria	3 (0.1)	84 (0.6)	289 (1.2)	1 (0.3)	1 (0.2)	56 (1.2)	1 (0.4)	435 (0.9)
Urticaria chronic	-	-	1 (0.0)	-	-	-	-	1 (0.0)
Use of accessory respiratory muscles	2 (0.1)	3 (0.0)	-	-	-	-	-	5 (0.0)
Uterine atony	-	-	3 (0.0)	-	-	-	-	3 (0.0)
Uterine disorder	-	1 (0.0)	-	-	-	-	-	1 (0.0)
Uterine haemorrhage	-	1 (0.0)	-	-	-	-	-	1 (0.0)
Uterine hypertonus	-	-	1 (0.0)	-	-	-	-	1 (0.0)
Uterine hypotonus	-	-	2 (0.0)	-	-	-	-	2 (0.0)
Uterine rupture	-	-	1 (0.0)	-	-	-	-	1 (0.0)
Uvulitis	-	-	2 (0.0)	-	-	-	-	2 (0.0)
Vaccination failure	-	1 (0.0)	-	-	-	-	-	1 (0.0)
Vaginal discharge	-	14 (0.1)	1 (0.0)	-	-	-	-	15 (0.0)
Vaginal flatulence	-	13 (0.1)	-	-	-	-	-	13 (0.0)
Vaginal haemorrhage	-	1 (0.0)	3 (0.0)	-	-	1 (0.0)	1 (0.4)	6 (0.0)
Valsalva maneuver	-	-	1 (0.0)	-	-	-	-	1 (0.0)
Varicella	-	-	1 (0.0)	-	-	-	-	1 (0.0)
Varicella meningitis	1 (0.0)	-	-	-	-	-	-	1 (0.0)
Varicose vein ruptured	-	1 (0.0)	-	-	-	-	-	1 (0.0)
Vascular access site erythema	-	-	-	-	-	1 (0.0)	-	1 (0.0)
Vascular access site haemorrhage	-	-	1 (0.0)	-	-	-	-	1 (0.0)
Vascular access site rupture	-	-	-	-	-	1 (0.0)	-	1 (0.0)
Vascular compression	-	1 (0.0)	-	-	-	-	-	1 (0.0)
Vascular device occlusion	1 (0.0)	-	-	-	-	-	-	1 (0.0)
Vascular malformation	-	-	1 (0.0)	-	-	-	-	1 (0.0)

Vascular pain	-	-	2 (0.0)	-	-	-	-	2 (0.0)
Vascular pseudoaneurysm	1 (0.0)	-	1 (0.0)	-	-	-	-	2 (0.0)
Vascular purpura	-	-	2 (0.0)	-	-	-	-	2 (0.0)
Vascular resistance pulmonary increased	4 (0.1)	-	-	-	-	-	-	4 (0.0)
Vascular resistance systemic decreased	-	1 (0.0)	1 (0.0)	-	-	-	-	2 (0.0)
Vascular skin disorder	-	-	1 (0.0)	-	-	-	-	1 (0.0)
Vascular stent thrombosis	-	-	1 (0.0)	-	-	-	-	1 (0.0)
Vasculitis	1 (0.0)	-	-	-	-	-	-	1 (0.0)
Vasculitis necrotising	-	1 (0.0)	-	-	-	-	-	1 (0.0)
Vasoconstriction	2 (0.1)	-	7 (0.0)	-	-	-	-	9 (0.0)
Vasodilatation	-	1 (0.0)	9 (0.0)	-	-	1 (0.0)	-	11 (0.0)
Vasoplegia syndrome	-	2 (0.0)	16 (0.1)	-	-	2 (0.0)	-	20 (0.0)
Vasospasm	-	2 (0.0)	4 (0.0)	-	-	1 (0.0)	-	7 (0.0)
Vein collapse	1 (0.0)	-	3 (0.0)	-	-	-	-	4 (0.0)
Vein discolouration	-	1 (0.0)	1 (0.0)	-	-	-	-	2 (0.0)
Vein disorder	-	-	1 (0.0)	-	-	1 (0.0)	-	2 (0.0)
Vein rupture	-	-	-	-	-	1 (0.0)	-	1 (0.0)
Vena cava embolism	-	-	1 (0.0)	-	-	-	-	1 (0.0)
Vena cava thrombosis	-	-	2 (0.0)	-	-	-	-	2 (0.0)
Venoocclusive disease	-	1 (0.0)	-	-	-	-	-	1 (0.0)
Venoocclusive liver disease	-	1 (0.0)	-	-	-	-	-	1 (0.0)
Venous injury	-	-	-	-	-	1 (0.0)	-	1 (0.0)
Venous occlusion	-	-	-	-	-	1 (0.0)	-	1 (0.0)
Venous oxygen saturation decreased	-	-	1 (0.0)	-	-	1 (0.0)	-	2 (0.0)
Venous pressure decreased	-	-	1 (0.0)	-	-	-	-	1 (0.0)
Venous pressure jugular increased	-	-	-	-	-	1 (0.0)	-	1 (0.0)
Venous stenosis	1 (0.0)	-	-	-	-	-	-	1 (0.0)
Venous thrombosis	-	1 (0.0)	5 (0.0)	-	-	1 (0.0)	-	7 (0.0)
Venous thrombosis limb	-	-	1 (0.0)	-	-	-	-	1 (0.0)
Venous valve ruptured	-	-	-	-	-	1 (0.0)	-	1 (0.0)
Ventilation perfusion mismatch	-	-	1 (0.0)	-	-	-	-	1 (0.0)
Ventricular arrhythmia	2 (0.1)	1 (0.0)	13 (0.1)	-	-	1 (0.0)	-	17 (0.0)

Ventricular asystole	2 (0.1)	-	6 (0.0)	-	-	-	-	8 (0.0)
Ventricular dysfunction	-	1 (0.0)	3 (0.0)	-	-	-	-	4 (0.0)
Ventricular dyskinesia	-	-	3 (0.0)	-	-	-	-	3 (0.0)
Ventricular dyssynchrony	-	-	1 (0.0)	-	-	-	-	1 (0.0)
Ventricular extrasystoles	6 (0.2)	13 (0.1)	25 (0.1)	-	-	8 (0.2)	-	52 (0.1)
Ventricular fibrillation	16 (0.4)	14 (0.1)	78 (0.3)	1 (0.3)	2 (0.4)	9 (0.2)	-	120 (0.2)
Ventricular flutter	-	-	1 (0.0)	-	-	-	-	1 (0.0)
Ventricular hypertrophy	-	-	1 (0.0)	-	-	-	-	1 (0.0)
Ventricular hypokinesia	1 (0.0)	2 (0.0)	10 (0.0)	-	1 (0.2)	5 (0.1)	-	19 (0.0)
Ventricular septal defect	-	-	1 (0.0)	-	-	-	-	1 (0.0)
Ventricular tachyarrhythmia	-	-	1 (0.0)	-	-	-	-	1 (0.0)
Ventricular tachycardia	13 (0.3)	11 (0.1)	87 (0.3)	-	2 (0.4)	13 (0.3)	-	126 (0.3)
Vertebrobasilar insufficiency	1 (0.0)	-	-	-	-	-	-	1 (0.0)
Vertebroplasty	-	-	1 (0.0)	-	-	-	-	1 (0.0)
Vertigo	-	8 (0.1)	6 (0.0)	-	-	1 (0.0)	-	15 (0.0)
Vessel puncture site inflammation	-	-	1 (0.0)	-	-	-	-	1 (0.0)
Vestibular disorder	-	-	1 (0.0)	-	-	-	-	1 (0.0)
Victim of chemical submission	-	-	-	-	-	1 (0.0)	-	1 (0.0)
Victim of child abuse	-	1 (0.0)	-	-	-	-	-	1 (0.0)
Victim of homicide	-	2 (0.0)	-	-	-	1 (0.0)	-	3 (0.0)
Victim of sexual abuse	-	-	-	-	-	1 (0.0)	-	1 (0.0)
VIIth nerve injury	-	-	1 (0.0)	-	-	-	-	1 (0.0)
Viral haemorrhagic cystitis	-	-	-	1 (0.3)	-	-	-	1 (0.0)
Viral infection	-	1 (0.0)	-	-	-	-	-	1 (0.0)
Viral myocarditis	1 (0.0)	-	-	-	-	-	-	1 (0.0)
Viral upper respiratory tract infection	-	-	1 (0.0)	-	-	-	-	1 (0.0)
Visceral congestion	-	-	-	-	-	1 (0.0)	-	1 (0.0)
Vision blurred	-	6 (0.0)	5 (0.0)	-	-	-	-	11 (0.0)
Visual acuity reduced	-	2 (0.0)	1 (0.0)	-	-	2 (0.0)	-	5 (0.0)
Visual field defect	-	2 (0.0)	-	-	-	-	-	2 (0.0)
Visual impairment	-	15 (0.1)	10 (0.0)	-	-	-	-	25 (0.1)
Vital capacity abnormal	-	-	1 (0.0)	-	-	-	-	1 (0.0)

Vital functions abnormal	-	2 (0.0)	2 (0.0)	-	-	-	-	4 (0.0)
Vitamin B12 deficiency	-	2 (0.0)	-	-	-	-	-	2 (0.0)
VIth nerve paralysis	-	1 (0.0)	-	-	-	-	-	1 (0.0)
Vitreous haemorrhage	-	-	1 (0.0)	-	-	-	-	1 (0.0)
Vocal cord disorder	-	1 (0.0)	1 (0.0)	-	-	1 (0.0)	-	3 (0.0)
Vocal cord dysfunction	-	-	2 (0.0)	-	-	-	-	2 (0.0)
Vocal cord paralysis	-	-	7 (0.0)	-	-	-	-	7 (0.0)
Vocal cord paresis	-	-	1 (0.0)	-	-	-	-	1 (0.0)
Vocal cord thickening	1 (0.0)	-	-	-	-	-	-	1 (0.0)
Volume blood decreased	-	-	1 (0.0)	-	-	-	-	1 (0.0)
Volvulus	-	-	3 (0.0)	-	-	-	-	3 (0.0)
Vomiting	15 (0.4)	123 (0.8)	234 (0.9)	1 (0.3)	5 (1.0)	35 (0.7)	-	413 (0.8)
Vth nerve injury	-	-	1 (0.0)	-	-	-	-	1 (0.0)
Vulval abscess	-	-	3 (0.0)	-	-	-	-	3 (0.0)
Vulval disorder	-	1 (0.0)	-	-	-	-	-	1 (0.0)
Vulvar erosion	-	1 (0.0)	-	-	-	-	-	1 (0.0)
Vulvovaginal burning sensation	-	-	-	-	-	1 (0.0)	-	1 (0.0)
Walking aid user	-	-	1 (0.0)	-	-	-	-	1 (0.0)
Walking disability	-	-	1 (0.0)	-	-	-	-	1 (0.0)
Wandering pacemaker	1 (0.0)	-	1 (0.0)	-	-	-	-	2 (0.0)
Waxy flexibility	-	-	1 (0.0)	-	-	-	-	1 (0.0)
Weaning failure	2 (0.1)	3 (0.0)	1 (0.0)	-	-	-	-	6 (0.0)
Weight bearing difficulty	-	-	1 (0.0)	-	-	-	-	1 (0.0)
Weight decreased	-	27 (0.2)	20 (0.1)	-	-	3 (0.1)	-	50 (0.1)
Weight gain poor	-	1 (0.0)	-	1 (0.3)	-	-	-	2 (0.0)
Weight increased	-	12 (0.1)	5 (0.0)	-	-	2 (0.0)	-	19 (0.0)
Wheelchair user	-	-	1 (0.0)	-	-	-	-	1 (0.0)
Wheezing	-	9 (0.1)	35 (0.1)	-	-	11 (0.2)	-	55 (0.1)
White blood cell count abnormal	-	-	-	1 (0.3)	-	-	-	1 (0.0)
White blood cell count decreased	5 (0.1)	2 (0.0)	5 (0.0)	-	-	-	-	12 (0.0)
White blood cell count increased	-	4 (0.0)	7 (0.0)	-	-	1 (0.0)	-	12 (0.0)
White matter lesion	-	5 (0.0)	2 (0.0)	-	-	-	-	7 (0.0)

Withdrawal catatonia	-	2 (0.0)	-	-	-	-	-	2 (0.0)
Withdrawal hypertension	5 (0.1)	-	-	-	-	-	-	5 (0.0)
Withdrawal syndrome	22 (0.6)	65 (0.4)	10 (0.0)	14 (4.2)	2 (0.4)	5 (0.1)	7 (2.6)	125 (0.3)
Wound	-	4 (0.0)	4 (0.0)	-	-	1 (0.0)	-	9 (0.0)
Wound haemorrhage	-	-	1 (0.0)	-	-	-	-	1 (0.0)
Wound infection	-	-	2 (0.0)	-	-	-	-	2 (0.0)
Wound secretion	-	-	1 (0.0)	-	-	-	-	1 (0.0)
Wrist deformity	-	1 (0.0)	-	-	-	-	-	1 (0.0)
Wrong dosage formulation	1 (0.0)	-	-	-	-	-	-	1 (0.0)
Wrong dose	1 (0.0)	3 (0.0)	1 (0.0)	-	-	-	-	5 (0.0)
Wrong drug	2 (0.1)	2 (0.0)	1 (0.0)	-	-	-	-	5 (0.0)
Wrong patient	-	2 (0.0)	-	-	-	-	-	2 (0.0)
Wrong patient received product	-	4 (0.0)	1 (0.0)	-	-	-	-	5 (0.0)
Wrong product administered	9 (0.2)	24 (0.2)	12 (0.0)	-	-	1 (0.0)	-	46 (0.1)
Wrong rate	-	10 (0.1)	1 (0.0)	-	-	-	-	11 (0.0)
Wrong schedule	-	1 (0.0)	-	-	-	-	-	1 (0.0)
Wrong strength	-	4 (0.0)	-	-	-	-	-	4 (0.0)
Wrong technique in product usage process	3 (0.1)	20 (0.1)	15 (0.1)	-	-	1 (0.0)	-	39 (0.1)
X-ray abnormal	-	1 (0.0)	-	-	-	-	-	1 (0.0)
Yawning	-	-	1 (0.0)	-	-	-	-	1 (0.0)

DEX: dexmedetomidine; MID: midazolam; PRO: propofol; DEX/MID: combinations of dexmedetomidine and midazolam; DEX/PRO: combinations of dexmedetomidine and propofol; MID/PRO: combinations of midazolam and propofol; DEX/MID/PRO: combinations of dexmedetomidine, midazolam and propofol.

Supplementary Figure S2. Reporting odds ratio (ROR) of a) rhabdomyolysis and b) all PT indicative of rhabdomyolysis for the subgroup analysis. CI, confidence interval.

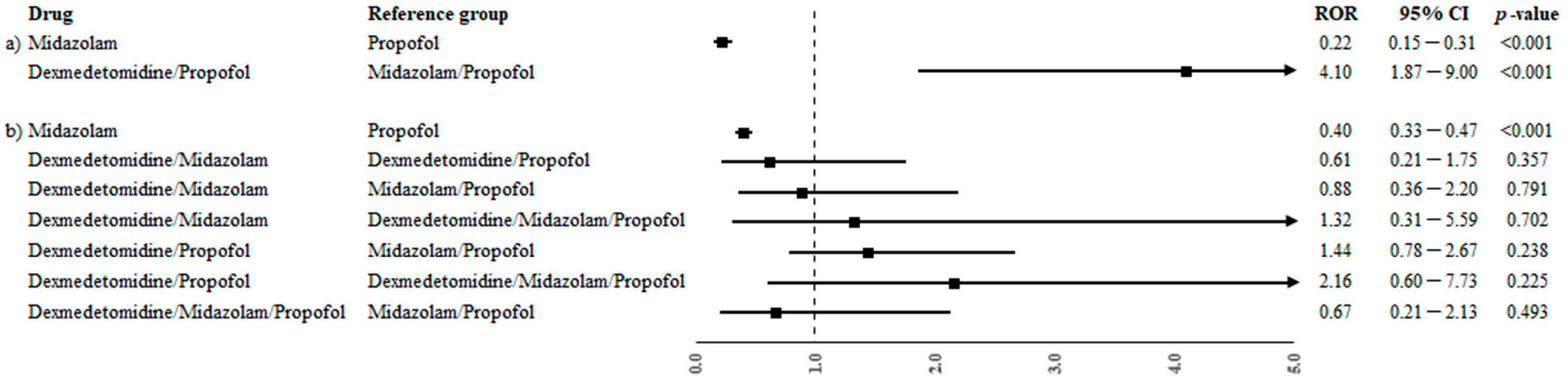

Supplementary Figure S3. Reporting odds ratio (ROR) of a) rhabdomyolysis and b) all PT indicative of rhabdomyolysis between gender (male vs. female). CI, confidence interval.

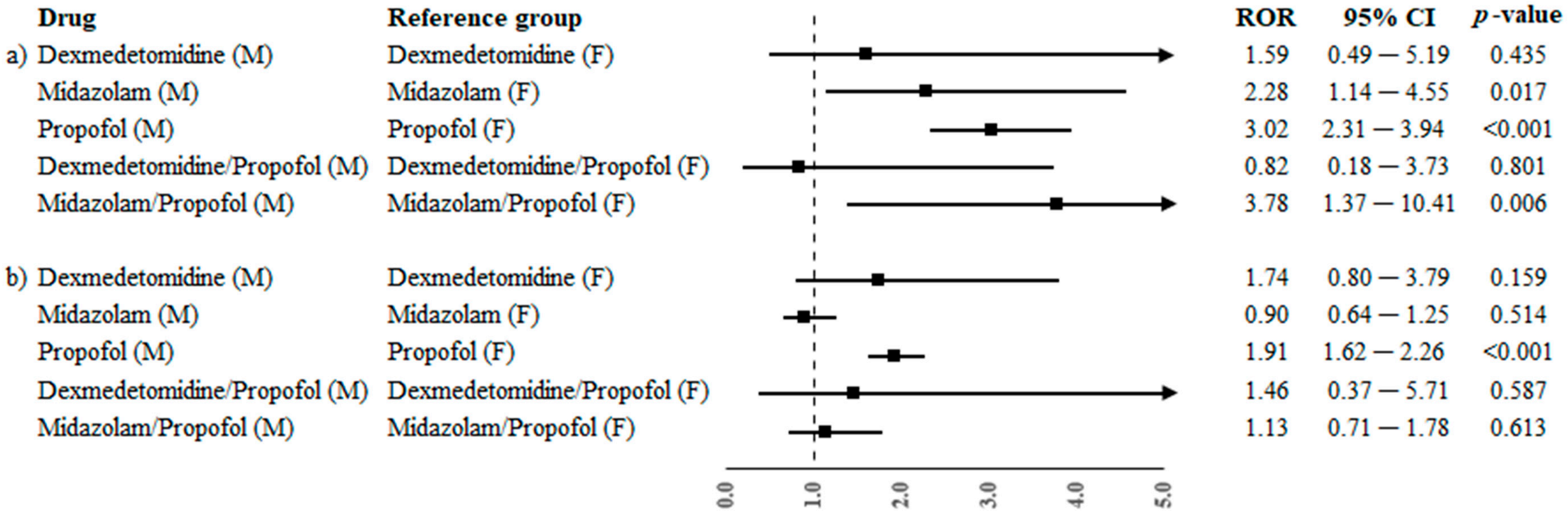

Supplement: Supplementary file 1 [file jpm-14-00961-s001.zip › jpm-3168079-supplementary.pdf]
